# VENNTURE–A Novel Venn Diagram Investigational Tool for Multiple Pharmacological Dataset Analysis

**DOI:** 10.1371/journal.pone.0036911

**Published:** 2012-05-14

**Authors:** Bronwen Martin, Wayne Chadwick, Tie Yi, Sung-Soo Park, Daoyuan Lu, Bin Ni, Shekhar Gadkaree, Kathleen Farhang, Kevin G. Becker, Stuart Maudsley

**Affiliations:** 1 Metabolism Unit, Laboratory of Clinical Investigation, National Institute on Aging, National Institutes of Health, Baltimore, Maryland, United States of America; 2 Receptor Pharmacology Unit, Laboratory of Neuroscience, National Institute on Aging, National Institutes of Health, Baltimore, Maryland, United States of America; 3 Diabetes Section, Laboratory of Clinical Investigation, National Institute on Aging, National Institutes of Health, Baltimore, Maryland, United States of America; 4 Gene Expression and Genomics Unit, Research Resources Branch, National Institute on Aging, National Institutes of Health, Baltimore, Maryland, United States of America; University College Dublin, Ireland

## Abstract

As pharmacological data sets become increasingly large and complex, new visual analysis and filtering programs are needed to aid their appreciation. One of the most commonly used methods for visualizing biological data is the Venn diagram. Currently used Venn analysis software often presents multiple problems to biological scientists, in that only a limited number of simultaneous data sets can be analyzed. An improved appreciation of the connectivity between multiple, highly-complex datasets is crucial for the next generation of data analysis of genomic and proteomic data streams. We describe the development of VENNTURE, a program that facilitates visualization of up to six datasets in a user-friendly manner. This program includes versatile output features, where grouped data points can be easily exported into a spreadsheet. To demonstrate its unique experimental utility we applied VENNTURE to a highly complex parallel paradigm, *i.e*. comparison of multiple G protein-coupled receptor drug dose phosphoproteomic data, in multiple cellular physiological contexts. VENNTURE was able to reliably and simply dissect six complex data sets into easily identifiable groups for straightforward analysis and data output. Applied to complex pharmacological datasets, VENNTURE’s improved features and ease of analysis are much improved over currently available Venn diagram programs. VENNTURE enabled the delineation of highly complex patterns of dose-dependent G protein-coupled receptor activity and its dependence on physiological cellular contexts. This study highlights the potential for such a program in fields such as pharmacology, genomics, and bioinformatics.

## Introduction

As our knowledge of the inter-connected complexity of physiological, cellular and molecular functions expands, there is an ever increasing need for novel methods to study multiple datasets simultaneously. One of the most common methods for data set analysis is the Venn diagram. These typically consist of multiple intersecting regions (usually circles), that allow analysis of all logical relationships between different data sets [1]. With Venn diagram analysis, the set-unique and set-intersection values can be easily illustrated for the viewer. For more than three data sets, the structure of the sets and intersections can however start to become problematical from a visualization standpoint, *i.e.* usable representation of all overlapping regions and Venn symmetry can often be lost. Venn himself generated visualization platforms that facilitate simultaneous analysis of higher numbers of sets, by using additional successive ellipses that intersect with the primary circles. He also gave a construction for Venn diagrams for any number of sets, where each successive curve delimiting a set is interleaved with previous curves, starting with the 3-circle diagram [1]. A. W. F. Edwards also developed elegant display methods for diagrams composed of higher numbers of sets that feature some interesting symmetries [2]. The Edwards Venn diagram was achieved by projecting the classical circular Venn diagram onto a sphere. In this format, three sets are represented by taking three hemispheres at right angles to each other, while a fourth set can be represented by taking a curve similar to the seam on a tennis ball which undulates around the sphere equator. The resulting sets can then be projected back to a flat plane, generating ‘cogwheel’ diagrams with increasing numbers of teeth.

Numerous online Venn diagram programs are currently available to create a simultaneous visual interpretation of large amounts of biological data with up to four independent datasets, examples of such programs include Pangloss Venn diagram generator (http://www.pangloss.com/seidel/Protocols/venn4.cgi) and Venny (http://bioinfogp.cnb.csic.es/tools/venny/index.html). These programs provide an invaluable resource, but are often designed primarily to generate images rather than assist facile output and attachment of additional identifiers to the data. Venn diagram programs can require laborious transfer of information from spreadsheets into text files before inputting. With the use of ever more complex datasets, this data handling can become a serious, time-limiting issue. In addition to input limitations, modern Venn diagram programs, such as the excellent program Venny, are capable of comparing only a maximum of four data sets with symmetrical ellipsoidal regions [3]. DrawVenn (http://theory.cs.uvic.ca/euler/DrawVenn/) and BioVenn [4] are also useful programs that instead allow the generation of area-proportional venn set analyses. Programs such as GeneVenn (http://www.bioinformatics.org/gvenn/) and VennMaster (http://www.informatik.uni-ulm.de/ni/staff/HKestler/vennhyp/) have the additional feature of linking genes within each group to related information in the NCBI Entrez Nucleotide database or the Gene Ontology database, but these programs are still limited to the analysis of two or three gene lists [5], [6]. GeneSpring (http://www.strandgenomics.com/GeneSpring) and SilicoCyte (http://www.cytogenomic.com/silicocyte.htm) are software packages specifically designed to analyze large volumes of microarray data with several visualizations, including Venn diagrams. However both of these programs are also limited in both the number of data sets and the variety of studies for which they can be employed. The program we have developed, VENNTURE, provides a simple interface for Edwards Venn diagram generation. It is designed with the following novel attributes in mind, *i.e.* ease of data importation and exportation, compatibility with commonly-used windows-based programs and a sufficient flexibility to facilitate the analysis of two to six sets of any length of dataset.

An ideal practical application for the simultaneous Venn analysis of more than four discrete sets is the investigation of concentration-dependent effects in log dose-response curve relationships generated from drug/ligand-mediated transmembrane receptor activation. The ligand-mediated log-dose response curve (LDRC) has long been one of the fundamental experimental paradigms in pharmacology. Such simple experiments allow the quantitative and qualitative investigation of ligand-mediated cell signaling effects. For example, a simple LDRC usually consists of a control ligand response effect (*i.e.* no drug/ligand added) and normally five logarithmically-increasing ligand/drug doses, each generating increasing or decreasing cellular responses. Ideally this drug/ligand dose range should encompass both the half-maximal effect of the drug/ligand and also the maximal cellular response. In recent times however, receptor research especially in the field of G protein-coupled receptors (GPCRs), has indicated potential limitations in LDRCs, *i.e.* they only identify the dose-dependent variation of signal generation for one output index, *e.g.* intracellular calcium mobilization. This single function output is now considered a serious limitation for GPCR analysis, as they are now known to activate many more signaling pathways simultaneously than previously thought [7]–[10]. Recent evidence has also suggested that distinct isomeric structures of the same GPCR, stabilized by differential intracellular protein interactions, can mediate qualitatively distinct signaling responses to different ligands [8], [9], [11]. It is therefore possible that for a given LDRC profile, distinct receptor isomers could differentially contribute to the creation of the various dose-related responses [8], [12]. In addition to our current adjustments to hypotheses of GPCR function, our historical appreciation of the cellular signaling pathways activated by GPCRs as linear and discrete entities, has now largely been superseded by a broader posit that resultant molecular signaling functions are the products of highly-interconnected molecular (gene or protein) network interactions [7]–[9], [13]. The functional output therefore of cellular signaling cascades may be best appreciated by studying the interaction and convergence of multiple pathways and signaling paradigms. Indeed, investigation and profiling of GPCR signaling pathways is now often performed at both the genomic and proteomic levels. These two techniques often can generate large volumes of data, especially with the introduction of paradigms involving multiple ligand structures, multiple drug doses and stimulation time-points as well as the use of diverse cellular receptor contexts [11], [14], [15]. This final aspect described, *i.e.* cellular receptor context, is emerging as a critical factor in analyzing receptor signaling function. We have recently demonstrated a novel cellular receptor context model, generated by molecular adaptive changes to a mild long-term oxidative stress (termed ‘chronic minimal peroxide-*CMP*’), which mimics multiple aspects of the neuronal aging process [14]. Using this model, we demonstrated that multiple receptor-mediated processes are profoundly altered at both the cell signaling and the genomic response level. The ability to appreciate numerous functional aspects and nuances in GPCR signaling is likely to assist with the development of more cellular context-specific and signal-selective GPCR-based therapeutics [7], [8]. GPCR-based therapeutic design has been one of the most important advances in clinical pharmacology, as nearly 60% of all current drugs are GPCR-targeted [16]. Therefore the ability to analyze multiple (up to 6) complex datasets from LDRC experiments, in parallel, may greatly assist our future understanding of the complexity of biological receptor signaling systems across multiple cellular contexts. In this study, we have employed our newly developed software program, to not only identify the multiple subtleties of basic dose-dependent GPCR signaling, but also to illuminate the importance of considering cellular context with respect to functional GPCR signaling outputs.

## Methods

### Cell Culture

Human neuroblastoma SH-SY5Y cells were used to generate the biological data employed in this study. Passage 5–10 SH-SY5Y cells were obtained from the American Type Culture Collection (ATCC: http://www.atcc.org/) and cultured according to ATCC protocols. SH-SY5Y cells were maintained in medium that was a 1∶1 mixture of advanced minimal essential medium (MEM) and F-12+ GlutaMAX-1 supplemented with 10% fetal bovine serum and 1% penicillin/streptomycin (Gibco-BRL), as described previously [14].

### Cell Stimulation Protocols

SH-SY5Y cells were allowed to grow in monolayers and were serum-deprived for 16 hours prior to experimental protocols. Cell monolayers of SH-SY5Y cells, at approximately 75% confluence, were stimulated with various doses, 10 nM, 100 nM, 1 µM, 10 µM or 100 µM, of the muscarinic G protein-coupled receptor agonist, acetyl-β-methylcholine chloride (MeCh: Sigma Aldrich, St Louis MO) for fifteen minutes before removal of the stimulating ligand by aspiration. Cell monolayers were then washed twice in ice-cold phosphate-buffered saline (PBS). Cells were then manually removed from the plates by scraping followed by centrifugal pelleting (1000×g, 5 minutes at 4°C). Recovered cell pellets were suspended in freshly prepared 8 M urea (Invitrogen, Carlsbad CA) with 50 mM NH_4_HCO_3_ (Fluka) and were completely disrupted using a Sonic Dismembrator (Model 100, Thermo Fisher Scientific). Protein lysate concentrations were subsequently determined by a BCA (bisinchoninic acid) protein assay kit (Thermo Fisher Scientific). Extracted proteins were immediately processed for subsequent phosphoprotein isolation and mass spectrometric analysis.

### Cellular Context Modification

To modify the cellular context of the SH-SY5Y cells, experimental ligand stimulation with MeCh was performed in both normal (control state) serum-deprived SH-SY5Y cells and also SH-SY5Y cells exposed to seven days of incubation with hydrogen peroxide (10 nM) [14]. This form of hydrogen peroxide exposure, simulates in several aspects, long-term minimal oxidative cell stress. This peroxide exposure paradigm that we have previously described as ‘chronic minimal peroxide’ (CMP), can induce multidimensional genomic and proteomic alterations that can generate a partial mimetic of aged neuronal tissues. In addition, this CMP treatment has also been shown to significantly alter ligand-mediated drug effects [14]. Whole-cell lysates were then produced as described in the previous section and phosphoproteins were purified using immobilized metal affinity chromatography and identified using a ThermoFinnigan LXQ tandem mass spectrometer.

### Western Blotting

To identify specific protein expression alterations in response to the CMP treatment, as well as to verify MeCh-induced protein phosphorylation targets, SH-SY5Y whole-cell extracts were first quantified using BCA reagent (ThermoScientific, Rockford IL) before resolution with SDS-PAGE and electrotransfer to PVDF membranes (Perkin Elmer, Waltham MA). Membranes were blocked for western blots, as described previously [13] and primary antibody immune-reactive complexes were identified using alkaline phosphatase-conjugated secondary antisera (Sigma Aldrich) with enzyme-linked chemifluorescence (GE Healthcare, Piscataway NJ) and quantified with a Typhoon 9410 variable-mode phosphorimager. Anti-lamin-A (LMNA), anti-calmodulin (CALM1), anti-calreticulin (CALR) sera were obtained from Santa Cruz Biotechnology (Santa Cruz, CA) and anti-G protein-coupled receptor kinase interacting ArfGAP 2 (GIT2) sera was obtained from NeuroMab (San Jose, CA). Anti-active, and anti-non-active extracellular signal-regulated kinase 1/2 (ERK1/2), anti-G protein-coupled receptor kinase interacting ArfGAP 1 (GIT1) sera were obtained from Cell Signaling Technology (Danvers, MA). ERK1/2 activation log dose-response relationships and any subsequent statistical analysis (Student’s t-test) were assessed using GraphPad Prism version 5.02.

### Immunoprecipitation Protocols

SH-SY5Y cells, either non-stimulated or MeCh-stimulated, were prepared for immunopurification of phosphorylation target proteins as follows. MeCh-containing media (post fifteen minute stimulation) was aspirated from the stimulated cell monolayers, which were then washed twice in ice-cold phosphate-buffered saline before lysis in an NP-40-based lysis buffer as described previously [17]. Cell lysates were prepared by agitation for 20 minutes at 4°C before centrifugal clarification (14000 rpm/10 minutes/4°C). Normalized supernatant protein lysates were pre-cleared using protein A/G pre-conjugated agarose beads (EMD Chemicals, Gibbstown NJ). Specific anti-protein sera (10 µg total), plus 30 µL of protein A/G pre-conjugated agarose beads, were then added to the pre-cleared supernatants with agitation at 4°C for four hours. Immunoprecipitates were collected via centrifugation, washed in the NP-40-based buffer and proteins were eluted in 30 µL Laemmli (8% SDS) buffer. Samples were then processed for western blotting. Specific antisera employed for immunoprecipitation studies were obtained from the following sources: anti- G protein-coupled receptor kinase interacting ArfGAP 1 (GIT1) and anti-p21 protein (Cdc42/Rac)-activated kinase 1 (PAK1) was obtained from Cell Signaling Technology (Danvers, MA); anti-G protein regulated inducer of neurite outgrowth 1 (GPRIN1) and anti-reticulon 4 (RTN4) were obtained from Abcam (Cambridge, MA); anti-Grb2-associated binding protein 2 (GAB2) and anti-microtubule-associated protein 2 (MAP2) were obtained from Santa Cruz Biotechnology (Santa Cruz, CA); anti-phosphoserine/phosphothreonine antisera were obtained from Millipore (Billerica, MA).

### Mass Spectrometry-targeted Primary Protein Extraction

Proteins were extracted from SH-SY5Y cell pellets for mass spectrometry analysis as follows. In brief, samples were initially reduced in 12.5 mM tris(2-carboxyethyl) phosphine (Sigma) at room temperature (RT) for 1 h, then alkylated with 25 mM iodoacetamide (Sigma) at RT for 30 min with protection from ambient light. The reaction was then quenched with incubation in 15 mM dithiothreitol (Sigma) at RT for 15 min. Resultant protein extracts were diluted in 50 mM NH_4_HCO_3_ to a final concentration of 1 M urea and were digested using sequencing-grade trypsin (Promega) at a 1∶75 (trypsin/protein, w/w, 37°C) ratio. After a sixteen hour digestion, formic acid (Fluka) was added to a final concentration of 0.5% (v/v). Peptide samples were then desalted using Sep-Pak Vac C18 cartridges (3cc/200 mg, Waters). Sep-Pak cartridges were then washed with 3 mL of acetonitrile (ACN, Thermo Fisher Scientific) and equilibrated with 3 mL of 0.1% formic acid. Cartridges were loaded with peptides (1.5 mg per cartridge), then washed with 6 mL of 0.1% formic acid. Peptides were subsequently eluted with 1 mL of 80% ACN/0.1% formic acid and dried to below 50 µL using a Savant SpeedVac (Thermo Fisher Scientific) before further processing and phosphoprotein purification.

### Phosphoprotein Extraction and Purification

All mass spectrometric columns employed were fabricated using 360 µm outer-diameter fused-silica capillaries (Polymicro Technologies). Phosphoproteins were extracted from general protein extracts using a modified immobilized metal affinity chromatography (IMAC) process using titanium dioxide (TiO_2_) particles. To create TiO_2_ IMAC columns, 150 µm inner-diameter capillary was cut and one end was placed in Kasil solution (Next Advance, Averill NY) to allow the solution to enter the capillary to a length of 3–4 cm by capillary action. Frit formation, to create a stable compartment to retain the TiO_2_ resin, was completed using heating of the packed capillary (16 h, 70°C). The excess frit was removed (1 cm remaining), then the capillary was packed with TiO_2_ particles (Titansphere TiO_2_, 5 µm, GL Sciences) and suspended in 50% methanol (J.T. Baker). Capillary column packing, washing, sample loading and elution, were carried out manually using a controllable nitrogen pressure loader (Next Advance-model PC 77). The amount of packed TiO_2_ particles was measured by weighing the column before and after packing, using a microbalance (Sartorius Instruments, Goettingen Germany) in dry laboratory conditions. Approximately 6 cm packing was equal to 2 mg of particles of TiO_2_ particles. An analytical C18 column, with integrated electrospray emitter tip, was prepared on a laser puller (P2000, Sutter Instruments, Novato CA) with a 75 µm internal-diameter fused-silica capillary, using a previously developed protocol [18]. C18 resins (Magic C18, 5 µm, 300 Å, Michrom Bioresources, Auburn CA) were packed to 8.5 cm of the 10.5-cm frit-less capillary column. The resulting C18 capillary column was used for standard analysis and as the electrospray interface for liquid chromatography-tandem mass spectrometry (LC–MS/MS) [19]. To enrich primary extracted peptide samples for phosphorylated species, the TiO_2_ capillary column was first treated with 1% ammonia (Fluka, St. Louis MO) and then with a modified wash buffer containing, 80% ACN, 2% trifluoroacetic acid and 1 M glycolic acid (‘ATG’ buffer), for 10 min each at a 1.5-µL/min flow rate using the nitrogen pressure loader. Peptide samples were mixed 1∶1 with the specific ATG buffer and loaded onto the column at a flow rate of 0.8 µL/min. The loaded amount of total peptide was 150 µg. The TiO_2_ column was then washed with ATG buffer for 10 min at 1.5 µL/min and then bound peptides were eluted with 1% ammonia (pH 11.3) for 5 min at 1.5 µL/min and then immediately acidified with 5 µL of 2% trifluoroacetic acid (Fluka). All of the samples were prepared as three replicates from tryptic digestions and were stored at –80°C until used.

### Mass Spectrometric Analysis

LC–MS/MS was performed using an LXQ linear ion-trap mass spectrometer coupled with a Surveyor LC system (ThermoFisher Scientific, West Palm Beach FL). Peptide samples were pressure-loaded onto pre-equilibrated analytical columns with a flow rate of 200–400 nL/min. Capillary tip columns were connected to the LXQ ion source in the MS system, and reversed-phase chromatographic separations were carried out using the following gradient settings: 100% A (0.1% formic acid in water) for 10 min, 0–50% B (0.1% formic acid in 100% ACN) for 100 min, 50–70% B for 10 min, and 70% B for 5 min. Pump flow rates were controlled to deliver 200 nL/min to the analytical column. This flow rate was consistently and reliably achieved by splitting the pump flow at a 1∶1000 ratio. LXQ MS settings were as follows: spray voltage of 1.6 kV, 1 microscan for MS scans at a maximum inject time of 10 ms with a mass range of *m/z* 400–1400, and 3 microscans for MS/MS at a maximum inject time of 100 ms with automatic mass range. The LXQ was operated in a data-dependent mode, that is, one MS scan for precursor ions followed by four data-dependent MS/MS scans for precursor ions above a threshold ion count of 500 with a normalized collision energy value of 35%.

MS/MS data processing and database searches were performed on BioWorks Browser 3.3.1 SP1 (Thermo Fisher Scientific). DTA files were generated from LC–MS/MS raw files with the following options applied: precursor ion tolerance of 1.5 amu, group scan 1, minimum group count 1, minimum ion count 20, and filtering through charge state analysis. The generated DTA files were searched against a composite target–decoy database [20]. This database was composed of human complete proteome (UniProt release-15.8, with 59,155 protein entries) and its reversed sequence version with the following criteria: enzyme, trypsin (KR/P); full enzymatic cleavage; missed cleavage sites, 3; peptide tolerance, 2.0 amu; fragment ion tolerance, 1.0 amu; variable modifications, carbamidomethylation (+57 Da), methionine oxidation (+16 Da), and STY phosphorylation (+80 Da); modifications per peptide, 4. The search result was filtered with Xcorr versus charge state (Xcorr of 1.5, 2.0, 2.5, and 3.0 for +1, +2, +3, and >+3 charges, respectively) and delta CN 0.08. To ensure that the false discovery rate (FDR) of peptide–spectral matches (PSMs) remained below 0.5%, the search results were further filtered with various Sf (Forial Score) values. An Sf value of 0.60 was used for an FDR of 0.49% for the overall results. Fragmentation spectra for peptides with only a single observation and for multiply phosphorylated peptides were verified through manual inspection according to previously published protocols [21].

### Ligand-mediated Phosphorylation Data Analysis

The baseline presence of phosphoproteins in the serum-deprived non-stimulated SH-SY5Y cells, as well as the induction of protein phosphorylation by the fifteen minute stimulation with 10 nM, 100 nM, 1 µM, 10 µM and 100 µM MeCh doses was assessed in the presence or absence of our applied CMP protocol. In addition to the primary protein analysis, we performed both Gene Ontology Analysis using WebGestalt (p≤0.05 for specific GO term groups) (http://bioinfo.vanderbilt.edu/webgestalt/) as well as canonical signaling pathway analysis using Ingenuity Pathways Analysis version 8.5 (http://www.ingenuity.com/). Gene Ontology (GO) term groups are curated functional annotations for protein activity (http://www.geneontology.org/). Proteins can be associated with multiple GO term groups after the generation of empirical data concerning the specific activities of that protein. Multifunctional proteins such as actin can be associated with many different GO term annotations, while more specific proteins are associated with a more narrow range of GO terms. Using our novel Venn diagram program, VENNTURE, we were then able to separate the multiple phosphoproteins, significantly-populated GO terms and significantly-populated canonical signaling pathways associated with the differing doses (in control or CMP environments) of MeCh stimulation.

### VENNTURE Software Development and Application

VENNTURE was programmed using the Microsoft Foundation Class (MFC) library, written in C++. The resulting MFC application can be used on any personal computer using any XP or higher Windows™ based system. VENNTURE version 1.1.0.2 can be accessed freely at the National Institute on Aging (http://www.nia.nih.gov). To upload biological data in VENNTURE, the data sets must be organized in an Excel™ spreadsheet, where each column corresponds to a specific data set. The first column in any given spreadsheet represents set 1 and the successive columns the additional sets (up to 62 sets for 6-way Edwards diagrams). To upload the data into VENNTURE simply requires activation of the ‘Load’ window option and browsing for the appropriate Excel™ spreadsheet file (*.xls or *.xlsx). VENNTURE then automatically searches for multiple sets within the first sheet of the Excel™ file. One Excel™ file should only contain data columns in the first sheet in the file. After the appropriate file is loaded, VENNTURE converts the datasets into strings, and uploads the information into internal arrays. Once the data has been uploaded, the data strings are compared to determine overlap and create the appropriate Edwards Venn diagram display categories. VENNTURE then draws the appropriate Venn diagram for the number of data sets contained in the uploaded Excel™ file. Depending on the number of datasets uploaded, VENNTURE will draw the appropriate Edwards Venn diagram variant. The Venn diagram will then be populated with the categorized data. In addition to creating the visual Edwards Venn image, VENNTURE simultaneously produces an Excel output *.xls file. In this Excel™ file, each overlapping category is separated into a column directly related to the numerical set order from the master Venn diagram output. The ‘Display Mode’ window for the Edwards Venn visual display allows the user to replace the numerical categories with specific textual representation of the input data points. The VENNTURE program also contains a mouse ‘roll-over’ demonstration function for demonstration of the contents of each of the specific sets in any format of Venn output. The Edwards Venn image can then be simply exported graphically in two different ways. One output option is to use the ‘Save As’ function to export the Edwards Venn image as an enhanced metafile format graphics (*.emf) file. The second option is to use the ‘Export’ function which instantly generates a Powerpoint™ file of the Edwards Venn. From this Powerpoint™ file a high resolution image (JPG, TIFF, PNG *etc.* ) can be generated using the Powerpoint™ ‘Save As’ function.

**Figure 1:**
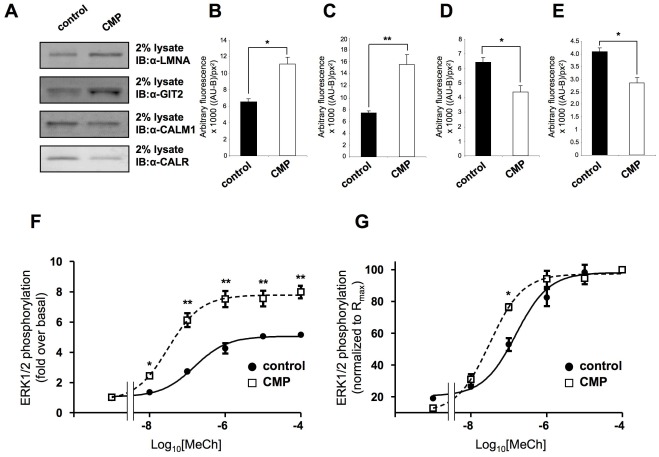
Chronic minimal peroxide-mediated alteration of protein expression and ligand responses. (A) Representative western immunoblot (IB) analysis of control (PBS-vehicle treated) or CMP treatment (7 days, 10 nM hydrogen peroxide treatment) effects upon cellular expression of lamin A (LMNA), G protein-coupled receptor kinase interacting ArfGAP 2 (GIT2), calreticulin (CALR) and calmodulin (CALM1). Quantification of CMP-mediated expression changes of LMNA (B), GIT2 (C), CALM1 (D) and CALR (E). Quantification was achieved as follows: western blot relative absorbance units (x1000) minus background absorbance subtraction per square pixel: ((AU-B)/px^2^). Quantification was performed using ImageQuant 5.2 and statistical analysis was performed on three independent experiments using GraphPad Prism version 5.02 with a Student’s t-test (p<0.05, *; p<0.01, **; p<0.001, ***). Data is represented as mean ± standard error of mean (SEM). (F) Log dose-response curves for acetyl-β-methycholine (MeCh)-mediated ERK1/2 activation in control (black circles) or CMP-treated (white square) cells. (G) Response-normalized MeCh ERK1/2 log dose-response curves. The maximal response for MeCh in each cellular context (control versus CMP) was considered to be 100% in each circumstance. Each experimental point on the curves (fitted using sigmoidal dose-response functions in GraphPad Prism) represents the mean ± SEM of three independent experiments.

## Results

### Cellular Context Modification and Ligand Responses

The proteomic effects, in human SH-SY5Y neural cells, of our previously-described chronic minimal peroxide (CMP) protocol was assessed, using a panel of divergently CMP-regulated proteins we had previously identified as specific markers of this modified cellular state [14] (Figure 1). Application of the CMP protocol to SH-SY5Y cells resulted in a significant upregulation of both lamin (LAMN) and GIT2 (G protein-coupled receptor kinase interacting ArfGAP 2: Figure 1A, B-C) and simultaneous, significant downregulation of calmodulin (CALM) and calreticulin (CALR) (Figure 1A, D-E). To assess the ability of the stable acetylcholine analog (acetyl-β-methylcholine: MeCh) to stimulate signaling activity in the SH-SY5Y cells, MeCh-mediated extracellular signal-regulated kinase (ERK1/2) activation was assessed. In both control-state and CMP-treated SH-SY5Y cells MeCh application resulted in a dose-dependent increase in the activity status of ERK1/2, as we have previously reported [14]. The basal level of non-phosphorylated as well as phosphorylated ERK1/2 was not significantly altered by the CMP protocol. From log dose-response analysis, it was apparent that the alteration in cellular context induced by CMP, caused an increase in the maximal response (R_max_) to MeCh (Figure 1F). ERK1/2 activation responses to each of the doses of MeCh tested (in both control- and CMP-state cells) were effectively blocked by the pre-exposure of the SH-SY5Y cells to the selective muscarinic G protein-coupled receptor antagonist pirenzipine (10 µM, 30 minutes incubation prior to MeCh exposure). In addition, no significant alteration of plasma membrane muscarinic receptor expression was noted between control and CMP conditions (Figure S1: [14]).

**Figure 2:**
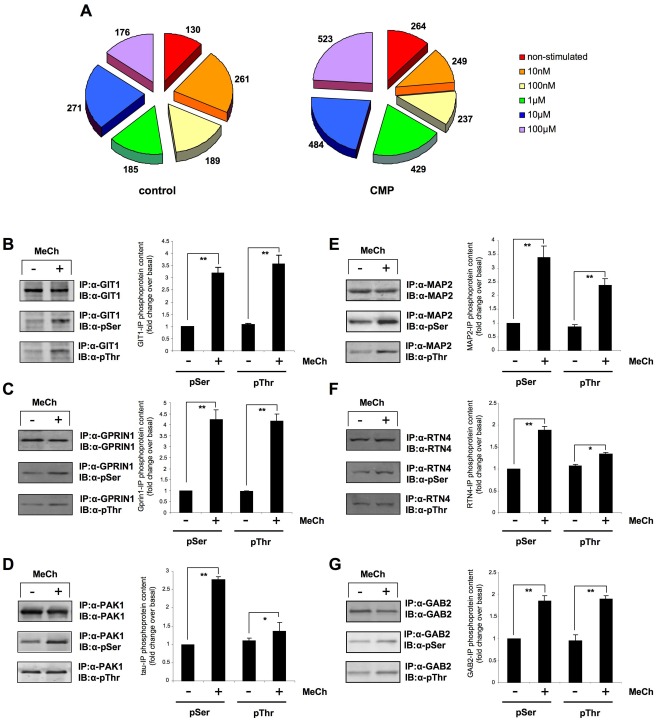
MeCh-induced protein phosphorylation in diverse cellular contexts. (A) Proportional diagrams illustrating the number of specifically enriched and identified phosphoproteins, basally occurring, and induced by MeCh stimulation (10 nM to 100 µM) of control or CMP-treated cells. (B) Selective protein immunoprecipitation (IP) and determination of MeCh-induced G protein-coupled receptor kinase interacting ArfGAP 1 (GIT1) serine and threonine phosphorylation, with antisera immunoblot (IB), in MeCh-stimulated control-state SH-SY5Y cells. The associated histogram (representing mean ± SEM data from three independent immunoprecipitations) indicates the extent of immunoprecipitated protein serine and threonine phosphorylation. Panels (C) and (D) indicate similar data from control-state cells for the MeCh-induced phosphorylation of G protein regulated inducer of neurite outgrowth 1 (GPRIN1) and p21 protein (Cdc42/Rac)-activated kinase 1 (PAK1). (E) Selective protein immunoprecipitation and determination of MeCh-induced microtubule-associated protein 2 (MAP2) serine and threonine phosphorylation, with antisera immunoblot, in MeCh-stimulated CMP-state SH-SY5Y cells. Panels (F) and (G) indicate similar data from CMP-state cells for the MeCh-induced phosphorylation of reticulon-4 (RTN4) and GRB2-associated binding protein 2 (GAB2). Statistical analysis was performed on three independent experiments using GraphPad Prism version 5.02 with a Student’s t-test (p<0.05, *; p<0.01, **; p<0.001, ***).

### Phosphoprotein Extraction and Enrichment

Using a titanium dioxide-mediated phosphoprotein extraction process [22], we were able to identify in SH-SY5Y cells the presence of multiple phosphoproteins in the non-stimulated control and peroxide-treated (CMP) state, as well as after the application of 10 nM, 100 nM, 1 µM, 10 µM and 100 µM acetyl-β-methylcholine (MeCh) in both cellular conditions (Protein identifications listed in Supplementary Tables as follows: control SH-SY5Y cells, non-stimulated-Table S1, 10 nM, 100 nM, 1 µM, 10 µM, 100µM MeCh-Tables S2, S3, S4, S5, S6 respectively; CMP SH-SY5Y cells, non-stimulated-Table S7, 10 nM, 100 nM, 1 µM, 10 µM, 100 µM MeCh-Tables S8, S9, S10, S11, 12 respectively). Therefore, in our paradigm of ligand dose-responses in diverse cellular contexts, we were able to generate six different phosphoprotein datasets for each cellular context, *i.e.* control or CMP-treated. The total number of phosphoproteins specifically enriched and identified (≥2 peptides required for identification of each specific protein) for each of the MeCh doses applied, as well as the proteins identified in the non-stimulated conditions for each cell context (control or CMP) are depicted in Figure 2A. We validated the presence of several protein serine or threonine phosphorylations, stimulated by MeCh at multiple doses (10 nM, 100 nM, 1 µM), in both cell contexts (control, Figure 2B–D; CMP, Figure 2E–G) using selective immunoprecipitation of the target protein. The isolated target protein was then immunoblotted with specific anti-phosphoserine and anti-phosphothreonine antisera. We did not assess the anti-phosphotyrosine content of the selected proteins due to the considerably lower extent of this form of protein phosphorylation typically observed with TiO_2_-mediated enrichment techniques [22]. Using immunoblotting, we verified our proteomic identification of the phosphorylation of G protein-coupled receptor kinase interacting ArfGAP 1 (GIT1: 10 nM MeCh), G protein-regulated inducer of neurite outgrowth 1 (GPRIN1: 100 nM MeCh) and p21 protein (Cdc42/Rac)-activated kinase 1 (PAK1: 1 µM MeCh) in control SH-SY5Y cells. We also verified the MeCh-induced increase in phosphorylation, identified with our TiO_2_-enrichment, of microtubule-associated protein 2 (MAP2: 10 nM MeCh), reticulon-4 (RTN4: 100 nM MeCh) and Grb2-associated binding protein 2 (GAB2: 1 µM MeCh) in the CMP-treated SH-SY5Y cells.

**Figure 3:**
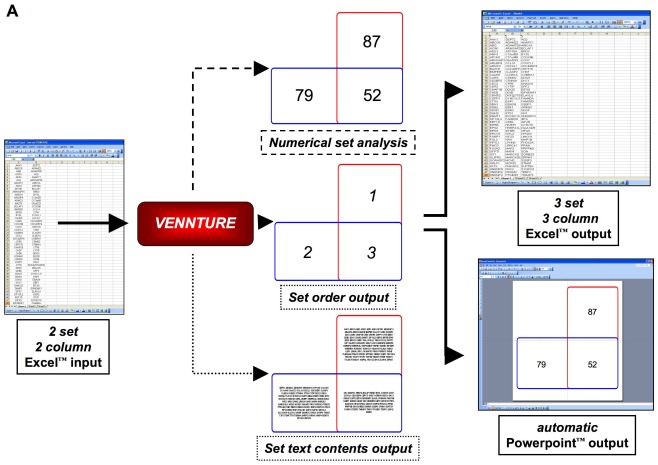
VENNTURE data input and output scenarios. Simplistic data (two set analysis) input into VENNTURE is achieved using an Excel™ input file. The data representation type can be selected using a drop-down menu for numerical set analysis, set order demonstration for eventual output and also textual set content. The resultant set data output can be directly generated for PowerPoint™ (Venn diagram) and Excel™ (dataset descriptions).

**Figure 4:**
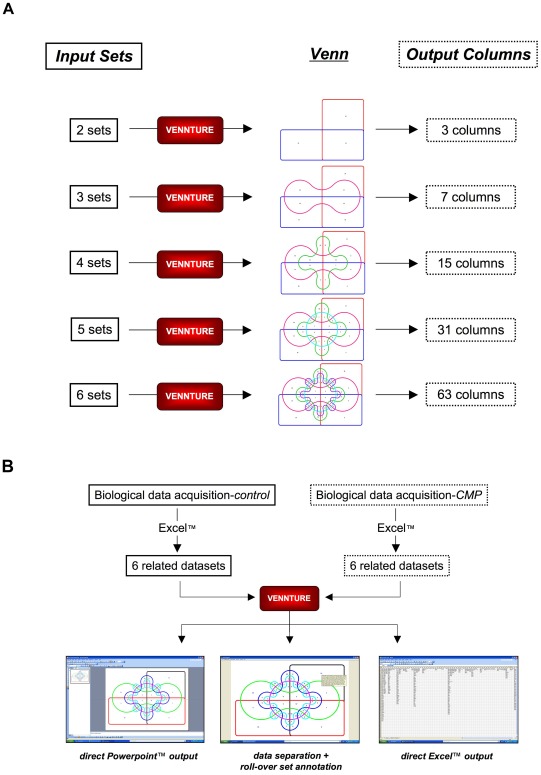
Multiple set VENNTURE data input. (A) In addition to simplistic data (two set analysis) input into VENNTURE, the program is flexible in that it can accept up to 6 input sets. With increasing set input, from Excel™ files, is a concomitant elevation in diagram complexity along with the eventual dataset output in column format in an Excel™ spreadsheet. With increasing numbers of input sets VENNTURE automatically alters its Venn output and organizes the eventual column display in Excel™. (B) Basic flow-chart for use of VENNTURE in the experimental investigation of the log dose-response of MeCh-induced phosphoproteomic changes in multiple cellular contexts. VENNTURE data output can be used for diagram generation (PowerPoint™ output), direct inspection Venn intersection contents (direct roll-over reveal option) and output for mathematical calculation (Excel™).

### VENNTURE Biological Data Analysis and Implementation

Our novel software program, VENNTURE, is constructed to allow simple data (*e.g.* two to six datasets of any length) uploading from commonly used software (Excel™), facilitate various forms of dataset annotation display and allow simple exportation of results to commonly used data and visual analytical tools (Excel™, Powerpoint™) (Figure 3). Employing a classical Edwards ‘cogwheel’ approach, VENNTURE is able to accommodate between 2 and 6 individual datasets that generate an ever-increasing series of Excel™ spreadsheet data output, due to a rapid increase in Venn diagram intersection complexity (Figure 4A). For our primary application in this study, we have generated two groups (control or CMP) of six separate datasets of proteins displaying a ligand-mediated phosphorylated status which we then uploaded into VENNTURE. We then employed VENNTURE for spreadsheet analysis, visual analytical output and also for direct visual appreciation of protein identification in each intersection using VENNTURE’s ‘roll-over’ annotation option (Figure 4B).

**Figure 5:**
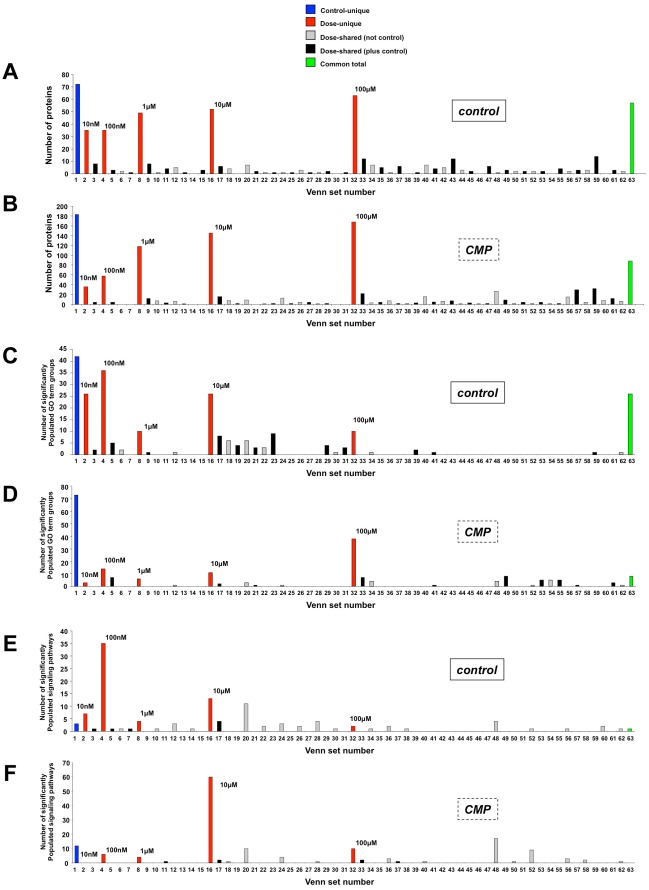
VENNTURE analysis of log dose-response ligand data in diverse cellular contexts. VENNTURE Venn diagram set distribution analysis (1–63 set intersections) of extracted phosphoproteins in the stimulated or non-stimulated SH-SY5Y cells in the control state (‘Control’ - A) or peroxide-treated state (‘CMP’ - B). Specific column color coding is as follows: blue - phosphoproteins unique to non-stimulated state only, red – phosphoproteins unique to a specific MeCh stimulation dose only; grey – phosphoproteins common to multiple MeCh doses but not present in the non-stimulated set; black – phosphoproteins common to multiple MeCh doses also present in the non-stimulated set; green – phosphoproteins common to all doses and the non-stimulated set. GO term enrichment analysis was performed using the initial phosphoprotein sets from cells in the control or CMP-treated states. The significantly enriched (p≤0.05) GO term groups for each non-stimulated or MeCh-stimulated set were then separated using VENNTURE in a similar manner to the actual phosphoprotein identifications. VENNTURE Venn diagram set distribution analysis of GO term groups significantly populated by the extracted phosphoproteins in the control state (C) or CMP-state (D) cells is depicted. Color-coding of the histogram is as described previously. Canonical signaling pathway enrichment analysis was also performed using the initial phosphoprotein sets from cells in the control or CMP-treated states. The significantly enriched (p≤0.05) canonical signaling pathways for each non-stimulated or MeCh-stimulated set were then separated using VENNTURE in a similar manner to the actual phosphoprotein identifications. VENNTURE Venn diagram set distribution analysis of the canonical signaling pathways significantly populated by the extracted phosphoproteins in the control state (E) or CMP-state (F) cells is depicted.

### VENNTURE-based Dose-specific Data Analysis

VENNTURE was used to rapidly separate and identify the multiple subsections of the TiO_2_-enriched phosphoproteomic data. The two input six-set phosphoprotein lists that we employed for this assessment (using official human protein symbols) are represented in Table S13 (control) and Table S14 (CMP). Using VENNTURE we were able to differentiate the highly-complex, dose-dependent phosphoproteomic effects of MeCh treatment, in both the control- and peroxide-treated (CMP) state cells (Figure 5). In both cases when using VENNTURE to separate out the specific subsets of phosphoproteins, it became evident that different MeCh ligand doses, in both cellular contexts employed (control- or CMP-state), produced complex and largely ‘dose-unique’ phosphoproteomic lists (Figure 5, control-state 5A, CMP-state 5B). Hence, many of the MeCh-stimulated phosphoproteins were unique to a specific log dose of the ligand (‘dose-unique’ protein sets are listed in Table S15 (control) and Table S16 (CMP)). Such ‘dose-unique’ activity may suggest that these large differences in applied MeCh dose interact preferentially with different stable ‘sub-states’ of the same receptor [8], [23]–[26]. However it is also possible that these specific signaling profiles may also be influenced by the differential availability of downstream signaling molecules, the progressive activation of distinct stimulatory and autoregulatory signaling events as well as micro temporal or kinetic variances in ligand-receptor interactions induced by the different ligand doses. Even with these potential complexities of receptor signaling, using the unique capacities of VENNTURE, we have shown that different doses of a single ligand, stimulating a unitary target receptor, can activate largely distinct pools of phosphoproteins across a log-dose response series. While phosphorylation is potentially an important process in the alteration of individual protein function, this information in itself may not be specifically indicative of a given functional activity, as most physiological actions involve interactions between multiple associated proteins. We therefore applied Gene Ontology (GO) term analysis (WebGestalt: http://bioinfo.vanderbilt.edu/webgestalt/ [27], [28]) and canonical signaling pathway analysis (Ingenuity Pathway Analysis: http://www.ingenuity.com/) to generate a higher order of appreciation of the functional connections between our protein sets, allowing us to further investigate the ‘dose-unique’ behavior observed at the phosphoprotein level (Figure 5A, B). Data lists of significantly populated GO term groups (n≥2 proteins per group, probability *(p)*≤0.05) were created for each non-stimulated, or MeCh-stimulated dose state (Tables S17, S18, S19, S20, S21, S22 (control state) and Tables S23, S24, S25, S26, S27, S28 (CMP state)). For simple VENNTURE input, these dose-dependent tables were then summarized (Table S29 (control) and Table S30 (CMP)), and then subjected to six-way VENNTURE separation (Figure 5C-control state, 5D-CMP state). In an analogous manner to the phosphoprotein VENNTURE separations, we noticed a strong ‘dose-specific’ allocation of the different GO-term groups for MeCh treatment of control and peroxide-treated cells. This may suggest that with the relatively specific dose-dependent protein phosphorylation, there is also a dose-dependent variation in the activation of various functional groups of proteins. The ‘dose-unique’ groups of significantly enriched GO terms, for each cellular context, are listed in Table S31 (control state) and Table S32 (CMP-state). Significantly populated canonical signaling pathway matrices (n≥2 proteins per signaling pathway, *p*≤0.05) were next created for each non-stimulated, or MeCh-stimulated dose state in both cellular contexts (Table S33 (control state) and Table S34 (CMP-state)). The signaling pathway analysis was then subjected to six-way VENNTURE separation. In a manner reminiscent to the phosphoprotein and GO-term VENNTURE separations, we noticed a strong dose-unique allocation of the different canonical signaling pathways for MeCh treatment of control or peroxide-treated CMP cells (Figure 5E and 5F respectively). The ‘dose-unique’ groups of significantly enriched canonical signaling pathways for each cellular context are listed in Table S35 (control-state) and Table S36 (CMP-state).

**Figure 6:**
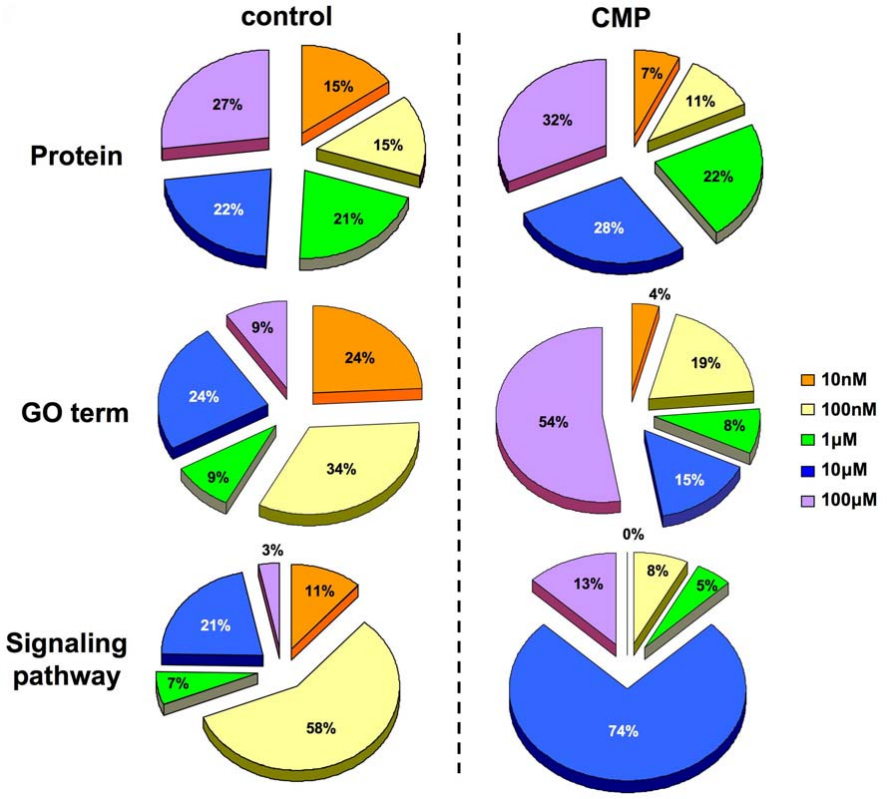
Dose-dependent distribution of MeCh-stimulated signaling activity in diverse cellular contexts. Proportional diagrams representing the relative distribution of phosphoproteins (Proteins)/GO term groups (GO term)/signaling pathways (Signaling Pathway) unique to an individual stimulating MeCh dose (color coded) in either cellular context (control or CMP).

**Figure 7:**
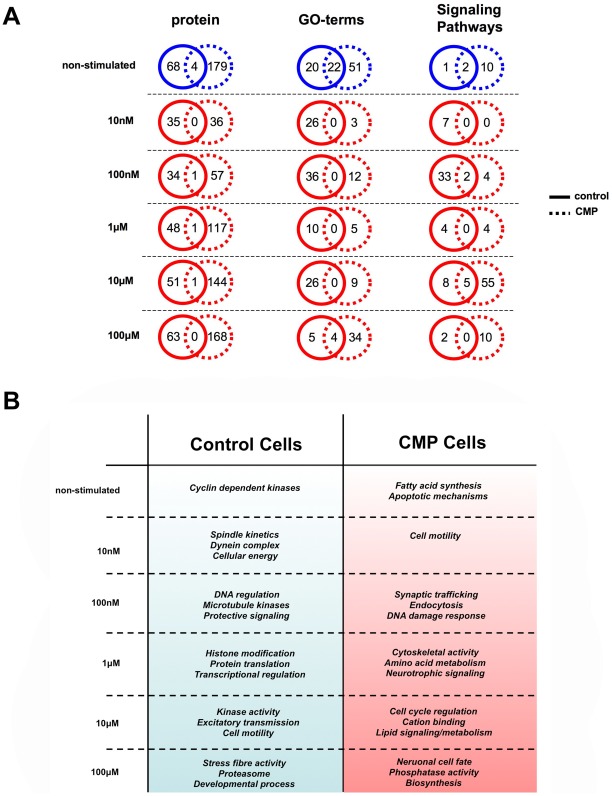
Cellular context modification of downstream receptor signaling activity in SH-SY5Y cells. (A) Comparison of phosphoprotein/GO term group/canonical signaling pathway activation in the non-stimulated (blue circle) and multiple MeCh-stimulated conditions (red circles) in either control-state (solid line) or CMP-state (dashed lines) SH-SY5Y cells. (B) Summary of potential non-stimulated and MeCh-induced signaling functionality (aggregate of highest-scoring GO-term group and canonical signaling pathway enrichments) in a dose-dependent manner compared between control-state or CMP-state cells.

### Dose-dependent Receptor Signaling Variation

Using VENNTURE, we were able to discern that many of the functional outputs of MeCh-mediated muscarinic receptor stimulation were strongly dose-dependent and also largely distinct at each different dose. We further investigated how this signaling diversity was specifically affected by dose between our two experimental cellular contexts, *i.e*. control state versus CMP state. We found that for phosphoproteins that were specific to MeCh-stimulated VENNTURE sets (Figure 5A–B, Venn sets 2–62) a similar percentage, in both cellular contexts, of these proteins were found in ‘dose-unique’ sets only (36% control-state, 38% CMP-sate). When analyzing the VENNTURE distribution of the GO terms and canonical signaling pathways, we found a largely similar distribution of factors to these ‘dose-unique’ Venn sets again for both cellular contexts, *i.e.* approximately 36–39% of the MeCh-stimulated GO term/signaling pathways (excluding phosphoproteins common to non-stimulated samples) were ‘dose-unique’ and in the CMP groups 35–38% were ‘dose-unique’ (data not shown). We then sub-divided these ‘dose-unique’ sectors into the respective dose-specific proportions of phosphoproteins/GO terms/signaling pathways (Figure 6). At the phosphoprotein level the percentage bias of each dose within the total number of ‘dose-unique’ phosphoproteins was generally similar in the control state cells (10 nM-15%, 100 nM-15%, 1 µM-21%, 10 µM-22%, 100 µM-27%). In contrast, for the CMP-state cells, a reduction in the percentage of phosphoproteins unique to the lower doses was observed (10 nM-7%, 100 nM-11%: Figure 6-Protein) compared to control-state cells. As proteins can possess multiple functions, and therefore could be grouped into several GO term groups or signaling pathways, it would be expected that a divergence in relative dose-specific proportions in these functional groups would occur. A stark contrast was observed with respect to the ‘dose-unique’ profile of GO term groups between the control and CMP-state cells. The relative proportions of ‘dose-unique’ GO term groups were roughly similar in control-state cells (Figure 6-GO term). However in CMP cells more than half (54%) of the total number of ‘dose-unique’ significantly-populated GO term groups were found at the 100 µM dose. Concomitantly, a large reduction of the low-MeCh dose-specific GO term groups was observed (10 nM-4% in CMP cells versus 24% in control cells: Figure 6-GO term). At the signaling pathway level, the dose-related functional output differences were even more diverse between control and CMP-state cells. In each cell context, one specific MeCh dose appeared to generate the most extensive signaling repertoire, *i.e.* ability to stimulate many forms of potential signaling cascades. Such activity is indicated by the largest numbers of significantly-populated signaling pathways populated by protein phosphorylation events induced by a specific ligand dose. In the control state cells this dose was 100 nM MeCh, while in the CMP-state cells this dose was 10 µM MeCh (Figure 6-Signaling pathway). No significant signaling pathways were populated by the lowest MeCh dose (10 nM) in the CMP-state cells, while 11% of total ‘dose-unique’ signaling pathways were stimulated specifically by 10 nM MeCh in the control-state cells. Such ligand dose- and cellular context-dependent alteration of receptor signaling profiles may be suggestive of altered populations of muscarinic receptors in stable isomeric conformations that are ‘hard-wired’ into specific signaling paradigms [8], [9], [13], [29], [30]. Reinforcing the considerable differences observed between ‘dose-unique’ proportions of phosphoprotein/GO term/signaling pathways in the control- or CMP-state cells, we found that in a dose-by-dose comparison there was minimal cross-over between proteins/GO terms/signaling pathways between these two cell contexts (Figure 7A). Combining and summarizing the functional annotation information (both GO term and canonical signaling pathway enrichment) for each dose of MeCh, it was apparent that distinct potential functional outcomes were generated by different MeCh doses within a single cellular context (Figure 7B). Moreover, in a dose-to-dose comparison, between cellular context models, profoundly distinct signaling outcomes were generated between these two cellular states (control versus CMP: Figure 7B). This may suggest that the functional outcomes of receptor stimulation may be considerably disrupted by the multiple proteomic/genomic alterations induced by CMP treatment which, in-part, mimics cell aging [14]. While the CMP treatment may affect dynamic responses to receptor stimulation, it clearly also exerts multiple and differential effects upon steady state (non-stimulated experiments) protein expression as well (Figure 7B: Tables S17, S23, S33, S34). Hence in non-stimulated control-state cells cyclin-dependent kinases signaling was evident, while *fatty acid synthesis* and *apoptotic mechanisms* were evident in non-stimulated CMP-state cells (Figure 7B). With respect to the dynamic signaling responses with 10 nM MeCh stimulation of control cells, an output related to *spindle kinetics*, *molecular motor function* and *energy regulatory pathways* was predicted, while similar MeCh-stimulation of CMP-treated SH-SY5Y cells suggested only effects upon *cell motility*. With 100 nM MeCh stimulation of control state cells, stimulation of *microtubule-associated protein kinases*, *DNA regulatory behavior* and *neuroprotective kinase* (phosphoinositide-3-kinase) signaling was predicted, while a distinct output phenotype was present in CMP cells, *i.e.* stimulation of *synaptic signaling* and *endocytic activity* was predicted. With 1 µM stimulation, again a strong divergence of signaling activity for MeCh was seen between control and CMP cellular states. In control cells, at a 1 µM dose, stimulation of *histone modifying activity* and *mRNA processing* was observed, while in CMP-cells signaling activity appeared to center more upon *neuronal filament/stress fiber modification*, *amine metabolism* and *neurotrophic signaling*. With 10 µM stimulation, MeCh-stimulation of control-state cells regulated *nucleotide-dependent kinase activity* as well as signaling involved in *pro-excitatory neurotransmission*. The 10 µM stimulation of CMP-state cells however was less phenotypically robust and was instead linked to *cation binding*, *genomic stabilization* and *lipid-mediated stress signaling*. At the highest dose of MeCh employed (100 µM), stimulation of *stress fiber*, *proteasomal* and *gene regulatory activity* was observed in control-state cells, while in CMP-state cells we noted a strong *neuronal differentiation*, *metabolic* and *biosynthetic* bias of signaling (Figure 7B: Tables S31, S32-GO terms; S35, S36-canonical signaling pathways).

## Discussion

Our novel program, VENNTURE, allows the facile simultaneous analysis of multiple large and complex pharmacological datasets. Using this program we have shown, with a high volume of complex input data, that VENNTURE is able to generate biological datasets that have aided the comprehension of higher orders of signaling behavior in ligand-receptor systems. Ligand-receptor interactions at GPCRs are perhaps one of the most important events in clinical and molecular pharmacology, as more than half of the current pharmacopeia is populated by GPCR-targeted drugs [8], [16]. For many years these molecular interactions were considered to be mechanistically relatively simple, *e.g.* the agonist-driven receptor structural change concept was described as the ‘two-state’ model of receptor activation. This model eventually was superseded by the ‘ternary complex model’ [31] that identified a third stable receptor conformation that was a ‘transition state’ between R and R* [32]. With experimental advances it has become clear that GPCR activity is even more complicated then ever imagined. *i.e.* GPCRs can demonstrate higher-order signaling complex assembly, downstream signaling promiscuity and signal-selective ligand behavior [33]–[41]. As both ligand interaction with GPCRs [23]–[25], [42]–[44] and the eventual downstream signaling induced by receptor activation [26], [45]–[49], are likely to be controlled by multiple cellular factors, such as stress-state or the presence of intracellular scaffolding proteins, it is imperative that our understanding of how these changes affect dose-dependent GPCR pharmacology be included in our drug discovery methodologies. Using VENNTURE we were able to distinguish and investigate highly nuanced dose- and context-specific ligand-receptor behavior. The ability to generate such an in-depth appreciation of the ligand-receptor signaling profile, in multiple cellular states, may be initially employed to greatly enhance the efficiency and accuracy of drug design. The ability to understand the changes in drug response profiles in different pharmacogenomic backgrounds, *e.g.* control and peroxide-treated states [14], may greatly assist in the generation of contextually-specific pharmacological agents. The functional experimental example demonstrated in this study outlines one potential use of VENNTURE, however, this novel program can be utilized for any research purpose that requires simultaneous comparison of multiple large data sets.

The specific application of our novel VENNTURE program to these extremely complex sets of signaling data allowed the facile elucidation of the subtle effects that environmental perturbations can exert upon dose-dependent ligand actions. Such findings may have profound implications for novel therapeutic design, as both the dose-specificity of signaling quality and its modulation by altered cellular context may be extremely important factors to consider for future GPCR drug discovery [8], [9], [14], [50].

The VENNTURE application can be used on any personal computer using any Windows™ XP or higher Windows™-based system. To make VENNTURE as accessible to as many potential users we have specifically tailored the program to employ the most commonly used MS-Office platforms, *e.g*. Excel™ and Powerpoint™. While the MS-Office suite is the most widely-used platform in the world, we appreciate that non-MS Office users also present an important user-base, therefore we intend to develop VENNTURE for use with non-MS-Office or OpenSource (*e.g*. OpenOffice) software platforms. Presently VENNTURE version 1.1.0.2 can be accessed freely at the National Institute on Aging (http://www.nia.nih.gov). There are some potential aspects to VENNTURE that can be expanded upon in the future, such as an elevation of dataset handling beyond only six sets. However, while VENNTURE is able to theoretically model an infinite number of data sets, the visual presentation of more than eight data sets ultimately becomes too visually complicated to extract actionable data. VENNTURE presents significant advancements in visual presentation, data analysis, and user-friendliness over currently available Venn diagram programs and it will be a useful tool for the analysis of complex datasets in pharmacology, genomics, and bioinformatics research fields.

## Supporting Information

Figure S1 **Effects of chronic minimal peroxide (CMP) exposure upon muscarinic receptor activity.** (A) Twenty micrograms of SH-SY5Y whole-cell lysate resolved using an SDS-polyacrylamide gel and then stained with coomassie for total protein detection (MW-molecular weight markers). Associated histogram and representative western blot indicate minimal alteration of m1-muscarinic acetylcholine receptor (m1AchR: antisera obtained from Sigma-Aldrich, St. Louis MO) expression (quantified as 1000× arbitrary intensity units (AU) minus background (B) intensity per square pixel (1000×(AU-B)/px^2^)) with application of CMP protocol (expression controlled using whole-cell coomassie stain). (B) Blockade of acetyl-β-methylcholine (MeCh)-mediated ERK1/2 activation in control-state cells with a 30 minute pre-exposure of cells to the muscarinic receptor antagonist pirenzipine (10 µM: gray shaded bars). (C) Blockade of MeCh-mediated ERK1/2 activation in CMP-state cells with a 30 minute pre-exposure of cells to the muscarinic receptor antagonist pirenzipine (10 µM: shaded bars).
(TIF)

Table S1 Phosphoproteins extracted from un-stimulated control-state human neuroblastoma SH-SY5Y cells. For each successfully identified protein official symbol, Uniprot accession code and number of peptides recovered are indicated.
(DOC)

Table S2 Phosphoproteins extracted from 10 nM MeCh-treated control-state human neuroblastoma SH-SY5Y cells. For each successfully identified protein official symbol, Uniprot accession code and number of peptides recovered are indicated.
(DOC)

Table S3 Phosphoproteins extracted from 100 nM MeCh-treated control-state human neuroblastoma SH-SY5Y cells. For each successfully identified protein official symbol, Uniprot accession code and number of peptides recovered are indicated.
(DOC)

Table S4 Phosphoproteins extracted from 1 µM MeCh-treated control-state human neuroblastoma SH-SY5Y cells. For each successfully identified protein official symbol, Uniprot accession code and number of peptides recovered are indicated.
(DOC)

Table S5 Phosphoproteins extracted from 10 µM MeCh-treated control-state human neuroblastoma SH-SY5Y cells. For each successfully identified protein official symbol, Uniprot accession code and number of peptides recovered are indicated.
(DOC)

Table S6 Phosphoproteins extracted from 100 µM MeCh-treated control-state human neuroblastoma SH-SY5Y cells. For each successfully identified protein official symbol, Uniprot accession code and number of peptides recovered are indicated.
(DOC)

Table S7 Phosphoproteins extracted from untreated chronic minimal peroxide (CMP)-state human neuroblastoma SH-SY5Y cells. For each successfully identified protein official symbol, Uniprot accession code and number of peptides recovered are indicated.
(DOC)

Table S8 Phosphoproteins extracted from 10 nM MeCh-stimulated chronic minimal peroxide (CMP)-state human neuroblastoma SH-SY5Y cells. For each successfully identified protein official symbol, Uniprot accession code and number of peptides recovered are indicated.
(DOC)

Table S9 Phosphoproteins extracted from 100 nM MeCh-stimulated chronic minimal peroxide (CMP)-state human neuroblastoma SH-SY5Y cells. For each successfully identified protein official symbol, Uniprot accession code and number of peptides recovered are indicated.
(DOC)

Table S10 Phosphoproteins extracted from 1 µM MeCh-stimulated chronic minimal peroxide (CMP)-state human neuroblastoma SH-SY5Y cells. For each successfully identified protein official symbol, Uniprot accession code and number of peptides recovered are indicated.
(DOC)

Table S11 Phosphoproteins extracted from 10 µM MeCh-stimulated chronic minimal peroxide (CMP)-state human neuroblastoma SH-SY5Y cells. For each successfully identified protein official symbol, Uniprot accession code and number of peptides recovered are indicated.
(DOC)

Table S12 Phosphoproteins extracted from 100 µM MeCh-stimulated chronic minimal peroxide (CMP)-state human neuroblastoma SH-SY5Y cells. For each successfully identified protein official symbol, Uniprot accession code and number of peptides recovered are indicated.
(DOC)

Table S13 Dose-dependent acetyl-β-methylcholine-stimulated phosphoproteins in control-state human neuroblastoma SH-SY5Y cells. Ligand stimulation with acetyl-β-methylcholine (MeCh) was for 15 minutes before cell lysate protein extraction and titanium dioxide-mediated purification.
(DOC)

Table S14 Dose-dependent acetyl-β-methylcholine-stimulated phosphoproteins in CMP-state human neuroblastoma SH-SY5Y cells. Ligand stimulation, performed after pre-treatment for seven days with 10 nM continuous hydrogen peroxide treatment (CMP protocol), with acetyl-β-methylcholine (MeCh) was for 15 minutes before cell lysate protein extraction and titanium dioxide-mediated purification.
(DOC)

Table S15 Dose-unique MeCh-stimulated protein phosphorylation in control-state SH-SY5Y cells. The proteins uniquely phosphorylated at the specified MeCh dose only in control-state SH-SY5Y cells are indicated by their protein symbol as well as Uniprot accession number.
(DOC)

Table S16 Dose-unique MeCh-stimulated protein phosphorylation in CMP-state SH-SY5Y cells. The proteins uniquely phosphorylated at the specified MeCh dose only in CMP-state SH-SY5Y cells are indicated by their protein symbol as well as Uniprot accession number.
(DOC)

Table S17 GO term groups populated by extracted phosphoproteins in non-stimulated control-state SH-SY5Y cells. GO term groups were considered enriched only if at least two proteins were present in each group and with a probability of ≤0.05. Hybrid GO term group scores were generated by multiplication of the GO term group enrichment score with the negative log_10_ of the probability result.
(DOC)

Table S18 GO term groups populated by extracted phosphoproteins in 10 nM MeCh-stimulated control-state SH-SY5Y cells. GO term groups were considered enriched only if at least two proteins were present in each group and with a probability of ≤0.05. Hybrid GO term group scores were generated by multiplication of the GO term group enrichment score with the negative log_10_ of the probability result.
(DOC)

Table S19 GO term groups populated by extracted phosphoproteins in 100 nM MeCh-stimulated control-state SH-SY5Y cells. GO term groups were considered enriched only if at least two proteins were present in each group and with a probability of ≤0.05. Hybrid GO term group scores were generated by multiplication of the GO term group enrichment score with the negative log_10_ of the probability result.
(DOC)

Table S20 GO term groups populated by extracted phosphoproteins in 1 µM MeCh-stimulated control-state SH-SY5Y cells. GO term groups were considered enriched only if at least two proteins were present in each group and with a probability of ≤0.05. Hybrid GO term group scores were generated by multiplication of the GO term group enrichment score with the negative log_10_ of the probability result.
(DOC)

Table S21 GO term groups populated by extracted phosphoproteins in 10 µM MeCh-stimulated control-state SH-SY5Y cells. GO term groups were considered enriched only if at least two proteins were present in each group and with a probability of ≤0.05. Hybrid GO term group scores were generated by multiplication of the GO term group enrichment score with the negative log_10_ of the probability result.
(DOC)

Table S22 GO term groups populated by extracted phosphoproteins in 100 µM MeCh-stimulated control-state SH-SY5Y cells. GO term groups were considered enriched only if at least two proteins were present in each group and with a probability of ≤0.05. Hybrid GO term group scores were generated by multiplication of the GO term group enrichment score with the negative log_10_ of the probability result.
(DOC)

Table S23 GO term groups populated by extracted phosphoproteins in non-stimulated CMP-state SH-SY5Y cells. GO term groups were considered enriched only if at least two proteins were present in each group and with a probability of ≤0.05. Hybrid GO term group scores were generated by multiplication of the GO term group enrichment score with the negative log_10_ of the probability result.
(DOC)

Table S24 GO term groups populated by extracted phosphoproteins in 10 nM MeCh-stimulated CMP-state SH-SY5Y cells. GO term groups were considered enriched only if at least two proteins were present in each group and with a probability of ≤0.05. Hybrid GO term group scores were generated by multiplication of the GO term group enrichment score with the negative log_10_ of the probability result.
(DOC)

Table S25 GO term groups populated by extracted phosphoproteins in 100 nM MeCh-stimulated CMP-state SH-SY5Y cells. GO term groups were considered enriched only if at least two proteins were present in each group and with a probability of ≤0.05. Hybrid GO term group scores were generated by multiplication of the GO term group enrichment score with the negative log_10_ of the probability result.
(DOC)

Table S26 GO term groups populated by extracted phosphoproteins in 1 µM MeCh-stimulated CMP-state SH-SY5Y cells. GO term groups were considered enriched only if at least two proteins were present in each group and with a probability of ≤0.05. Hybrid GO term group scores were generated by multiplication of the GO term group enrichment score with the negative log_10_ of the probability result.
(DOC)

Table S27 GO term groups populated by extracted phosphoproteins in 10 µM MeCh-stimulated CMP-state SH-SY5Y cells. GO term groups were considered enriched only if at least two proteins were present in each group and with a probability of ≤0.05. Hybrid GO term group scores were generated by multiplication of the GO term group enrichment score with the negative log_10_ of the probability result.
(DOC)

Table S28 GO term groups populated by extracted phosphoproteins in 100 µM MeCh-stimulated CMP-state SH-SY5Y cells. GO term groups were considered enriched only if at least two proteins were present in each group and with a probability of ≤0.05. Hybrid GO term group scores were generated by multiplication of the GO term group enrichment score with the negative log_10_ of the probability result.
(DOC)

Table S29 Cumulated significantly populated Gene Ontology term groups generated from dose-dependent acetyl-β-methylcholine-stimulated phosphoproteins in control-state human neuroblastoma SH-SY5Y cells. Ligand stimulation with acetyl-β-methylcholine (MeCh: 10 nM–100 µM) was for 15 minutes before cell lysate protein extraction and titanium dioxide-mediated purification.
(DOC)

Table S30 Cumulated significantly populated Gene Ontology term groups generated from dose-dependent acetyl-β-methylcholine-stimulated phosphoproteins in peroxide (CMP)-treated-state human neuroblastoma SH-SY5Y cells. Ligand stimulation with acetyl-β-methylcholine (MeCh: 10 nM–100 µM) was for 15 minutes before cell lysate protein extraction and titanium dioxide-mediated purification.
(DOC)

Table S31 MeCh dose-unique GO term population in control-state SH-SY5Y cells. The GO term groups uniquely and significantly (*p*≤0.05, n>2 proteins per group) at the specified MeCh dose only, in control-state SH-SY5Y cells are indicated. Hybrid scores for GO term population were generated by multiplication of the GO term enrichment ratio with the –log_10_ of the enrichment probability.
(DOC)

Table S32 MeCh dose-unique GO term population in CMP-state SH-SY5Y cells. The GO term groups uniquely and significantly (*p*≤0.05, n>2 proteins per group) at the specified MeCh dose only, in CMP-state SH-SY5Y cells are indicated. Hybrid scores for GO term population were generated by multiplication of the GO term enrichment ratio with the –log_10_ of the enrichment probability.
(DOC)

Table S33 Canonical signaling pathways populated by extracted phosphoproteins in control-state SH-SY5Y cells. Canonical signaling pathways enriched by phosphoproteins extracted from non-stimulated and MeCh-stimulated control-state SH-SY5Y cells are represented in a simple matrix format. Canonical signaling pathways were considered enriched only if at least two proteins were present in each signaling pathway and with a probability of ≤0.05. Hybrid signaling pathway scores, indicated in specific cells of the matrix, were generated by multiplication of the pathway enrichment ratio with the negative log_10_ of the probability result. Cells in the matrix not possessing a hybrid score were not significantly populated by proteins in that specific stimulation condition.
(DOC)

Table S34 Canonical signaling pathways populated by extracted phosphoproteins in CMP-state SH-SY5Y cells. Canonical signaling pathways enriched by phosphoproteins extracted from non-stimulated and MeCh-stimulated CMP-state SH-SY5Y cells are represented in a simple matrix format. Canonical signaling pathways were considered enriched only if at least two proteins were present in each signaling pathway and with a probability of ≤0.05. Hybrid signaling pathway scores, indicated in specific cells of the matrix, were generated by multiplication of the pathway enrichment ratio with the negative log_10_ of the probability result. Cells in the matrix not possessing a hybrid score were not significantly populated by proteins in that specific stimulation condition.
(DOC)

Table S35 Canonical signaling pathways populated by extracted phosphoproteins unique to non-stimulated or MeCh-stimulated control-state SH-SY5Y cells. Significantly populated canonical signaling pathways, unique to a specific stimulation condition (non-stimulated or with a specific MeCh dose) are listed. Canonical signaling pathways were considered enriched only if at least two proteins were present in each signaling pathway and with a probability of ≤0.05. Hybrid signaling pathway scores indicated were generated by multiplication of the pathway enrichment ratio with the negative log_10_ of the probability result.
(DOC)

Table S36 Canonical signaling pathways populated by extracted phosphoproteins unique to non-stimulated or MeCh-stimulated CMP-state SH-SY5Y cells. Significantly populated canonical signaling pathways, unique to a specific stimulation condition (non-stimulated or with a specific MeCh dose) are listed. Canonical signaling pathways were considered enriched only if at least two proteins were present in each signaling pathway and with a probability of ≤0.05. Hybrid signaling pathway scores indicated were generated by multiplication of the pathway enrichment ratio with the negative log_10_ of the probability result.
(DOC)
