# Correction: VENNTURE–A Novel Venn Diagram Investigational Tool for Multiple Pharmacological Dataset Analysis

**DOI:** 10.1371/annotation/27f1021c-b6f2-4b90-98bc-fcacd2679185

**Published:** 2012-05-31

**Authors:** Bronwen Martin, Wayne Chadwick, Tie Yi, Sung-Soo Park, Daoyuan Lu, Bin Ni, Shekhar Gadkaree, Kathleen Farhang, Kevin G. Becker, Stuart Maudsley

The published VENNTURE link is no longer accurate. VENNTURE version 1.1.0.2 can be accessed freely at the National Institute on Aging (http://www.grc.nia.nih.gov/).

