# Supplementary figures and images for: VENNTURE–A Novel Venn Diagram Investigational Tool for Multiple Pharmacological Dataset Analysis
Source: PLoS One. 2012 May 14;7(5):e36911. doi: 10.1371/journal.pone.0036911 (PMC3351456; doi:10.1371/journal.pone.0036911)

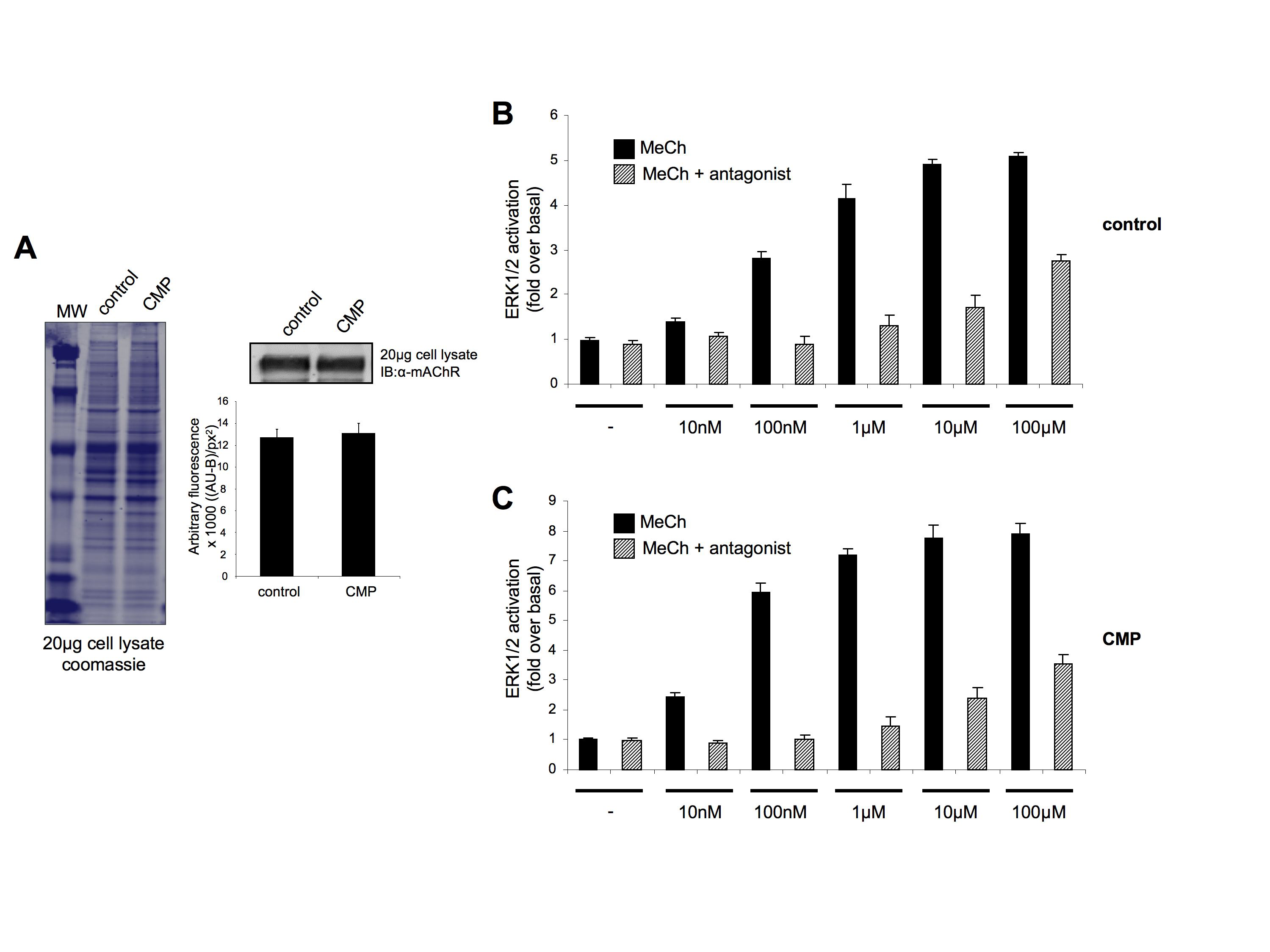

Supplement: Figure S1 — Effects of chronic minimal peroxide (CMP) exposure upon muscarinic receptor activity. (A) Twenty micrograms of SH-SY5Y whole-cell lysate resolved using an SDS-polyacrylamide gel and then stained with coomassie for total protein detection (MW-molecular weight markers). Associated histogram and representative western blot indicate minimal alteration of m1-muscarinic acetylcholine receptor (m1AchR: antisera obtained from Sigma-Aldrich, St. Louis MO) expression (quantified as 1000× arbitrary intensity units (AU) minus background (B) intensity per square pixel (1000×(AU-B)/px2)) with application of CMP protocol (expression controlled using whole-cell coomassie stain). (B) Blockade of acetyl-β-methylcholine (MeCh)-mediated ERK1/2 activation in control-state cells with a 30 minute pre-exposure of cells to the muscarinic receptor antagonist pirenzipine (10 µM: gray shaded bars). (C) Blockade of MeCh-mediated ERK1/2 activation in CMP-state cells with a 30 minute pre-exposure of cells to the muscarinic receptor antagonist pirenzipine (10 µM: shaded bars). (TIF) [file pone.0036911.s001.tif]
