# Supplementary material for: VENNTURE–A Novel Venn Diagram Investigational Tool for Multiple Pharmacological Dataset Analysis
Source: PLoS One. 2012 May 14;7(5):e36911. doi: 10.1371/journal.pone.0036911 (PMC3351456; doi:10.1371/journal.pone.0036911)
Supplement: Table S1 — Phosphoproteins extracted from un-stimulated control-state human neuroblastoma SH-SY5Y cells. For each successfully identified protein official symbol, Uniprot accession code and number of peptides recovered are indicated. (DOC) [file pone.0036911.s002.doc]

**Table S1.** Phosphoproteins extracted from un-stimulated control-state human neuroblastoma SH-SY5Y cells. For each successfully identified protein official symbol, Uniprot accession code and number of peptides recovered are indicated.

| **Protein Identification** | **Symbol** | **Accession** | **Peptide** |
| --- | --- | --- | --- |
| thymopoietin | TMPO | Q9P1N8 | 10 |
| YTH domain containing 1 | YTHDC1 | YTDC1 | 8 |
| leucine rich repeat (in FLII) interacting protein 1 | LRRFIP1 | Q9Y607 | 6 |
| SAM domain, SH3 domain and nuclear localization signals 1 | SAMSN1 | SAMN1 | 6 |
| adenosylmethionine decarboxylase 1 | AMD1 | Q5VXN4 | 5 |
| family with sequence similarity 76, member B | FAM76B | FA76B | 4 |
| inhibitor of Bruton agammaglobulinemia tyrosine kinase | IBTK | IBTK | 4 |
| NFKB activating protein | NKAP | Q05D22 | 4 |
| zinc finger protein 618 | ZNF618 | Q71JB3 | 4 |
| SR-related CTD-associated factor 1 | SCAF1 | Q7Z6I4 | 4 |
| topoisomerase (DNA) II beta 180kDa | TOP2B | Q8WTY5 | 4 |
| neurofilament, medium polypeptide | NEFM | Q9UK51 | 4 |
| ribonucleotide reductase M2 polypeptide | RRM2 | Q9UKM0 | 4 |
| glutamate dehydrogenase 1 | GLUD1 | Q9UQV0 | 4 |
| myelin expression factor 2 | MYEF2 | Q9Y655 | 4 |
| p21 protein (Cdc42/Rac)-activated kinase 1 | PAK1 | Q9Y6B5 | 4 |
| D4, zinc and double PHD fingers family 2 | DPF2 | REQU | 4 |
| solute carrier family 5 (sodium/glucose cotransporter), member 10 | SLC5A10 | SC5AA | 4 |
| sex comb on midleg homolog 1 (Drosophila) | SCMH1 | SCMH1 | 4 |
| SAFB-like, transcription modulator | SLTM | SLTM | 4 |
| suppressor of Ty 5 homolog (S. cerevisiae) | SUPT5H | SPT5H | 4 |
| serine/arginine repetitive matrix 1 | SRRM1 | SRRM1 | 4 |
| peter pan homolog (Drosophila) | PPAN | SSF1 | 4 |
| STIP1 homology and U-box containing protein 1 | STUB1 | STUB1 | 4 |
| synaptopodin 2 | SYNPO2 | SYNP2 | 4 |
| prostaglandin E synthase 3 (cytosolic) | PTGES3 | TEBP | 4 |
| telomeric repeat binding factor 2 | TERF2 | TERF2 | 4 |
| transcription factor CP2 | TFCP2 | TFCP2 | 4 |
| tumor protein p53 binding protein 1 | TP53BP1 | TP53B | 4 |
| TRAF-type zinc finger domain containing 1 | TRAFD1 | TRAD1 | 4 |
| twist homolog 1 (Drosophila) | TWIST1 | TWST1 | 4 |
| thioredoxin domain containing 11 | TXNDC11 | TXD11 | 4 |
| ubiquitin specific peptidase 24 | USP24 | UBP24 | 4 |
| WD repeat domain 43 | WDR43 | WDR43 | 4 |
| zinc finger protein 683 | ZNF683 | ZN683 | 4 |
| zinc finger protein 828 | ZNF828 | ZN828 | 4 |
| beta-1,4-N-acetyl-galactosaminyl transferase 4 | B4GALNT4 | B4GN4 | 3 |
| latrophilin 3 | LPHN3 | Q4W5J9 | 3 |
| similar to RNA binding motif protein 39; RNA binding motif protein 39 | RBM39 | Q68DD9 | 3 |
| similar to Bcl-2-associated transcription factor 1 (Btf); BCL2-associated transcription factor 1 | BCLAF1 | Q6DCA8 | 3 |
| erythrocyte membrane protein band 4.1 like 4B | EPB41L4B | Q9NX84 | 3 |
| similar to chromobox homolog 3; chromobox homolog 3 (HP1 gamma homolog, Drosophila) | CBX3 | A4D177 | 2 |
| glutamate receptor, metabotropic 3 | GRM3 | A4D1D0 | 2 |
| myeloid leukemia factor 2 | MLF2 | A8K1F4 | 2 |
| ataxin 2-like | ATXN2L | A8K1R6 | 2 |
| leucine rich repeat containing 41 | LRRC41 | A8K5G8 | 2 |
| SUMO1/sentrin specific peptidase 7 | SENP7 | A8MW39 | 2 |
| LIM and calponin homology domains 1 | LIMCH1 | A8MXC3 | 2 |
| dapper, antagonist of beta-catenin, homolog 1 (Xenopus laevis) | DACT1 | A8MYJ2 | 2 |
| hypothetical protein LOC387763 | AG2 | AG2 | 2 |
| A kinase (PRKA) anchor protein 4 | AKAP4 | AKAP4 | 2 |
| anaphase promoting complex subunit 1; similar to anaphase promoting complex subunit 1 | ANAPC1 | APC1 | 2 |
| Rho guanine nucleotide exchange factor (GEF) 12 | ARHGEF12 | ARHGC | 2 |
| CDC42 effector protein (Rho GTPase binding) 4 | CDC42EP4 | B2R6D8 | 2 |
| coiled-coil domain containing 88B | CCDC88B | B2RTU8 | 2 |
| synaptopodin 2 | SYNPO2 | B2RWP6 | 2 |
| zinc finger protein 8 | ZNF8 | B3KS94 | 2 |
| Sp6 transcription factor | SP6 | B3KXP2 | 2 |
| shroom family member 3 | SHROOM3 | B3KY47 | 2 |
| ADP-ribosylation-like factor 6 interacting protein 4 | ARL6IP4 | B3V0L0 | 2 |
| FIP1 like 1 (S. cerevisiae) | FIP1L1 | B4DIR3 | 2 |
| collagen, type VI, alpha 3 | COL6A3 | B4E3U5 | 2 |
| stathmin 1 | STMN1 | B7Z8N4 | 2 |
| bromodomain adjacent to zinc finger domain, 1B | BAZ1B | BAZ1B | 2 |
| B double prime 1, subunit of RNA polymerase III transcription initiation factor IIIB | BDP1 | BDP1 | 2 |
| bromodomain containing 3 | BRD3 | BRD3 | 2 |
| chromosome 17 open reading frame 49 | C17orf49 | C9J4G0 | 2 |
| cyclin L1 | CCNL1 | CCNL1 | 2 |
| cyclin Y | CCNY | CCNY | 2 |
| cyclin Y-like 1 | CCNYL1 | CCYL1 | 2 |
| chromosome 6 open reading frame 223 | C6orf223 | CF223 | 2 |
| calcium regulated heat stable protein 1, 24kDa | CARHSP1 | CHSP1 | 2 |
| COMM domain containing 5 | COMMD5 | COMD5 | 2 |
| Ctr9, Paf1/RNA polymerase II complex component, homolog (S. cerevisiae) | CTR9 | CTR9 | 2 |
| chromosome X open reading frame 58 | CXorf58 | CX058 | 2 |
| dyskeratosis congenita 1, dyskerin | DKC1 | DKC1 | 2 |
| drebrin 1 | DBN1 | DREB | 2 |
| dynein, axonemal, heavy chain 1 | DNAH1 | DYH1 | 2 |
| EPM2A (laforin) interacting protein 1 | EPM2AIP1 | EPMIP | 2 |
| glucocorticoid induced transcript 1 | GLCCI1 | GLCI1 | 2 |
| G protein regulated inducer of neurite outgrowth 1 | GPRIN1 | GRIN1 | 2 |
| glucocorticoid receptor DNA binding factor 1 | GRLF1 | GRLF1 | 2 |
| heat shock protein 90kDa alpha (cytosolic), class B member 2 (pseudogene) | HSP90AB2P | H90B2 | 2 |
| heat shock 27kDa protein-like 2 pseudogene; heat shock 27kDa protein 1 | HSPB1 | HSPB1 | 2 |
| chloride channel, nucleotide-sensitive, 1A | CLNS1A | ICLN | 2 |
| KIAA0947 | KIAA0947 | K0947 | 2 |
| potassium channel tetramerisation domain containing 15 | KCTD15 | KCD15 | 2 |
| potassium large conductance calcium-activated channel, subfamily M, beta member 1 | KCNMB1 | KCMB1 | 2 |
| lamin B receptor | LBR | LBR | 2 |
| LIM domain binding 1 | LDB1 | LDB1 | 2 |
| lipin 3 | LPIN3 | LPIN3 | 2 |
| leucine zipper, putative tumor suppressor 1 | LZTS1 | LZTS1 | 2 |
| MARCKS-like 1 | MARCKSL1 | MRP | 2 |
| mucin 12, cell surface associated; similar to mucin 11 | MUC12 | MUC12 | 2 |
| NIMA (never in mitosis gene a)-related kinase 5 | NEK5 | NEK5 | 2 |
| cofactor of BRCA1 | COBRA1 | NELFB | 2 |
| PDZ and LIM domain 4 | PDLIM4 | PDLI4 | 2 |
| PDS5, regulator of cohesion maintenance, homolog B (S. cerevisiae) | PDS5B | PDS5B | 2 |
| periphilin 1 | PPHLN1 | PPHLN | 2 |
| peptidylprolyl isomerase domain and WD repeat containing 1 | PPWD1 | PPWD1 | 2 |
| protein tyrosine phosphatase-like A domain containing 1 | PTPLAD1 | PTAD1 | 2 |
| serine/arginine repetitive matrix 2; hypothetical LOC100132779 | SRRM2 | Q05BI2 | 2 |
| tumor protein p53 binding protein, 2 | TP53BP2 | Q05BL1 | 2 |
| myristoylated alanine-rich protein kinase C substrate | MARCKS | Q05C82 | 2 |
| centrosomal protein 350kDa | CEP350 | Q05D72 | 2 |
| ring finger protein 20 | RNF20 | Q05DC0 | 2 |
| remodeling and spacing factor 1 | RSF1 | Q05DG0 | 2 |
| ubiquitin specific peptidase 42 | USP42 | Q0VIN8 | 2 |
| heterogeneous nuclear ribonucleoprotein D (AU-rich element RNA binding protein 1, 37kDa) | HNRNPD | Q12771 | 2 |
| retinoblastoma binding protein 6 | RBBP6 | Q147T5 | 2 |
| spectrin, alpha, non-erythrocytic 1 (alpha-fodrin) | SPTAN1 | Q14917 | 2 |
| thyroid hormone receptor interactor 12 | TRIP12 | Q14CF1 | 2 |
| septin 2 | SEPT2 | Q15019.1 | 2 |
| similar to hCG1820375; PRP4 pre-mRNA processing factor 4 homolog B (yeast) | PRPF4B | Q15456 | 2 |
| Y box binding protein 1 | YBX1 | Q15905 | 2 |
| RNA binding motif protein 25 | RBM25 | Q16083 | 2 |
| protein tyrosine phosphatase, non-receptor type 12 | PTPN12 | Q16128 | 2 |
| ELAV (embryonic lethal, abnormal vision, Drosophila)-like 4 (Hu antigen D) | ELAVL4 | Q16234 | 2 |
| protein tyrosine phosphatase, receptor type, S | PTPRS | Q16343 | 2 |
| TPI1 pseudogene; triosephosphate isomerase 1 | TPI1 | Q2QD09 | 2 |
| ribosomal L1 domain containing 1 | RSL1D1 | Q32Q62 | 2 |
| HECT, UBA and WWE domain containing 1 | HUWE1 | Q3B7K0 | 2 |
| c-abl oncogene 1, receptor tyrosine kinase | ABL1 | Q3B836 | 2 |
| SEC62 homolog (S. cerevisiae) | SEC62 | Q3KR08 | 2 |
| DEAD (Asp-Glu-Ala-Asp) box polypeptide 21 | DDX21 | Q3SWU7 | 2 |
| histidyl-tRNA synthetase 2, mitochondrial (putative); D-tyrosyl-tRNA deacylase 1 homolog (S. cerevisiae) | DTD1 | Q496C9 | 2 |
| proline-rich nuclear receptor coactivator 1 | PNRC1 | Q49A59 | 2 |
| ATPase, class V, type 10A | ATP10A | Q4G0S9 | 2 |
| microtubule-associated protein 1B | MAP1B | Q4VBY4 | 2 |
| telomeric repeat binding factor 2, interacting protein | TERF2IP | Q4W4Y2 | 2 |
| thyroid peroxidase | TPO | Q502Y3 | 2 |
| eukaryotic translation initiation factor 4 gamma, 3 | EIF4G3 | Q504Z1 | 2 |
| myelin associated glycoprotein | MAG | Q53ES7 | 2 |
| basic leucine zipper nuclear factor 1 | BLZF1 | Q53EV6 | 2 |
| cysteine-rich protein 2 | CRIP2 | Q53FN1 | 2 |
| ribosomal protein S3 pseudogene 3; ribosomal protein S3 | RPS3 | Q53G83 | 2 |
| solute carrier family 35, member C2 | SLC35C2 | Q53GK3 | 2 |
| abl interactor 2 | ABI2 | Q53SH3 | 2 |
| general transcription factor IIIC, polypeptide 2, beta 110kDa | GTF3C2 | Q53SY8 | 2 |
| dipeptidyl-peptidase 7 | DPP7 | Q59EM4 | 2 |
| insulin-like growth factor 2 receptor | IGF2R | Q59EZ3 | 2 |
| spectrin, beta, non-erythrocytic 1 | SPTBN1 | Q59FA9 | 2 |
| drebrin-like | DBNL | Q59FH4 | 2 |
| mitogen-activated protein kinase kinase kinase 12 | MAP3K12 | Q59FK7 | 2 |
| microtubule-associated protein 2 | MAP2 | Q59FX9 | 2 |
| Treacher Collins-Franceschetti syndrome 1 | TCOF1 | Q59FZ2 | 2 |
| SWI/SNF related, matrix associated, actin dependent regulator of chromatin, subfamily a, member 4 | SMARCA4 | Q59FZ6 | 2 |
| dihydropyrimidinase-like 2 | DPYSL2 | Q59GB4 | 2 |
| SWI/SNF related, matrix associated, actin dependent regulator of chromatin, subfamily c, member 2 | SMARCC2 | Q59GV3 | 2 |
| apolipoprotein B (including Ag(x) antigen) | APOB | Q59HB3 | 2 |
| nuclear mitotic apparatus protein 1 | NUMA1 | Q59HB8 | 2 |
| FERM, RhoGEF (ARHGEF) and pleckstrin domain protein 1 (chondrocyte-derived) | FARP1 | Q5JV94 | 2 |
| karyopherin alpha 3 (importin alpha 4) | KPNA3 | Q5JVM9 | 2 |
| hepatoma-derived growth factor (high-mobility group protein 1-like) | HDGF | Q5SZ08 | 2 |
| doublecortin domain containing 2 | DCDC2 | Q5T3Y5 | 2 |
| bystin-like | BYSL | Q5T8J2 | 2 |
| zinc finger CCCH-type containing 13 | ZC3H13 | Q5T9K0 | 2 |
| chromosome 1 open reading frame 26 | C1orf26 | Q5TC96 | 2 |
| zinc finger protein 687 | ZNF687 | Q5VWB8 | 2 |
| sorting nexin 16 | SNX16 | Q658L0 | 2 |
| kinesin family member 21A | KIF21A | Q68D13 | 2 |
| glycogen synthase kinase 3 alpha | GSK3A | Q68D16 | 2 |
| heterogeneous nuclear ribonucleoprotein H1 (H) | HNRNPH1 | Q68DG4 | 2 |
| PTPRF interacting protein, binding protein 2 (liprin beta 2) | PPFIBP2 | Q68DV6 | 2 |
| DnaJ (Hsp40) homolog, subfamily C, member 5 | DNAJC5 | Q6AHX3 | 2 |
| eukaryotic translation initiation factor 3, subunit G | EIF3G | Q6IAM0 | 2 |
| vesicle-associated membrane protein 4 | VAMP4 | Q6IAZ3 | 2 |
| progesterone receptor membrane component 1 | PGRMC1 | Q6IB11 | 2 |
| splicing factor, arginine/serine-rich 11 | SFRS11 | Q6N079 | 2 |
| v-yes-1 Yamaguchi sarcoma viral related oncogene homolog | LYN | Q6NUK7 | 2 |
| laminin, gamma 1 (formerly LAMB2) | LAMC1 | Q6NVY8 | 2 |
| structural maintenance of chromosomes 4 | SMC4 | Q6P169 | 2 |
| kinesin family member 4B; kinesin family member 4A | KIF4A | Q6PKB2 | 2 |
| SON DNA binding protein | SON | Q6PKE0 | 2 |
| protein interacting with cyclin A1 | PROCA1 | Q6PKN3 | 2 |
| myosin, heavy chain 9, non-muscle | MYH9 | Q6ZNL4 | 2 |
| SEC16 homolog A (S. cerevisiae) | SEC16A | Q6ZP13 | 2 |
| GTPase activating protein (SH3 domain) binding protein 1 | G3BP1 | Q6ZP53 | 2 |
| mitogen-activated protein kinase kinase 2 pseudogene; mitogen-activated protein kinase kinase 2 | MAP2K2 | Q6ZPC5 | 2 |
| family with sequence similarity 40, member A | FAM40A | Q6ZU72 | 2 |
| coiled-coil domain containing 86 | CCDC86 | Q6ZW18 | 2 |
| antigen identified by monoclonal antibody Ki-67 | MKI67 | Q70L73 | 2 |
| syntaxin 1A (brain) | STX1A | Q75ME0 | 2 |
| fibrillin 1 | FBN1 | Q75N88 | 2 |
| PWWP domain containing 2A | PWWP2A | Q7Z4D8 | 2 |
| microtubule-associated protein 1A | MAP1A | Q7Z5F9 | 2 |
| thyroid hormone receptor associated protein 3 | THRAP3 | Q7Z5U1 | 2 |
| metastasis associated 1 | MTA1 | Q86SW2 | 2 |
| microtubule-associated protein 4 | MAP4 | Q86Y04 | 2 |
| zinc finger CCCH-type containing 18 | ZC3H18 | Q86YU5 | 2 |
| phosphoinositide-3-kinase adaptor protein 1 | PIK3AP1 | Q86YV3 | 2 |
| glutamate receptor, ionotropic, N-methyl D-aspartate 2C | GRIN2C | Q8IW23 | 2 |
| centromere protein C 1 | CENPC1 | Q8IW27 | 2 |
| pericentrin | PCNT | Q8IWJ7 | 2 |
| KIAA0913 | KIAA0913 | Q8N420 | 2 |
| eukaryotic translation initiation factor 5B | EIF5B | Q8N5A0 | 2 |
| cortactin | CTTN | Q8N707 | 2 |
| similar to RNA binding motif protein, X-linked; similar to hCG2011544; RNA binding motif protein, X-linked | RBMX | Q8N8Y7 | 2 |
| chromosome 7 open reading frame 50 | C7orf50 | Q8TAS9 | 2 |
| chromosome 6 open reading frame 134 | C6orf134 | Q8TAV6 | 2 |
| WD repeat domain 43 | WDR43 | Q8TB67 | 2 |
| GRB2-associated binding protein 1 | GAB1 | Q8TE87 | 2 |
| heparan sulfate proteoglycan 2 | HSPG2 | Q8TEU3 | 2 |
| myosin IXB | MYO9B | Q8WVD2 | 2 |
| wings apart-like homolog (Drosophila) | WAPAL | Q8WVX6 | 2 |
| neural proliferation, differentiation and control, 1 | NPDC1 | Q8WXX4 | 2 |
| microtubule-actin crosslinking factor 1 | MACF1 | Q8WXY4 | 2 |
| nuclear casein kinase and cyclin-dependent kinase substrate 1 | NUCKS1 | Q8WYF8 | 2 |
| spire homolog 1 (Drosophila) | SPIRE1 | Q96AS4 | 2 |
| heterogeneous nuclear ribonucleoprotein U (scaffold attachment factor A) | HNRNPU | Q96BA7 | 2 |
| papillary renal cell carcinoma (translocation-associated) | PRCC | Q96FT4 | 2 |
| paired-like homeobox 2a | PHOX2A | Q96KQ2 | 2 |
| microtubule-associated protein tau | MAPT | Q96N45 | 2 |
| AT rich interactive domain 1A (SWI-like) | ARID1A | Q96SM7 | 2 |
| sorbin and SH3 domain containing 3 | SORBS3 | Q9BT70 | 2 |
| AP2 associated kinase 1 | AAK1 | Q9BUD9 | 2 |
| minichromosome maintenance complex component 2 | MCM2 | Q9BWF4 | 2 |
| phosphoglucomutase 1 | PGM1 | Q9H1D2 | 2 |
| hematological and neurological expressed 1 | HN1 | Q9H3K0 | 2 |
| FERM domain containing 1 | FRMD1 | Q9H645 | 2 |
| mucin 16, cell surface associated | MUC16 | Q9H7S7 | 2 |
| poly(A) polymerase gamma | PAPOLG | Q9H7W6 | 2 |
| SAPS domain family, member 3 | SAPS3 | Q9H880 | 2 |
| pleckstrin homology domain containing, family O member 2 | PLEKHO2 | Q9HBR5 | 2 |
| nucleoporin 98kDa | NUP98 | Q9HDC8 | 2 |
| chromosome 15 open reading frame 39 | C15orf39 | Q9NPN3 | 2 |
| centrosomal protein 170kDa | CEP170 | Q9NSN9 | 2 |
| TBC1 domain family, member 15 | TBC1D15 | Q9NSR4 | 2 |
| single stranded DNA binding protein 3; hypothetical LOC100131851 | SSBP3 | Q9NW25 | 2 |
| arginine/serine-rich coiled-coil 2 | RSRC2 | Q9P068 | 2 |
| Rho guanine nucleotide exchange factor (GEF) 12 | ARHGEF12 | Q9P149 | 2 |
| similar to U5 snRNP-specific protein, 200 kDa; small nuclear ribonucleoprotein 200kDa (U5) | SNRNP200 | Q9P172 | 2 |
