# Supplementary material for: VENNTURE–A Novel Venn Diagram Investigational Tool for Multiple Pharmacological Dataset Analysis
Source: PLoS One. 2012 May 14;7(5):e36911. doi: 10.1371/journal.pone.0036911 (PMC3351456; doi:10.1371/journal.pone.0036911)
Supplement: Table S2 — Phosphoproteins extracted from 10 nM MeCh-treated control-state human neuroblastoma SH-SY5Y cells. For each successfully identified protein official symbol, Uniprot accession code and number of peptides recovered are indicated. (DOC) [file pone.0036911.s003.doc]

**Table S2.** Phosphoproteins extracted from 10nM MeCh-treated control-state human neuroblastoma SH-SY5Y cells. For each successfully identified protein official symbol, Uniprot accession code and number of peptides recovered are indicated.

| **Protein Identification** | **Symbol** | **Accession** | **Peptide** |
| --- | --- | --- | --- |
| fibrinogen-like 2 | FGL2 | Q8WWE4 | 15 |
| nuclear mitotic apparatus protein 1 | NUMA1 | Q9UNL7 | 10 |
| chromodomain helicase DNA binding protein 8 | CHD8 | Q6DKH9 | 9 |
| heat shock protein 90kDa alpha (cytosolic), class B member 2 (pseudogene) | HSP90AB2P | Q58FF8 | 6 |
| HECT, UBA and WWE domain containing 1 | HUWE1 | Q3B7K0 | 5 |
| SEC24 family, member A (S. cerevisiae) | SEC24A | Q96GP7 | 5 |
| myosin, heavy chain 9, non-muscle | MYH9 | Q99529 | 5 |
| spectrin, beta, non-erythrocytic 1 | SPTBN1 | Q53R99 | 4 |
| lamin A/C | LMNA | Q5I6Y6 | 4 |
| heterogeneous nuclear ribonucleoprotein U (scaffold attachment factor A) | HNRNPU | Q5RI19 | 4 |
| cyclin Y-like 1 | CCNYL1 | Q6NX60 | 4 |
| zinc finger CCCH-type containing 14 | ZC3H14 | Q6PUI8 | 4 |
| p21 protein (Cdc42/Rac)-activated kinase 1 | PAK1 | Q86W79 | 4 |
| microtubule-associated protein 2 | MAP2 | Q99976 | 4 |
| hypothetical protein LOC387763 | AG2 | Q7Z7L8 | 3 |
| sorbin and SH3 domain containing 3 | SORBS3 | Q5BJE4 | 3 |
| similar to Bcl-2-associated transcription factor 1 (Btf); BCL2-associated transcription factor 1 | BCLAF1 | Q6DCA8 | 3 |
| thyroid hormone receptor associated protein 3 | THRAP3 | Q6P0P7 | 3 |
| taxilin alpha | TXLNA | Q86T86 | 3 |
| DEAD (Asp-Glu-Ala-Asp) box polypeptide 51 | DDX51 | Q8IXK5 | 3 |
| ADP-ribosylation-like factor 6 interacting protein 4 | ARL6IP4 | Q96BI2 | 3 |
| family with sequence similarity 40, member A | FAM40A | Q96SN2 | 3 |
| progesterone receptor membrane component 1 | PGRMC1 | Q9UGJ9 | 3 |
| v-yes-1 Yamaguchi sarcoma viral related oncogene homolog | LYN | A0AVQ5 | 2 |
| stathmin 1 | STMN1 | A2A2D1 | 2 |
| centrosome and spindle pole associated protein 1 | CSPP1 | A6ND63 | 2 |
| non-SMC condensin II complex, subunit D3 | NCAPD3 | A6NFS2 | 2 |
| glucocorticoid receptor DNA binding factor 1 | GRLF1 | A7E2A4 | 2 |
| potassium channel tetramerisation domain containing 15 | KCTD15 | A8K600 | 2 |
| epsin 3 | EPN3 | A8K6J3 | 2 |
| D4, zinc and double PHD fingers family 2 | DPF2 | A8K7C9 | 2 |
| interferon regulatory factor 2 binding protein 2 | IRF2BP2 | B1AM36 | 2 |
| ribosomal protein S3 pseudogene 3; ribosomal protein S3 | RPS3 | B2R7N5 | 2 |
| flavin containing monooxygenase 3 | FMO3 | B2R816 | 2 |
| RNA binding motif protein 25 | RBM25 | B2RNA8 | 2 |
| sushi, von Willebrand factor type A, EGF and pentraxin domain containing 1 | SVEP1 | B3KQM1 | 2 |
| TBC1 domain family, member 15 | TBC1D15 | B4DMT9 | 2 |
| suppressor of Ty 5 homolog (S. cerevisiae) | SUPT5H | O00267 | 2 |
| TRAF-type zinc finger domain containing 1 | TRAFD1 | O14545 | 2 |
| paired-like homeobox 2a | PHOX2A | O14813 | 2 |
| dyskeratosis congenita 1, dyskerin | DKC1 | O60832 | 2 |
| apoptotic chromatin condensation inducer 1 | ACIN1 | O75158 | 2 |
| protein kinase D3 | PRKD3 | O94806 | 2 |
| structural maintenance of chromosomes 4 | SMC4 | O95752 | 2 |
| eukaryotic translation initiation factor 5B | EIF5B | O95805 | 2 |
| neurofilament, medium polypeptide | NEFM | P07197 | 2 |
| thymopoietin | TMPO | P08919 | 2 |
| ribonucleotide reductase M2 polypeptide | RRM2 | P31350 | 2 |
| cerebellar degeneration-related protein 2, 62kDa | CDR2 | Q01850 | 2 |
| NFKB activating protein | NKAP | Q05D22 | 2 |
| ankyrin 3, node of Ranvier (ankyrin G) | ANK3 | Q12955 | 2 |
| chromatin assembly factor 1, subunit B (p60) | CHAF1B | Q13112 | 2 |
| heterogeneous nuclear ribonucleoprotein D (AU-rich element RNA binding protein 1, 37kDa) | HNRNPD | Q14100 | 2 |
| similar to RNA binding motif protein 39; RNA binding motif protein 39 | RBM39 | Q14498 | 2 |
| phosphoprotein enriched in astrocytes 15 | PEA15 | Q14801 | 2 |
| telomeric repeat binding factor 2 | TERF2 | Q15554 | 2 |
| lymphocyte-specific protein 1 | LSP1 | Q16004 | 2 |
| protein tyrosine phosphatase, non-receptor type 12 | PTPN12 | Q16128 | 2 |
| adducin 1 (alpha) | ADD1 | Q16156 | 2 |
| ELAV (embryonic lethal, abnormal vision, Drosophila)-like 4 (Hu antigen D) | ELAVL4 | Q16234 | 2 |
| diacylglycerol kinase, delta 130kDa | DGKD | Q16760 | 2 |
| thyrotropin-releasing hormone receptor | TRHR | Q2M339 | 2 |
| receptor-interacting serine-threonine kinase 2 | RIPK2 | Q2TU65 | 2 |
| ubiquitin specific peptidase 42 | USP42 | Q3C166 | 2 |
| adaptor-related protein complex 1, mu 1 subunit | AP1M1 | Q4TTY5 | 2 |
| AP2 associated kinase 1 | AAK1 | Q4ZFZ3 | 2 |
| abl interactor 2 | ABI2 | Q53RS4 | 2 |
| thyroid hormone receptor interactor 12 | TRIP12 | Q53TE7 | 2 |
| insulin-like growth factor 2 receptor | IGF2R | Q59EZ3 | 2 |
| topoisomerase (DNA) II beta 180kDa | TOP2B | Q59H80 | 2 |
| cysteinyl-tRNA synthetase | CARS | Q5HYE4 | 2 |
| family with sequence similarity 76, member B | FAM76B | Q5HYJ3 | 2 |
| FERM, RhoGEF (ARHGEF) and pleckstrin domain protein 1 (chondrocyte-derived) | FARP1 | Q5JV94 | 2 |
| karyopherin alpha 3 (importin alpha 4) | KPNA3 | Q5JVN1 | 2 |
| RNA binding motif protein, X-linked 2 | RBMX2 | Q5JY82 | 2 |
| hepatoma-derived growth factor (high-mobility group protein 1-like) | HDGF | Q5SZ07 | 2 |
| bystin-like | BYSL | Q5T8J2 | 2 |
| myeloid leukemia factor 2 | MLF2 | Q5U0N1 | 2 |
| GTPase activating protein (SH3 domain) binding protein 1 | G3BP1 | Q5U0Q1 | 2 |
| wings apart-like homolog (Drosophila) | WAPAL | Q5VSK5 | 2 |
| serine/arginine repetitive matrix 1 | SRRM1 | Q5VVN4 | 2 |
| antigen identified by monoclonal antibody Ki-67 | MKI67 | Q5VWH2 | 2 |
| heterogeneous nuclear ribonucleoprotein H1 (H) | HNRNPH1 | Q68DG4 | 2 |
| eukaryotic translation initiation factor 3, subunit G | EIF3G | Q6IAM0 | 2 |
| heterogeneous nuclear ribonucleoprotein K; similar to heterogeneous nuclear ribonucleoprotein K | HNRNPK | Q6IBN1 | 2 |
| myosin light chain kinase | MYLK | Q6LAL4 | 2 |
| LIM and calponin homology domains 1 | LIMCH1 | Q6N054 | 2 |
| myristoylated alanine-rich protein kinase C substrate | MARCKS | Q6NVI1 | 2 |
| microtubule-associated protein 1B | MAP1B | Q6PJD3 | 2 |
| KH domain containing, RNA binding, signal transduction associated 1 | KHDRBS1 | Q6PJX7 | 2 |
| ring finger and SPRY domain containing 1 | RSPRY1 | Q6UX21 | 2 |
| cortactin | CTTN | Q76MU0 | 2 |
| Y box binding protein 1 | YBX1 | Q7KZ24 | 2 |
| tumor protein p53 binding protein 1 | TP53BP1 | Q7Z3U4 | 2 |
| chromodomain helicase DNA binding protein 7 | CHD7 | Q7Z7Q2 | 2 |
| bromodomain adjacent to zinc finger domain, 1B | BAZ1B | Q86UJ6 | 2 |
| retinoblastoma 1 | RB1 | Q86WG4 | 2 |
| microtubule-associated protein 4 | MAP4 | Q86Y04 | 2 |
| glutamate receptor, ionotropic, N-methyl D-aspartate 2C | GRIN2C | Q8IW23 | 2 |
| Rho GTPase activating protein 15 | ARHGAP15 | Q8IXX1 | 2 |
| zinc finger protein 687 | ZNF687 | Q8N1G0 | 2 |
| thioredoxin domain containing 11 | TXNDC11 | Q8N2Q4 | 2 |
| GLI pathogenesis-related 2 | GLIPR2 | Q8N2S6 | 2 |
| heterogeneous nuclear ribonucleoprotein U-like 2 | HNRNPUL2 | Q8N3B3 | 2 |
| chromosome 4 open reading frame 37 | C4orf37 | Q8N412 | 2 |
| sorting nexin 16 | SNX16 | Q8N4U3 | 2 |
| armadillo repeat containing 3 | ARMC3 | Q8N7B0 | 2 |
| BMP binding endothelial regulator | BMPER | Q8N8U9 | 2 |
| dihydropyrimidinase-like 2 | DPYSL2 | Q8NAN9 | 2 |
| nucleolin | NCL | Q8NB06 | 2 |
| ATP-binding cassette, sub-family C, member 6 pseudogene 2; ATP-binding cassette, sub-family C (CFTR/MRP), member 6 | ABCC6 | Q8TCY8 | 2 |
| kinase non-catalytic C-lobe domain (KIND) containing 1 | KNDC1 | Q8TEE5 | 2 |
| cyclin Y | CCNY | Q8TEX3 | 2 |
| glutamine-fructose-6-phosphate transaminase 1 | GFPT1 | Q8WYR5 | 2 |
| damage-specific DNA binding protein 2, 48kDa | DDB2 | Q92466 | 2 |
| twist homolog 1 (Drosophila) | TWIST1 | Q92487 | 2 |
| H1 histone family, member X | H1FX | Q92522 | 2 |
| TBC1 domain family, member 5 | TBC1D5 | Q92609 | 2 |
| bromodomain containing 3 | BRD3 | Q92645 | 2 |
| eukaryotic translation elongation factor 1 delta (guanine nucleotide exchange protein) | EEF1D | Q969J1 | 2 |
| minichromosome maintenance complex component 2 | MCM2 | Q969W7 | 2 |
| forkhead box A2 | FOXA2 | Q96DF7 | 2 |
| zinc finger CCCH-type containing 18 | ZC3H18 | Q96DG4 | 2 |
| cofactor of BRCA1 | COBRA1 | Q96EW5 | 2 |
| CDC42 effector protein (Rho GTPase binding) 4 | CDC42EP4 | Q96FT3 | 2 |
| RIMS binding protein 2 | RIMBP2 | Q96ID2 | 2 |
| microtubule-actin crosslinking factor 1 | MACF1 | Q96IQ1 | 2 |
| Leo1, Paf1/RNA polymerase II complex component, homolog (S. cerevisiae) | LEO1 | Q96N99 | 2 |
| protein tyrosine phosphatase-like A domain containing 1 | PTPLAD1 | Q96T12 | 2 |
| remodeling and spacing factor 1 | RSF1 | Q96T23 | 2 |
| AT rich interactive domain 1A (SWI-like) | ARID1A | Q96T89 | 2 |
| anaphase promoting complex subunit 1; similar to anaphase promoting complex subunit 1 | ANAPC1 | Q9BSE6 | 2 |
| G protein-coupled receptor kinase interacting ArfGAP 1 | GIT1 | Q9BSI3 | 2 |
| neural proliferation, differentiation and control, 1 | NPDC1 | Q9BTD6 | 2 |
| KIAA1429 | KIAA1429 | Q9BTH4 | 2 |
| single stranded DNA binding protein 3; hypothetical LOC100131851 | SSBP3 | Q9BTM0 | 2 |
| SUMO1/sentrin specific peptidase 7 | SENP7 | Q9C0F6 | 2 |
| FIP1 like 1 (S. cerevisiae) | FIP1L1 | Q9H077 | 2 |
| phosphoglucomutase 1 | PGM1 | Q9H1D2 | 2 |
| nuclear casein kinase and cyclin-dependent kinase substrate 1 | NUCKS1 | Q9H1E3 | 2 |
| SAPS domain family, member 3 | SAPS3 | Q9H2K6 | 2 |
| hematological and neurological expressed 1 | HN1 | Q9H3K0 | 2 |
| DnaJ (Hsp40) homolog, subfamily C, member 5 | DNAJC5 | Q9H3Z5 | 2 |
| coiled-coil domain containing 86 | CCDC86 | Q9H6F5 | 2 |
| epsin 2 | EPN2 | Q9H7Z2 | 2 |
| myelin expression factor 2 | MYEF2 | Q9H922 | 2 |
| ring finger protein 20 | RNF20 | Q9H9Y7 | 2 |
| RAB24, member RAS oncogene family | RAB24 | Q9HAG2 | 2 |
| otoferlin | OTOF | Q9HC10 | 2 |
| peter pan homolog (Drosophila) | PPAN | Q9NQ55 | 2 |
| DEAD (Asp-Glu-Ala-Asp) box polypeptide 21 | DDX21 | Q9NR30 | 2 |
| excision repair cross-complementing rodent repair deficiency, complementation group 5 | ERCC5 | Q9NR54 | 2 |
| eukaryotic translation initiation factor 4E nuclear import factor 1 | EIF4ENIF1 | Q9NRA8 | 2 |
| centrosomal protein 170kDa | CEP170 | Q9NSN9 | 2 |
| KIAA0947 | KIAA0947 | Q9NTH9 | 2 |
| SAFB-like, transcription modulator | SLTM | Q9NWH9 | 2 |
| periphilin 1 | PPHLN1 | Q9NXL4 | 2 |
| kinesin family member 4B; kinesin family member 4A | KIF4A | Q9NY24 | 2 |
| serine/arginine repetitive matrix 2; hypothetical LOC100132779 | SRRM2 | Q9P0G1 | 2 |
| phosphatidylinositol 4-kinase, catalytic, beta | PI4KB | Q9UBF8 | 2 |
| heat shock 27kDa protein-like 2 pseudogene; heat shock 27kDa protein 1 | HSPB1 | Q9UC31 | 2 |
| transcription factor CP2 | TFCP2 | Q9UD75 | 2 |
| similar to hCG1820375; PRP4 pre-mRNA processing factor 4 homolog B (yeast) | PRPF4B | Q9UEE6 | 2 |
| Treacher Collins-Franceschetti syndrome 1 | TCOF1 | Q9UFD4 | 2 |
| golgi reassembly stacking protein 2, 55kDa | GORASP2 | Q9UFW4 | 2 |
| drebrin 1 | DBN1 | Q9UFZ5 | 2 |
| nucleoporin 98kDa | NUP98 | Q9UHX0 | 2 |
| synaptopodin 2 | SYNPO2 | Q9UK89 | 2 |
| SON DNA binding protein | SON | Q9UKP9 | 2 |
| KIAA1211 | KIAA1211 | Q9ULK9 | 2 |
| CD2 (cytoplasmic tail) binding protein 2 | CD2BP2 | Q9ULP2 | 2 |
| DNA (cytosine-5-)-methyltransferase 1 | DNMT1 | Q9UMZ6 | 2 |
| pleckstrin homology domain containing, family A member 6 | PLEKHA6 | Q9Y2H5 | 2 |
| PDS5, regulator of cohesion maintenance, homolog B (S. cerevisiae) | PDS5B | Q9Y2I5 | 2 |
| kelch-like 4 (Drosophila) | KLHL4 | Q9Y3J5 | 2 |
| inhibitor of Bruton agammaglobulinemia tyrosine kinase | IBTK | Q9Y3T8 | 2 |
| ribosomal L1 domain containing 1 | RSL1D1 | Q9Y3Z9 | 2 |
| PCTK1 protein | Pctk1 | BC009852 | 2 |
