# Supplementary material for: VENNTURE–A Novel Venn Diagram Investigational Tool for Multiple Pharmacological Dataset Analysis
Source: PLoS One. 2012 May 14;7(5):e36911. doi: 10.1371/journal.pone.0036911 (PMC3351456; doi:10.1371/journal.pone.0036911)
Supplement: Table S3 — Phosphoproteins extracted from 100 nM MeCh-treated control-state human neuroblastoma SH-SY5Y cells. For each successfully identified protein official symbol, Uniprot accession code and number of peptides recovered are indicated. (DOC) [file pone.0036911.s004.doc]

**Table S3.** Phosphoproteins extracted from 100nM MeCh-treated control-state human neuroblastoma SH-SY5Y cells. For each successfully identified protein official symbol, Uniprot accession code and number of peptides recovered are indicated.

| **Protein Identification** | **Symbol** | **Accession** | **Peptide** |
| --- | --- | --- | --- |
| ADAM metallopeptidase with thrombospondin type 1 motif, 9 | ADAMTS9 | A1L4L0 | 12 |
| stathmin 1 | STMN1 | A2A2D1 | 7 |
| ring finger protein 222 | RNF222 | A6NCQ9 | 4 |
| ghrelin/obestatin prepropeptide | GHRL | A8CF38 | 4 |
| potassium channel tetramerisation domain containing 15 | KCTD15 | A8K600 | 3 |
| D4, zinc and double PHD fingers family 2 | DPF2 | A8K7C9 | 3 |
| neural cell adhesion molecule 1 | NCAM1 | A8K8T8 | 3 |
| ribosomal protein S3 pseudogene 3; ribosomal protein S3 | RPS3 | B2R7N5 | 3 |
| thrombospondin, type I, domain containing 1 | THSD1 | B2RCF5 | 3 |
| chloride channel, nucleotide-sensitive, 1A | CLNS1A | B2RCS9 | 3 |
| chemokine (C-C motif) ligand 14; chemokine (C-C motif) ligand 15 | CCL14 | B2RU34 | 3 |
| SAPS domain family, member 2 | SAPS2 | B7Z7T3 | 3 |
| chromosome 17 open reading frame 49 | C17orf49 | C9J4G0 | 3 |
| suppressor of Ty 5 homolog (S. cerevisiae) | SUPT5H | O00267 | 2 |
| papillary renal cell carcinoma (translocation-associated) | PRCC | O00665 | 2 |
| paired-like homeobox 2a | PHOX2A | O14813 | 2 |
| dyskeratosis congenita 1, dyskerin | DKC1 | O60832 | 2 |
| latrophilin 3 | LPHN3 | O94867 | 2 |
| isocitrate dehydrogenase 3 (NAD+) beta | IDH3B | O95106 | 2 |
| structural maintenance of chromosomes 4 | SMC4 | O95752 | 2 |
| thymopoietin | TMPO | P08919 | 2 |
| tubulin, alpha 4a | TUBA4A | P68366 | 2 |
| catenin (cadherin-associated protein), alpha 1, 102kDa | CTNNA1 | Q12795 | 2 |
| v-ets erythroblastosis virus E26 oncogene homolog 1 (avian) | ETS1 | Q14278 | 2 |
| similar to RNA binding motif protein 39; RNA binding motif protein 39 | RBM39 | Q14498 | 2 |
| phosphoprotein enriched in astrocytes 15 | PEA15 | Q14801 | 2 |
| poly(rC) binding protein 1 | PCBP1 | Q14975 | 2 |
| Ctr9, Paf1/RNA polymerase II complex component, homolog (S. cerevisiae) | CTR9 | Q15015 | 2 |
| prostaglandin E synthase 3 (cytosolic) | PTGES3 | Q15185 | 2 |
| telomeric repeat binding factor 2 | TERF2 | Q15554 | 2 |
| cytohesin 1 interacting protein | CYTIP | Q15630 | 2 |
| ELAV (embryonic lethal, abnormal vision, Drosophila)-like 4 (Hu antigen D) | ELAVL4 | Q16234 | 2 |
| mediator of DNA-damage checkpoint 1 | MDC1 | Q2TAZ4 | 2 |
| ubiquitin specific peptidase 42 | USP42 | Q3C166 | 2 |
| heterogeneous nuclear ribonucleoprotein A1-like 3 | HNRPA1L3 | Q3MI39 | 2 |
| solute carrier family 35, member C2 | SLC35C2 | Q53GK3 | 2 |
| Rho GTPase activating protein 25 | ARHGAP25 | Q53QF7 | 2 |
| thyroid hormone receptor interactor 12 | TRIP12 | Q53TE7 | 2 |
| CUB and Sushi multiple domains 2 | CSMD2 | Q53TY4 | 2 |
| insulin-like growth factor 2 receptor | IGF2R | Q59EZ3 | 2 |
| exosome component 10 | EXOSC10 | Q59G73 | 2 |
| sorbin and SH3 domain containing 3 | SORBS3 | Q5BJE4 | 2 |
| family with sequence similarity 76, member B | FAM76B | Q5HYJ3 | 2 |
| karyopherin alpha 3 (importin alpha 4) | KPNA3 | Q5JVN1 | 2 |
| solute carrier family 25, member 30 | SLC25A30 | Q5SVS4 | 2 |
| hepatoma-derived growth factor (high-mobility group protein 1-like) | HDGF | Q5SZ07 | 2 |
| bystin-like | BYSL | Q5T8J2 | 2 |
| leucine rich repeat containing 41 | LRRC41 | Q5TDF5 | 2 |
| GTPase activating protein (SH3 domain) binding protein 1 | G3BP1 | Q5U0Q1 | 2 |
| nucleolar and coiled-body phosphoprotein 1 | NOLC1 | Q5VV70 | 2 |
| serine/arginine repetitive matrix 1 | SRRM1 | Q5VVN4 | 2 |
| human immunodeficiency virus type I enhancer binding protein 1 | HIVEP1 | Q5VW60 | 2 |
| antigen identified by monoclonal antibody Ki-67 | MKI67 | Q5VWH2 | 2 |
| ubiquitin-like with PHD and ring finger domains 2 | UHRF2 | Q5VYR1 | 2 |
| ribonucleotide reductase M2 polypeptide | RRM2 | Q5WRU7 | 2 |
| heterogeneous nuclear ribonucleoprotein H1 (H) | HNRNPH1 | Q68DG4 | 2 |
| IGF-like family member 2 | IGFL2 | Q6B9Z3 | 2 |
| similar to Bcl-2-associated transcription factor 1 (Btf); BCL2-associated transcription factor 1 | BCLAF1 | Q6DCA8 | 2 |
| eukaryotic translation initiation factor 3, subunit G | EIF3G | Q6IAM0 | 2 |
| Nipped-B homolog (Drosophila) | NIPBL | Q6KCD6 | 2 |
| LIM and calponin homology domains 1 | LIMCH1 | Q6N054 | 2 |
| myristoylated alanine-rich protein kinase C substrate | MARCKS | Q6NVI1 | 2 |
| MARCKS-like 1 | MARCKSL1 | Q6NXS5 | 2 |
| UV radiation resistance associated gene | UVRAG | Q6P1X0 | 2 |
| microtubule-associated protein 1B | MAP1B | Q6PJD3 | 2 |
| estrogen receptor binding site associated, antigen, 9 | EBAG9 | Q6R3F1 | 2 |
| family with sequence similarity 83, member H | FAM83H | Q6ZRV2 | 2 |
| zinc finger, C3H1-type containing | ZFC3H1 | Q6ZV36 | 2 |
| synaptopodin | SYNPO | Q71HJ6 | 2 |
| ADAM metallopeptidase domain 22 | ADAM22 | Q75MS7 | 2 |
| titin | TTN | Q7Z2X3 | 2 |
| tumor protein p53 binding protein 1 | TP53BP1 | Q7Z3U4 | 2 |
| hypothetical protein LOC387763 | AG2 | Q7Z7L8 | 2 |
| chromodomain helicase DNA binding protein 7 | CHD7 | Q7Z7Q2 | 2 |
| retinoblastoma 1 | RB1 | Q86WG4 | 2 |
| microtubule-associated protein 4 | MAP4 | Q86Y04 | 2 |
| signal-induced proliferation-associated 1 like 3 | SIPA1L3 | Q8IUV1 | 2 |
| poly (ADP-ribose) polymerase 1 | PARP1 | Q8IUZ9 | 2 |
| DEAD (Asp-Glu-Ala-Asp) box polypeptide 6 | DDX6 | Q8IV96 | 2 |
| chromosome 13 open reading frame 29 | C13orf29 | Q8IVM7 | 2 |
| glutamate receptor, ionotropic, N-methyl D-aspartate 2C | GRIN2C | Q8IW23 | 2 |
| zinc finger protein 683 | ZNF683 | Q8IZ20 | 2 |
| prominin 2 | PROM2 | Q8N271 | 2 |
| chromosome 6 open reading frame 223 | C6orf223 | Q8N575 | 2 |
| NFKB activating protein | NKAP | Q8N5F7 | 2 |
| cyclin Y-like 1 | CCNYL1 | Q8N7R7 | 2 |
| olfactory receptor, family 5, subfamily AR, member 1 | OR5AR1 | Q8NGP9 | 2 |
| cyclin Y | CCNY | Q8TEX3 | 2 |
| kinesin family member 23 | KIF23 | Q8WVP0 | 2 |
| bromodomain containing 3 | BRD3 | Q92645 | 2 |
| ATPase, class V, type 10A | ATP10A | Q969I4 | 2 |
| minichromosome maintenance complex component 2 | MCM2 | Q969W7 | 2 |
| septin 2 | SEPT2 | Q96CB0 | 2 |
| cofactor of BRCA1 | COBRA1 | Q96EW5 | 2 |
| microspherule protein 1 | MCRS1 | Q96EZ8 | 2 |
| cytoplasmic linker associated protein 2 | CLASP2 | Q96F87 | 2 |
| CDC42 effector protein (Rho GTPase binding) 4 | CDC42EP4 | Q96FT3 | 2 |
| SWI/SNF related, matrix associated, actin dependent regulator of chromatin, subfamily c, member 2 | SMARCC2 | Q96GY4 | 2 |
| vesicle-associated membrane protein 4 | VAMP4 | Q96J20 | 2 |
| tight junction protein 3 (zona occludens 3) | TJP3 | Q96KB4 | 2 |
| family with sequence similarity 40, member A | FAM40A | Q96SN2 | 2 |
| protein tyrosine phosphatase-like A domain containing 1 | PTPLAD1 | Q96T12 | 2 |
| remodeling and spacing factor 1 | RSF1 | Q96T23 | 2 |
| AT rich interactive domain 1A (SWI-like) | ARID1A | Q96T89 | 2 |
| microtubule-associated protein 2 | MAP2 | Q99976 | 2 |
| metastasis associated 1 | MTA1 | Q9BRL8 | 2 |
| anaphase promoting complex subunit 1; similar to anaphase promoting complex subunit 1 | ANAPC1 | Q9BSE6 | 2 |
| neural proliferation, differentiation and control, 1 | NPDC1 | Q9BTD6 | 2 |
| KIAA1429 | KIAA1429 | Q9BTH4 | 2 |
| single stranded DNA binding protein 3; hypothetical LOC100131851 | SSBP3 | Q9BTM0 | 2 |
| KRI1 homolog (S. cerevisiae) | KRI1 | Q9BU50 | 2 |
| ribosomal protein L10 | RPL10 | Q9GZW2 | 2 |
| phosphoglucomutase 1 | PGM1 | Q9H1D2 | 2 |
| nuclear casein kinase and cyclin-dependent kinase substrate 1 | NUCKS1 | Q9H1E3 | 2 |
| SAPS domain family, member 3 | SAPS3 | Q9H2K6 | 2 |
| hematological and neurological expressed 1 | HN1 | Q9H3K0 | 2 |
| DnaJ (Hsp40) homolog, subfamily C, member 5 | DNAJC5 | Q9H3Z5 | 2 |
| coiled-coil domain containing 86 | CCDC86 | Q9H6F5 | 2 |
| myelin expression factor 2 | MYEF2 | Q9H922 | 2 |
| ring finger protein 20 | RNF20 | Q9H9Y7 | 2 |
| mediator complex subunit 1 | MED1 | Q9HD39 | 2 |
| peter pan homolog (Drosophila) | PPAN | Q9NQ55 | 2 |
| DEAD (Asp-Glu-Ala-Asp) box polypeptide 21 | DDX21 | Q9NR30 | 2 |
| eukaryotic translation initiation factor 4E nuclear import factor 1 | EIF4ENIF1 | Q9NRA8 | 2 |
| centrosomal protein 170kDa | CEP170 | Q9NSN9 | 2 |
| kinesin family member 4B; kinesin family member 4A | KIF4A | Q9NY24 | 2 |
| similar to hCG1820375; PRP4 pre-mRNA processing factor 4 homolog B (yeast) | PRPF4B | Q9UEE6 | 2 |
| early B-cell factor 1 | EBF1 | Q9UH73 | 2 |
| estrogen receptor 2 (ER beta) | ESR2 | Q9UHD3 | 2 |
| DEAD (Asp-Glu-Ala-Asp) box polypeptide 25 | DDX25 | Q9UHL0 | 2 |
| nucleoporin 98kDa | NUP98 | Q9UHX0 | 2 |
| synaptopodin 2 | SYNPO2 | Q9UK89 | 2 |
| CDC42 effector protein (Rho GTPase binding) 3 | CDC42EP3 | Q9UKI2 | 2 |
| ADAM metallopeptidase domain 22 | ADAM22 | Q9UKK0 | 2 |
| SON DNA binding protein | SON | Q9UKP9 | 2 |
| nuclear mitotic apparatus protein 1 | NUMA1 | Q9UNL7 | 2 |
| thyroid stimulating hormone receptor | TSHR | Q9UPH3 | 2 |
| PDZ and LIM domain 4 | PDLIM4 | Q9Y292 | 2 |
| PDS5, regulator of cohesion maintenance, homolog B (S. cerevisiae) | PDS5B | Q9Y2I5 | 2 |
| thyroid hormone receptor associated protein 3 | THRAP3 | Q9Y2W1 | 2 |
| inhibitor of Bruton agammaglobulinemia tyrosine kinase | IBTK | Q9Y3T8 | 2 |
| ribosomal L1 domain containing 1 | RSL1D1 | Q9Y3Z9 | 2 |
| dynein, cytoplasmic 1, light intermediate chain 1 | DYNC1LI1 | Q9Y6G9 | 2 |
| CNGB1 cyclic nucleotide gated channel beta 1 | RCNC2 | Q14028.2 | 2 |
| phosphatidylinositol 4-kinase, catalytic, alpha pseudogene 2 | PI4KAP2 | A4QPH2.2 | 2 |
| chemokine (C-C motif) ligand 18 (pulmonary and activation-regulated) | PARC | P55774.1 | 2 |
| chromosome 16 open reading frame 53 | PA1 | Q9BTK6.1 | 2 |
