# Supplementary material for: VENNTURE–A Novel Venn Diagram Investigational Tool for Multiple Pharmacological Dataset Analysis
Source: PLoS One. 2012 May 14;7(5):e36911. doi: 10.1371/journal.pone.0036911 (PMC3351456; doi:10.1371/journal.pone.0036911)
Supplement: Table S4 — Phosphoproteins extracted from 1 µM MeCh-treated control-state human neuroblastoma SH-SY5Y cells. For each successfully identified protein official symbol, Uniprot accession code and number of peptides recovered are indicated. (DOC) [file pone.0036911.s005.doc]

**Table S4.** Phosphoproteins extracted from 1µM MeCh-treated control-state human neuroblastoma SH-SY5Y cells. For each successfully identified protein official symbol, Uniprot accession code and number of peptides recovered are indicated.

| **Protein Identification** | **Symbol** | **Accession** | **Peptide** |
| --- | --- | --- | --- |
| v-yes-1 Yamaguchi sarcoma viral related oncogene homolog | LYN | A0AVQ5 | 9 |
| cysteine-rich protein 2 | CRIP2 | A1A4U1 | 8 |
| stathmin 1 | STMN1 | A2A2D1 | 5 |
| polymerase (DNA directed) nu | POLN | A2A336 | 4 |
| non-SMC condensin II complex, subunit D3 | NCAPD3 | A6NFS2 | 4 |
| glucocorticoid receptor DNA binding factor 1 | GRLF1 | A7E2A4 | 3 |
| transmembrane protein 200A | TMEM200A | A8K2A1 | 3 |
| leucine rich repeat containing 41 | LRRC41 | A8K5G8 | 3 |
| D4, zinc and double PHD fingers family 2 | DPF2 | A8K7C9 | 2 |
| neural cell adhesion molecule 1 | NCAM1 | A8K8T8 | 2 |
| ribosomal protein S3 pseudogene 3; ribosomal protein S3 | RPS3 | B2R7N5 | 2 |
| chloride channel, nucleotide-sensitive, 1A | CLNS1A | B2RCS9 | 2 |
| resistance to inhibitors of cholinesterase 3 homolog (C. elegans) | RIC3 | B2RD25 | 2 |
| RNA binding motif protein 25 | RBM25 | B2RNA8 | 2 |
| NOL1/NOP2/Sun domain family, member 2 | NSUN2 | B2RNR4 | 2 |
| suppressor of Ty 5 homolog (S. cerevisiae) | SUPT5H | O00267 | 2 |
| paired-like homeobox 2a | PHOX2A | O14813 | 2 |
| zinc finger protein 609 | ZNF609 | O15014 | 2 |
| dyskeratosis congenita 1, dyskerin | DKC1 | O60832 | 2 |
| serpin peptidase inhibitor, clade I (pancpin), member 2 | SERPINI2 | O75830 | 2 |
| EPM2A (laforin) interacting protein 1 | EPM2AIP1 | O94866 | 2 |
| structural maintenance of chromosomes 4 | SMC4 | O95752 | 2 |
| eukaryotic translation initiation factor 5B | EIF5B | O95805 | 2 |
| thymopoietin | TMPO | P08919 | 2 |
| microtubule-associated protein tau | MAPT | P10636 | 2 |
| heterogeneous nuclear ribonucleoprotein C (C1/C2) | HNRNPC | P22628 | 2 |
| TPI1 pseudogene; triosephosphate isomerase 1 | TPI1 | P60174 | 2 |
| tubulin, alpha 4a | TUBA4A | P68366 | 2 |
| glutamyl-prolyl-tRNA synthetase | EPRS | Q05BP6 | 2 |
| NFKB activating protein | NKAP | Q05D22 | 2 |
| mitochondrial translational initiation factor 2 | MTIF2 | Q05DA1 | 2 |
| PCF11, cleavage and polyadenylation factor subunit, homolog (S. cerevisiae) | PCF11 | Q0D2H7 | 2 |
| proteasome (prosome, macropain) 26S subunit, non-ATPase, 2 | PSMD2 | Q13200 | 2 |
| c-abl oncogene 1, receptor tyrosine kinase | ABL1 | Q13688 | 2 |
| bystin-like | BYSL | Q13895 | 2 |
| phosphoprotein enriched in astrocytes 15 | PEA15 | Q14801 | 2 |
| poly(rC) binding protein 1 | PCBP1 | Q14975 | 2 |
| Ctr9, Paf1/RNA polymerase II complex component, homolog (S. cerevisiae) | CTR9 | Q15015 | 2 |
| Sec23 homolog A (S. cerevisiae) | SEC23A | Q15436 | 2 |
| telomeric repeat binding factor 2 | TERF2 | Q15554 | 2 |
| protein tyrosine phosphatase, non-receptor type 12 | PTPN12 | Q16128 | 2 |
| adducin 1 (alpha) | ADD1 | Q16156 | 2 |
| ELAV (embryonic lethal, abnormal vision, Drosophila)-like 4 (Hu antigen D) | ELAVL4 | Q16234 | 2 |
| serpin peptidase inhibitor, clade G (C1 inhibitor), member 1 | SERPING1 | Q16304 | 2 |
| mediator of DNA-damage checkpoint 1 | MDC1 | Q2TAZ4 | 2 |
| calcium regulated heat stable protein 1, 24kDa | CARHSP1 | Q2YDX5 | 2 |
| HECT, UBA and WWE domain containing 1 | HUWE1 | Q3B7K0 | 2 |
| deleted in liver cancer 1 | DLC1 | Q45XF9 | 2 |
| solute carrier family 35, member C2 | SLC35C2 | Q53GK3 | 2 |
| RAD18 homolog (S. cerevisiae) | RAD18 | Q53H10 | 2 |
| potassium voltage-gated channel, subfamily H (eag-related), member 7 | KCNH7 | Q53QU4 | 2 |
| spectrin, beta, non-erythrocytic 1 | SPTBN1 | Q53R99 | 2 |
| abl interactor 2 | ABI2 | Q53RS4 | 2 |
| contactin associated protein-like 5 | CNTNAP5 | Q53RX1 | 2 |
| thyroid hormone receptor interactor 12 | TRIP12 | Q53TE7 | 2 |
| heat shock protein 90kDa alpha (cytosolic), class B member 2 (pseudogene) | HSP90AB2P | Q58FF8 | 2 |
| insulin-like growth factor 2 receptor | IGF2R | Q59EZ3 | 2 |
| erythrocyte membrane protein band 4.1-like 2 | EPB41L2 | Q59FD8 | 2 |
| zinc finger, FYVE domain containing 19 | ZFYVE19 | Q59G85 | 2 |
| topoisomerase (DNA) II beta 180kDa | TOP2B | Q59H80 | 2 |
| sorbin and SH3 domain containing 3 | SORBS3 | Q5BJE4 | 2 |
| matrin 3 | MATR3 | Q5CZA7 | 2 |
| lamin A/C | LMNA | Q5I6Y6 | 2 |
| tight junction associated protein 1 (peripheral) | TJAP1 | Q5JTD1 | 2 |
| FERM, RhoGEF (ARHGEF) and pleckstrin domain protein 1 (chondrocyte-derived) | FARP1 | Q5JV94 | 2 |
| karyopherin alpha 3 (importin alpha 4) | KPNA3 | Q5JVN1 | 2 |
| forkhead-associated (FHA) phosphopeptide binding domain 1 | FHAD1 | Q5JYW1 | 2 |
| heterogeneous nuclear ribonucleoprotein U (scaffold attachment factor A) | HNRNPU | Q5RI19 | 2 |
| hepatoma-derived growth factor (high-mobility group protein 1-like) | HDGF | Q5SZ07 | 2 |
| LEM domain containing 2 | LEMD2 | Q5T972 | 2 |
| myeloid leukemia factor 2 | MLF2 | Q5U0N1 | 2 |
| zinc finger homeobox 4 | ZFHX4 | Q5U3C1 | 2 |
| wings apart-like homolog (Drosophila) | WAPAL | Q5VSK5 | 2 |
| serine/arginine repetitive matrix 1 | SRRM1 | Q5VVN4 | 2 |
| antigen identified by monoclonal antibody Ki-67 | MKI67 | Q5VWH2 | 2 |
| ribonucleotide reductase M2 polypeptide | RRM2 | Q5WRU7 | 2 |
| heterogeneous nuclear ribonucleoprotein H1 (H) | HNRNPH1 | Q68DG4 | 2 |
| similar to Bcl-2-associated transcription factor 1 (Btf); BCL2-associated transcription factor 1 | BCLAF1 | Q6DCA8 | 2 |
| eukaryotic translation initiation factor 3, subunit G | EIF3G | Q6IAM0 | 2 |
| heterogeneous nuclear ribonucleoprotein K; similar to heterogeneous nuclear ribonucleoprotein K | HNRNPK | Q6IBN1 | 2 |
| polybromo 1 | PBRM1 | Q6IRX1 | 2 |
| LIM and calponin homology domains 1 | LIMCH1 | Q6N054 | 2 |
| myristoylated alanine-rich protein kinase C substrate | MARCKS | Q6NVI1 | 2 |
| MARCKS-like 1 | MARCKSL1 | Q6NXS5 | 2 |
| thyroid hormone receptor associated protein 3 | THRAP3 | Q6P0P7 | 2 |
| NIMA (never in mitosis gene a)-related kinase 5 | NEK5 | Q6P3R8 | 2 |
| similar to Serine-protein kinase ATM (Ataxia telangiectasia mutated) | ATM | Q6P7P1 | 2 |
| chromosome 17 open reading frame 97 | C17orf97 | Q6PFW9 | 2 |
| microtubule-associated protein 1B | MAP1B | Q6PJD3 | 2 |
| KH domain containing, RNA binding, signal transduction associated 1 | KHDRBS1 | Q6PJX7 | 2 |
| splicing factor, arginine/serine-rich 11 | SFRS11 | Q6PJY9 | 2 |
| DEAD (Asp-Glu-Ala-Asp) box polypeptide 60 | DDX60 | Q6PK35 | 2 |
| ADAM metallopeptidase with thrombospondin type 1 motif, 13 | ADAMTS13 | Q6QNA7 | 2 |
| estrogen receptor binding site associated, antigen, 9 | EBAG9 | Q6R3F1 | 2 |
| synaptopodin | SYNPO | Q71HJ6 | 2 |
| growth differentiation factor 7 | gdf7 | Q75RY1 | 2 |
| cortactin | CTTN | Q76MU0 | 2 |
| similar to U5 snRNP-specific protein, 200 kDa; small nuclear ribonucleoprotein 200kDa (U5) | SNRNP200 | Q7L5W4 | 2 |
| titin | TTN | Q7Z2X3 | 2 |
| mitogen-activated protein kinase kinase 2 pseudogene; mitogen-activated protein kinase kinase 2 | MAP2K2 | Q7Z370 | 2 |
| tumor protein p53 binding protein 1 | TP53BP1 | Q7Z3U4 | 2 |
| protein tyrosine phosphatase, receptor type, F | PTPRF | Q7Z3X4 | 2 |
| hypothetical protein LOC387763 | AG2 | Q7Z7L8 | 2 |
| taxilin alpha | TXLNA | Q86T86 | 2 |
| bromodomain adjacent to zinc finger domain, 1B | BAZ1B | Q86UJ6 | 2 |
| p21 protein (Cdc42/Rac)-activated kinase 1 | PAK1 | Q86W79 | 2 |
| retinoblastoma 1 | RB1 | Q86WG4 | 2 |
| microtubule-associated protein 4 | MAP4 | Q86Y04 | 2 |
| spinster homolog 2 (Drosophila) | SPNS2 | Q8IVW8 | 2 |
| glutamate receptor, ionotropic, N-methyl D-aspartate 2C | GRIN2C | Q8IW23 | 2 |
| DEAD (Asp-Glu-Ala-Asp) box polypeptide 51 | DDX51 | Q8IXK5 | 2 |
| phosphoinositide-3-kinase, regulatory subunit 3 (gamma) | PIK3R3 | Q8N381 | 2 |
| chromosome 6 open reading frame 223 | C6orf223 | Q8N575 | 2 |
| dihydropyrimidinase-like 2 | DPYSL2 | Q8NAN9 | 2 |
| mucin 20, cell surface associated | MUC20 | Q8NBY6 | 2 |
| chromosome 16 open reading frame 71 | C16orf71 | Q8NCV0 | 2 |
| unc-13 homolog C (C. elegans) | UNC13C | Q8ND48 | 2 |
| olfactory receptor, family 5, subfamily AR, member 1 | OR5AR1 | Q8NGP9 | 2 |
| sphingomyelin synthase 2 | SGMS2 | Q8NHU3 | 2 |
| prospero homeobox 1 | PROX1 | Q8TB91 | 2 |
| cyclin Y | CCNY | Q8TEX3 | 2 |
| H1 histone family, member X | H1FX | Q92522 | 2 |
| bromodomain containing 3 | BRD3 | Q92645 | 2 |
| eukaryotic translation elongation factor 1 delta (guanine nucleotide exchange protein) | EEF1D | Q969J1 | 2 |
| minichromosome maintenance complex component 2 | MCM2 | Q969W7 | 2 |
| ADP-ribosylation-like factor 6 interacting protein 4 | ARL6IP4 | Q96BI2 | 2 |
| coiled-coil domain containing 124 | CCDC124 | Q96CT7 | 2 |
| zinc finger CCCH-type containing 18 | ZC3H18 | Q96DG4 | 2 |
| peptidase M20 domain containing 1 | PM20D1 | Q96DM4 | 2 |
| G protein-coupled receptor 110 | GPR110 | Q96DQ1 | 2 |
| cofactor of BRCA1 | COBRA1 | Q96EW5 | 2 |
| CDC42 effector protein (Rho GTPase binding) 4 | CDC42EP4 | Q96FT3 | 2 |
| vesicle-associated membrane protein 4 | VAMP4 | Q96J20 | 2 |
| nuclear factor I/B | NFIB | Q96J45 | 2 |
| beta-1,4-N-acetyl-galactosaminyl transferase 4 | B4GALNT4 | Q96LV2 | 2 |
| G protein regulated inducer of neurite outgrowth 1 | GPRIN1 | Q96PZ4 | 2 |
| family with sequence similarity 40, member A | FAM40A | Q96SN2 | 2 |
| protein tyrosine phosphatase-like A domain containing 1 | PTPLAD1 | Q96T12 | 2 |
| remodeling and spacing factor 1 | RSF1 | Q96T23 | 2 |
| AT rich interactive domain 1A (SWI-like) | ARID1A | Q96T89 | 2 |
| myosin, heavy chain 9, non-muscle | MYH9 | Q99529 | 2 |
| microtubule-associated protein 2 | MAP2 | Q99976 | 2 |
| anaphase promoting complex subunit 1; similar to anaphase promoting complex subunit 1 | ANAPC1 | Q9BSE6 | 2 |
| neural proliferation, differentiation and control, 1 | NPDC1 | Q9BTD6 | 2 |
| single stranded DNA binding protein 3; hypothetical LOC100131851 | SSBP3 | Q9BTM0 | 2 |
| von Willebrand factor A domain containing 5B2 | VWA5B2 | Q9BVH8 | 2 |
| dedicator of cytokinesis 7 | DOCK7 | Q9C092 | 2 |
| FIP1 like 1 (S. cerevisiae) | FIP1L1 | Q9H077 | 2 |
| phosphoglucomutase 1 | PGM1 | Q9H1D2 | 2 |
| nuclear casein kinase and cyclin-dependent kinase substrate 1 | NUCKS1 | Q9H1E3 | 2 |
| SAPS domain family, member 3 | SAPS3 | Q9H2K6 | 2 |
| G protein-coupled receptor 18 | GPR18 | Q9H2L2 | 2 |
| hematological and neurological expressed 1 | HN1 | Q9H3K0 | 2 |
| DnaJ (Hsp40) homolog, subfamily C, member 5 | DNAJC5 | Q9H3Z5 | 2 |
| retinoblastoma binding protein 6 | RBBP6 | Q9H5M5 | 2 |
| coiled-coil domain containing 86 | CCDC86 | Q9H6F5 | 2 |
| myelin expression factor 2 | MYEF2 | Q9H922 | 2 |
| polymerase (RNA) I polypeptide B, 128kDa | POLR1B | Q9H9Y6 | 2 |
| ring finger protein 20 | RNF20 | Q9H9Y7 | 2 |
| pumilio homolog 2 (Drosophila) | PUM2 | Q9HAN2 | 2 |
| chromobox homolog 8 (Pc class homolog, Drosophila) | CBX8 | Q9NR07 | 2 |
| DEAD (Asp-Glu-Ala-Asp) box polypeptide 21 | DDX21 | Q9NR30 | 2 |
| excision repair cross-complementing rodent repair deficiency, complementation group 5 | ERCC5 | Q9NR54 | 2 |
| centrosomal protein 170kDa | CEP170 | Q9NSN9 | 2 |
| KIAA0947 | KIAA0947 | Q9NTH9 | 2 |
| v-myc myelocytomatosis viral oncogene homolog 1, lung carcinoma derived (avian) | MYCL1 | Q9NUE9 | 2 |
| SAFB-like, transcription modulator | SLTM | Q9NWH9 | 2 |
| ubiquitin specific peptidase 24 | USP24 | Q9NXD1 | 2 |
| periphilin 1 | PPHLN1 | Q9NXL4 | 2 |
| kinesin family member 4B; kinesin family member 4A | KIF4A | Q9NY24 | 2 |
| serine/arginine repetitive matrix 2; hypothetical LOC100132779 | SRRM2 | Q9P0G1 | 2 |
| KN motif and ankyrin repeat domains 2 | KANK2 | Q9P210 | 2 |
| heat shock 27kDa protein-like 2 pseudogene; heat shock 27kDa protein 1 | HSPB1 | Q9UC31 | 2 |
| similar to hCG1820375; PRP4 pre-mRNA processing factor 4 homolog B (yeast) | PRPF4B | Q9UEE6 | 2 |
| Treacher Collins-Franceschetti syndrome 1 | TCOF1 | Q9UFD4 | 2 |
| chromosome 20 open reading frame 26 | C20orf26 | Q9UFV8 | 2 |
| drebrin 1 | DBN1 | Q9UFZ5 | 2 |
| progesterone receptor membrane component 1 | PGRMC1 | Q9UGJ9 | 2 |
| LIM domain binding 1 | LDB1 | Q9UGM4 | 2 |
| early B-cell factor 1 | EBF1 | Q9UH73 | 2 |
| nucleoporin 98kDa | NUP98 | Q9UHX0 | 2 |
| cyclin L1 | CCNL1 | Q9UK58 | 2 |
| synaptopodin 2 | SYNPO2 | Q9UK89 | 2 |
| SON DNA binding protein | SON | Q9UKP9 | 2 |
| nuclear mitotic apparatus protein 1 | NUMA1 | Q9UNL7 | 2 |
| pleckstrin homology domain containing, family A member 6 | PLEKHA6 | Q9Y2H5 | 2 |
| PDS5, regulator of cohesion maintenance, homolog B (S. cerevisiae) | PDS5B | Q9Y2I5 | 2 |
| inhibitor of Bruton agammaglobulinemia tyrosine kinase | IBTK | Q9Y3T8 | 2 |
| ribosomal L1 domain containing 1 | RSL1D1 | Q9Y3Z9 | 2 |
| zinc finger CCCH-type containing 4 | ZC3H4 | Q9Y420 | 2 |
| myotubularin related protein 4 | MTMR4 | Q9Y4D5 | 2 |
| REX1, RNA exonuclease 1 homolog (S. cerevisiae)-like 2 (pseudogene) | REXO1L2P | A0PJM3.2 | 2 |
| ras homolog gene family, member B | MST081 | Q7Z4F6 | 2 |
| heat shock transcription factor 1 | HSF1 | Q3KQR6 | 2 |
| Ral GTPase activating protein, alpha subunit 1 (catalytic) | GARNL1 | Q9H984 | 2 |
| B1 protein | B1 | X84838.1 | 2 |
