# Supplementary material for: VENNTURE–A Novel Venn Diagram Investigational Tool for Multiple Pharmacological Dataset Analysis
Source: PLoS One. 2012 May 14;7(5):e36911. doi: 10.1371/journal.pone.0036911 (PMC3351456; doi:10.1371/journal.pone.0036911)
Supplement: Table S5 — Phosphoproteins extracted from 10 µM MeCh-treated control-state human neuroblastoma SH-SY5Y cells. For each successfully identified protein official symbol, Uniprot accession code and number of peptides recovered are indicated. (DOC) [file pone.0036911.s006.doc]

**Table S5.** Phosphoproteins extracted from 10µM MeCh-treated control-state human neuroblastoma SH-SY5Y cells. For each successfully identified protein official symbol, Uniprot accession code and number of peptides recovered are indicated.

| **Protein Identification** | **Symbol** | **Accession** | **Peptide** |
| --- | --- | --- | --- |
| v-yes-1 Yamaguchi sarcoma viral related oncogene homolog | LYN | A0AVQ5 | 11 |
| cysteine-rich protein 2 | CRIP2 | A1A4U1 | 9 |
| stathmin 1 | STMN1 | A2A2D1 | 6 |
| ring finger protein 222 | RNF222 | A6NCQ9 | 5 |
| centrosome and spindle pole associated protein 1 | CSPP1 | A6ND63 | 5 |
| non-SMC condensin II complex, subunit D3 | NCAPD3 | A6NFS2 | 4 |
| glucocorticoid receptor DNA binding factor 1 | GRLF1 | A7E2A4 | 4 |
| DEAD (Asp-Glu-Ala-Asp) box polypeptide 3, X-linked | DDX3X | A8K538 | 4 |
| potassium channel tetramerisation domain containing 15 | KCTD15 | A8K600 | 4 |
| D4, zinc and double PHD fingers family 2 | DPF2 | A8K7C9 | 4 |
| ribosomal protein S3 pseudogene 3; ribosomal protein S3 | RPS3 | B2R7N5 | 3 |
| chloride channel, nucleotide-sensitive, 1A | CLNS1A | B2RCS9 | 3 |
| RNA binding motif protein 25 | RBM25 | B2RNA8 | 3 |
| defensin, beta 132 | DEFB132 | B2RP72 | 3 |
| SWI/SNF related, matrix associated, actin dependent regulator of chromatin, subfamily a, member 4 | SMARCA4 | B3KNW7 | 3 |
| kinesin light chain 4 | KLC4 | B3KSQ3 | 3 |
| transducin-like enhancer of split 3 (E(sp1) homolog, Drosophila) | TLE3 | B3KUA2 | 3 |
| SAPS domain family, member 2 | SAPS2 | B7Z7T3 | 3 |
| chromosome 17 open reading frame 49 | C17orf49 | C9J4G0 | 3 |
| suppressor of Ty 5 homolog (S. cerevisiae) | SUPT5H | O00267 | 3 |
| TRAF-type zinc finger domain containing 1 | TRAFD1 | O14545 | 3 |
| paired-like homeobox 2a | PHOX2A | O14813 | 2 |
| glycogen synthase kinase 3 alpha | GSK3A | O14959 | 2 |
| zinc finger protein 354A | ZNF354A | O60765 | 2 |
| dyskeratosis congenita 1, dyskerin | DKC1 | O60832 | 2 |
| ATPase, Ca++ transporting, type 2C, member 2 | ATP2C2 | O75185 | 2 |
| ankyrin repeat domain 24 | ANKRD24 | O75268 | 2 |
| protein kinase D3 | PRKD3 | O94806 | 2 |
| structural maintenance of chromosomes 4 | SMC4 | O95752 | 2 |
| eukaryotic translation initiation factor 5B | EIF5B | O95805 | 2 |
| thymopoietin | TMPO | P08919 | 2 |
| translocated promoter region (to activated MET oncogene) | TPR | P12270 | 2 |
| phosphatidylinositol glycan anchor biosynthesis, class A | PIGA | P37287 | 2 |
| thymopoietin | TMPO | P42167 | 2 |
| TPI1 pseudogene; triosephosphate isomerase 1 | TPI1 | P60174 | 2 |
| potassium large conductance calcium-activated channel, subfamily M, beta member 1 | KCNMB1 | P78475 | 2 |
| glycophorin A (MNS blood group) | GYPA | Q03867 | 2 |
| family with sequence similarity 178, member A | FAM178A | Q05BG6 | 2 |
| glutamyl-prolyl-tRNA synthetase | EPRS | Q05BP6 | 2 |
| golgi-specific brefeldin A resistant guanine nucleotide exchange factor 1 | GBF1 | Q05BW6 | 2 |
| NFKB activating protein | NKAP | Q05D22 | 2 |
| PCF11, cleavage and polyadenylation factor subunit, homolog (S. cerevisiae) | PCF11 | Q0D2H7 | 2 |
| catenin (cadherin-associated protein), alpha 1, 102kDa | CTNNA1 | Q12795 | 2 |
| p21 protein (Cdc42/Rac)-activated kinase 2 | PAK2 | Q13154 | 2 |
| dual specificity phosphatase 4 | DUSP4 | Q13649 | 2 |
| ceruloplasmin (ferroxidase) | CP | Q14063 | 2 |
| phosphoprotein enriched in astrocytes 15 | PEA15 | Q14801 | 2 |
| mitogen-activated protein kinase binding protein 1 | MAPKBP1 | Q14CD8 | 2 |
| Ctr9, Paf1/RNA polymerase II complex component, homolog (S. cerevisiae) | CTR9 | Q15015 | 2 |
| prostaglandin E synthase 3 (cytosolic) | PTGES3 | Q15185 | 2 |
| telomeric repeat binding factor 2 | TERF2 | Q15554 | 2 |
| luteinizing hormone/choriogonadotropin receptor | LHCGR | Q15996 | 2 |
| protein tyrosine phosphatase, non-receptor type 12 | PTPN12 | Q16128 | 2 |
| adducin 1 (alpha) | ADD1 | Q16156 | 2 |
| ELAV (embryonic lethal, abnormal vision, Drosophila)-like 4 (Hu antigen D) | ELAVL4 | Q16234 | 2 |
| KIAA0528 | KIAA0528 | Q17RY7 | 2 |
| receptor-interacting serine-threonine kinase 2 | RIPK2 | Q2TU65 | 2 |
| HECT, UBA and WWE domain containing 1 | HUWE1 | Q3B7K0 | 2 |
| heterogeneous nuclear ribonucleoprotein A1-like 3 | HNRPA1L3 | Q3MI39 | 2 |
| pleckstrin homology domain containing, family N member 1 | PLEKHN1 | Q494U1 | 2 |
| KIAA1377 | KIAA1377 | Q4G0U6 | 2 |
| hyperpolarization activated cyclic nucleotide-gated potassium channel 3 | HCN3 | Q4VX12 | 2 |
| AP2 associated kinase 1 | AAK1 | Q4ZFZ3 | 2 |
| Bloom syndrome, RecQ helicase-like | BLM | Q52M96 | 2 |
| RNA binding protein, autoantigenic (hnRNP-associated with lethal yellow homolog (mouse)) | RALY | Q53GL6 | 2 |
| general transcription factor IIIC, polypeptide 2, beta 110kDa | GTF3C2 | Q53QN0 | 2 |
| thyroid hormone receptor interactor 12 | TRIP12 | Q53TE7 | 2 |
| mitochondrial ribosomal protein S12 | MRPS12 | Q53X98 | 2 |
| heat shock protein 90kDa alpha (cytosolic), class B member 2 (pseudogene) | HSP90AB2P | Q58FF8 | 2 |
| insulin-like growth factor 2 receptor | IGF2R | Q59EZ3 | 2 |
| topoisomerase (DNA) II beta 180kDa | TOP2B | Q59H80 | 2 |
| sorbin and SH3 domain containing 3 | SORBS3 | Q5BJE4 | 2 |
| family with sequence similarity 76, member B | FAM76B | Q5HYJ3 | 2 |
| karyopherin alpha 3 (importin alpha 4) | KPNA3 | Q5JVN1 | 2 |
| ATP-binding cassette, sub-family A (ABC1), member 2 | ABCA2 | Q5SPZ4 | 2 |
| death-domain associated protein | DAXX | Q5STR5 | 2 |
| PDZ domain containing 1 | PDZK1 | Q5T2W1 | 2 |
| bystin-like | BYSL | Q5T8J2 | 2 |
| wingless-type MMTV integration site family, member 2B | WNT2B | Q5TEH9 | 2 |
| DNA methyltransferase 1 associated protein 1 | DMAP1 | Q5TG40 | 2 |
| GTPase activating protein (SH3 domain) binding protein 1 | G3BP1 | Q5U0Q1 | 2 |
| serine/arginine repetitive matrix 1 | SRRM1 | Q5VVN4 | 2 |
| complement component 4 binding protein, beta | C4BPB | Q5VVR0 | 2 |
| antigen identified by monoclonal antibody Ki-67 | MKI67 | Q5VWH2 | 2 |
| ribonucleotide reductase M2 polypeptide | RRM2 | Q5WRU7 | 2 |
| cell division cycle associated 2 | CDCA2 | Q69YH5 | 2 |
| similar to Bcl-2-associated transcription factor 1 (Btf); BCL2-associated transcription factor 1 | BCLAF1 | Q6DCA8 | 2 |
| eukaryotic translation initiation factor 3, subunit G | EIF3G | Q6IAM0 | 2 |
| heterogeneous nuclear ribonucleoprotein K; similar to heterogeneous nuclear ribonucleoprotein K | HNRNPK | Q6IBN1 | 2 |
| LIM and calponin homology domains 1 | LIMCH1 | Q6N054 | 2 |
| complement factor H-related 3 | CFHR3 | Q6NSD3 | 2 |
| myristoylated alanine-rich protein kinase C substrate | MARCKS | Q6NVI1 | 2 |
| MARCKS-like 1 | MARCKSL1 | Q6NXS5 | 2 |
| thyroid hormone receptor associated protein 3 | THRAP3 | Q6P0P7 | 2 |
| NIMA (never in mitosis gene a)-related kinase 5 | NEK5 | Q6P3R8 | 2 |
| microtubule-associated protein 1B | MAP1B | Q6PJD3 | 2 |
| NDC80 homolog, kinetochore complex component (S. cerevisiae) | NDC80 | Q6PJX2 | 2 |
| KH domain containing, RNA binding, signal transduction associated 1 | KHDRBS1 | Q6PJX7 | 2 |
| zinc finger, C3H1-type containing | ZFC3H1 | Q6ZV36 | 2 |
| cortactin | CTTN | Q76MU0 | 2 |
| similar to U5 snRNP-specific protein, 200 kDa; small nuclear ribonucleoprotein 200kDa (U5) | SNRNP200 | Q7L5W4 | 2 |
| KIAA0947 | KIAA0947 | Q7Z3A9 | 2 |
| tumor protein p53 binding protein 1 | TP53BP1 | Q7Z3U4 | 2 |
| hypothetical protein LOC387763 | AG2 | Q7Z7L8 | 2 |
| chromodomain helicase DNA binding protein 7 | CHD7 | Q7Z7Q2 | 2 |
| ELK1, member of ETS oncogene family | ELK1 | Q86SR6 | 2 |
| bromodomain adjacent to zinc finger domain, 1B | BAZ1B | Q86UJ6 | 2 |
| retinoblastoma 1 | RB1 | Q86WG4 | 2 |
| microtubule-associated protein 4 | MAP4 | Q86Y04 | 2 |
| unc-51-like kinase 2 (C. elegans) | ULK2 | Q8IYT8 | 2 |
| chromosome 6 open reading frame 223 | C6orf223 | Q8N575 | 2 |
| cyclin Y-like 1 | CCNYL1 | Q8N7R7 | 2 |
| dedicator of cytokinesis 4 | DOCK4 | Q8NB45 | 2 |
| olfactory receptor, family 5, subfamily AR, member 1 | OR5AR1 | Q8NGP9 | 2 |
| dynein, cytoplasmic 1, light intermediate chain 2 | DYNC1LI2 | Q8TAT3 | 2 |
| mutS homolog 6 (E. coli) | MSH6 | Q8TCX4 | 2 |
| ATP-binding cassette, sub-family C | ABCC6 | Q8TCY8 | 2 |
| FERM domain containing 1 | FRMD1 | Q8TEL2 | 2 |
| cyclin Y | CCNY | Q8TEX3 | 2 |
| telomeric repeat binding factor 2, interacting protein | TERF2IP | Q8WYZ3 | 2 |
| TBC1 domain family, member 5 | TBC1D5 | Q92609 | 2 |
| bromodomain containing 3 | BRD3 | Q92645 | 2 |
| eukaryotic translation elongation factor 1 delta (guanine nucleotide exchange protein) | EEF1D | Q969J1 | 2 |
| minichromosome maintenance complex component 2 | MCM2 | Q969W7 | 2 |
| microtubule associated monoxygenase, calponin and LIM domain containing 3 | MICAL3 | Q96DF2 | 2 |
| G protein-coupled receptor 110 | GPR110 | Q96DQ1 | 2 |
| cofactor of BRCA1 | COBRA1 | Q96EW5 | 2 |
| microspherule protein 1 | MCRS1 | Q96EZ8 | 2 |
| cytoplasmic linker associated protein 2 | CLASP2 | Q96F87 | 2 |
| SWI/SNF related, matrix associated, actin dependent regulator of chromatin, subfamily c, member 2 | SMARCC2 | Q96GY4 | 2 |
| vesicle-associated membrane protein 4 | VAMP4 | Q96J20 | 2 |
| chromosome 1 open reading frame 83 | C1orf83 | Q96MN5 | 2 |
| arginine/serine-rich coiled-coil 1 | RSRC1 | Q96QK2 | 2 |
| family with sequence similarity 40, member A | FAM40A | Q96SN2 | 2 |
| protein tyrosine phosphatase-like A domain containing 1 | PTPLAD1 | Q96T12 | 2 |
| remodeling and spacing factor 1 | RSF1 | Q96T23 | 2 |
| AT rich interactive domain 1A (SWI-like) | ARID1A | Q96T89 | 2 |
| RAD9 homolog A (S. pombe) | RAD9A | Q99638 | 2 |
| chromosome 7 open reading frame 50 | C7orf50 | Q9BRJ6 | 2 |
| anaphase promoting complex subunit 1; similar to anaphase promoting complex subunit 1 | ANAPC1 | Q9BSE6 | 2 |
| neural proliferation, differentiation and control, 1 | NPDC1 | Q9BTD6 | 2 |
| single stranded DNA binding protein 3; hypothetical LOC100131851 | SSBP3 | Q9BTM0 | 2 |
| leucine rich repeat containing 1 | LRRC1 | Q9BTT6 | 2 |
| serine/threonine protein kinase MST4 | MST4 | Q9BXC3 | 2 |
| recombination activating gene 1 | RAG1 | Q9BYY2 | 2 |
| ribosomal protein L10 | RPL10 | Q9GZW2 | 2 |
| FIP1 like 1 (S. cerevisiae) | FIP1L1 | Q9H077 | 2 |
| nuclear casein kinase and cyclin-dependent kinase substrate 1 | NUCKS1 | Q9H1E3 | 2 |
| SAPS domain family, member 3 | SAPS3 | Q9H2K6 | 2 |
| hematological and neurological expressed 1 | HN1 | Q9H3K0 | 2 |
| DnaJ (Hsp40) homolog, subfamily C, member 5 | DNAJC5 | Q9H3Z5 | 2 |
| coiled-coil domain containing 86 | CCDC86 | Q9H6F5 | 2 |
| myelin expression factor 2 | MYEF2 | Q9H922 | 2 |
| ring finger protein 20 | RNF20 | Q9H9Y7 | 2 |
| peter pan homolog (Drosophila) | PPAN | Q9NQ55 | 2 |
| DEAD (Asp-Glu-Ala-Asp) box polypeptide 21 | DDX21 | Q9NR30 | 2 |
| chromosome 21 open reading frame 70; hypothetical LOC729774; hypothetical LOC729535 | C21orf70 | Q9NSI2 | 2 |
| CDKN2A interacting protein | CDKN2AIP | Q9NXV6 | 2 |
| kinesin family member 4B; kinesin family member 4A | KIF4A | Q9NY24 | 2 |
| serine/arginine repetitive matrix 2; hypothetical LOC100132779 | SRRM2 | Q9P0G1 | 2 |
| heat shock 27kDa protein-like 2 pseudogene; heat shock 27kDa protein 1 | HSPB1 | Q9UC31 | 2 |
| similar to hCG1820375; PRP4 pre-mRNA processing factor 4 homolog B (yeast) | PRPF4B | Q9UEE6 | 2 |
| G-protein signaling modulator 1 (AGS3-like, C. elegans) | GPSM1 | Q9UFS8 | 2 |
| drebrin 1 | DBN1 | Q9UFZ5 | 2 |
| progesterone receptor membrane component 1 | PGRMC1 | Q9UGJ9 | 2 |
| nucleoporin 98kDa | NUP98 | Q9UHX0 | 2 |
| synaptopodin 2 | SYNPO2 | Q9UK89 | 2 |
| SON DNA binding protein | SON | Q9UKP9 | 2 |
| DNA (cytosine-5-)-methyltransferase 1 | DNMT1 | Q9UMZ6 | 2 |
| myosin IXA | MYO9A | Q9UNJ2 | 2 |
| nuclear mitotic apparatus protein 1 | NUMA1 | Q9UNL7 | 2 |
| pleckstrin homology domain containing, family A member 6 | PLEKHA6 | Q9Y2H5 | 2 |
| inhibitor of Bruton agammaglobulinemia tyrosine kinase | IBTK | Q9Y3T8 | 2 |
| ribosomal L1 domain containing 1 | RSL1D1 | Q9Y3Z9 | 2 |
| up-regulated gene 4 isoform 1 | URG4 | AY078404.1 | 2 |
| G-protein coupled receptor KPG_008 | KPG_008 | AB041941.1 | 2 |
| autogenous vein graft remodeling associated protein 5 | AVGR5 | AM233521.1 | 2 |
