# Supplementary material for: VENNTURE–A Novel Venn Diagram Investigational Tool for Multiple Pharmacological Dataset Analysis
Source: PLoS One. 2012 May 14;7(5):e36911. doi: 10.1371/journal.pone.0036911 (PMC3351456; doi:10.1371/journal.pone.0036911)
Supplement: Table S6 — Phosphoproteins extracted from 100 µM MeCh-treated control-state human neuroblastoma SH-SY5Y cells. For each successfully identified protein official symbol, Uniprot accession code and number of peptides recovered are indicated. (DOC) [file pone.0036911.s007.doc]

**Table S6.** Phosphoproteins extracted from 100µM MeCh-treated control-state human neuroblastoma SH-SY5Y cells. For each successfully identified protein official symbol, Uniprot accession code and number of peptides recovered are indicated.

| **Protein Identification** | **Symbol** | **Accession** | **Peptide** |
| --- | --- | --- | --- |
| RNA binding motif protein 25 | RBM25 | B2RNA8 | 10 |
| cysteine-rich protein 2 | CRIP2 | A1A4U1 | 8 |
| saitohin | STH | A1L3X7 | 6 |
| stathmin 1 | STMN1 | A2A2D1 | 6 |
| polymerase (DNA directed) nu | POLN | A2A336 | 5 |
| melanoma antigen family B, 16 | MAGEB16 | A2A368 | 5 |
| RAP1 interacting factor homolog (yeast) | RIF1 | A6NC27 | 5 |
| processing of precursor 1, ribonuclease P/MRP subunit (S. cerevisiae) | POP1 | A8K5W9 | 5 |
| D4, zinc and double PHD fingers family 2 | DPF2 | A8K7C9 | 5 |
| v-yes-1 Yamaguchi sarcoma viral related oncogene homolog | LYN | A0AVQ5 | 4 |
| neural cell adhesion molecule 1 | NCAM1 | A8K8T8 | 4 |
| RWD domain containing 3 | RWDD3 | A8K9F0 | 4 |
| ubiquitin fusion degradation 1 like (yeast) | UFD1L | A8MW31 | 4 |
| interferon regulatory factor 2 binding protein 2 | IRF2BP2 | B1AM36 | 4 |
| ribosomal protein S3 pseudogene 3; ribosomal protein S3 | RPS3 | B2R7N5 | 4 |
| NOL1/NOP2/Sun domain family, member 2 | NSUN2 | B2RNR4 | 3 |
| SEC23 interacting protein | SEC23IP | B3KM47 | 3 |
| TBC1 domain family, member 15 | TBC1D15 | B4DMT9 | 3 |
| catenin (cadherin-associated protein), alpha 2 | CTNNA2 | B7Z898 | 3 |
| suppressor of Ty 5 homolog (S. cerevisiae) | SUPT5H | O00267 | 3 |
| TRAF-type zinc finger domain containing 1 | TRAFD1 | O14545 | 3 |
| paired-like homeobox 2a | PHOX2A | O14813 | 3 |
| glycogen synthase kinase 3 alpha | GSK3A | O14959 | 3 |
| RER1 retention in endoplasmic reticulum 1 homolog (S. cerevisiae) | RER1 | O15258 | 3 |
| squamous cell carcinoma antigen recognized by T cells | SART1 | O43290 | 3 |
| dyskeratosis congenita 1, dyskerin | DKC1 | O60832 | 3 |
| apoptotic chromatin condensation inducer 1 | ACIN1 | O75158 | 3 |
| protein kinase D3 | PRKD3 | O94806 | 3 |
| kelch repeat and BTB (POZ) domain containing 11 | KBTBD11 | O94819 | 3 |
| structural maintenance of chromosomes 4 | SMC4 | O95752 | 3 |
| V-set and immunoglobulin domain containing 2 | VSIG2 | O95791 | 2 |
| eukaryotic translation initiation factor 5B | EIF5B | O95805 | 2 |
| thymopoietin | TMPO | P08919 | 2 |
| TPI1 pseudogene; triosephosphate isomerase 1 | TPI1 | P60174 | 2 |
| similar to Acidic leucine-rich nuclear phosphoprotein 32 family member B | ANP32B | P78459 | 2 |
| glutamyl-prolyl-tRNA synthetase | EPRS | Q05BP6 | 2 |
| NFKB activating protein | NKAP | Q05D22 | 2 |
| KIAA1704 | KIAA1704 | Q05D87 | 2 |
| interleukin enhancer binding factor 3, 90kDa | ILF3 | Q12906 | 2 |
| c-abl oncogene 1, receptor tyrosine kinase | ABL1 | Q13688 | 2 |
| heterogeneous nuclear ribonucleoprotein D (AU-rich element RNA binding protein 1, 37kDa) | HNRNPD | Q14100 | 2 |
| similar to RNA binding motif protein 39; RNA binding motif protein 39 | RBM39 | Q14498 | 2 |
| phosphoprotein enriched in astrocytes 15 | PEA15 | Q14801 | 2 |
| poly(rC) binding protein 1 | PCBP1 | Q14975 | 2 |
| Ctr9, Paf1/RNA polymerase II complex component, homolog (S. cerevisiae) | CTR9 | Q15015 | 2 |
| prostaglandin E synthase 3 (cytosolic) | PTGES3 | Q15185 | 2 |
| coiled-coil domain containing 6 | CCDC6 | Q15250 | 2 |
| bromodomain containing 2 | BRD2 | Q15310 | 2 |
| telomeric repeat binding factor 2 | TERF2 | Q15554 | 2 |
| protein tyrosine phosphatase, non-receptor type 12 | PTPN12 | Q16128 | 2 |
| ELAV (embryonic lethal, abnormal vision, Drosophila)-like 4 (Hu antigen D) | ELAVL4 | Q16234 | 2 |
| PWWP domain containing 2A | PWWP2A | Q2HJJ2 | 2 |
| heparan sulfate proteoglycan 2 | HSPG2 | Q2VPA1 | 2 |
| nestin | NES | Q2YDX4 | 2 |
| phosphoribosylglycinamide formyltransferase | GART | Q3B7A7 | 2 |
| HECT, UBA and WWE domain containing 1 | HUWE1 | Q3B7K0 | 2 |
| suppressor of defective silencing 3 homolog (S. cerevisiae) | SUDS3 | Q4KMQ5 | 2 |
| death inducer-obliterator 1 | DIDO1 | Q4VXS2 | 2 |
| AP2 associated kinase 1 | AAK1 | Q4ZFZ3 | 2 |
| LIM and calponin homology domains 1 | LIMCH1 | Q503B5 | 2 |
| solute carrier family 35, member C2 | SLC35C2 | Q53GK3 | 2 |
| RNA binding motif protein 14; RNA binding motif protein 4 | RBM4 | Q53GV1 | 2 |
| RAD18 homolog (S. cerevisiae) | RAD18 | Q53H10 | 2 |
| general transcription factor IIIC, polypeptide 2, beta 110kDa | GTF3C2 | Q53QN0 | 2 |
| spectrin, beta, non-erythrocytic 1 | SPTBN1 | Q53R99 | 2 |
| abl interactor 2 | ABI2 | Q53RS4 | 2 |
| thyroid hormone receptor interactor 12 | TRIP12 | Q53TE7 | 2 |
| heat shock protein 90kDa alpha (cytosolic), class B member 2 (pseudogene) | HSP90AB2P | Q58FF8 | 2 |
| drebrin-like | DBNL | Q59FH4 | 2 |
| topoisomerase (DNA) II beta 180kDa | TOP2B | Q59H80 | 2 |
| sorbin and SH3 domain containing 3 | SORBS3 | Q5BJE4 | 2 |
| family with sequence similarity 76, member B | FAM76B | Q5HYJ3 | 2 |
| lamin A/C | LMNA | Q5I6Y6 | 2 |
| GDNF family receptor alpha 4 | GFRA4 | Q5JT74 | 2 |
| karyopherin alpha 3 (importin alpha 4) | KPNA3 | Q5JVN1 | 2 |
| RNA binding motif protein, X-linked 2 | RBMX2 | Q5JY82 | 2 |
| hepatoma-derived growth factor (high-mobility group protein 1-like) | HDGF | Q5SZ07 | 2 |
| AT rich interactive domain 1A (SWI-like) | ARID1A | Q5T0W3 | 2 |
| bystin-like | BYSL | Q5T8J2 | 2 |
| DMRT-like family A2 | DMRTA2 | Q5TFQ3 | 2 |
| myeloid leukemia factor 2 | MLF2 | Q5U0N1 | 2 |
| GTPase activating protein (SH3 domain) binding protein 1 | G3BP1 | Q5U0Q1 | 2 |
| serine/arginine repetitive matrix 1 | SRRM1 | Q5VVN4 | 2 |
| antigen identified by monoclonal antibody Ki-67 | MKI67 | Q5VWH2 | 2 |
| v-abl Abelson murine leukemia viral oncogene homolog 2 (arg, Abelson-related gene) | ABL2 | Q5W0C5 | 2 |
| ribonucleotide reductase M2 polypeptide | RRM2 | Q5WRU7 | 2 |
| heterogeneous nuclear ribonucleoprotein H1 (H) | HNRNPH1 | Q68DG4 | 2 |
| pleckstrin and Sec7 domain containing 3 | PSD3 | Q6B003 | 2 |
| similar to Bcl-2-associated transcription factor 1 (Btf); BCL2-associated transcription factor 1 | BCLAF1 | Q6DCA8 | 2 |
| jun oncogene | JUN | Q6FHM7 | 2 |
| eukaryotic translation initiation factor 3, subunit G | EIF3G | Q6IAM0 | 2 |
| heterogeneous nuclear ribonucleoprotein K; similar to heterogeneous nuclear ribonucleoprotein K | HNRNPK | Q6IBN1 | 2 |
| polybromo 1 | PBRM1 | Q6IRX1 | 2 |
| LIM and calponin homology domains 1 | LIMCH1 | Q6N054 | 2 |
| myristoylated alanine-rich protein kinase C substrate | MARCKS | Q6NVI1 | 2 |
| MARCKS-like 1 | MARCKSL1 | Q6NXS5 | 2 |
| thyroid hormone receptor associated protein 3 | THRAP3 | Q6P0P7 | 2 |
| PC4 and SFRS1 interacting protein 1 | PSIP1 | Q6P391 | 2 |
| NIMA (never in mitosis gene a)-related kinase 4 | NEK4 | Q6P576 | 2 |
| microtubule-associated protein 1B | MAP1B | Q6PJD3 | 2 |
| KH domain containing, RNA binding, signal transduction associated 1 | KHDRBS1 | Q6PJX7 | 2 |
| zinc finger CCCH-type containing 14 | ZC3H14 | Q6PUI8 | 2 |
| KIAA1324 | KIAA1324 | Q6UXG2 | 2 |
| potassium channel tetramerisation domain containing 1 | KCTD1 | Q719H9 | 2 |
| cortactin | CTTN | Q76MU0 | 2 |
| similar to U5 snRNP-specific protein, 200 kDa; small nuclear ribonucleoprotein 200kDa (U5) | SNRNP200 | Q7L5W4 | 2 |
| alpha thalassemia/mental retardation syndrome X-linked (RAD54 homolog, S. cerevisiae) | ATRX | Q7Z2J1 | 2 |
| titin | TTN | Q7Z2X3 | 2 |
| tumor protein p53 binding protein 1 | TP53BP1 | Q7Z3U4 | 2 |
| methyl-CpG binding domain protein 4 | MBD4 | Q7Z4T3 | 2 |
| tripartite motif-containing 28 | TRIM28 | Q7Z632 | 2 |
| kinesin family member 21A | KIF21A | Q7Z668 | 2 |
| insulin-like growth factor 2 receptor | IGF2R | Q7Z7G9 | 2 |
| hypothetical protein LOC387763 | AG2 | Q7Z7L8 | 2 |
| taxilin alpha | TXLNA | Q86T86 | 2 |
| bromodomain adjacent to zinc finger domain, 1B | BAZ1B | Q86UJ6 | 2 |
| retinoblastoma 1 | RB1 | Q86WG4 | 2 |
| microtubule-associated protein 4 | MAP4 | Q86Y04 | 2 |
| DEAD (Asp-Glu-Ala-Asp) box polypeptide 51 | DDX51 | Q8IXK5 | 2 |
| zinc finger protein 683 | ZNF683 | Q8IZ20 | 2 |
| heterogeneous nuclear ribonucleoprotein U-like 2 | HNRNPUL2 | Q8N3B3 | 2 |
| regulatory factor X, 7 | RFX7 | Q8N3J0 | 2 |
| hypothetical MGC50722 | MGC50722 | Q8N5P7 | 2 |
| cyclin Y-like 1 | CCNYL1 | Q8N7R7 | 2 |
| similar to RNA binding motif protein, X-linked; similar to hCG2011544 | RBMX | Q8N8Y7 | 2 |
| dihydropyrimidinase-like 2 | DPYSL2 | Q8NAN9 | 2 |
| breast carcinoma amplified sequence 3 | BCAS3 | Q8NDR8 | 2 |
| CaM kinase-like vesicle-associated | CAMKV | Q8NDU4 | 2 |
| WD repeat domain 43 | WDR43 | Q8TB67 | 2 |
| prospero homeobox 1 | PROX1 | Q8TB91 | 2 |
| dynein, axonemal, heavy chain 1 | DNAH1 | Q8TEJ4 | 2 |
| cyclin Y | CCNY | Q8TEX3 | 2 |
| damage-specific DNA binding protein 2, 48kDa | DDB2 | Q92466 | 2 |
| H1 histone family, member X | H1FX | Q92522 | 2 |
| TBC1 domain family, member 5 | TBC1D5 | Q92609 | 2 |
| bromodomain containing 3 | BRD3 | Q92645 | 2 |
| minichromosome maintenance complex component 2 | MCM2 | Q969W7 | 2 |
| ADP-ribosylation-like factor 6 interacting protein 4 | ARL6IP4 | Q96BI2 | 2 |
| septin 2 | SEPT2 | Q96CB0 | 2 |
| zinc finger CCCH-type containing 18 | ZC3H18 | Q96DG4 | 2 |
| G protein-coupled receptor 110 | GPR110 | Q96DQ1 | 2 |
| cofactor of BRCA1 | COBRA1 | Q96EW5 | 2 |
| microspherule protein 1 | MCRS1 | Q96EZ8 | 2 |
| glucocorticoid induced transcript 1 | GLCCI1 | Q96FD0 | 2 |
| BCL2/adenovirus E1B 19kDa interacting protein 3 | BNIP3 | Q96GP0 | 2 |
| chromosome 5 open reading frame 30 | C5orf30 | Q96GV9 | 2 |
| SWI/SNF related, matrix associated, actin dependent regulator of chromatin, subfamily c, member 2 | SMARCC2 | Q96GY4 | 2 |
| vesicle-associated membrane protein 4 | VAMP4 | Q96J20 | 2 |
| vacuolar protein sorting 33 homolog B (yeast) | VPS33B | Q96K14 | 2 |
| NK6 homeobox 3 | NKX6-3 | Q96LR0 | 2 |
| chromosome 14 open reading frame 145 | C14orf145 | Q96ML4 | 2 |
| G protein regulated inducer of neurite outgrowth 1 | GPRIN1 | Q96PZ4 | 2 |
| family with sequence similarity 40, member A | FAM40A | Q96SN2 | 2 |
| protein tyrosine phosphatase-like A domain containing 1 | PTPLAD1 | Q96T12 | 2 |
| remodeling and spacing factor 1 | RSF1 | Q96T23 | 2 |
| myosin, heavy chain 9, non-muscle | MYH9 | Q99529 | 2 |
| A kinase (PRKA) anchor protein 12 | AKAP12 | Q99970 | 2 |
| microtubule-associated protein 2 | MAP2 | Q99976 | 2 |
| RD RNA binding protein | RDBP | Q9BQJ6 | 2 |
| RNA binding motif protein 15 | RBM15 | Q9BRA5 | 2 |
| chromosome 7 open reading frame 50 | C7orf50 | Q9BRJ6 | 2 |
| metastasis associated 1 | MTA1 | Q9BRL8 | 2 |
| anaphase promoting complex subunit 1; similar to anaphase promoting complex subunit 1 | ANAPC1 | Q9BSE6 | 2 |
| G protein-coupled receptor kinase interacting ArfGAP 1 | GIT1 | Q9BSI3 | 2 |
| chromosome 11 open reading frame 84 | C11orf84 | Q9BUA3 | 2 |
| polypyrimidine tract binding protein 1 | PTBP1 | Q9BUQ0 | 2 |
| dCMP deaminase | DCTD | Q9BVD8 | 2 |
| Wilms tumor 1 associated protein | WTAP | Q9BZS4 | 2 |
| dedicator of cytokinesis 7 | DOCK7 | Q9C092 | 2 |
| FIP1 like 1 (S. cerevisiae) | FIP1L1 | Q9H077 | 2 |
| nuclear casein kinase and cyclin-dependent kinase substrate 1 | NUCKS1 | Q9H1E3 | 2 |
| SAPS domain family, member 3 | SAPS3 | Q9H2K6 | 2 |
| hematological and neurological expressed 1 | HN1 | Q9H3K0 | 2 |
| DnaJ (Hsp40) homolog, subfamily C, member 5 | DNAJC5 | Q9H3Z5 | 2 |
| cadherin-like 23 | CDH23 | Q9H4K9 | 2 |
| retinoblastoma binding protein 6 | RBBP6 | Q9H5M5 | 2 |
| coiled-coil domain containing 86 | CCDC86 | Q9H6F5 | 2 |
| arginine/serine-rich coiled-coil 2 | RSRC2 | Q9H864 | 2 |
| myelin expression factor 2 | MYEF2 | Q9H922 | 2 |
| ring finger protein 20 | RNF20 | Q9H9Y7 | 2 |
| otoferlin | OTOF | Q9HC10 | 2 |
| peter pan homolog (Drosophila) | PPAN | Q9NQ55 | 2 |
| transcription factor 12 | TCF12 | Q9NQY9 | 2 |
| DEAD (Asp-Glu-Ala-Asp) box polypeptide 21 | DDX21 | Q9NR30 | 2 |
| ankyrin repeat domain 26 | ANKRD26 | Q9NSK9 | 2 |
| centrosomal protein 170kDa | CEP170 | Q9NSN9 | 2 |
| KIAA0947 | KIAA0947 | Q9NTH9 | 2 |
| complement factor H | CFH | Q9NU86 | 2 |
| SAFB-like, transcription modulator | SLTM | Q9NWH9 | 2 |
| periphilin 1 | PPHLN1 | Q9NXL4 | 2 |
| kinesin family member 4B; kinesin family member 4A | KIF4A | Q9NY24 | 2 |
| thymocyte nuclear protein 1 | THYN1 | Q9P016 | 2 |
| serine/arginine repetitive matrix 2; hypothetical LOC100132779 | SRRM2 | Q9P0G1 | 2 |
| heat shock 27kDa protein-like 2 pseudogene; heat shock 27kDa protein 1 | HSPB1 | Q9UC31 | 2 |
| transcription factor CP2 | TFCP2 | Q9UD75 | 2 |
| similar to hCG1820375; PRP4 pre-mRNA processing factor 4 homolog B (yeast) | PRPF4B | Q9UEE6 | 2 |
| Treacher Collins-Franceschetti syndrome 1 | TCOF1 | Q9UFD4 | 2 |
| La ribonucleoprotein domain family, member 1 | LARP1 | Q9UFD7 | 2 |
| G-protein signaling modulator 1 (AGS3-like, C. elegans) | GPSM1 | Q9UFS8 | 2 |
| drebrin 1 | DBN1 | Q9UFZ5 | 2 |
| progesterone receptor membrane component 1 | PGRMC1 | Q9UGJ9 | 2 |
| ubiquitin associated protein 2-like | UBAP2L | Q9UGL5 | 2 |
| nucleoporin 98kDa | NUP98 | Q9UHX0 | 2 |
| cyclin L1 | CCNL1 | Q9UK58 | 2 |
| synaptopodin 2 | SYNPO2 | Q9UK89 | 2 |
| CDC42 effector protein (Rho GTPase binding) 3 | CDC42EP3 | Q9UKI2 | 2 |
| SON DNA binding protein | SON | Q9UKP9 | 2 |
| nucleoporin 50kDa | NUP50 | Q9UKX7 | 2 |
| ubiquitin specific peptidase 31 | USP31 | Q9ULL7 | 2 |
| STIP1 homology and U-box containing protein 1 | STUB1 | Q9UNE7 | 2 |
| nuclear mitotic apparatus protein 1 | NUMA1 | Q9UNL7 | 2 |
| metastasis associated 1 family, member 2 | MTA2 | Q9UQB5 | 2 |
| PDZ and LIM domain 4 | PDLIM4 | Q9Y292 | 2 |
| pleckstrin homology domain containing, family A member 6 | PLEKHA6 | Q9Y2H5 | 2 |
| PDS5, regulator of cohesion maintenance, homolog B (S. cerevisiae) | PDS5B | Q9Y2I5 | 2 |
| inhibitor of Bruton agammaglobulinemia tyrosine kinase | IBTK | Q9Y3T8 | 2 |
| La ribonucleoprotein domain family, member 7 | LARP7 | Q9Y3Z8 | 2 |
| ribosomal L1 domain containing 1 | RSL1D1 | Q9Y3Z9 | 2 |
| neural proliferation, differentiation and control, 1 | NPDC1 | Q9Y434 | 2 |
| dynein, cytoplasmic 1, light intermediate chain 1 | DYNC1LI1 | Q9Y6G9 | 2 |
| solute carrier family 4, sodium bicarbonate cotransporter, member 4 | SLC4A4 | Q9Y6R1 | 2 |
| Hypothetical protein | DKFZp686O16217 | BX640710.1 | 2 |
