# Supplementary material for: VENNTURE–A Novel Venn Diagram Investigational Tool for Multiple Pharmacological Dataset Analysis
Source: PLoS One. 2012 May 14;7(5):e36911. doi: 10.1371/journal.pone.0036911 (PMC3351456; doi:10.1371/journal.pone.0036911)
Supplement: Table S7 — Phosphoproteins extracted from untreated chronic minimal peroxide (CMP)-state human neuroblastoma SH-SY5Y cells. For each successfully identified protein official symbol, Uniprot accession code and number of peptides recovered are indicated. (DOC) [file pone.0036911.s008.doc]

**Table S7.** Phosphoproteins extracted from untreated chronic minimal peroxide (CMP)-state human neuroblastoma SH-SY5Y cells. For each successfully identified protein official symbol, Uniprot accession code and number of peptides recovered are indicated.

| **Protein Identification** | **Symbol** | **Accession** | **Peptide** |
| --- | --- | --- | --- |
| leucine rich repeat (in FLII) interacting protein 2 | LRRFIP2 | Q9Y608 | 24 |
| nuclear receptor co-repressor 2 | NCOR2 | Q9Y5U0 | 24 |
| interferon regulatory factor 2 binding protein 1 | IRF2BP1 | Q9Y4P4 | 17 |
| Dmx-like 1 | DMXL1 | Q9Y485 | 14 |
| ribosomal L1 domain containing 1 | RSL1D1 | Q9Y3Z9 | 14 |
| matrix-remodelling associated 5 | MXRA5 | Q9Y3Y8 | 14 |
| PDS5, regulator of cohesion maintenance, homolog B (S. cerevisiae) | PDS5B | Q9Y2I5 | 13 |
| pleckstrin homology domain containing, family A member 6 | PLEKHA6 | Q9Y2H5 | 10 |
| DIP2 disco-interacting protein 2 homolog C (Drosophila) | DIP2C | Q9Y2E4 | 10 |
| canopy 2 homolog (zebrafish) | CNPY2 | Q9Y2B0 | 10 |
| PDZ and LIM domain 4 | PDLIM4 | Q9Y292 | 9 |
| SMAD family member 5 | SMAD5 | Q9UQA1 | 9 |
| signal-induced proliferation-associated 1 like 1 | SIPA1L1 | Q9UNU4 | 9 |
| nuclear mitotic apparatus protein 1 | NUMA1 | Q9UNL7 | 9 |
| apolipoprotein B (including Ag(x) antigen) | APOB | Q9UMN0 | 8 |
| ankyrin 1, erythrocytic | ANK1 | Q9UMG4 | 8 |
| F-box protein 40 | FBXO40 | Q9ULM5 | 8 |
| ubiquitin specific peptidase 31 | USP31 | Q9ULL7 | 8 |
| KIAA1211 | KIAA1211 | Q9ULK9 | 7 |
| microtubule-associated protein 1A | MAP1A | Q9UL09 | 7 |
| SON DNA binding protein | SON | Q9UKP9 | 7 |
| general transcription factor IIIC, polypeptide 4, 90kDa | GTF3C4 | Q9UKN8 | 6 |
| G patch domain containing 8 | GPATCH8 | Q9UKJ3 | 6 |
| synaptopodin 2 | SYNPO2 | Q9UK89 | 6 |
| cyclin L1 | CCNL1 | Q9UK58 | 6 |
| neurofilament, medium polypeptide | NEFM | Q9UK51 | 6 |
| midline 2 | MID2 | Q9UJR9 | 6 |
| mediator complex subunit 12-like | MED12L | Q9UI69 | 6 |
| nucleoporin 98kDa | NUP98 | Q9UHX0 | 6 |
| EGF-like-domain, multiple 7 | EGFL7 | Q9UHF1 | 6 |
| transcription factor 20 (AR1) | TCF20 | Q9UGU0 | 5 |
| ubiquitin associated protein 2-like | UBAP2L | Q9UGL5 | 5 |
| progesterone receptor membrane component 1 | PGRMC1 | Q9UGJ9 | 5 |
| drebrin 1 | DBN1 | Q9UFZ5 | 5 |
| phospholipase C, gamma 1 | PLCG1 | Q9UFY1 | 5 |
| G-protein signaling modulator 1 (AGS3-like, C. elegans) | GPSM1 | Q9UFS8 | 5 |
| La ribonucleoprotein domain family, member 1 | LARP1 | Q9UFD7 | 5 |
| Treacher Collins-Franceschetti syndrome 1 | TCOF1 | Q9UFD4 | 5 |
| F-box and leucine-rich repeat protein 17 | FBXL17 | Q9UF56 | 5 |
| similar to hCG1820375; PRP4 pre-mRNA processing factor 4 homolog B (yeast) | PRPF4B | Q9UEE6 | 5 |
| transcription factor CP2 | TFCP2 | Q9UD75 | 5 |
| heat shock 27kDa protein-like 2 pseudogene; heat shock 27kDa protein 1 | HSPB1 | Q9UC31 | 5 |
| phosphatidylinositol 4-kinase, catalytic, beta | PI4KB | Q9UBF8 | 5 |
| kelch-like 9 (Drosophila) | KLHL9 | Q9P2J3 | 5 |
| TRM5 tRNA methyltransferase 5 homolog (S. cerevisiae) | TRMT5 | Q9P2F4 | 5 |
| HIG1 hypoxia inducible domain family, member 1B | HIGD1B | Q9P297 | 5 |
| similar to ABT1-associated protein; ESF1, nucleolar pre-rRNA processing protein, homolog (S. cerevisiae) | ESF1 | Q9P1S6 | 5 |
| Rho guanine nucleotide exchange factor (GEF) 12 | ARHGEF12 | Q9P149 | 5 |
| cyclin-dependent kinase-like 3 | CDKL3 | Q9P114 | 5 |
| serine/arginine repetitive matrix 2; hypothetical LOC100132779 | SRRM2 | Q9P0G1 | 5 |
| serologically defined colon cancer antigen 8 | SDCCAG8 | Q9P0F1 | 5 |
| dapper, antagonist of beta-catenin, homolog 1 (Xenopus laevis) | DACT1 | Q9NYF0 | 5 |
| kinesin family member 4B; kinesin family member 4A | KIF4A | Q9NY24 | 5 |
| ubiquitin specific peptidase 24 | USP24 | Q9NXD1 | 4 |
| latrophilin 3 | LPHN3 | Q9NWK5 | 4 |
| SAFB-like, transcription modulator | SLTM | Q9NWH9 | 4 |
| B-cell CLL/lymphoma 11A (zinc finger protein) | BCL11A | Q9NWA7 | 4 |
| kelch-like 13 (Drosophila) | KLHL13 | Q9NW71 | 4 |
| ATP-binding cassette, sub-family F (GCN20), member 3 | ABCF3 | Q9NUQ8 | 4 |
| KIAA1109 | KIAA1109 | Q9NTI4 | 4 |
| KIAA0947 | KIAA0947 | Q9NTH9 | 4 |
| chromosome 12 open reading frame 11 | C12orf11 | Q9NTB6 | 4 |
| SAM domain, SH3 domain and nuclear localization signals 1 | SAMSN1 | Q9NSI8 | 4 |
| eukaryotic translation initiation factor 4E nuclear import factor 1 | EIF4ENIF1 | Q9NRA8 | 4 |
| SR-related CTD-associated factor 1 | SCAF1 | Q9NR59 | 4 |
| DEAD (Asp-Glu-Ala-Asp) box polypeptide 21 | DDX21 | Q9NR30 | 4 |
| peter pan homolog (Drosophila) | PPAN | Q9NQ55 | 4 |
| platelet/endothelial cell adhesion molecule | PECAM1 | Q9NPB7 | 4 |
| STIP1 homology and U-box containing protein 1 | STUB1 | Q9HBT1 | 4 |
| pumilio homolog 2 (Drosophila) | PUM2 | Q9HAN2 | 4 |
| RAB24, member RAS oncogene family | RAB24 | Q9HAG2 | 4 |
| ring finger protein 20 | RNF20 | Q9H9Y7 | 3 |
| myelin expression factor 2 | MYEF2 | Q9H922 | 3 |
| tetratricopeptide repeat domain 17 | TTC17 | Q9H906 | 3 |
| arginine/serine-rich coiled-coil 2 | RSRC2 | Q9H864 | 3 |
| coiled-coil domain containing 86 | CCDC86 | Q9H6F5 | 3 |
| retinoblastoma binding protein 6 | RBBP6 | Q9H5M5 | 3 |
| NFKB activating protein-like | NKAPL | Q9H4Q7 | 3 |
| pericentriolar material 1 | PCM1 | Q9H4A2 | 3 |
| DnaJ (Hsp40) homolog, subfamily C, member 5 | DNAJC5 | Q9H3Z5 | 3 |
| SAM domain and HD domain 1 | SAMHD1 | Q9H3U9 | 3 |
| hematological and neurological expressed 1 | HN1 | Q9H3K0 | 3 |
| chromosome 3 open reading frame 63 | C3orf63 | Q9H2F7 | 3 |
| UPF3 regulator of nonsense transcripts homolog B (yeast) | UPF3B | Q9H1J0 | 3 |
| nuclear casein kinase and cyclin-dependent kinase substrate 1 | NUCKS1 | Q9H1E3 | 3 |
| phosphoglucomutase 1 | PGM1 | Q9H1D2 | 3 |
| FIP1 like 1 (S. cerevisiae) | FIP1L1 | Q9H077 | 3 |
| zinc finger protein 518B | ZNF518B | Q9C0D4 | 3 |
| zinc finger homeobox 2 | ZFHX2 | Q9C0A1 | 3 |
| dedicator of cytokinesis 7 | DOCK7 | Q9C092 | 3 |
| SERPINE1 mRNA binding protein 1 | SERBP1 | Q9BUM4 | 3 |
| transgelin 2 | TAGLN2 | Q9BUH5 | 3 |
| chromosome 5 open reading frame 45 | C5orf45 | Q9BUC1 | 3 |
| chromosome 11 open reading frame 84 | C11orf84 | Q9BUA3 | 3 |
| single stranded DNA binding protein 3; hypothetical LOC100131851 | SSBP3 | Q9BTM0 | 3 |
| neural proliferation, differentiation and control, 1 | NPDC1 | Q9BTD6 | 3 |
| villin-like | VILL | Q9BT80 | 3 |
| potassium voltage-gated channel, subfamily H (eag-related), member 2 | KCNH2 | Q9BT72 | 3 |
| metastasis associated 1 | MTA1 | Q9BRL8 | 3 |
| phospholipase C, beta 4 | PLCB4 | Q9BQW8 | 3 |
| microtubule-associated protein 2 | MAP2 | Q99976 | 3 |
| A kinase (PRKA) anchor protein 12 | AKAP12 | Q99970 | 3 |
| myosin, heavy chain 9, non-muscle | MYH9 | Q99529 | 3 |
| peroxisomal biogenesis factor 6 | PEX6 | Q99476 | 3 |
| AT rich interactive domain 1A (SWI-like) | ARID1A | Q96T89 | 3 |
| remodeling and spacing factor 1 | RSF1 | Q96T23 | 3 |
| protein tyrosine phosphatase-like A domain containing 1 | PTPLAD1 | Q96T12 | 2 |
| family with sequence similarity 40, member A | FAM40A | Q96SN2 | 2 |
| CTF18, chromosome transmission fidelity factor 18 homolog (S. cerevisiae) | CHTF18 | Q96S08 | 2 |
| MAP/microtubule affinity-regulating kinase 3 | MARK3 | Q96RG1 | 2 |
| G protein regulated inducer of neurite outgrowth 1 | GPRIN1 | Q96PZ4 | 2 |
| A kinase (PRKA) anchor protein 13 | AKAP13 | Q96P79 | 2 |
| G protein-coupled receptor 82 | GPR82 | Q96P67 | 2 |
| chromosome 21 open reading frame 59 | C21orf59 | Q96NJ2 | 2 |
| Leo1, Paf1/RNA polymerase II complex component, homolog (S. cerevisiae) | LEO1 | Q96N99 | 2 |
| chromosome 14 open reading frame 81 | C14orf81 | Q96MM1 | 2 |
| melanoma antigen family B, 18 | MAGEB18 | Q96M61 | 2 |
| zinc finger protein 828 | ZNF828 | Q96JM3 | 2 |
| vesicle-associated membrane protein 4 | VAMP4 | Q96J20 | 2 |
| microtubule-actin crosslinking factor 1 | MACF1 | Q96IQ1 | 2 |
| U2 small nuclear RNA auxiliary factor 2 | U2AF2 | Q96HC5 | 2 |
| SWI/SNF related, matrix associated, actin dependent regulator of chromatin, subfamily c, member 2 | SMARCC2 | Q96GY4 | 2 |
| CDC42 effector protein (Rho GTPase binding) 4 | CDC42EP4 | Q96FT3 | 2 |
| zinc finger with KRAB and SCAN domains 1 | ZKSCAN1 | Q96FA2 | 2 |
| cytoplasmic linker associated protein 2 | CLASP2 | Q96F87 | 2 |
| RecQ protein-like 4 | RECQL4 | Q96F55 | 2 |
| cofactor of BRCA1 | COBRA1 | Q96EW5 | 2 |
| nuclear transcription factor, X-box binding 1 | NFX1 | Q96EL5 | 2 |
| chromosome 3 open reading frame 54 | C3orf54 | Q96EL1 | 2 |
| coiled-coil domain containing 126 | CCDC126 | Q96EE4 | 2 |
| neuroepithelial cell transforming 1 | NET1 | Q96D82 | 2 |
| fibronectin type III domain containing 3B | FNDC3B | Q96D78 | 2 |
| septin 2 | SEPT2 | Q96CB0 | 2 |
| scribbled homolog (Drosophila) | SCRIB | Q96C69 | 2 |
| AKT1 substrate 1 (proline-rich) | AKT1S1 | Q96B36 | 2 |
| minichromosome maintenance complex component 2 | MCM2 | Q969W7 | 2 |
| TRK-fused gene | TFG | Q92734 | 2 |
| bromodomain containing 3 | BRD3 | Q92645 | 2 |
| transmembrane 9 superfamily protein member 4 | TM9SF4 | Q92544 | 2 |
| twist homolog 1 (Drosophila) | TWIST1 | Q92487 | 2 |
| damage-specific DNA binding protein 2, 48kDa | DDB2 | Q92466 | 2 |
| pleckstrin homology domain containing, family O member 2 | PLEKHO2 | Q8WYS8 | 2 |
| RNA binding motif protein 23 | RBM23 | Q8WY40 | 2 |
| spectrin repeat containing, nuclear envelope 2 | SYNE2 | Q8WWW3 | 2 |
| sodium channel, voltage-gated, type IX, alpha subunit | SCN9A | Q8WWN4 | 2 |
| kinesin family member 23 | KIF23 | Q8WVP0 | 2 |
| peptidylprolyl isomerase (cyclophilin)-like 4 | PPIL4 | Q8WUA2 | 2 |
| zinc finger protein 831 | ZNF831 | Q8TCP0 | 2 |
| chromosome 1 open reading frame 144 | C1orf144 | Q8TBV3 | 2 |
| glutamate receptor, metabotropic 3 | GRM3 | Q8TBH9 | 2 |
| prospero homeobox 1 | PROX1 | Q8TB91 | 2 |
| WD repeat domain 43 | WDR43 | Q8TB67 | 2 |
| tuberous sclerosis 2 | TSC2 | Q8TAZ1 | 2 |
| F-box protein 34 | FBXO34 | Q8NFA9 | 2 |
| cation channel, sperm associated 1 | CATSPER1 | Q8NEC5 | 2 |
| nucleolin | NCL | Q8NB06 | 2 |
| selenophosphate synthetase 2 | SEPHS2 | Q8NAW0 | 2 |
| dihydropyrimidinase-like 2 | DPYSL2 | Q8NAN9 | 2 |
| similar to RNA binding motif protein, X-linked; similar to hCG2011544; RNA binding motif protein, X-linked | RBMX | Q8N8Y7 | 2 |
| RNA binding motif protein 44 | RBM44 | Q8N7S3 | 2 |
| ribonucleotide reductase M2 polypeptide | RRM2 | Q8N6S3 | 2 |
| hypothetical MGC50722 | MGC50722 | Q8N5P7 | 2 |
| cytoplasmic linker associated protein 1 | CLASP1 | Q8N5B8 | 2 |
| collagen, type VI, alpha 3 | COL6A3 | Q8N4Z1 | 2 |
| DEAH (Asp-Glu-Ala-Asp/His) box polypeptide 57 | DHX57 | Q8N4U2 | 2 |
| chromosome 19 open reading frame 28 | C19orf28 | Q8N459 | 2 |
| ligand dependent nuclear receptor corepressor-like | LCORL | Q8N3X6 | 2 |
| microtubule-associated protein 1S | MAP1S | Q8N3W5 | 2 |
| heterogeneous nuclear ribonucleoprotein U-like 2 | HNRNPUL2 | Q8N3B3 | 2 |
| SEC16 homolog A (S. cerevisiae) | SEC16A | Q8N347 | 2 |
| chromosome 6 open reading frame 223 | C6orf223 | Q8N319 | 2 |
| prominin 2 | PROM2 | Q8N271 | 2 |
| cysteine-rich PAK1 inhibitor | CRIPAK | Q8N1N5 | 2 |
| zinc finger protein 174 | ZNF174 | Q8IZN5 | 2 |
| G protein-coupled receptor 116 | GPR116 | Q8IZF2 | 2 |
| zinc finger protein 683 | ZNF683 | Q8IZ20 | 2 |
| DEAD (Asp-Glu-Ala-Asp) box polypeptide 3, Y-linked | DDX3Y | Q8IYV7 | 2 |
| unc-51-like kinase 2 (C. elegans) | ULK2 | Q8IYT8 | 2 |
| membrane protein, palmitoylated 7 (MAGUK p55 subfamily member 7) | MPP7 | Q8IY28 | 2 |
| chromodomain helicase DNA binding protein 4 | CHD4 | Q8IXZ5 | 2 |
| formin-like 3 | FMNL3 | Q8IVF7 | 2 |
| poly (ADP-ribose) polymerase 1 | PARP1 | Q8IUZ9 | 2 |
| acyl-CoA synthetase long-chain family member 3 | ACSL3 | Q8IUM9 | 2 |
| phosphodiesterase 8B | PDE8B | Q8IUJ9 | 2 |
| Hermansky-Pudlak syndrome 6 | HPS6 | Q86YV9 | 2 |
| DEAD (Asp-Glu-Ala-Asp) box polypeptide 54 | DDX54 | Q86YT8 | 2 |
| chondroitin sulfate synthase 3 | CHSY3 | Q86Y52 | 2 |
| microtubule-associated protein 4 | MAP4 | Q86Y04 | 2 |
| sperm associated antigen 9 | SPAG9 | Q86WC7 | 2 |
| p21 protein (Cdc42/Rac)-activated kinase 1 | PAK1 | Q86W79 | 2 |
| chromosome 16 open reading frame 62 | C16orf62 | Q86W66 | 2 |
| TBC1 domain family, member 10B | TBC1D10B | Q86VC5 | 2 |
| target of myb1-like 2 (chicken) | TOM1L2 | Q86V61 | 2 |
| bromodomain adjacent to zinc finger domain, 1B | BAZ1B | Q86UJ6 | 2 |
| taxilin alpha | TXLNA | Q86T86 | 2 |
| chromodomain helicase DNA binding protein 7 | CHD7 | Q7Z7Q2 | 2 |
| hypothetical protein LOC387763 | AG2 | Q7Z7L8 | 2 |
| alveolar soft part sarcoma chromosome region, candidate 1 | ASPSCR1 | Q7Z6N7 | 2 |
| spectrin, alpha, non-erythrocytic 1 (alpha-fodrin) | SPTAN1 | Q7Z6M5 | 2 |
| kinesin family member 21A | KIF21A | Q7Z668 | 2 |
| immunoglobulin-like domain containing receptor 1 | ILDR1 | Q7Z578 | 2 |
| mucin 1, cell surface associated | MUC1 | Q7Z540 | 2 |
| NADH dehydrogenase (ubiquinone) 1 alpha subcomplex, 10, 42kDa | NDUFA10 | Q7Z518 | 2 |
| eukaryotic translation initiation factor 2A, 65kDa | EIF2A | Q7Z4E9 | 2 |
| tumor protein p53 binding protein 1 | TP53BP1 | Q7Z3U4 | 2 |
| zinc finger protein 185 (LIM domain) | ZNF185 | Q7Z3Q8 | 2 |
| titin | TTN | Q7Z2X3 | 2 |
| alpha thalassemia/mental retardation syndrome X-linked (RAD54 homolog, S. cerevisiae) | ATRX | Q7Z2J1 | 2 |
| achaete-scute complex homolog 5 (Drosophila) | ASCL5 | Q7RTU5 | 2 |
| similar to U5 snRNP-specific protein, 200 kDa; small nuclear ribonucleoprotein 200kDa (U5) | SNRNP200 | Q7L5W4 | 2 |
| Y box binding protein 1 | YBX1 | Q7KZ24 | 2 |
| pinin, desmosome associated protein | PNN | Q7KYL1 | 2 |
| cortactin | CTTN | Q76MU0 | 2 |
| LSM14A, SCD6 homolog A (S. cerevisiae) | LSM14A | Q76LX7 | 2 |
| growth differentiation factor 7 | gdf7 | Q75RY1 | 2 |
| topoisomerase (DNA) II alpha 170kDa | TOP2A | Q71UQ5 | 2 |
| synaptopodin | SYNPO | Q71HJ6 | 2 |
| NMDA receptor regulated 2 | NARG2 | Q71H65 | 2 |
| ankyrin 3, node of Ranvier (ankyrin G) | ANK3 | Q6ZT73 | 2 |
| leucine rich repeat containing 9 | LRRC9 | Q6ZRR7 | 2 |
| mitogen-activated protein kinase kinase 2 pseudogene; mitogen-activated protein kinase kinase 2 | MAP2K2 | Q6ZPC5 | 2 |
| GRB10 interacting GYF protein 2 | GIGYF2 | Q6Y7W6 | 2 |
| dachsous 2 (Drosophila) | DCHS2 | Q6V1P8 | 2 |
| transmembrane protein 119 | TMEM119 | Q6UXE5 | 2 |
| estrogen receptor binding site associated, antigen, 9 | EBAG9 | Q6R3F1 | 2 |
| splicing factor, arginine/serine-rich 11 | SFRS11 | Q6PJY9 | 2 |
| KH domain containing, RNA binding, signal transduction associated 1 | KHDRBS1 | Q6PJX7 | 2 |
| RAB4B, member RAS oncogene family | RAB4B | Q6PIK3 | 2 |
| triadin | TRDN | Q6PG49 | 2 |
| chromosome 20 open reading frame 132 | C20orf132 | Q6PF12 | 2 |
| KIAA1370 | KIAA1370 | Q6PDA3 | 2 |
| Ctr9, Paf1/RNA polymerase II complex component, homolog (S. cerevisiae) | CTR9 | Q6PD62 | 2 |
| mucolipin 3 | MCOLN3 | Q6P9H1 | 2 |
| Rho GTPase activating protein 18 | ARHGAP18 | Q6P679 | 2 |
| ATP-binding cassette, sub-family A (ABC1), member 9 | ABCA9 | Q6P655 | 2 |
| NIMA (never in mitosis gene a)-related kinase 5 | NEK5 | Q6P3R8 | 2 |
| enhancer of mRNA decapping 4 | EDC4 | Q6P2E9 | 2 |
| thyroid hormone receptor associated protein 3 | THRAP3 | Q6P0P7 | 2 |
| MARCKS-like 1 | MARCKSL1 | Q6NXS5 | 2 |
| histone cluster 1, H3j | HIST2H3A | Q6NWP9 | 2 |
| myristoylated alanine-rich protein kinase C substrate | MARCKS | Q6NVI1 | 2 |
| LIM and calponin homology domains 1 | LIMCH1 | Q6N054 | 2 |
| keratin associated protein 5-4 | KRTAP5-4 | Q6L8H1 | 2 |
| RAB12, member RAS oncogene family | RAB12 | Q6IQ22 | 2 |
| centrosomal protein 250kDa | CEP250 | Q6IPL3 | 2 |
| KIAA1656 protein | KIAA1656 | Q6ICM1 | 2 |
| heterogeneous nuclear ribonucleoprotein K; similar to heterogeneous nuclear ribonucleoprotein K | HNRNPK | Q6IBN1 | 2 |
| eukaryotic translation initiation factor 3, subunit G | EIF3G | Q6IAM0 | 2 |
| potassium intermediate/small conductance calcium-activated channel, subfamily N, member 1 | KCNN1 | Q6DJU4 | 2 |
| similar to Bcl-2-associated transcription factor 1 (Btf); BCL2-associated transcription factor 1 | BCLAF1 | Q6DCA8 | 2 |
| heterogeneous nuclear ribonucleoprotein H1 (H) | HNRNPH1 | Q68DG4 | 2 |
| exportin 1 (CRM1 homolog, yeast) | XPO1 | Q63HP8 | 2 |
| insulin-like growth factor 2 mRNA binding protein 3 | IGF2BP3 | Q63HM0 | 2 |
| rhomboid 5 homolog 2 (Drosophila) | RHBDF2 | Q5YGQ8 | 2 |
| zinc finger, MYM-type 4 | ZMYM4 | Q5VZL5 | 2 |
| tight junction protein 2 (zona occludens 2) | TJP2 | Q5VXL0 | 2 |
| nebulin-related anchoring protein | NRAP | Q5VWI4 | 2 |
| antigen identified by monoclonal antibody Ki-67 | MKI67 | Q5VWH2 | 2 |
| human immunodeficiency virus type I enhancer binding protein 1 | HIVEP1 | Q5VW60 | 2 |
| serine/arginine repetitive matrix 1 | SRRM1 | Q5VVN4 | 2 |
| wings apart-like homolog (Drosophila) | WAPAL | Q5VSK5 | 2 |
| GTPase activating protein (SH3 domain) binding protein 1 | G3BP1 | Q5U0Q1 | 2 |
| myeloid leukemia factor 2 | MLF2 | Q5U0N1 | 2 |
| nucleolar protein 8 | NOL8 | Q5TCD7 | 2 |
| bystin-like | BYSL | Q5T8J2 | 2 |
| progestagen-associated endometrial protein | PAEP | Q5T6T1 | 2 |
| calmodulin regulated spectrin-associated protein 1 | CAMSAP1 | Q5T5Y3 | 2 |
| HORMA domain containing 1 | HORMAD1 | Q5T5I4 | 2 |
| hepatoma-derived growth factor (high-mobility group protein 1-like) | HDGF | Q5SZ07 | 2 |
| TAF5 RNA polymerase II, TATA box binding protein (TBP)-associated factor, 100kDa | TAF5 | Q5SYD5 | 2 |
| death-domain associated protein | DAXX | Q5STR5 | 2 |
| chromosome 1 open reading frame 173 | C1orf173 | Q5RHP9 | 2 |
| bromodomain and WD repeat domain containing 1 | BRWD1 | Q5R2V1 | 2 |
| forkhead-associated (FHA) phosphopeptide binding domain 1 | FHAD1 | Q5JYW1 | 2 |
| chromosome 22 open reading frame 23 | C22orf23 | Q5JYU9 | 2 |
| karyopherin alpha 3 (importin alpha 4) | KPNA3 | Q5JVN1 | 2 |
| centrosomal protein 110kDa | CEP110 | Q5JVD6 | 2 |
| FERM, RhoGEF (ARHGEF) and pleckstrin domain protein 1 (chondrocyte-derived) | FARP1 | Q5JV94 | 2 |
| mitogen-activated protein kinase kinase kinase 15 | MAP3K15 | Q5JPR4 | 2 |
| ubiquitin specific peptidase 45 | USP45 | Q5JPK1 | 2 |
| lamin A/C | LMNA | Q5I6Y6 | 2 |
| zinc finger CCCH-type containing 12B | ZC3H12B | Q5HYM0 | 2 |
| family with sequence similarity 76, member B | FAM76B | Q5HYJ3 | 2 |
| zinc finger protein 652 | ZNF652 | Q5H9Q0 | 2 |
| sorbin and SH3 domain containing 3 | SORBS3 | Q5BJE4 | 2 |
| topoisomerase (DNA) II beta 180kDa | TOP2B | Q59H80 | 2 |
| drebrin-like | DBNL | Q59FH4 | 2 |
| insulin-like growth factor 2 receptor | IGF2R | Q59EZ3 | 2 |
| heat shock protein 90kDa alpha (cytosolic), class B member 2 (pseudogene) | HSP90AB2P | Q58FF8 | 2 |
| transmembrane protein 131 | TMEM131 | Q584P2 | 2 |
| anaplastic lymphoma receptor tyrosine kinase | ALK | Q580I3 | 2 |
| CUB and Sushi multiple domains 2 | CSMD2 | Q53TY4 | 2 |
| thyroid hormone receptor interactor 12 | TRIP12 | Q53TE7 | 2 |
| erythrocyte membrane protein band 4.1 like 5 | EPB41L5 | Q53T34 | 2 |
| activating transcription factor 2 | ATF2 | Q53RY2 | 2 |
| AP2 associated kinase 1 | AAK1 | Q53RX6 | 2 |
| abl interactor 2 | ABI2 | Q53RS4 | 2 |
| spectrin, beta, non-erythrocytic 1 | SPTBN1 | Q53R99 | 2 |
| Kruppel-like factor 11 | KLF11 | Q53QU8 | 2 |
| general transcription factor IIIC, polypeptide 2, beta 110kDa | GTF3C2 | Q53QN0 | 2 |
| Rho GTPase activating protein 25 | ARHGAP25 | Q53QF7 | 2 |
| RNA binding protein, autoantigenic (hnRNP-associated with lethal yellow homolog (mouse)) | RALY | Q53GL6 | 2 |
| solute carrier family 35, member C2 | SLC35C2 | Q53GK3 | 2 |
| tyrosyl-tRNA synthetase | YARS | Q53EN1 | 2 |
| atonal homolog 8 (Drosophila) | ATOH8 | Q504S2 | 2 |
| R3H domain containing 1 | R3HDM1 | Q4ZG59 | 2 |
| kinesin family member 5A | KIF5A | Q4LE26 | 2 |
| suppressor of defective silencing 3 homolog (S. cerevisiae) | SUDS3 | Q4KMQ5 | 2 |
| chromosome 5 open reading frame 42 | C5orf42 | Q4G174 | 2 |
| DiGeorge syndrome critical region gene 14 | DGCR14 | Q49AH7 | 2 |
| heterogeneous nuclear ribonucleoprotein A1-like 3 | HNRPA1L3 | Q3MI39 | 2 |
| reticulon 4 | RTN4 | Q3LIF4 | 2 |
| ubiquitin specific peptidase 42 | USP42 | Q3C166 | 2 |
| HECT, UBA and WWE domain containing 1 | HUWE1 | Q3B7K0 | 2 |
| splicing factor 3b, subunit 1, 155kDa | SF3B1 | Q32Q20 | 2 |
| calcium regulated heat stable protein 1, 24kDa | CARHSP1 | Q2YDX5 | 2 |
| nestin | NES | Q2YDX4 | 2 |
| receptor-interacting serine-threonine kinase 2 | RIPK2 | Q2TU65 | 2 |
| inhibitor of Bruton agammaglobulinemia tyrosine kinase | IBTK | Q2QKU3 | 2 |
| KIAA0355 | KIAA0355 | Q2M3W4 | 2 |
| similar to Rho-associated, coiled-coil containing protein kinase 1; Rho-associated, coiled-coil containing protein kinase 1 | ROCK1 | Q2KHM4 | 2 |
| protein inhibitor of activated STAT, 1 | PIAS1 | Q1XBU8 | 2 |
| integrin, beta 3 (platelet glycoprotein IIIa, antigen CD61) | ITGB3 | Q1PBM2 | 2 |
| KIAA0528 | KIAA0528 | Q17RY7 | 2 |
| acetyl-Coenzyme A carboxylase beta | ACACB | Q16852 | 2 |
| glyceraldehyde-3-phosphate dehydrogenase-like 6 | GAPDH | Q16768 | 2 |
| ELAV (embryonic lethal, abnormal vision, Drosophila)-like 4 (Hu antigen D) | ELAVL4 | Q16234 | 2 |
| adducin 1 (alpha) | ADD1 | Q16156 | 2 |
| protein tyrosine phosphatase, non-receptor type 12 | PTPN12 | Q16128 | 2 |
| small nuclear ribonucleoprotein 70kDa (U1) | SNRNP70 | Q15687 | 2 |
| telomeric repeat binding factor 2 | TERF2 | Q15554 | 2 |
| Sec23 homolog A (S. cerevisiae) | SEC23A | Q15436 | 2 |
| prostaglandin E synthase 3 (cytosolic) | PTGES3 | Q15185 | 2 |
| poly(rC) binding protein 1 | PCBP1 | Q14975 | 2 |
| Notch homolog 3 (Drosophila) | NOTCH3 | Q14962 | 2 |
| phosphoprotein enriched in astrocytes 15 | PEA15 | Q14801 | 2 |
| tenascin C | TNC | Q14583 | 2 |
| similar to RNA binding motif protein 39; RNA binding motif protein 39 | RBM39 | Q14498 | 2 |
| heterogeneous nuclear ribonucleoprotein D (AU-rich element RNA binding protein 1, 37kDa) | HNRNPD | Q14100 | 2 |
| c-abl oncogene 1, receptor tyrosine kinase | ABL1 | Q13688 | 2 |
| chromatin assembly factor 1, subunit B (p60) | CHAF1B | Q13112 | 2 |
| RAN binding protein 2 | RANBP2 | Q13073 | 2 |
| nucleoporin 160kDa | NUP160 | Q12769 | 2 |
| coiled-coil domain containing 88C | CCDC88C | Q0P665 | 2 |
| similar to acetyl-Coenzyme A acyltransferase 2 (mitochondrial 3-oxoacyl-Coenzyme A thiolase) | MYO5B | Q0P656 | 2 |
| PCF11, cleavage and polyadenylation factor subunit, homolog (S. cerevisiae) | PCF11 | Q0D2H7 | 2 |
| calmodulin regulated spectrin-associated protein 1-like 1 | CAMSAP1L1 | Q08AD1 | 2 |
| KIAA1704 | KIAA1704 | Q05D87 | 2 |
| early endosome antigen 1 | EEA1 | Q05D76 | 2 |
| NFKB activating protein | NKAP | Q05D22 | 2 |
| PC4 and SFRS1 interacting protein 1 | PSIP1 | Q05CM9 | 2 |
| glutamyl-prolyl-tRNA synthetase | EPRS | Q05BP6 | 2 |
| armadillo repeat gene deletes in velocardiofacial syndrome | ARVCF | Q05BN6 | 2 |
| zinc finger protein 91 | ZNF91 | Q05481 | 2 |
| similar to Acidic leucine-rich nuclear phosphoprotein 32 family member B (PHAPI2 protein) | ANP32B | P78459 | 2 |
| tubulin, alpha 4a | TUBA4A | P68366 | 2 |
| TPI1 pseudogene; triosephosphate isomerase 1 | TPI1 | P60174 | 2 |
| chromosome 21 open reading frame 82 | C21orf82 | P59036 | 2 |
| IQ motif containing GTPase activating protein 1 | IQGAP1 | P46940 | 2 |
| NOP2 nucleolar protein homolog (yeast) | NOP2 | P46087 | 2 |
| transcription elongation factor A (SII), 1 pseudogene 2; transcription elongation factor A (SII), 1 | TCEA1 | P23193 | 2 |
| neural cell adhesion molecule 1 | NCAM1 | P13593 | 2 |
| cyclin-dependent kinase 4 | CDK4 | P11802 | 2 |
| microtubule-associated protein tau | MAPT | P10636 | 2 |
| thymopoietin | TMPO | P08919 | 2 |
| ribosomal protein S17 | RPS17 | P08708 | 2 |
| carcinoembryonic antigen-related cell adhesion molecule 5 | CEACAM5 | P06731 | 2 |
| hepsin | HPN | P05981 | 2 |
| eukaryotic translation initiation factor 5B | EIF5B | O95805 | 2 |
| structural maintenance of chromosomes 4 | SMC4 | O95752 | 2 |
| EPM2A (laforin) interacting protein 1 | EPM2AIP1 | O94866 | 2 |
| kelch repeat and BTB (POZ) domain containing 11 | KBTBD11 | O94819 | 2 |
| HIRA interacting protein 3 | HIRIP3 | O75708 | 2 |
| eukaryotic translation initiation factor 4E family member 2 | EIF4E2 | O75349 | 2 |
| gamma-glutamyl cyclotransferase | GGCT | O75223 | 2 |
| ATPase, Ca++ transporting, type 2C, member 2 | ATP2C2 | O75185 | 2 |
| phosphatase and actin regulator 2 | PHACTR2 | O75167 | 2 |
| apoptotic chromatin condensation inducer 1 | ACIN1 | O75158 | 2 |
| dyskeratosis congenita 1, dyskerin | DKC1 | O60832 | 2 |
| eukaryotic translation initiation factor 4 gamma, 3 | EIF4G3 | O43432 | 2 |
| heat shock 70kDa protein 12A | HSPA12A | O43301 | 2 |
| API5-like 1; apoptosis inhibitor 5 | API5 | O15441 | 2 |
| RER1 retention in endoplasmic reticulum 1 homolog (S. cerevisiae) | RER1 | O15258 | 2 |
| NAC alpha domain containing | NACAD | O15069 | 2 |
| glycogen synthase kinase 3 alpha | GSK3A | O14959 | 2 |
| general transcription factor II, i; general transcription factor II, i, pseudogene | GTF2I | O14743 | 2 |
| TRAF-type zinc finger domain containing 1 | TRAFD1 | O14545 | 2 |
| neuregulin 2 | NRG2 | O14511 | 2 |
| chloride intracellular channel 1 | CLIC1 | O00299 | 2 |
| suppressor of Ty 5 homolog (S. cerevisiae) | SUPT5H | O00267 | 2 |
| chromosome 17 open reading frame 49 | C17orf49 | C9J4G0 | 2 |
| potassium channel, subfamily T, member 1 | KCNT1 | B7ZVY4 | 2 |
| carbamoyl-phosphate synthetase 1, mitochondrial | CPS1 | B7Z818 | 2 |
| TBC1 domain family, member 15 | TBC1D15 | B4DMT9 | 2 |
| otoferlin | OTOF | B4DJX0 | 2 |
| protein kinase N1 | PKN1 | B3KVN3 | 2 |
| transducin-like enhancer of split 3 (E(sp1) homolog, Drosophila) | TLE3 | B3KUA2 | 2 |
| kinesin light chain 4 | KLC4 | B3KSQ3 | 2 |
| family with sequence similarity 110, member B | FAM110B | B3KRT5 | 2 |
| synaptotagmin-like 2 | SYTL2 | B3KRS3 | 2 |
| formin binding protein 4 | FNBP4 | B3KNP0 | 2 |
| odz, odd Oz/ten-m homolog 1(Drosophila) | ODZ1 | B2RTR5 | 2 |
| NOL1/NOP2/Sun domain family, member 2 | NSUN2 | B2RNR4 | 2 |
| RNA binding motif protein 25 | RBM25 | B2RNA8 | 2 |
| regulator of G-protein signaling 18 | RGS18 | B2RD23 | 2 |
| CD209 molecule | CD209 | B2R907 | 2 |
| serine/threonine kinase 10 | STK10 | B2R8F5 | 2 |
| ribosomal protein S3 pseudogene 3; ribosomal protein S3 | RPS3 | B2R7N5 | 2 |
| solute carrier family 5 (sodium/glucose cotransporter), member 1 | SLC5A1 | B2R7E2 | 2 |
| histamine receptor H4 | HRH4 | B2KJ48 | 2 |
| interferon regulatory factor 2 binding protein 2 | IRF2BP2 | B1AM36 | 2 |
| family with sequence similarity 178, member A | FAM178A | B1AL17 | 2 |
| SWI/SNF related, matrix associated, actin dependent regulator of chromatin, subfamily a, member 4 | SMARCA4 | B1A8Z5 | 2 |
| pleckstrin homology-like domain, family B, member 1 | PHLDB1 | B0YJ63 | 2 |
| doublecortin | DCX | A9Z1V8 | 2 |
| GTPase activating protein and VPS9 domains 1 | GAPVD1 | A8MYK3 | 2 |
| epsin 3 | EPN3 | A8KAB2 | 2 |
| RNA binding motif protein, X-linked 2 | RBMX2 | A8K9Z0 | 2 |
| D4, zinc and double PHD fingers family 2 | DPF2 | A8K7C9 | 2 |
| potassium channel tetramerisation domain containing 15 | KCTD15 | A8K600 | 2 |
| leucine rich repeat containing 41 | LRRC41 | A8K5G8 | 2 |
| paired-like homeobox 2a | PHOX2A | A8K3N0 | 2 |
| ataxin 2-like | ATXN2L | A8K1R6 | 2 |
| glucocorticoid receptor DNA binding factor 1 | GRLF1 | A7E2A4 | 2 |
| SH3-domain GRB2-like (endophilin) interacting protein 1 | SGIP1 | A6NL81 | 2 |
| family with sequence similarity 65, member B | FAM65B | A6NHP2 | 2 |
| family with sequence similarity 186, member A | FAM186A | A6NE01 | 2 |
| family with sequence similarity 54, member B | FAM54B | A6NCB4 | 2 |
| chromosome 7 open reading frame 47 | C7orf47 | A4D2C5 | 2 |
| glucocorticoid induced transcript 1 | GLCCI1 | A4D103 | 2 |
| protein phosphatase 2A activator, regulatory subunit 4 | PPP2R4 | A2A347 | 2 |
| stathmin 1 | STMN1 | A2A2D1 | 2 |
| cysteine-rich protein 2 | CRIP2 | A1A4U1 | 2 |
| U2-associated SR140 protein | SR140 | A0PJ60 | 2 |
| v-yes-1 Yamaguchi sarcoma viral related oncogene homolog | LYN | A0AVQ5 | 2 |
