# Supplementary material for: VENNTURE–A Novel Venn Diagram Investigational Tool for Multiple Pharmacological Dataset Analysis
Source: PLoS One. 2012 May 14;7(5):e36911. doi: 10.1371/journal.pone.0036911 (PMC3351456; doi:10.1371/journal.pone.0036911)
Supplement: Table S8 — Phosphoproteins extracted from 10 nM MeCh-stimulated chronic minimal peroxide (CMP)-state human neuroblastoma SH-SY5Y cells. For each successfully identified protein official symbol, Uniprot accession code and number of peptides recovered are indicated. (DOC) [file pone.0036911.s009.doc]

**Table S8.** Phosphoproteins extracted from 10nM MeCh-stimulated chronic minimal peroxide (CMP)-state human neuroblastoma SH-SY5Y cells. For each successfully identified protein official symbol, Uniprot accession code and number of peptides recovered are indicated.

| **Protein Identification** | **Symbol** | **Accession** | **Peptide** |
| --- | --- | --- | --- |
| cysteine-rich protein 2 | CRIP2 | A1A4U1 | 10 |
| stathmin 1 | STMN1 | A2A2D1 | 10 |
| family with sequence similarity 54, member B | FAM54B | A6NCB4 | 9 |
| leucine rich repeat containing 41 | LRRC41 | A8K5G8 | 8 |
| potassium channel tetramerisation domain containing 15 | KCTD15 | A8K600 | 7 |
| epsin 3 | EPN3 | A8K6J3 | 7 |
| D4, zinc and double PHD fingers family 2 | DPF2 | A8K7C9 | 6 |
| neural cell adhesion molecule 1 | NCAM1 | A8K8T8 | 6 |
| doublecortin | DCX | A9Z1V8 | 6 |
| HLA-B associated transcript 3 | BAT3 | B0UX84 | 5 |
| interferon regulatory factor 2 binding protein 2 | IRF2BP2 | B1AM36 | 5 |
| lamin B receptor | LBR | B2R5P3 | 5 |
| DEAD (Asp-Glu-Ala-Asp) box polypeptide 23 | DDX23 | B2R600 | 5 |
| ribosomal protein S3 pseudogene 3; ribosomal protein S3 | RPS3 | B2R7N5 | 4 |
| SPARC related modular calcium binding 1 | SMOC1 | B2R7P5 | 4 |
| RNA binding motif protein 25 | RBM25 | B2RNA8 | 4 |
| NOL1/NOP2/Sun domain family, member 2 | NSUN2 | B2RNR4 | 4 |
| transmembrane protein 44 | TMEM44 | B7ZLZ5 | 4 |
| suppressor of Ty 5 homolog (S. cerevisiae) | SUPT5H | O00267 | 4 |
| TRAF-type zinc finger domain containing 1 | TRAFD1 | O14545 | 4 |
| paired-like homeobox 2a | PHOX2A | O14813 | 3 |
| glycogen synthase kinase 3 alpha | GSK3A | O14959 | 3 |
| eukaryotic translation initiation factor 4 gamma, 3 | EIF4G3 | O43432 | 3 |
| dyskeratosis congenita 1, dyskerin | DKC1 | O60832 | 3 |
| apoptotic chromatin condensation inducer 1 | ACIN1 | O75158 | 3 |
| tripartite motif-containing 3 | TRIM3 | O75382 | 3 |
| melanoma antigen family D, 2 | MAGED2 | O76058 | 3 |
| kelch repeat and BTB (POZ) domain containing 11 | KBTBD11 | O94819 | 3 |
| EPM2A (laforin) interacting protein 1 | EPM2AIP1 | O94866 | 3 |
| structural maintenance of chromosomes 4 | SMC4 | O95752 | 3 |
| eukaryotic translation initiation factor 5B | EIF5B | O95805 | 3 |
| thymopoietin | TMPO | P08919 | 3 |
| microtubule-associated protein tau | MAPT | P10636 | 2 |
| olfactory receptor, family 4, subfamily D, member 2 | OR4D2 | P58180 | 2 |
| TPI1 pseudogene; triosephosphate isomerase 1 | TPI1 | P60174 | 2 |
| tubulin, alpha 4a | TUBA4A | P68366 | 2 |
| suppression of tumorigenicity 5 | ST5 | P78524 | 2 |
| brain abundant, membrane attached signal protein 1 | BASP1 | P80723 | 2 |
| chromobox homolog 1 (HP1 beta homolog Drosophila ) | CBX1 | P83916 | 2 |
| glutamyl-prolyl-tRNA synthetase | EPRS | Q05BP6 | 2 |
| Dmx-like 1 | DMXL1 | Q05C95 | 2 |
| NFKB activating protein | NKAP | Q05D22 | 2 |
| calmodulin regulated spectrin-associated protein 1-like 1 | CAMSAP1L1 | Q08AD1 | 2 |
| WD repeat domain 17 | WDR17 | Q0QD35 | 2 |
| Rho GTPase activating protein 21 | ARHGAP21 | Q0VF98 | 2 |
| nuclear factor of activated T-cells, cytoplasmic, calcineurin-dependent 2 | NFATC2 | Q13469 | 2 |
| c-abl oncogene 1, receptor tyrosine kinase | ABL1 | Q13688 | 2 |
| heterogeneous nuclear ribonucleoprotein D (AU-rich element RNA binding protein 1, 37kDa) | HNRNPD | Q14100 | 2 |
| similar to RNA binding motif protein 39; RNA binding motif protein 39 | RBM39 | Q14498 | 2 |
| poly(rC) binding protein 1 | PCBP1 | Q14975 | 2 |
| telomeric repeat binding factor 2 | TERF2 | Q15554 | 2 |
| adducin 1 (alpha) | ADD1 | Q16156 | 2 |
| ELAV (embryonic lethal, abnormal vision, Drosophila)-like 4 (Hu antigen D) | ELAVL4 | Q16234 | 2 |
| KIAA0528 | KIAA0528 | Q17RY7 | 2 |
| syntrophin, gamma 1 | SNTG1 | Q2M3Q0 | 2 |
| nestin | NES | Q2YDX4 | 2 |
| zinc finger protein, multitype 2 | ZFPM2 | Q32MA6 | 2 |
| protein kinase, cAMP-dependent, catalytic, alpha | PRKACA | Q32P54 | 2 |
| HECT, UBA and WWE domain containing 1 | HUWE1 | Q3B7K0 | 2 |
| ubiquitin specific peptidase 42 | USP42 | Q3C166 | 2 |
| zinc finger protein 534; zinc finger protein 528 | ZNF534 | Q3MIS6 | 2 |
| Cdon homolog (mouse) | CDON | Q4KMG0 | 2 |
| suppressor of defective silencing 3 homolog (S. cerevisiae) | SUDS3 | Q4KMQ5 | 2 |
| multiple PDZ domain protein | MPDZ | Q4LE30 | 2 |
| ferredoxin reductase | FDXR | Q4PJI0 | 2 |
| G protein-coupled receptor 52 | GPR52 | Q4VBL6 | 2 |
| yrdC domain containing (E. coli) | YRDC | Q4W4X8 | 2 |
| AP2 associated kinase 1 | AAK1 | Q4ZFZ3 | 2 |
| solute carrier family 35, member C2 | SLC35C2 | Q53GK3 | 2 |
| spectrin, beta, non-erythrocytic 1 | SPTBN1 | Q53R99 | 2 |
| activating transcription factor 2 | ATF2 | Q53RY2 | 2 |
| thyroid hormone receptor interactor 12 | TRIP12 | Q53TE7 | 2 |
| heat shock protein 90kDa alpha (cytosolic), class B member 2 (pseudogene) | HSP90AB2P | Q58FF8 | 2 |
| insulin-like growth factor 2 receptor | IGF2R | Q59EZ3 | 2 |
| drebrin-like | DBNL | Q59FH4 | 2 |
| cyclin K | CCNK | Q59FT6 | 2 |
| topoisomerase (DNA) II beta 180kDa | TOP2B | Q59H80 | 2 |
| cleavage stimulation factor, 3' pre-RNA, subunit 2, 64kDa | CSTF2 | Q5H951 | 2 |
| family with sequence similarity 76, member B | FAM76B | Q5HYJ3 | 2 |
| exportin 5 | XPO5 | Q5JTE7 | 2 |
| karyopherin alpha 3 (importin alpha 4) | KPNA3 | Q5JVN1 | 2 |
| NSFL1 (p97) cofactor (p47) | NSFL1C | Q5JXA5 | 2 |
| RNA binding motif protein, X-linked 2 | RBMX2 | Q5JY82 | 2 |
| hepatoma-derived growth factor (high-mobility group protein 1-like) | HDGF | Q5SZ07 | 2 |
| bystin-like | BYSL | Q5T8J2 | 2 |
| DNA methyltransferase 1 associated protein 1 | DMAP1 | Q5TG40 | 2 |
| GTPase activating protein (SH3 domain) binding protein 1 | G3BP1 | Q5U0Q1 | 2 |
| wings apart-like homolog (Drosophila) | WAPAL | Q5VSK5 | 2 |
| synapse defective 1, Rho GTPase, homolog 2 (C. elegans) | SYDE2 | Q5VT97 | 2 |
| serine/arginine repetitive matrix 1 | SRRM1 | Q5VVN4 | 2 |
| ribonucleotide reductase M2 polypeptide | RRM2 | Q5WRU7 | 2 |
| DnaJ (Hsp40) homolog, subfamily C, member 16 | DNAJC16 | Q68D57 | 2 |
| heterogeneous nuclear ribonucleoprotein H1 (H) | HNRNPH1 | Q68DG4 | 2 |
| eukaryotic translation initiation factor 2 alpha kinase 4 | EIF2AK4 | Q69YL7 | 2 |
| ATP-binding cassette, sub-family F (GCN20), member 1 | ABCF1 | Q69YP6 | 2 |
| similar to Bcl-2-associated transcription factor 1 (Btf); BCL2-associated transcription factor 1 | BCLAF1 | Q6DCA8 | 2 |
| eukaryotic translation initiation factor 3, subunit G | EIF3G | Q6IAM0 | 2 |
| heterogeneous nuclear ribonucleoprotein K; similar to heterogeneous nuclear ribonucleoprotein K | HNRNPK | Q6IBN1 | 2 |
| RAB12, member RAS oncogene family | RAB12 | Q6IQ22 | 2 |
| polybromo 1 | PBRM1 | Q6IRX1 | 2 |
| LIM and calponin homology domains 1 | LIMCH1 | Q6N054 | 2 |
| myristoylated alanine-rich protein kinase C substrate | MARCKS | Q6NVI1 | 2 |
| MARCKS-like 1 | MARCKSL1 | Q6NXS5 | 2 |
| thyroid hormone receptor associated protein 3 | THRAP3 | Q6P0P7 | 2 |
| PC4 and SFRS1 interacting protein 1 | PSIP1 | Q6P391 | 2 |
| TAF3 RNA polymerase II, TATA box binding protein (TBP)-associated factor, 140kDa | TAF3 | Q6P6B5 | 2 |
| sterile alpha motif domain containing 1 | SAMD1 | Q6PIS7 | 2 |
| microtubule-associated protein 1B | MAP1B | Q6PJD3 | 2 |
| splicing factor, arginine/serine-rich 11 | SFRS11 | Q6PJY9 | 2 |
| formin 1 | FMN1 | Q6ZSY1 | 2 |
| tankyrase 1 binding protein 1, 182kDa | TNKS1BP1 | Q6ZV74 | 2 |
| RNA binding motif protein 6 | RBM6 | Q6ZVV4 | 2 |
| synaptopodin | SYNPO | Q71HJ6 | 2 |
| RNA binding motif protein 33 | RBM33 | Q75ML5 | 2 |
| nucleoporin 214kDa | NUP214 | Q75R47 | 2 |
| ligase I, DNA, ATP-dependent | LIG1 | Q76GR4 | 2 |
| cortactin | CTTN | Q76MU0 | 2 |
| similar to U5 snRNP-specific protein, 200 kDa; small nuclear ribonucleoprotein 200kDa (U5) | SNRNP200 | Q7L5W4 | 2 |
| alpha thalassemia/mental retardation syndrome X-linked (RAD54 homolog, S. cerevisiae) | ATRX | Q7Z2J1 | 2 |
| titin | TTN | Q7Z2X3 | 2 |
| mitogen-activated protein kinase kinase 2 pseudogene; mitogen-activated protein kinase kinase 2 | MAP2K2 | Q7Z370 | 2 |
| methyl CpG binding protein 2 (Rett syndrome) | MECP2 | Q7Z384 | 2 |
| tumor protein p53 binding protein 1 | TP53BP1 | Q7Z3U4 | 2 |
| eukaryotic translation initiation factor 2A, 65kDa | EIF2A | Q7Z4E9 | 2 |
| immunoglobulin-like domain containing receptor 1 | ILDR1 | Q7Z578 | 2 |
| mucin 19, oligomeric | MUC19 | Q7Z5P9 | 2 |
| kinesin family member 21A | KIF21A | Q7Z668 | 2 |
| hypothetical protein LOC387763 | AG2 | Q7Z7L8 | 2 |
| taxilin alpha | TXLNA | Q86T86 | 2 |
| bromodomain adjacent to zinc finger domain, 1B | BAZ1B | Q86UJ6 | 2 |
| microtubule-associated protein 4 | MAP4 | Q86Y04 | 2 |
| DEAD (Asp-Glu-Ala-Asp) box polypeptide 54 | DDX54 | Q86YT8 | 2 |
| tau tubulin kinase 2 | TTBK2 | Q8IWY7 | 2 |
| DEAD (Asp-Glu-Ala-Asp) box polypeptide 51 | DDX51 | Q8IXK5 | 2 |
| zinc finger protein 683 | ZNF683 | Q8IZ20 | 2 |
| heterogeneous nuclear ribonucleoprotein U-like 2 | HNRNPUL2 | Q8N3B3 | 2 |
| cytoplasmic linker associated protein 1 | CLASP1 | Q8N5B8 | 2 |
| similar to RNA binding motif protein, X-linked; similar to hCG2011544 | RBMX | Q8N8Y7 | 2 |
| dihydropyrimidinase-like 2 | DPYSL2 | Q8NAN9 | 2 |
| PRP38 pre-mRNA processing factor 38 (yeast) domain containing A | PRPF38A | Q8NAV1 | 2 |
| nucleolin | NCL | Q8NB06 | 2 |
| WD repeat domain 43 | WDR43 | Q8TB67 | 2 |
| prospero homeobox 1 | PROX1 | Q8TB91 | 2 |
| cancer antigen 1 | CAGE1 | Q8TC20 | 2 |
| peptidylprolyl isomerase (cyclophilin)-like 4 | PPIL4 | Q8WUA2 | 2 |
| myosin IXB | MYO9B | Q8WVD2 | 2 |
| twist homolog 1 (Drosophila) | TWIST1 | Q92487 | 2 |
| H1 histone family, member X | H1FX | Q92522 | 2 |
| bromodomain containing 3 | BRD3 | Q92645 | 2 |
| eukaryotic translation elongation factor 1 delta (guanine nucleotide exchange protein) | EEF1D | Q969J1 | 2 |
| gap junction protein, alpha 10, 62kDa | GJA10 | Q969M2 | 2 |
| minichromosome maintenance complex component 2 | MCM2 | Q969W7 | 2 |
| scribbled homolog (Drosophila) | SCRIB | Q96C69 | 2 |
| septin 2 | SEPT2 | Q96CB0 | 2 |
| similar to chromobox homolog 3; chromobox homolog 3 (HP1 gamma homolog, Drosophila) | CBX3 | Q96CD7 | 2 |
| ligase III, DNA, ATP-dependent | LIG3 | Q96DF0 | 2 |
| zinc finger CCCH-type containing 18 | ZC3H18 | Q96DG4 | 2 |
| ribophorin II | RPN2 | Q96E21 | 2 |
| zinc finger with KRAB and SCAN domains 1 | ZKSCAN1 | Q96FA2 | 2 |
| glucocorticoid induced transcript 1 | GLCCI1 | Q96FD0 | 2 |
| CDC42 effector protein (Rho GTPase binding) 4 | CDC42EP4 | Q96FT3 | 2 |
| leucine-rich repeats and WD repeat domain containing 1 | LRWD1 | Q96GJ2 | 2 |
| SWI/SNF related, matrix associated, actin dependent regulator of chromatin, subfamily c, member 2 | SMARCC2 | Q96GY4 | 2 |
| retinoic acid induced 1 | RAI1 | Q96JK5 | 2 |
| zinc finger protein 828 | ZNF828 | Q96JM3 | 2 |
| G protein regulated inducer of neurite outgrowth 1 | GPRIN1 | Q96PZ4 | 2 |
| family with sequence similarity 40, member A | FAM40A | Q96SN2 | 2 |
| nuclear receptor binding protein 1 | NRBP1 | Q96SU3 | 2 |
| protein tyrosine phosphatase-like A domain containing 1 | PTPLAD1 | Q96T12 | 2 |
| remodeling and spacing factor 1 | RSF1 | Q96T23 | 2 |
| AT rich interactive domain 1A (SWI-like) | ARID1A | Q96T89 | 2 |
| human immunodeficiency virus type I enhancer binding protein 3 | HIVEP3 | Q99302 | 2 |
| myosin, heavy chain 9, non-muscle | MYH9 | Q99529 | 2 |
| A kinase (PRKA) anchor protein 12 | AKAP12 | Q99970 | 2 |
| microtubule-associated protein 2 | MAP2 | Q99976 | 2 |
| phospholipase C, beta 4 | PLCB4 | Q9BQW8 | 2 |
| potassium voltage-gated channel, subfamily H (eag-related), member 2 | KCNH2 | Q9BT72 | 2 |
| SERPINE1 mRNA binding protein 1 | SERBP1 | Q9BUM4 | 2 |
| phosphatidylserine synthase 2 | PTDSS2 | Q9BVG9 | 2 |
| Leber congenital amaurosis 5 | LCA5 | Q9BWX7 | 2 |
| dedicator of cytokinesis 7 | DOCK7 | Q9C092 | 2 |
| SUMO1/sentrin specific peptidase 7 | SENP7 | Q9C0F6 | 2 |
| FIP1 like 1 (S. cerevisiae) | FIP1L1 | Q9H077 | 2 |
| phosphoglucomutase 1 | PGM1 | Q9H1D2 | 2 |
| nuclear casein kinase and cyclin-dependent kinase substrate 1 | NUCKS1 | Q9H1E3 | 2 |
| G protein-coupled receptor 18 | GPR18 | Q9H2L2 | 2 |
| hematological and neurological expressed 1 | HN1 | Q9H3K0 | 2 |
| DnaJ (Hsp40) homolog, subfamily C, member 5 | DNAJC5 | Q9H3Z5 | 2 |
| tumor protein D52-like 2 | TPD52L2 | Q9H3Z6 | 2 |
| pericentriolar material 1 | PCM1 | Q9H4A2 | 2 |
| E2F transcription factor 8 | E2F8 | Q9H5M0 | 2 |
| retinoblastoma binding protein 6 | RBBP6 | Q9H5M5 | 2 |
| coiled-coil domain containing 86 | CCDC86 | Q9H6F5 | 2 |
| arginine/serine-rich coiled-coil 2 | RSRC2 | Q9H864 | 2 |
| myelin expression factor 2 | MYEF2 | Q9H922 | 2 |
| pumilio homolog 2 (Drosophila) | PUM2 | Q9HAN2 | 2 |
| STIP1 homology and U-box containing protein 1 | STUB1 | Q9HBT1 | 2 |
| centrosomal protein 170kDa | CEP170 | Q9NSN9 | 2 |
| KIAA0947 | KIAA0947 | Q9NTH9 | 2 |
| SAFB-like, transcription modulator | SLTM | Q9NWH9 | 2 |
| ubiquitin specific peptidase 24 | USP24 | Q9NXD1 | 2 |
| dipeptidyl-peptidase 8 | DPP8 | Q9NXF4 | 2 |
| periphilin 1 | PPHLN1 | Q9NXL4 | 2 |
| serine/arginine repetitive matrix 2; hypothetical LOC100132779 | SRRM2 | Q9P0G1 | 2 |
| NHS-like 1 | NHSL1 | Q9P2J0 | 2 |
| phosphatidylinositol 4-kinase, catalytic, beta | PI4KB | Q9UBF8 | 2 |
| transcription factor CP2 | TFCP2 | Q9UD75 | 2 |
| similar to hCG1820375; PRP4 pre-mRNA processing factor 4 homolog B (yeast) | PRPF4B | Q9UEE6 | 2 |
| Treacher Collins-Franceschetti syndrome 1 | TCOF1 | Q9UFD4 | 2 |
| La ribonucleoprotein domain family, member 1 | LARP1 | Q9UFD7 | 2 |
| G-protein signaling modulator 1 (AGS3-like, C. elegans) | GPSM1 | Q9UFS8 | 2 |
| drebrin 1 | DBN1 | Q9UFZ5 | 2 |
| ubiquitin associated protein 2-like | UBAP2L | Q9UGL5 | 2 |
| zinc finger protein 91 homolog (mouse); ZFP91-CNTF ciliary neurotrophic factor | ZFP91 | Q9UI87 | 2 |
| ventral anterior homeobox 2 | VAX2 | Q9UIW0 | 2 |
| synaptopodin 2 | SYNPO2 | Q9UK89 | 2 |
| SON DNA binding protein | SON | Q9UKP9 | 2 |
| microtubule-associated protein 1A | MAP1A | Q9UL09 | 2 |
| coiled-coil domain containing 88A | CCDC88A | Q9ULK8 | 2 |
| KIAA1211 | KIAA1211 | Q9ULK9 | 2 |
| nuclear mitotic apparatus protein 1 | NUMA1 | Q9UNL7 | 2 |
| PDS5, regulator of cohesion maintenance, homolog B (S. cerevisiae) | PDS5B | Q9Y2I5 | 2 |
| ribosomal L1 domain containing 1 | RSL1D1 | Q9Y3Z9 | 2 |
| PDS5, regulator of cohesion maintenance, homolog A (S. cerevisiae) | PDS5A | Q9Y4D4 | 2 |
| family with sequence similarity 179, member B | FAM179B | Q9Y4F4 | 2 |
| chromodomain helicase DNA binding protein 3 | CHD3 | Q9Y4I0 | 2 |
| zinc finger protein 608 | ZNF608 | Q9Y5A1 | 2 |
| oxysterol-binding protein-related protein 8 isoform a | MST120 | AF392452.1 | 2 |
| Novel protein | RP13-347D8.5-001 | AL772284.5 | 2 |
| JMJD2B protein | JMJD2B | BC144292.1 | 2 |
