# Supplementary material for: VENNTURE–A Novel Venn Diagram Investigational Tool for Multiple Pharmacological Dataset Analysis
Source: PLoS One. 2012 May 14;7(5):e36911. doi: 10.1371/journal.pone.0036911 (PMC3351456; doi:10.1371/journal.pone.0036911)
Supplement: Table S9 — Phosphoproteins extracted from 100 nM MeCh-stimulated chronic minimal peroxide (CMP)-state human neuroblastoma SH-SY5Y cells. For each successfully identified protein official symbol, Uniprot accession code and number of peptides recovered are indicated. (DOC) [file pone.0036911.s010.doc]

**Table S9.** Phosphoproteins extracted from 100nM MeCh-stimulated chronic minimal peroxide (CMP)-state human neuroblastoma SH-SY5Y cells. For each successfully identified protein official symbol, Uniprot accession code and number of peptides recovered are indicated.

| **Protein Identification** | **Symbol** | **Accession** | **Peptide** |
| --- | --- | --- | --- |
| dynein, cytoplasmic 1, light intermediate chain 1 | DYNC1LI1 | Q9Y6G9 | 11 |
| PDS5, regulator of cohesion maintenance, homolog A (S. cerevisiae) | PDS5A | Q9Y4D4 | 8 |
| PDS5, regulator of cohesion maintenance, homolog B (S. cerevisiae) | PDS5B | Q9Y2I5 | 8 |
| paralemmin | PALM | Q9UQS3 | 8 |
| nuclear RNA export factor 1 | NXF1 | Q9UQL2 | 5 |
| signal-induced proliferation-associated 1 like 1 | SIPA1L1 | Q9UNU4 | 5 |
| nuclear mitotic apparatus protein 1 | NUMA1 | Q9UNL7 | 5 |
| GATA zinc finger domain containing 2B | GATAD2B | Q9ULS0 | 4 |
| zinc finger protein 236 | ZNF236 | Q9UL37 | 4 |
| microtubule-associated protein 1A | MAP1A | Q9UL09 | 4 |
| SON DNA binding protein | SON | Q9UKP9 | 4 |
| synaptopodin 2 | SYNPO2 | Q9UK89 | 4 |
| cyclin L1 | CCNL1 | Q9UK58 | 3 |
| phospholipase A2, group VI (cytosolic, calcium-independent) | PLA2G6 | Q9UIT0 | 3 |
| drebrin 1 | DBN1 | Q9UFZ5 | 3 |
| Treacher Collins-Franceschetti syndrome 1 | TCOF1 | Q9UFD4 | 3 |
| similar to hCG1820375; PRP4 pre-mRNA processing factor 4 homolog B (yeast) | PRPF4B | Q9UEE6 | 3 |
| heat shock 27kDa protein-like 2 pseudogene; heat shock 27kDa protein 1 | HSPB1 | Q9UC31 | 3 |
| cingulin | CGN | Q9P2M7 | 3 |
| serine/arginine repetitive matrix 2; hypothetical LOC100132779 | SRRM2 | Q9P0G1 | 3 |
| myomesin 1, 185kDa | MYOM1 | Q9NZL8 | 3 |
| vacuolar protein sorting 13 homolog C (S. cerevisiae) | VPS13C | Q9NXN8 | 3 |
| SAFB-like, transcription modulator | SLTM | Q9NWH9 | 3 |
| transmembrane protein 45A | TMEM45A | Q9NWC5 | 3 |
| centrosomal protein 170kDa | CEP170 | Q9NSN9 | 3 |
| eukaryotic translation initiation factor 4E nuclear import factor 1 | EIF4ENIF1 | Q9NRA8 | 2 |
| excision repair cross-complementing rodent repair deficiency, complementation group 5 | ERCC5 | Q9NR54 | 2 |
| STIP1 homology and U-box containing protein 1 | STUB1 | Q9HBT1 | 2 |
| ring finger protein 20 | RNF20 | Q9H9Y7 | 2 |
| coiled-coil domain containing 71 | CCDC71 | Q9H9F1 | 2 |
| ankyrin repeat, family A (RFXANK-like), 2 | ANKRA2 | Q9H9E1 | 2 |
| NAD kinase | NADK | Q9H931 | 2 |
| myelin expression factor 2 | MYEF2 | Q9H922 | 2 |
| H2A histone family, member Y | H2AFY | Q9H8P3 | 2 |
| GINS complex subunit 3 (Psf3 homolog) | GINS3 | Q9H870 | 2 |
| coiled-coil domain containing 86 | CCDC86 | Q9H6F5 | 2 |
| retinoblastoma binding protein 6 | RBBP6 | Q9H5M5 | 2 |
| pericentriolar material 1 | PCM1 | Q9H4A2 | 2 |
| SAM domain and HD domain 1 | SAMHD1 | Q9H3U9 | 2 |
| hematological and neurological expressed 1 | HN1 | Q9H3K0 | 2 |
| activating signal cointegrator 1 complex subunit 3 | ASCC3 | Q9H1I9 | 2 |
| phosphoglucomutase 1 | PGM1 | Q9H1D2 | 2 |
| glycosylphosphatidylinositol specific phospholipase D1 | GPLD1 | Q9H167 | 2 |
| dedicator of cytokinesis 7 | DOCK7 | Q9C092 | 2 |
| BRCA1 interacting protein C-terminal helicase 1 | BRIP1 | Q9BX63 | 2 |
| transforming, acidic coiled-coil containing protein 2 | TACC2 | Q9BVQ1 | 2 |
| polypyrimidine tract binding protein 1 | PTBP1 | Q9BUQ0 | 2 |
| neural proliferation, differentiation and control, 1 | NPDC1 | Q9BTD6 | 2 |
| anaphase promoting complex subunit 1; similar to anaphase promoting complex subunit 1 | ANAPC1 | Q9BSE6 | 2 |
| metastasis associated 1 | MTA1 | Q9BRL8 | 2 |
| microtubule-associated protein 2 | MAP2 | Q99976 | 2 |
| AT rich interactive domain 1A (SWI-like) | ARID1A | Q96T89 | 2 |
| remodeling and spacing factor 1 | RSF1 | Q96T23 | 2 |
| protein tyrosine phosphatase-like A domain containing 1 | PTPLAD1 | Q96T12 | 2 |
| family with sequence similarity 40, member A | FAM40A | Q96SN2 | 2 |
| fizzy/cell division cycle 20 related 1 (Drosophila) | FZR1 | Q96NW8 | 2 |
| septin 5 | SEPT5 | Q96MY5 | 2 |
| solute carrier family 44, member 1 | SLC44A1 | Q96KU3 | 2 |
| zinc finger protein 828 | ZNF828 | Q96JM3 | 2 |
| SWI/SNF related, matrix associated, actin dependent regulator of chromatin, subfamily c, member 2 | SMARCC2 | Q96GY4 | 2 |
| leucine-rich repeats and WD repeat domain containing 1 | LRWD1 | Q96GJ2 | 2 |
| glucocorticoid induced transcript 1 | GLCCI1 | Q96FD0 | 2 |
| cytoplasmic linker associated protein 2 | CLASP2 | Q96F87 | 2 |
| cofactor of BRCA1 | COBRA1 | Q96EW5 | 2 |
| AHNAK nucleoprotein | AHNAK | Q96EC4 | 2 |
| zinc finger CCCH-type containing 18 | ZC3H18 | Q96DG4 | 2 |
| ligase III, DNA, ATP-dependent | LIG3 | Q96DF0 | 2 |
| serine/threonine kinase 11 interacting protein | STK11IP | Q96CN3 | 2 |
| septin 2 | SEPT2 | Q96CB0 | 2 |
| scribbled homolog (Drosophila) | SCRIB | Q96C69 | 2 |
| minichromosome maintenance complex component 2 | MCM2 | Q969W7 | 2 |
| bridging integrator 1 | BIN1 | Q92944 | 2 |
| bromodomain containing 3 | BRD3 | Q92645 | 2 |
| TBC1 domain family, member 5 | TBC1D5 | Q92609 | 2 |
| H1 histone family, member X | H1FX | Q92522 | 2 |
| glutamine-fructose-6-phosphate transaminase 1 | GFPT1 | Q8WYR5 | 2 |
| kinesin light chain 3 | KLC3 | Q8WWJ9 | 2 |
| mutS homolog 6 (E. coli) | MSH6 | Q8TCX4 | 2 |
| death effector domain containing 2 | DEDD2 | Q8NBR2 | 2 |
| DENN/MADD domain containing 4A | DENND4A | Q8NB93 | 2 |
| nucleolin | NCL | Q8NB06 | 2 |
| partner and localizer of BRCA2 | PALB2 | Q8N7Y6 | 2 |
| pleckstrin homology domain containing, family H (with MyTH4 domain) member 2 | PLEKHH2 | Q8N3Q3 | 2 |
| SEC16 homolog A (S. cerevisiae) | SEC16A | Q8N347 | 2 |
| zinc finger protein 687 | ZNF687 | Q8N1G0 | 2 |
| zinc finger protein 683 | ZNF683 | Q8IZ20 | 2 |
| DEAD (Asp-Glu-Ala-Asp) box polypeptide 6 | DDX6 | Q8IV96 | 2 |
| microtubule-associated protein 4 | MAP4 | Q86Y04 | 2 |
| sperm associated antigen 9 | SPAG9 | Q86WC7 | 2 |
| bromodomain adjacent to zinc finger domain, 1B | BAZ1B | Q86UJ6 | 2 |
| von Willebrand factor A domain containing 3B | VWA3B | Q86T73 | 2 |
| pleckstrin homology domain containing, family A member 5 | PLEKHA5 | Q86ST7 | 2 |
| chromodomain helicase DNA binding protein 7 | CHD7 | Q7Z7Q2 | 2 |
| hypothetical protein LOC387763 | AG2 | Q7Z7L8 | 2 |
| spectrin, alpha, non-erythrocytic 1 (alpha-fodrin) | SPTAN1 | Q7Z6M5 | 2 |
| kinesin family member 21A | KIF21A | Q7Z668 | 2 |
| double C2-like domains, alpha | DOC2A | Q7Z5G0 | 2 |
| tumor protein p53 binding protein 1 | TP53BP1 | Q7Z3U4 | 2 |
| ankyrin 2, neuronal | ANK2 | Q7Z3L5 | 2 |
| methyl CpG binding protein 2 (Rett syndrome) | MECP2 | Q7Z384 | 2 |
| titin | TTN | Q7Z2X3 | 2 |
| kinesin light chain 1 | KLC1 | Q7RTQ4 | 2 |
| MICAL-like 1 | MICALL1 | Q7RTP5 | 2 |
| MICAL-like 2 | MICALL2 | Q7RTP4 | 2 |
| cortactin | CTTN | Q76MU0 | 2 |
| RNA binding motif protein 33 | RBM33 | Q75ML5 | 2 |
| synaptopodin | SYNPO | Q71HJ6 | 2 |
| similar to protein phosphatase 1, regulatory subunit 2; protein phosphatase 1, regulatory (inhibitor) | C13orf18 | Q6ZU68 | 2 |
| microtubule-associated protein 1B | MAP1B | Q6PJD3 | 2 |
| sterile alpha motif domain containing 1 | SAMD1 | Q6PIS7 | 2 |
| thyroid hormone receptor associated protein 3 | THRAP3 | Q6P0P7 | 2 |
| MARCKS-like 1 | MARCKSL1 | Q6NXS5 | 2 |
| myristoylated alanine-rich protein kinase C substrate | MARCKS | Q6NVI1 | 2 |
| mediator complex subunit 11 | MED11 | Q6NS89 | 2 |
| LIM and calponin homology domains 1 | LIMCH1 | Q6N054 | 2 |
| mannan-binding lectin serine peptidase 1 (C4/C2 activating component of Ra-reactive factor) | MASP1 | Q6MZL2 | 2 |
| Nipped-B homolog (Drosophila) | NIPBL | Q6KCD6 | 2 |
| RAB12, member RAS oncogene family | RAB12 | Q6IQ22 | 2 |
| GTP binding protein 1 | GTPBP1 | Q6IC67 | 2 |
| proteasome (prosome, macropain) 26S subunit, ATPase, 1 | PSMC1 | Q6IAW0 | 2 |
| eukaryotic translation initiation factor 3, subunit G | EIF3G | Q6IAM0 | 2 |
| potassium intermediate/small conductance calcium-activated channel, subfamily N, member 1 | KCNN1 | Q6DJU4 | 2 |
| similar to Bcl-2-associated transcription factor 1 (Btf); BCL2-associated transcription factor 1 | BCLAF1 | Q6DCA8 | 2 |
| bromodomain adjacent to zinc finger domain, 2A | BAZ2A | Q68DI8 | 2 |
| heterogeneous nuclear ribonucleoprotein H1 (H) | HNRNPH1 | Q68DG4 | 2 |
| ribonucleotide reductase M2 polypeptide | RRM2 | Q5WRU7 | 2 |
| antigen identified by monoclonal antibody Ki-67 | MKI67 | Q5VWH2 | 2 |
| serine/arginine repetitive matrix 1 | SRRM1 | Q5VVN4 | 2 |
| wings apart-like homolog (Drosophila) | WAPAL | Q5VSK5 | 2 |
| GTPase activating protein (SH3 domain) binding protein 1 | G3BP1 | Q5U0Q1 | 2 |
| HORMA domain containing 1 | HORMAD1 | Q5T5I4 | 2 |
| hepatoma-derived growth factor (high-mobility group protein 1-like) | HDGF | Q5SZ07 | 2 |
| death-domain associated protein | DAXX | Q5STR5 | 2 |
| BMS1 homolog, ribosome assembly protein (yeast) pseudogene | BMS1 | Q5QPT5 | 2 |
| deoxynucleotidyltransferase, terminal, interacting protein 2 | DNTTIP2 | Q5QJE6 | 2 |
| RNA binding motif protein, X-linked 2 | RBMX2 | Q5JY82 | 2 |
| family with sequence similarity 76, member B | FAM76B | Q5HYJ3 | 2 |
| sorbin and SH3 domain containing 3 | SORBS3 | Q5BJE4 | 2 |
| topoisomerase (DNA) II beta 180kDa | TOP2B | Q59H80 | 2 |
| drebrin-like | DBNL | Q59FH4 | 2 |
| heat shock protein 90kDa alpha (cytosolic), class B member 2 (pseudogene) | HSP90AB2P | Q58FF8 | 2 |
| dishevelled, dsh homolog 2 (Drosophila) | DVL2 | Q53XM0 | 2 |
| gamma-aminobutyric acid (GABA) A receptor, alpha 5 | GABRA5 | Q53XL6 | 2 |
| erythrocyte membrane protein band 4.1 like 5 | EPB41L5 | Q53T34 | 2 |
| mitogen-activated protein kinase kinase kinase 2 | MAP3K2 | Q53S75 | 2 |
| activating transcription factor 2 | ATF2 | Q53RY2 | 2 |
| spectrin, beta, non-erythrocytic 1 | SPTBN1 | Q53R99 | 2 |
| solute carrier family 35, member C2 | SLC35C2 | Q53GK3 | 2 |
| fibronectin type III domain containing 1 | FNDC1 | Q4ZHG4 | 2 |
| AP2 associated kinase 1 | AAK1 | Q4ZFZ3 | 2 |
| SEC24 family, member D (S. cerevisiae) | SEC24D | Q4W5D3 | 2 |
| heterogeneous nuclear ribonucleoprotein A1-like 3 | HNRPA1L3 | Q3MI39 | 2 |
| reticulon 4 | RTN4 | Q3LIF4 | 2 |
| ubiquitin specific peptidase 42 | USP42 | Q3C166 | 2 |
| heparan sulfate proteoglycan 2 | HSPG2 | Q2VPA1 | 2 |
| LIM domain and actin binding 1 | LIMA1 | Q2TAN7 | 2 |
| low density lipoprotein-related protein 1 (alpha-2-macroglobulin receptor) | LRP1 | Q2PP12 | 2 |
| zinc finger, MYND-type containing 8 | ZMYND8 | Q2HXV9 | 2 |
| KIAA0528 | KIAA0528 | Q17RY7 | 2 |
| ELAV (embryonic lethal, abnormal vision, Drosophila)-like 4 (Hu antigen D) | ELAVL4 | Q16234 | 2 |
| telomeric repeat binding factor 2 | TERF2 | Q15554 | 2 |
| bromodomain containing 2 | BRD2 | Q15310 | 2 |
| non-POU domain containing, octamer-binding | NONO | Q15233 | 2 |
| inositol 1,4,5-triphosphate receptor, type 2 | ITPR2 | Q14571 | 2 |
| phosphoribosyl pyrophosphate synthetase-associated protein 1 | PRPSAP1 | Q14558 | 2 |
| similar to RNA binding motif protein 39; RNA binding motif protein 39 | RBM39 | Q14498 | 2 |
| heterogeneous nuclear ribonucleoprotein D (AU-rich element RNA binding protein 1, 37kDa) | HNRNPD | Q14100 | 2 |
| caldesmon 1 | CALD1 | Q13979 | 2 |
| tumor protein p53 binding protein, 2 | TP53BP2 | Q13625 | 2 |
| ataxia telangiectasia and Rad3 related; similar to ataxia telangiectasia and Rad3 related protein | ATR | Q13535 | 2 |
| interleukin enhancer binding factor 3, 90kDa | ILF3 | Q12906 | 2 |
| NFKB activating protein | NKAP | Q05D22 | 2 |
| glutamyl-prolyl-tRNA synthetase | EPRS | Q05BP6 | 2 |
| tubulin, alpha 4a | TUBA4A | P68366 | 2 |
| TPI1 pseudogene; triosephosphate isomerase 1 | TPI1 | P60174 | 2 |
| integrin, alpha 1 | ITGA1 | P56199 | 2 |
| NOP2 nucleolar protein homolog (yeast) | NOP2 | P46087 | 2 |
| carbamoyl-phosphate synthetase 2, aspartate transcarbamylase, and dihydroorotase | CAD | P27708 | 2 |
| guanylate cyclase 2C (heat stable enterotoxin receptor) | GUCY2C | P25092 | 2 |
| GATA binding protein 3 | GATA3 | P23771 | 2 |
| GATA binding protein 1 (globin transcription factor 1) | GATA1 | P15976 | 2 |
| microtubule-associated protein tau | MAPT | P10636 | 2 |
| androgen receptor | AR | P10275 | 2 |
| chromosome 14 open reading frame 38 | C14orf38 | P0C221 | 2 |
| thymopoietin | TMPO | P08919 | 2 |
| ribosomal protein S17 | RPS17 | P08708 | 2 |
| p21 protein (Cdc42/Rac)-activated kinase 4 | PAK4 | O96013 | 2 |
| eukaryotic translation initiation factor 5B | EIF5B | O95805 | 2 |
| MYST histone acetyltransferase 2; similar to MYST histone acetyltransferase 2 | MYST2 | O95251 | 2 |
| kelch repeat and BTB (POZ) domain containing 11 | KBTBD11 | O94819 | 2 |
| protein kinase D3 | PRKD3 | O94806 | 2 |
| regulator of G-protein signaling 12 | RGS12 | O75338 | 2 |
| eukaryotic translation initiation factor 4 gamma, 3 | EIF4G3 | O43432 | 2 |
| glycogen synthase kinase 3 alpha | GSK3A | O14959 | 2 |
| paired-like homeobox 2a | PHOX2A | O14813 | 2 |
| TRAF-type zinc finger domain containing 1 | TRAFD1 | O14545 | 2 |
| protein phosphatase 1, regulatory (inhibitor) subunit 10 | PPP1R10 | O00405 | 2 |
| suppressor of Ty 5 homolog (S. cerevisiae) | SUPT5H | O00267 | 2 |
| chromosome 17 open reading frame 49 | C17orf49 | C9J4G0 | 2 |
| nuclear receptor subfamily 1, group H, member 4 | NR1H4 | B7Z412 | 2 |
| inositol(myo)-1(or 4)-monophosphatase 1 | IMPA1 | B4DLN3 | 2 |
| zinc finger protein 391 | ZNF391 | B4DH77 | 2 |
| kinesin light chain 4 | KLC4 | B3KSQ3 | 2 |
| chemokine (C-C motif) ligand 14; chemokine (C-C motif) ligand 15 | CCL14 | B2RU34 | 2 |
| ERBB receptor feedback inhibitor 1 | ERRFI1 | B2RDX9 | 2 |
| ribosomal protein S3 pseudogene 3; ribosomal protein S3 | RPS3 | B2R7N5 | 2 |
| interferon regulatory factor 2 binding protein 2 | IRF2BP2 | B1AM36 | 2 |
| HLA-B associated transcript 3 | BAT3 | B0UX84 | 2 |
| neural cell adhesion molecule 1 | NCAM1 | A8K8T8 | 2 |
| D4, zinc and double PHD fingers family 2 | DPF2 | A8K7C9 | 2 |
| potassium channel tetramerisation domain containing 15 | KCTD15 | A8K600 | 2 |
| leucine rich repeat containing 41 | LRRC41 | A8K5G8 | 2 |
| Paralemmin-3 | PALM3 | A6NDB9 | 2 |
| family with sequence similarity 54, member B | FAM54B | A6NCB4 | 2 |
| chromosome 7 open reading frame 47 | C7orf47 | A4D2C5 | 2 |
| AF4/FMR2 family, member 2 | AFF2 | A2RTY4 | 2 |
| stathmin 1 | STMN1 | A2A2D1 | 2 |
| cysteine-rich protein 2 | CRIP2 | A1A4U1 | 2 |
| zinc finger CCCH-type containing 13 | ZC3H13 | A0PJJ2 | 2 |
