# Supplementary material for: VENNTURE–A Novel Venn Diagram Investigational Tool for Multiple Pharmacological Dataset Analysis
Source: PLoS One. 2012 May 14;7(5):e36911. doi: 10.1371/journal.pone.0036911 (PMC3351456; doi:10.1371/journal.pone.0036911)
Supplement: Table S10 — Phosphoproteins extracted from 1 µM MeCh-stimulated chronic minimal peroxide (CMP)-state human neuroblastoma SH-SY5Y cells. For each successfully identified protein official symbol, Uniprot accession code and number of peptides recovered are indicated. (DOC) [file pone.0036911.s011.doc]

**Table S10.** Phosphoproteins extracted from 1µM MeCh-stimulated chronic minimal peroxide (CMP)-state human neuroblastoma SH-SY5Y cells. For each successfully identified protein official symbol, Uniprot accession code and number of peptides recovered are indicated.

| **Protein Identification** | **Symbol** | **Accession** | **Peptide** |
| --- | --- | --- | --- |
| zinc finger RNA binding protein | ZFR | Q9Y687 | 28 |
| solute carrier family 9 (sodium/hydrogen exchanger), member 5 | SLC9A5 | Q9Y626 | 27 |
| leucine rich repeat (in FLII) interacting protein 2 | LRRFIP2 | Q9Y608 | 24 |
| interferon regulatory factor 2 binding protein 1 | IRF2BP1 | Q9Y4P4 | 23 |
| chromodomain helicase DNA binding protein 3 | CHD3 | Q9Y4I0 | 17 |
| PDS5, regulator of cohesion maintenance, homolog A (S. cerevisiae) | PDS5A | Q9Y4D4 | 16 |
| ribosomal RNA processing 12 homolog (S. cerevisiae) | RRP12 | Q9Y4C7 | 15 |
| ribosomal L1 domain containing 1 | RSL1D1 | Q9Y3Z9 | 12 |
| La ribonucleoprotein domain family, member 7 | LARP7 | Q9Y3Z8 | 11 |
| inhibitor of Bruton agammaglobulinemia tyrosine kinase | IBTK | Q9Y3T8 | 11 |
| PDS5, regulator of cohesion maintenance, homolog B (S. cerevisiae) | PDS5B | Q9Y2I5 | 10 |
| pleckstrin homology domain containing, family A member 6 | PLEKHA6 | Q9Y2H5 | 10 |
| PDZ and LIM domain 4 | PDLIM4 | Q9Y292 | 9 |
| GRB2-associated binding protein 2 | GAB2 | Q9UQC2 | 9 |
| signal-induced proliferation-associated 1 like 1 | SIPA1L1 | Q9UNU4 | 9 |
| nuclear mitotic apparatus protein 1 | NUMA1 | Q9UNL7 | 9 |
| apolipoprotein B (including Ag(x) antigen) | APOB | Q9UMN0 | 8 |
| CD2 (cytoplasmic tail) binding protein 2 | CD2BP2 | Q9ULP2 | 8 |
| shroom family member 4 | SHROOM4 | Q9ULL8 | 8 |
| KIAA1211 | KIAA1211 | Q9ULK9 | 8 |
| ArfGAP with SH3 domain, ankyrin repeat and PH domain 1 | ASAP1 | Q9ULH1 | 7 |
| microtubule-associated protein 1A | MAP1A | Q9UL09 | 7 |
| nucleoporin 50kDa | NUP50 | Q9UKX7 | 7 |
| SON DNA binding protein | SON | Q9UKP9 | 7 |
| G patch domain containing 8 | GPATCH8 | Q9UKJ3 | 6 |
| synaptopodin 2 | SYNPO2 | Q9UK89 | 6 |
| chromatin assembly factor 1, subunit A (p150) | CHAF1A | Q9UJY8 | 6 |
| neurofilament, heavy polypeptide | NEFH | Q9UJS7 | 6 |
| kinesin family member 25 | KIF25 | Q9UIL4 | 6 |
| nucleoporin 98kDa | NUP98 | Q9UHX0 | 6 |
| caspase recruitment domain family, member 10 | CARD10 | Q9UGR6 | 6 |
| LIM domain binding 1 | LDB1 | Q9UGM4 | 6 |
| ubiquitin associated protein 2-like | UBAP2L | Q9UGL5 | 6 |
| progesterone receptor membrane component 1 | PGRMC1 | Q9UGJ9 | 6 |
| regulatory factor X, 5 (influences HLA class II expression) | RFX5 | Q9UG77 | 6 |
| drebrin 1 | DBN1 | Q9UFZ5 | 6 |
| G-protein signaling modulator 1 (AGS3-like, C. elegans) | GPSM1 | Q9UFS8 | 6 |
| Treacher Collins-Franceschetti syndrome 1 | TCOF1 | Q9UFD4 | 6 |
| MYB binding protein (P160) 1a | MYBBP1A | Q9UF99 | 6 |
| similar to hCG1820375; PRP4 pre-mRNA processing factor 4 homolog B (yeast) | PRPF4B | Q9UEE6 | 6 |
| ubiquitin specific peptidase 6 (Tre-2 oncogene) | USP6 | Q9UDD3 | 5 |
| transcription factor CP2 | TFCP2 | Q9UD75 | 5 |
| heat shock 27kDa protein-like 2 pseudogene; heat shock 27kDa protein 1 | HSPB1 | Q9UC31 | 5 |
| phosphatidylinositol 4-kinase, catalytic, beta | PI4KB | Q9UBF8 | 5 |
| sema domain, transmembrane domain (TM), and cytoplasmic domain, (semaphorin) 6D | SEMA6D | Q9P249 | 5 |
| forkhead box N3 | FOXN3 | Q9P1I8 | 5 |
| Rho guanine nucleotide exchange factor (GEF) 12 | ARHGEF12 | Q9P149 | 5 |
| serine/arginine repetitive matrix 2; hypothetical LOC100132779 | SRRM2 | Q9P0G1 | 5 |
| kinesin family member 4B; kinesin family member 4A | KIF4A | Q9NY24 | 5 |
| periphilin 1 | PPHLN1 | Q9NXL4 | 5 |
| SAFB-like, transcription modulator | SLTM | Q9NWH9 | 5 |
| PRP38 pre-mRNA processing factor 38 (yeast) domain containing B | PRPF38B | Q9NW40 | 5 |
| protein phosphatase 1, regulatory (inhibitor) subunit 12C | PPP1R12C | Q9NT00 | 5 |
| centrosomal protein 170kDa | CEP170 | Q9NSN9 | 5 |
| eukaryotic translation initiation factor 4E nuclear import factor 1 | EIF4ENIF1 | Q9NRA8 | 5 |
| potassium voltage-gated channel, KQT-like subfamily, member 5 | KCNQ5 | Q9NR82 | 5 |
| excision repair cross-complementing rodent repair deficiency, complementation group 5 | ERCC5 | Q9NR54 | 5 |
| DEAD (Asp-Glu-Ala-Asp) box polypeptide 21 | DDX21 | Q9NR30 | 5 |
| exosome component 5 | EXOSC5 | Q9NQT4 | 4 |
| peter pan homolog (Drosophila) | PPAN | Q9NQ55 | 4 |
| chromosome 7 open reading frame 54 | C7orf54 | Q9HBX3 | 4 |
| STIP1 homology and U-box containing protein 1 | STUB1 | Q9HBT1 | 4 |
| pumilio homolog 2 (Drosophila) | PUM2 | Q9HAN2 | 4 |
| RAB24, member RAS oncogene family | RAB24 | Q9HAG2 | 4 |
| ring finger protein 20 | RNF20 | Q9H9Y7 | 4 |
| zinc finger, DHHC-type containing 5 | ZDHHC5 | Q9H923 | 4 |
| myelin expression factor 2 | MYEF2 | Q9H922 | 4 |
| cache domain containing 1 | CACHD1 | Q9H7W4 | 4 |
| enhancer of mRNA decapping 3 homolog (S. cerevisiae) | EDC3 | Q9H797 | 4 |
| erythrocyte membrane protein band 4.1 like 4B | EPB41L4B | Q9H709 | 4 |
| coiled-coil domain containing 86 | CCDC86 | Q9H6F5 | 4 |
| retinoblastoma binding protein 6 | RBBP6 | Q9H5M5 | 4 |
| pericentriolar material 1 | PCM1 | Q9H4A2 | 4 |
| histidyl-tRNA synthetase 2, mitochondrial (putative); D-tyrosyl-tRNA deacylase 1 homolog (S. cerevisiae) | DTD1 | Q9H464 | 4 |
| tumor protein D52-like 2 | TPD52L2 | Q9H3Z6 | 4 |
| DnaJ (Hsp40) homolog, subfamily C, member 5 | DNAJC5 | Q9H3Z5 | 4 |
| SAM domain and HD domain 1 | SAMHD1 | Q9H3U9 | 4 |
| hematological and neurological expressed 1 | HN1 | Q9H3K0 | 4 |
| SAPS domain family, member 3 | SAPS3 | Q9H2K6 | 4 |
| nuclear casein kinase and cyclin-dependent kinase substrate 1 | NUCKS1 | Q9H1E3 | 4 |
| phosphoglucomutase 1 | PGM1 | Q9H1D2 | 3 |
| family with sequence similarity 186, member B | FAM186B | Q9H0L3 | 3 |
| FIP1 like 1 (S. cerevisiae) | FIP1L1 | Q9H077 | 3 |
| dedicator of cytokinesis 7 | DOCK7 | Q9C092 | 3 |
| solute carrier family 25 (mitochondrial carrier; ornithine transporter) member 2 | SLC25A2 | Q9BXI2 | 3 |
| dual specificity phosphatase 15; chromosome 20 open reading frame 57 | DUSP15 | Q9BX24 | 3 |
| mitochondrial fission factor | MFF | Q9BVZ1 | 3 |
| chromodomain helicase DNA binding protein 1-like | CHD1L | Q9BVJ1 | 3 |
| phosphatidylserine synthase 2 | PTDSS2 | Q9BVG9 | 3 |
| proline/serine-rich coiled-coil 1 | PSRC1 | Q9BV77 | 3 |
| SERPINE1 mRNA binding protein 1 | SERBP1 | Q9BUM4 | 3 |
| transgelin 2 | TAGLN2 | Q9BUH5 | 3 |
| chromosome 11 open reading frame 84 | C11orf84 | Q9BUA3 | 3 |
| polyhomeotic homolog 1B (Drosophila); polyhomeotic homolog 1 (Drosophila) | PHC1 | Q9BU63 | 3 |
| single stranded DNA binding protein 3; hypothetical LOC100131851 | SSBP3 | Q9BTM0 | 3 |
| KIAA1429 | KIAA1429 | Q9BTH4 | 3 |
| neural proliferation, differentiation and control, 1 | NPDC1 | Q9BTD6 | 3 |
| mitochondrial ribosomal protein S2 | MRPS2 | Q9BSQ4 | 3 |
| anaphase promoting complex subunit 1; similar to anaphase promoting complex subunit 1 | ANAPC1 | Q9BSE6 | 3 |
| metastasis associated 1 | MTA1 | Q9BRL8 | 3 |
| chromosome 7 open reading frame 50 | C7orf50 | Q9BRJ6 | 3 |
| torsin family 1, member B (torsin B) | TOR1B | Q9BR69 | 3 |
| RD RNA binding protein | RDBP | Q9BQJ6 | 3 |
| superkiller viralicidic activity 2-like (S. cerevisiae) | SKIV2L | Q9BQJ5 | 3 |
| microtubule-associated protein 2 | MAP2 | Q99976 | 3 |
| A kinase (PRKA) anchor protein 12 | AKAP12 | Q99970 | 3 |
| myosin, heavy chain 9, non-muscle | MYH9 | Q99529 | 3 |
| AT rich interactive domain 1A (SWI-like) | ARID1A | Q96T89 | 3 |
| remodeling and spacing factor 1 | RSF1 | Q96T23 | 3 |
| protein tyrosine phosphatase-like A domain containing 1 | PTPLAD1 | Q96T12 | 3 |
| family with sequence similarity 40, member A | FAM40A | Q96SN2 | 3 |
| checkpoint with forkhead and ring finger domains | CHFR | Q96SL3 | 3 |
| arginine/serine-rich coiled-coil 1 | RSRC1 | Q96QK2 | 3 |
| G protein regulated inducer of neurite outgrowth 1 | GPRIN1 | Q96PZ4 | 3 |
| chromosome 14 open reading frame 145 | C14orf145 | Q96ML4 | 3 |
| chromosome 9 open reading frame 84 | C9orf84 | Q96M73 | 3 |
| chromosome 3 open reading frame 30 | C3orf30 | Q96M34 | 3 |
| zinc finger protein 474 | ZNF474 | Q96M07 | 3 |
| hemoglobin, alpha 2; hemoglobin, alpha 1 | HBA1 | Q96KF1 | 2 |
| dachsous 1 (Drosophila) | DCHS1 | Q96JQ0 | 2 |
| zinc finger protein 828 | ZNF828 | Q96JM3 | 2 |
| retinoic acid induced 1 | RAI1 | Q96JK5 | 2 |
| MAP/microtubule affinity-regulating kinase 4 | MARK4 | Q96JG7 | 2 |
| protein phosphatase 1, regulatory (inhibitor) subunit 16A | PPP1R16A | Q96I34 | 2 |
| U2 small nuclear RNA auxiliary factor 2 | U2AF2 | Q96HC5 | 2 |
| SWI/SNF related, matrix associated, actin dependent regulator of chromatin, subfamily c, member 2 | SMARCC2 | Q96GY4 | 2 |
| leucine-rich repeats and WD repeat domain containing 1 | LRWD1 | Q96GJ2 | 2 |
| claudin 15 | CLDN15 | Q96FX9 | 2 |
| glucocorticoid induced transcript 1 | GLCCI1 | Q96FD0 | 2 |
| zinc finger with KRAB and SCAN domains 1 | ZKSCAN1 | Q96FA2 | 2 |
| cytoplasmic linker associated protein 2 | CLASP2 | Q96F87 | 2 |
| cofactor of BRCA1 | COBRA1 | Q96EW5 | 2 |
| ribophorin II | RPN2 | Q96E21 | 2 |
| zinc finger CCCH-type containing 18 | ZC3H18 | Q96DG4 | 2 |
| coiled-coil domain containing 124 | CCDC124 | Q96CT7 | 2 |
| serine/threonine kinase 11 interacting protein | STK11IP | Q96CN3 | 2 |
| septin 2 | 40788 | Q96CB0 | 2 |
| scribbled homolog (Drosophila) | SCRIB | Q96C69 | 2 |
| ATPase, class II, type 9A | ATP9A | Q96B35 | 2 |
| minichromosome maintenance complex component 2 | MCM2 | Q969W7 | 2 |
| eukaryotic translation elongation factor 1 delta (guanine nucleotide exchange protein) | EEF1D | Q969J1 | 2 |
| bromodomain containing 3 | BRD3 | Q92645 | 2 |
| H1 histone family, member X | H1FX | Q92522 | 2 |
| twist homolog 1 (Drosophila) | TWIST1 | Q92487 | 2 |
| damage-specific DNA binding protein 2, 48kDa | DDB2 | Q92466 | 2 |
| telomeric repeat binding factor 2, interacting protein | TERF2IP | Q8WYZ3 | 2 |
| family with sequence similarity 48, member A | FAM48A | Q8WYR6 | 2 |
| vacuolar protein sorting 11 homolog (S. cerevisiae) | VPS11 | Q8WY89 | 2 |
| centrosomal protein 350kDa | CEP350 | Q8WY20 | 2 |
| spectrin repeat containing, nuclear envelope 2 | SYNE2 | Q8WWW3 | 2 |
| myosin IXB | MYO9B | Q8WVD2 | 2 |
| cyclin Y | CCNY | Q8TEX3 | 2 |
| glutamate receptor, metabotropic 3 | GRM3 | Q8TBH9 | 2 |
| prospero homeobox 1 | PROX1 | Q8TB91 | 2 |
| WD repeat domain 43 | WDR43 | Q8TB67 | 2 |
| hypothetical LOC642946 | LQK1 | Q8TAF5 | 2 |
| zinc finger protein 318 | ZNF318 | Q8NEM6 | 2 |
| zinc finger protein 663 | ZNF663 | Q8NDT4 | 2 |
| testis expressed 14 | TEX14 | Q8ND97 | 2 |
| pygopus homolog 2 (Drosophila) | PYGO2 | Q8NBG9 | 2 |
| DENN/MADD domain containing 4A | DENND4A | Q8NB93 | 2 |
| nucleolin | NCL | Q8NB06 | 2 |
| PRP38 pre-mRNA processing factor 38 (yeast) domain containing A | PRPF38A | Q8NAV1 | 2 |
| dihydropyrimidinase-like 2 | DPYSL2 | Q8NAN9 | 2 |
| similar to RNA binding motif protein, X-linked; similar to hCG2011544; RNA binding motif protein, X-linked | RBMX | Q8N8Y7 | 2 |
| dispatched homolog 1 (Drosophila) | DISP1 | Q8N7C2 | 2 |
| splicing factor, arginine/serine-rich 17A | SFRS17A | Q8N6U9 | 2 |
| cytoplasmic linker associated protein 1 | CLASP1 | Q8N5B8 | 2 |
| DEAH (Asp-Glu-Ala-Asp/His) box polypeptide 57 | DHX57 | Q8N4U2 | 2 |
| jumonji domain containing 1C | JMJD1C | Q8N3U0 | 2 |
| heterogeneous nuclear ribonucleoprotein U-like 2 | HNRNPUL2 | Q8N3B3 | 2 |
| zinc finger protein 687 | ZNF687 | Q8N1G0 | 2 |
| serpin peptidase inhibitor, clade B (ovalbumin), member 8 | SERPINB8 | Q8N178 | 2 |
| 5'-3' exoribonuclease 1 | XRN1 | Q8IZH2 | 2 |
| zinc finger protein 683 | ZNF683 | Q8IZ20 | 2 |
| cartilage intermediate layer protein, nucleotide pyrophosphohydrolase | CILP | Q8IYI5 | 2 |
| suppressor of var1, 3-like 1 (S. cerevisiae) | SUPV3L1 | Q8IYB8 | 2 |
| erythrocyte membrane protein band 4.1 (elliptocytosis 1, RH-linked) | EPB41 | Q8IXV9 | 2 |
| DEAD (Asp-Glu-Ala-Asp) box polypeptide 51 | DDX51 | Q8IXK5 | 2 |
| chromosome 7 open reading frame 27 | C7orf27 | Q8IW85 | 2 |
| lipoxygenase homology domains 1 | LOXHD1 | Q8IVV2 | 2 |
| EPH receptor A8 | EPHA8 | Q8IUX6 | 2 |
| signal-induced proliferation-associated 1 like 3 | SIPA1L3 | Q8IUV1 | 2 |
| DEAD (Asp-Glu-Ala-Asp) box polypeptide 54 | DDX54 | Q86YT8 | 2 |
| microtubule-associated protein 4 | MAP4 | Q86Y04 | 2 |
| chromosome 17 open reading frame 82 | C17orf82 | Q86X59 | 2 |
| kinectin 1 (kinesin receptor) | KTN1 | Q86W57 | 2 |
| erbb2 interacting protein | ERBB2IP | Q86W38 | 2 |
| DnaJ (Hsp40) homolog, subfamily C, member 21 | DNAJC21 | Q86VC6 | 2 |
| bromodomain adjacent to zinc finger domain, 1B | BAZ1B | Q86UJ6 | 2 |
| taxilin alpha | TXLNA | Q86T86 | 2 |
| hypothetical protein LOC387763 | AG2 | Q7Z7L8 | 2 |
| kinesin family member 21A | KIF21A | Q7Z668 | 2 |
| mucin 19, oligomeric | MUC19 | Q7Z5P9 | 2 |
| eukaryotic translation initiation factor 2A, 65kDa | EIF2A | Q7Z4E9 | 2 |
| tumor protein p53 binding protein 1 | TP53BP1 | Q7Z3U4 | 2 |
| WD repeat domain 44 | WDR44 | Q7Z3P6 | 2 |
| cleavage and polyadenylation specific factor 7, 59kDa | CPSF7 | Q7Z3H9 | 2 |
| methyl CpG binding protein 2 (Rett syndrome) | MECP2 | Q7Z384 | 2 |
| alpha thalassemia/mental retardation syndrome X-linked (RAD54 homolog, S. cerevisiae) | ATRX | Q7Z2J1 | 2 |
| similar to U5 snRNP-specific protein, 200 kDa; small nuclear ribonucleoprotein 200kDa (U5) | SNRNP200 | Q7L5W4 | 2 |
| pinin, desmosome associated protein | PNN | Q7KYL1 | 2 |
| cortactin | CTTN | Q76MU0 | 2 |
| ligase I, DNA, ATP-dependent | LIG1 | Q76GR4 | 2 |
| nucleoporin 214kDa | NUP214 | Q75R47 | 2 |
| ADAM metallopeptidase domain 22 | ADAM22 | Q75MS7 | 2 |
| cytokine inducible SH2-containing protein | CISH | Q71V34 | 2 |
| synaptopodin | SYNPO | Q71HJ6 | 2 |
| Rho GTPase activating protein 17 | ARHGAP17 | Q6ZUS4 | 2 |
| formin 1 | FMN1 | Q6ZSY1 | 2 |
| shisa homolog 2 (Xenopus laevis) | SHISA2 | Q6UWI4 | 2 |
| estrogen receptor binding site associated, antigen, 9 | EBAG9 | Q6R3F1 | 2 |
| zinc finger CCCH-type containing 14 | ZC3H14 | Q6PUI8 | 2 |
| splicing factor, arginine/serine-rich 11 | SFRS11 | Q6PJY9 | 2 |
| KH domain containing, RNA binding, signal transduction associated 1 | KHDRBS1 | Q6PJX7 | 2 |
| microtubule-associated protein 1B | MAP1B | Q6PJD3 | 2 |
| Kruppel-like factor 3 (basic) | KLF3 | Q6PIR1 | 2 |
| RAS protein activator like 2 | RASAL2 | Q6P4F9 | 2 |
| thyroid hormone receptor associated protein 3 | THRAP3 | Q6P0P7 | 2 |
| MARCKS-like 1 | MARCKSL1 | Q6NXS5 | 2 |
| myristoylated alanine-rich protein kinase C substrate | MARCKS | Q6NVI1 | 2 |
| trinucleotide repeat containing 6A | TNRC6A | Q6NVB5 | 2 |
| LIM and calponin homology domains 1 | LIMCH1 | Q6N054 | 2 |
| Nipped-B homolog (Drosophila) | NIPBL | Q6KCD6 | 2 |
| heterogeneous nuclear ribonucleoprotein K; similar to heterogeneous nuclear ribonucleoprotein K | HNRNPK | Q6IBN1 | 2 |
| lymphocyte antigen 6 complex, locus H | LY6H | Q6IAX0 | 2 |
| eukaryotic translation initiation factor 3, subunit G | EIF3G | Q6IAM0 | 2 |
| mitogen-activated protein kinase associated protein 1 | MAPKAP1 | Q6GVJ2 | 2 |
| matrix metallopeptidase 14 (membrane-inserted) | MMP14 | Q6GSF3 | 2 |
| similar to Bcl-2-associated transcription factor 1 (Btf); BCL2-associated transcription factor 1 | BCLAF1 | Q6DCA8 | 2 |
| ring finger protein 40 | RNF40 | Q6AHZ6 | 2 |
| ATP-binding cassette, sub-family F (GCN20), member 1 | ABCF1 | Q69YP6 | 2 |
| heterogeneous nuclear ribonucleoprotein H1 (H) | HNRNPH1 | Q68DG4 | 2 |
| ribonucleotide reductase M2 polypeptide | RRM2 | Q5WRU7 | 2 |
| zinc finger, MYM-type 4 | ZMYM4 | Q5VZL5 | 2 |
| antigen identified by monoclonal antibody Ki-67 | MKI67 | Q5VWH2 | 2 |
| serine/arginine repetitive matrix 1 | SRRM1 | Q5VVN4 | 2 |
| myeloid cell nuclear differentiation antigen | MNDA | Q5VUU6 | 2 |
| wings apart-like homolog (Drosophila) | WAPAL | Q5VSK5 | 2 |
| GTPase activating protein (SH3 domain) binding protein 1 | G3BP1 | Q5U0Q1 | 2 |
| chromosome 22 open reading frame 30 | C22orf30 | Q5THK4 | 2 |
| AT hook, DNA binding motif, containing 1 | AHDC1 | Q5TGY4 | 2 |
| DNA methyltransferase 1 associated protein 1 | DMAP1 | Q5TG40 | 2 |
| outer dense fiber of sperm tails 2-like | ODF2L | Q5TBX3 | 2 |
| nucleoporin 153kDa | NUP153 | Q5T9I7 | 2 |
| bystin-like | BYSL | Q5T8J2 | 2 |
| HORMA domain containing 1 | HORMAD1 | Q5T5I4 | 2 |
| hepatoma-derived growth factor (high-mobility group protein 1-like) | HDGF | Q5SZ07 | 2 |
| death-domain associated protein | DAXX | Q5STR5 | 2 |
| calcium channel, voltage-dependent, beta 2 subunit | CACNB2 | Q5QJA0 | 2 |
| septin 7 | 40793 | Q5JXL7 | 2 |
| NSFL1 (p97) cofactor (p47) | NSFL1C | Q5JXA5 | 2 |
| karyopherin alpha 3 (importin alpha 4) | KPNA3 | Q5JVN1 | 2 |
| FERM, RhoGEF (ARHGEF) and pleckstrin domain protein 1 (chondrocyte-derived) | FARP1 | Q5JV94 | 2 |
| tight junction associated protein 1 (peripheral) | TJAP1 | Q5JTD1 | 2 |
| phosphoglycerate kinase 1 | PGK1 | Q5J7W1 | 2 |
| lamin A/C | LMNA | Q5I6Y6 | 2 |
| family with sequence similarity 76, member B | FAM76B | Q5HYJ3 | 2 |
| cleavage stimulation factor, 3' pre-RNA, subunit 2, 64kDa | CSTF2 | Q5H951 | 2 |
| activity-dependent neuroprotector homeobox | ADNP | Q5BKU2 | 2 |
| sorbin and SH3 domain containing 3 | SORBS3 | Q5BJE4 | 2 |
| topoisomerase (DNA) II beta 180kDa | TOP2B | Q59H80 | 2 |
| sperm antigen with calponin homology and coiled-coil domains 1-like | SPECC1L | Q59GT7 | 2 |
| cyclin K | CCNK | Q59FT6 | 2 |
| drebrin-like | DBNL | Q59FH4 | 2 |
| insulin-like growth factor 2 receptor | IGF2R | Q59EZ3 | 2 |
| aryl hydrocarbon receptor nuclear translocator | ARNT | Q59ED4 | 2 |
| heat shock protein 90kDa alpha (cytosolic), class B member 2 (pseudogene) | HSP90AB2P | Q58FF8 | 2 |
| protein tyrosine phosphatase, non-receptor type 4 (megakaryocyte) | PTPN4 | Q580X3 | 2 |
| dapper, antagonist of beta-catenin, homolog 2 (Xenopus laevis) | DACT2 | Q569G0 | 2 |
| POTE ankyrin domain family, member E | A26C1A | Q562Q2 | 2 |
| thyroid hormone receptor interactor 12 | TRIP12 | Q53TE7 | 2 |
| activating transcription factor 2 | ATF2 | Q53RY2 | 2 |
| abl interactor 2 | ABI2 | Q53RS4 | 2 |
| spectrin, beta, non-erythrocytic 1 | SPTBN1 | Q53R99 | 2 |
| general transcription factor IIIC, polypeptide 2, beta 110kDa | GTF3C2 | Q53QN0 | 2 |
| RAD18 homolog (S. cerevisiae) | RAD18 | Q53H10 | 2 |
| RNA binding protein, autoantigenic (hnRNP-associated with lethal yellow homolog (mouse)) | RALY | Q53GL6 | 2 |
| solute carrier family 35, member C2 | SLC35C2 | Q53GK3 | 2 |
| chromosome 12 open reading frame 49 | C12orf49 | Q53GE8 | 2 |
| chromogranin A (parathyroid secretory protein 1) | CHGA | Q53FA8 | 2 |
| AP2 associated kinase 1 | AAK1 | Q4ZFZ3 | 2 |
| yrdC domain containing (E. coli) | YRDC | Q4W4X8 | 2 |
| chromodomain helicase DNA binding protein 9 | CHD9 | Q461N2 | 2 |
| heterogeneous nuclear ribonucleoprotein A1-like 3 | HNRPA1L3 | Q3MI39 | 2 |
| transmembrane protein 95 | TMEM95 | Q3KNT9 | 2 |
| ubiquitin specific peptidase 42 | USP42 | Q3C166 | 2 |
| HECT, UBA and WWE domain containing 1 | HUWE1 | Q3B7K0 | 2 |
| calcium regulated heat stable protein 1, 24kDa | CARHSP1 | Q2YDX5 | 2 |
| nestin | NES | Q2YDX4 | 2 |
| heparan sulfate proteoglycan 2 | HSPG2 | Q2VPA1 | 2 |
| receptor-interacting serine-threonine kinase 2 | RIPK2 | Q2TU65 | 2 |
| LIM domain and actin binding 1 | LIMA1 | Q2TAN7 | 2 |
| RNA binding motif protein 26 | RBM26 | Q2NKM2 | 2 |
| similar to Rho-associated, coiled-coil containing protein kinase 1; Rho-associated, coiled-coil containing protein kinase 1 | ROCK1 | Q2KHM4 | 2 |
| KIAA0528 | KIAA0528 | Q17RY7 | 2 |
| SWI/SNF related, matrix associated, actin dependent regulator of chromatin, subfamily c, member 1 | SMARCC1 | Q17RS0 | 2 |
| ELAV (embryonic lethal, abnormal vision, Drosophila)-like 4 (Hu antigen D) | ELAVL4 | Q16234 | 2 |
| adducin 1 (alpha) | ADD1 | Q16156 | 2 |
| small nuclear ribonucleoprotein 70kDa (U1) | SNRNP70 | Q15687 | 2 |
| telomeric repeat binding factor 2 | TERF2 | Q15554 | 2 |
| non-POU domain containing, octamer-binding | NONO | Q15233 | 2 |
| Ctr9, Paf1/RNA polymerase II complex component, homolog (S. cerevisiae) | CTR9 | Q15015 | 2 |
| chromosome 20 open reading frame 117 | C20orf117 | Q14DB2 | 2 |
| poly(rC) binding protein 1 | PCBP1 | Q14975 | 2 |
| phosphoprotein enriched in astrocytes 15 | PEA15 | Q14801 | 2 |
| similar to RNA binding motif protein 39; RNA binding motif protein 39 | RBM39 | Q14498 | 2 |
| heterogeneous nuclear ribonucleoprotein D (AU-rich element RNA binding protein 1, 37kDa) | HNRNPD | Q14100 | 2 |
| chromatin assembly factor 1, subunit B (p60) | CHAF1B | Q13112 | 2 |
| RAN binding protein 2 | RANBP2 | Q13073 | 2 |
| interleukin enhancer binding factor 3, 90kDa | ILF3 | Q12906 | 2 |
| testis specific protein, Y-linked 2 | TSPY2 | Q0VAD3 | 2 |
| dehydrogenase/reductase (SDR family) member 3 | DHRS3 | Q0QD44 | 2 |
| PNMA-like 2 | PNMAL2 | Q08E79 | 2 |
| NFKB activating protein | NKAP | Q05D22 | 2 |
| Dmx-like 1 | DMXL1 | Q05C95 | 2 |
| glutamyl-prolyl-tRNA synthetase | EPRS | Q05BP6 | 2 |
| TPI1 pseudogene; triosephosphate isomerase 1 | TPI1 | P60174 | 2 |
| ATP citrate lyase | ACLY | P53396 | 2 |
| gamma-aminobutyric acid (GABA) A receptor, alpha 4 | GABRA4 | P48169 | 2 |
| galanin receptor 1 | GALR1 | P47211 | 2 |
| NOP2 nucleolar protein homolog (yeast) | NOP2 | P46087 | 2 |
| solute carrier family 1 (glutamate/neutral amino acid transporter), member 4 | SLC1A4 | P43007 | 2 |
| phosphatidylinositol glycan anchor biosynthesis, class A | PIGA | P37287 | 2 |
| guanylate cyclase 2C (heat stable enterotoxin receptor) | GUCY2C | P25092 | 2 |
| transcription elongation factor A (SII), 1 pseudogene 2; transcription elongation factor A (SII), 1 | TCEA1 | P23193 | 2 |
| microtubule-associated protein tau | MAPT | P10636 | 2 |
| neuropeptide S | NPS | P0C0P6 | 2 |
| thymopoietin | TMPO | P08919 | 2 |
| ribosomal protein S17 | RPS17 | P08708 | 2 |
| neurofilament, medium polypeptide | NEFM | P07197 | 2 |
| eukaryotic translation initiation factor 5B | EIF5B | O95805 | 2 |
| structural maintenance of chromosomes 4 | SMC4 | O95752 | 2 |
| heat shock 105kDa/110kDa protein 1 | HSPH1 | O95739 | 2 |
| EPM2A (laforin) interacting protein 1 | EPM2AIP1 | O94866 | 2 |
| protein kinase D3 | PRKD3 | O94806 | 2 |
| N-terminal EF-hand calcium binding protein 2 | NECAB2 | O75547 | 2 |
| apoptotic chromatin condensation inducer 1 | ACIN1 | O75158 | 2 |
| dyskeratosis congenita 1, dyskerin | DKC1 | O60832 | 2 |
| eukaryotic translation initiation factor 4 gamma, 3 | EIF4G3 | O43432 | 2 |
| squamous cell carcinoma antigen recognized by T cells | SART1 | O43290 | 2 |
| RER1 retention in endoplasmic reticulum 1 homolog (S. cerevisiae) | RER1 | O15258 | 2 |
| tetratricopeptide repeat and ankyrin repeat containing 1 | LBA1 | O15050.4 | 2 |
| zinc finger protein 609 | ZNF609 | O15014 | 2 |
| glycogen synthase kinase 3 alpha | GSK3A | O14959 | 2 |
| paired-like homeobox 2a | PHOX2A | O14813 | 2 |
| TRAF-type zinc finger domain containing 1 | TRAFD1 | O14545 | 2 |
| protein phosphatase 1, regulatory (inhibitor) subunit 10 | PPP1R10 | O00405 | 2 |
| suppressor of Ty 5 homolog (S. cerevisiae) | SUPT5H | O00267 | 2 |
| forty-two-three domain containing 1 | FYTTD1 | C9J7P6 | 2 |
| chromosome 17 open reading frame 49 | C17orf49 | C9J4G0 | 2 |
| bone morphogenetic protein 2 | BMP2 | C8C060 | 2 |
| ARD1A protein | ARD1A | BC063377.1 | 2 |
| family with sequence similarity 62, member C | FAM62C | BC037292.1 | 2 |
| platelet derived growth factor C | PDGFC | B9EGR8 | 2 |
| chromosome 2 open reading frame 49 | C2orf49 | B3KXN3 | 2 |
| kinesin light chain 4 | KLC4 | B3KSQ3 | 2 |
| nudE nuclear distribution gene E homolog (A. nidulans)-like 1 | NDEL1 | B3KP93 | 2 |
| SWI/SNF related, matrix associated, actin dependent regulator of chromatin, subfamily a, member 4 | SMARCA4 | B3KNW7 | 2 |
| pleckstrin homology domain interacting protein | PHIP | B2RPK4 | 2 |
| NOL1/NOP2/Sun domain family, member 2 | NSUN2 | B2RNR4 | 2 |
| RNA binding motif protein 25 | RBM25 | B2RNA8 | 2 |
| NLR family, pyrin domain containing 9 | NLRP9 | B2RN12 | 2 |
| ERBB receptor feedback inhibitor 1 | ERRFI1 | B2RDX9 | 2 |
| sphingosine-1-phosphate phosphatase 1 | SGPP1 | B2RAH0 | 2 |
| matrix metallopeptidase 10 (stromelysin 2) | MMP10 | B2R9X9 | 2 |
| coilin | COIL | B2R931 | 2 |
| serine/threonine kinase 10 | STK10 | B2R8F5 | 2 |
| ribosomal protein S3 pseudogene 3; ribosomal protein S3 | RPS3 | B2R7N5 | 2 |
| lamin B receptor | LBR | B2R5P3 | 2 |
| granzyme K (granzyme 3; tryptase II) | GZMK | B2R563 | 2 |
| interferon regulatory factor 2 binding protein 2 | IRF2BP2 | B1AM36 | 2 |
| RIO kinase 3 (yeast) | RIOK3 | B0YJ89 | 2 |
| Unknown protein | pp14450 | AF318341.1 | 2 |
| doublecortin | DCX | A9Z1V8 | 2 |
| ubiquitin interaction motif containing 1 | UIMC1 | A8MSA1 | 2 |
| neural cell adhesion molecule 1 | NCAM1 | A8K8T8 | 2 |
| D4, zinc and double PHD fingers family 2 | DPF2 | A8K7C9 | 2 |
| epsin 3 | EPN3 | A8K6J3 | 2 |
| leucine rich repeat containing 41 | LRRC41 | A8K5G8 | 2 |
| leucine rich repeat containing 37A | LRRC37A | A6NMS7 | 2 |
| tolloid-like 2 | TLL2 | A6NDK0 | 2 |
| Paralemmin-3 | PALM3 | A6NDB9 | 2 |
| family with sequence similarity 54, member B | FAM54B | A6NCB4 | 2 |
| RAP1 interacting factor homolog (yeast) | RIF1 | A6NC27 | 2 |
| KIAA0408; chromosome 6 open reading frame 174 | C6orf174 | A5PLQ8 | 2 |
| stathmin 1 | STMN1 | A2A2D1 | 2 |
| cysteine-rich protein 2 | CRIP2 | A1A4U1 | 2 |
| zinc finger CCCH-type containing 13 | ZC3H13 | A0PJJ2 | 2 |
| v-yes-1 Yamaguchi sarcoma viral related oncogene homolog | LYN | A0AVQ5 | 2 |
| vomeronasal 1 receptor 5 | VN1R5 | A0AVG1 | 2 |
| hypothetical protein | DKFZp779J2370 | BX640928.1 | 2 |
