# Supplementary material for: VENNTURE–A Novel Venn Diagram Investigational Tool for Multiple Pharmacological Dataset Analysis
Source: PLoS One. 2012 May 14;7(5):e36911. doi: 10.1371/journal.pone.0036911 (PMC3351456; doi:10.1371/journal.pone.0036911)
Supplement: Table S11 — Phosphoproteins extracted from 10 µM MeCh-stimulated chronic minimal peroxide (CMP)-state human neuroblastoma SH-SY5Y cells. For each successfully identified protein official symbol, Uniprot accession code and number of peptides recovered are indicated. (DOC) [file pone.0036911.s012.doc]

**Table S11.** Phosphoproteins extracted from 10µM MeCh-stimulated chronic minimal peroxide (CMP)-state human neuroblastoma SH-SY5Y cells. For each successfully identified protein official symbol, Uniprot accession code and number of peptides recovered are indicated.

| **Protein Identification** | **Symbol** | **Accession** | **Peptide** |
| --- | --- | --- | --- |
| v-yes-1 Yamaguchi sarcoma viral related oncogene homolog | LYN | A0AVQ5 | 32 |
| U2-associated SR140 protein | SR140 | A0PJ60 | 31 |
| REX1, RNA exonuclease 1 homolog (S. cerevisiae)-like 2 (pseudogene) | REXO1L2P | A0PJM3.2 | 30 |
| cysteine-rich protein 2 | CRIP2 | A1A4U1 | 25 |
| discs, large (Drosophila) homolog-associated protein 2 | DLGAP2 | A1QCF8 | 24 |
| stathmin 1 | STMN1 | A2A2D1 | 20 |
| keratin 26 | KRT26 | A2RUL2 | 13 |
| chromosome 7 open reading frame 47 | C7orf47 | A4D2C5 | 13 |
| RAN binding protein 10 | RANBP10 | A4FTY2 | 10 |
| family with sequence similarity 54, member B | FAM54B | A6NCB4 | 10 |
| centrosome and spindle pole associated protein 1 | CSPP1 | A6ND63 | 10 |
| tolloid-like 2 | TLL2 | A6NDK0 | 10 |
| non-SMC condensin II complex, subunit D3 | NCAPD3 | A6NFS2 | 10 |
| zinc finger protein 655 | ZNF655 | A6NGD3 | 10 |
| chromosome 4 open reading frame 47 | C4orf47 | A7E2U8 | 10 |
| protein kinase, interferon-inducible double stranded RNA dependent activator | PRKRA | A8K3I6 | 9 |
| leucine rich repeat containing 41 | LRRC41 | A8K5G8 | 9 |
| D4, zinc and double PHD fingers family 2 | DPF2 | A8K7C9 | 9 |
| neural cell adhesion molecule 1 | NCAM1 | A8K8T8 | 9 |
| cat eye syndrome chromosome region, candidate 2 | CECR2 | A8MS90 | 8 |
| ubiquitin fusion degradation 1 like (yeast) | UFD1L | A8MW31 | 8 |
| doublecortin | DCX | A9Z1V8 | 8 |
| Ku86 autoantigen related protein 1 | KARP-1 | AAC52087.1 | 8 |
| PEX26T35insC protein | PEX26T35insC | AB103104.1 | 7 |
| Unknown protein | pp10472 | AF318321.1 | 7 |
| Unknown protein | pp10472 | AF318321.1 | 7 |
| family with sequence similarity 178, member A | FAM178A | B1AL17 | 7 |
| interferon regulatory factor 2 binding protein 2 | IRF2BP2 | B1AM36 | 7 |
| adenosine deaminase, RNA-specific | ADAR | B1AQQ9 | 7 |
| DEAD (Asp-Glu-Ala-Asp) box polypeptide 23 | DDX23 | B2R600 | 6 |
| ribosomal protein S3 pseudogene 3; ribosomal protein S3 | RPS3 | B2R7N5 | 6 |
| dickkopf homolog 1 (Xenopus laevis) | DKK1 | B2RC19 | 6 |
| RNA binding motif protein 25 | RBM25 | B2RNA8 | 6 |
| pleckstrin homology domain interacting protein | PHIP | B2RPK4 | 6 |
| family with sequence similarity 110, member B | FAM110B | B3KRT5 | 6 |
| cofilin 1 (non-muscle) | CFL1 | B3KUQ1 | 6 |
| mucin 16, cell surface associated | MUC16 | B3KY81 | 6 |
| sciellin | SCEL | B7Z797 | 6 |
| coiled-coil domain containing 142 | CCDC142 | B7ZKV5 | 6 |
| potassium channel, subfamily T, member 1 | KCNT1 | B7ZVY4 | 6 |
| chromosome 17 open reading frame 49 | C17orf49 | C9J4G0 | 6 |
| hCG_2015407 | hCG_2015407 | CH471054.1 | 6 |
| hCG_2044975 | hCG_2044975 | CH471066.2 | 6 |
| hCG_2026193 | hCG_2026193 | CH471083.1 | 6 |
| Hypothetical protein | DKFZp686K09128 | CR749605.1 | 6 |
| suppressor of Ty 5 homolog (S. cerevisiae) | SUPT5H | O00267 | 5 |
| protein phosphatase 1, regulatory (inhibitor) subunit 10 | PPP1R10 | O00405 | 5 |
| phosphoinositide-3-kinase, class 2, alpha polypeptide | PIK3C2A | O00443 | 5 |
| TRAF-type zinc finger domain containing 1 | TRAFD1 | O14545 | 5 |
| paired-like homeobox 2a | PHOX2A | O14813 | 5 |
| glycogen synthase kinase 3 alpha | GSK3A | O14959 | 5 |
| zinc finger protein 609 | ZNF609 | O15014 | 5 |
| API5-like 1; apoptosis inhibitor 5 | API5 | O15441 | 5 |
| squamous cell carcinoma antigen recognized by T cells | SART1 | O43290 | 5 |
| heat shock 70kDa protein 12A | HSPA12A | O43301 | 5 |
| hypocretin (orexin) receptor 2 | HCRTR2 | O43614 | 5 |
| asparaginyl-tRNA synthetase | NARS | O43776 | 5 |
| zinc finger protein 73 | ZNF73 | O43830.1 | 5 |
| GTPase activating protein (SH3 domain) binding protein 2 | G3BP2 | O60606 | 5 |
| dyskeratosis congenita 1, dyskerin | DKC1 | O60832 | 5 |
| apoptotic chromatin condensation inducer 1 | ACIN1 | O75158 | 5 |
| arachidonate 12-lipoxygenase, 12R type | ALOX12B | O75342 | 5 |
| tripartite motif-containing 3 | TRIM3 | O75382 | 5 |
| unc-51-like kinase 1 (C. elegans) | ULK1 | O75385 | 5 |
| protein kinase D3 | PRKD3 | O94806 | 5 |
| kelch repeat and BTB (POZ) domain containing 11 | KBTBD11 | O94819 | 5 |
| EPM2A (laforin) interacting protein 1 | EPM2AIP1 | O94866 | 4 |
| MYST histone acetyltransferase 2; similar to MYST histone acetyltransferase 2 | MYST2 | O95251 | 4 |
| structural maintenance of chromosomes 4 | SMC4 | O95752 | 4 |
| eukaryotic translation initiation factor 5B | EIF5B | O95805 | 4 |
| p21 protein (Cdc42/Rac)-activated kinase 4 | PAK4 | O96013 | 4 |
| neurofilament, medium polypeptide | NEFM | P07197 | 4 |
| ribosomal protein S17 | RPS17 | P08708 | 4 |
| thymopoietin | TMPO | P08919 | 4 |
| microtubule-associated protein tau | MAPT | P10636 | 4 |
| translocated promoter region (to activated MET oncogene) | TPR | P12270 | 4 |
| heterogeneous nuclear ribonucleoprotein C (C1/C2) | HNRNPC | P22628 | 4 |
| transcription elongation factor A (SII), 1 pseudogene 2; transcription elongation factor A (SII), 1 | TCEA1 | P23193 | 4 |
| ribosomal protein L23a pseudogene 63 | RPL23A | P29316 | 4 |
| NOP2 nucleolar protein homolog (yeast) | NOP2 | P46087 | 4 |
| ATP citrate lyase | ACLY | P53396 | 4 |
| glycoprotein 2 (zymogen granule membrane) | GP2 | P55259 | 4 |
| TPI1 pseudogene; triosephosphate isomerase 1 | TPI1 | P60174 | 4 |
| tubulin, alpha 4a | TUBA4A | P68366 | 4 |
| nitrogen permease regulator-like 3 (S. cerevisiae) | MARE | P78384 | 4 |
| brain abundant, membrane attached signal protein 1 | BASP1 | P80723 | 4 |
| chromobox homolog 1 (HP1 beta homolog Drosophila ) | CBX1 | P83916 | 4 |
| PCTAIRE protein kinase 1 | PCTK1 | Q00536 | 4 |
| glutamyl-prolyl-tRNA synthetase | EPRS | Q05BP6 | 3 |
| host cell factor C1 (VP16-accessory protein) | HCFC1 | Q05C05 | 3 |
| Dmx-like 1 | DMXL1 | Q05C95 | 3 |
| NFKB activating protein | NKAP | Q05D22 | 3 |
| matrix-remodelling associated 7 | MXRA7 | Q0P5W3 | 3 |
| interleukin enhancer binding factor 3, 90kDa | ILF3 | Q12906 | 3 |
| chromatin assembly factor 1, subunit B (p60) | CHAF1B | Q13112 | 3 |
| proteasome (prosome, macropain) 26S subunit, non-ATPase, 2 | PSMD2 | Q13200 | 3 |
| nuclear factor of activated T-cells, cytoplasmic, calcineurin-dependent 2 | NFATC2 | Q13469 | 3 |
| caldesmon 1 | CALD1 | Q13979 | 3 |
| heterogeneous nuclear ribonucleoprotein D (AU-rich element RNA binding protein 1, 37kDa) | HNRNPD | Q14100 | 3 |
| transcription factor 3 (E2A immunoglobulin enhancer binding factors E12/E47) | TCF3 | Q14208 | 3 |
| similar to RNA binding motif protein 39; RNA binding motif protein 39 | RBM39 | Q14498 | 3 |
| sodium channel, voltage-gated, type V, alpha subunit | Nav1.5 | Q14524.2 | 3 |
| phosphoprotein enriched in astrocytes 15 | PEA15 | Q14801 | 3 |
| poly(rC) binding protein 1 | PCBP1 | Q14975 | 3 |
| hypothetical LOC728026; prothymosin, alpha; hypothetical gene supported by BC013859 | PTMAP4 | Q15204 | 3 |
| non-POU domain containing, octamer-binding | NONO | Q15233 | 3 |
| telomeric repeat binding factor 2 | TERF2 | Q15554 | 3 |
| protein tyrosine phosphatase, receptor type, S | PTPRS | Q15718 | 3 |
| fibrillin 1 | FBN1 | Q15972 | 3 |
| adducin 1 (alpha) | ADD1 | Q16156 | 3 |
| ELAV (embryonic lethal, abnormal vision, Drosophila)-like 4 (Hu antigen D) | ELAVL4 | Q16234 | 3 |
| KIAA0528 | KIAA0528 | Q17RY7 | 3 |
| YY1 associated protein 1; gon-4-like (C. elegans) | GON4L | Q1ED43 | 3 |
| LIM domain and actin binding 1 | LIMA1 | Q2TAN7 | 3 |
| heparan sulfate proteoglycan 2 | HSPG2 | Q2VPA1 | 3 |
| nestin | NES | Q2YDX4 | 3 |
| calcium regulated heat stable protein 1, 24kDa | CARHSP1 | Q2YDX5 | 3 |
| zinc finger and BTB domain containing 49 | ZNF509 | Q32MK9 | 3 |
| protein kinase, cAMP-dependent, catalytic, alpha | PRKACA | Q32P54 | 3 |
| HECT, UBA and WWE domain containing 1 | HUWE1 | Q3B7K0 | 3 |
| ubiquitin specific peptidase 42 | USP42 | Q3C166 | 3 |
| reticulon 4 | RTN4 | Q3LIF4 | 3 |
| heterogeneous nuclear ribonucleoprotein A1-like 3 | HNRPA1L3 | Q3MI39 | 3 |
| carbohydrate (chondroitin 4) sulfotransferase 13 | CHST13 | Q3SYA5 | 3 |
| Cdon homolog (mouse) | CDON | Q4KMG0 | 3 |
| death inducer-obliterator 1 | DIDO1 | Q4VXS2 | 3 |
| protein tyrosine phosphatase, non-receptor type 13 (APO-1/CD95 (Fas)-associated phosphatase) | PTPN13 | Q4W5F5 | 2 |
| SWI/SNF related, matrix associated, actin dependent regulator of chromatin, subfamily a, member 5 | SMARCA5 | Q4W5G3 | 2 |
| zinc finger protein 141 | ZNF141 | Q4W5N2 | 2 |
| AP2 associated kinase 1 | AAK1 | Q4ZFZ3 | 2 |
| splicing factor, arginine/serine-rich 9 | SFRS9 | Q52LD1 | 2 |
| LIM homeobox 2 | LHX2 | Q52M57 | 2 |
| RNA binding motif protein 14; RNA binding motif protein 4 | RBM4 | Q53GV1 | 2 |
| Rho GTPase activating protein 25 | ARHGAP25 | Q53QF7 | 2 |
| general transcription factor IIIC, polypeptide 2, beta 110kDa | GTF3C2 | Q53QN0 | 2 |
| potassium voltage-gated channel, subfamily H (eag-related), member 7 | KCNH7 | Q53QU4 | 2 |
| ArfGAP with FG repeats 1 | AGFG1 | Q53R11 | 2 |
| spectrin, beta, non-erythrocytic 1 | SPTBN1 | Q53R99 | 2 |
| contactin associated protein-like 5 | CNTNAP5 | Q53RX1 | 2 |
| activating transcription factor 2 | ATF2 | Q53RY2 | 2 |
| thyroid hormone receptor interactor 12 | TRIP12 | Q53TE7 | 2 |
| solute carrier family 31 (copper transporters), member 2 | SLC31A2 | Q53X94 | 2 |
| galactosidase, alpha | GLA | Q53Y83 | 2 |
| heat shock protein 90kDa alpha (cytosolic), class B member 2 (pseudogene) | HSP90AB2P | Q58FF8 | 2 |
| mutS homolog 5 (E. coli) | MSH5 | Q59EC5 | 2 |
| insulin-like growth factor 2 receptor | IGF2R | Q59EZ3 | 2 |
| N-glycanase 1 | NGLY1 | Q59FB1 | 2 |
| drebrin-like | DBNL | Q59FH4 | 2 |
| cyclin K | CCNK | Q59FT6 | 2 |
| exosome component 10 | EXOSC10 | Q59G73 | 2 |
| KIAA0182 protein | GSE1 | Q59GZ0 | 2 |
| crumbs homolog 1 (Drosophila) | CRB1 | Q59H36 | 2 |
| topoisomerase (DNA) II beta 180kDa | TOP2B | Q59H80 | 2 |
| sorbin and SH3 domain containing 3 | SORBS3 | Q5BJE4 | 2 |
| matrin 3 | MATR3 | Q5CZA7 | 2 |
| family with sequence similarity 76, member B | FAM76B | Q5HYJ3 | 2 |
| lamin A/C | LMNA | Q5I6Y6 | 2 |
| mitogen-activated protein kinase kinase kinase 15 | MAP3K15 | Q5JPR4 | 2 |
| FERM, RhoGEF (ARHGEF) and pleckstrin domain protein 1 (chondrocyte-derived) | FARP1 | Q5JV94 | 2 |
| karyopherin alpha 3 (importin alpha 4) | KPNA3 | Q5JVN1 | 2 |
| septin 7 | 40793 | Q5JXL7 | 2 |
| RNA binding motif protein, X-linked 2 | RBMX2 | Q5JY82 | 2 |
| serum response factor binding protein 1 | SRFBP1 | Q5QFI2 | 2 |
| patched homolog 2 (Drosophila) | PTCH2 | Q5QP87 | 2 |
| death-domain associated protein | DAXX | Q5STR5 | 2 |
| hepatoma-derived growth factor (high-mobility group protein 1-like) | HDGF | Q5SZ07 | 2 |
| DENN/MADD domain containing 1B | DENND1B | Q5T3B9 | 2 |
| nucleoporin 153kDa | NUP153 | Q5T9I7 | 2 |
| nucleolar protein 8 | NOL8 | Q5TCD7 | 2 |
| AT hook, DNA binding motif, containing 1 | AHDC1 | Q5TGY4 | 2 |
| myeloid leukemia factor 2 | MLF2 | Q5U0N1 | 2 |
| GTPase activating protein (SH3 domain) binding protein 1 | G3BP1 | Q5U0Q1 | 2 |
| wings apart-like homolog (Drosophila) | WAPAL | Q5VSK5 | 2 |
| serine/arginine repetitive matrix 1 | SRRM1 | Q5VVN4 | 2 |
| antigen identified by monoclonal antibody Ki-67 | MKI67 | Q5VWH2 | 2 |
| ubiquitin-like with PHD and ring finger domains 2 | UHRF2 | Q5VYR1 | 2 |
| zinc finger, MYM-type 4 | ZMYM4 | Q5VZL5 | 2 |
| ribonucleotide reductase M2 polypeptide | RRM2 | Q5WRU7 | 2 |
| heterogeneous nuclear ribonucleoprotein H1 (H) | HNRNPH1 | Q68DG4 | 2 |
| ATP-binding cassette, sub-family F (GCN20), member 1 | ABCF1 | Q69YP6 | 2 |
| epidermal growth factor receptor pathway substrate 15-like 1 | EPS15L1 | Q69YZ4 | 2 |
| IGF-like family member 2 | IGFL2 | Q6B9Z3 | 2 |
| similar to Bcl-2-associated transcription factor 1 (Btf); BCL2-associated transcription factor 1 | BCLAF1 | Q6DCA8 | 2 |
| jun oncogene | JUN | Q6FHM7 | 2 |
| gap junction protein, alpha 1, 43kDa | GJA1 | Q6FHU1 | 2 |
| ADAM metallopeptidase domain 20 | ADAM20 | Q6GTZ1 | 2 |
| mitogen-activated protein kinase associated protein 1 | MAPKAP1 | Q6GVJ2 | 2 |
| eukaryotic translation initiation factor 3, subunit G | EIF3G | Q6IAM0 | 2 |
| suppressor of Ty 7 (S. cerevisiae)-like | SUPT7L | Q6IB21 | 2 |
| heterogeneous nuclear ribonucleoprotein K; similar to heterogeneous nuclear ribonucleoprotein K | HNRNPK | Q6IBN1 | 2 |
| 3-oxoacid CoA transferase 2 | OXCT2 | Q6INA3 | 2 |
| chromosome 9 open reading frame 82 | C9orf82 | Q6IPE6 | 2 |
| BRF1 homolog, subunit of RNA polymerase III transcription initiation factor IIIB (S. cerevisiae) | BRF1 | Q6IQ02 | 2 |
| WD repeat domain 77 | WDR77 | Q6JZZ5 | 2 |
| G protein-coupled receptor 126 | GPR126 | Q6MZU7 | 2 |
| LIM and calponin homology domains 1 | LIMCH1 | Q6N054 | 2 |
| trinucleotide repeat containing 6A | TNRC6A | Q6NVB5 | 2 |
| myristoylated alanine-rich protein kinase C substrate | MARCKS | Q6NVI1 | 2 |
| MARCKS-like 1 | MARCKSL1 | Q6NXS5 | 2 |
| thyroid hormone receptor associated protein 3 | THRAP3 | Q6P0P7 | 2 |
| PC4 and SFRS1 interacting protein 1 | PSIP1 | Q6P391 | 2 |
| MYC binding protein 2 | MYCBP2 | Q6PIB6 | 2 |
| microtubule-associated protein 1B | MAP1B | Q6PJD3 | 2 |
| KH domain containing, RNA binding, signal transduction associated 1 | KHDRBS1 | Q6PJX7 | 2 |
| splicing factor, arginine/serine-rich 11 | SFRS11 | Q6PJY9 | 2 |
| estrogen receptor binding site associated, antigen, 9 | EBAG9 | Q6R3F1 | 2 |
| ring finger and SPRY domain containing 1 | RSPRY1 | Q6UX21 | 2 |
| major histocompatibility complex, class I, C; major histocompatibility complex, class I, B | HLA-C | Q6V4Z4 | 2 |
| BTB (POZ) domain containing 8 | BTBD8 | Q6V9S5 | 2 |
| GRB10 interacting GYF protein 2 | GIGYF2 | Q6Y7W6 | 2 |
| zinc finger protein 841 | ZNF841 | Q6ZN82 | 2 |
| hypothetical LOC100130009; high mobility group AT-hook 1 | HMGA1 | Q6ZP45 | 2 |
| RUN and FYVE domain containing 4 | RUFY4 | Q6ZR96 | 2 |
| formin 1 | FMN1 | Q6ZSY1 | 2 |
| zinc finger, C3H1-type containing | ZFC3H1 | Q6ZV36 | 2 |
| tankyrase 1 binding protein 1, 182kDa | TNKS1BP1 | Q6ZV74 | 2 |
| RNA binding motif protein 6 | RBM6 | Q6ZVV4 | 2 |
| diacylglycerol kinase, zeta 104kDa | DGKZ | Q6ZWA5 | 2 |
| signal peptide, CUB domain, EGF-like 2 | SCUBE2 | Q6ZWI1 | 2 |
| potassium channel tetramerisation domain containing 1 | KCTD1 | Q719H9 | 2 |
| synaptopodin | SYNPO | Q71HJ6 | 2 |
| RNA binding motif protein 33 | RBM33 | Q75ML5 | 2 |
| DBF4 homolog (S. cerevisiae) | DBF4 | Q75MS6 | 2 |
| coiled-coil domain containing 132 | CCDC132 | Q75N11 | 2 |
| zinc finger and AT hook domain containing | ZFAT | Q75PJ7 | 2 |
| nucleoporin 214kDa | NUP214 | Q75R47 | 2 |
| cortactin | CTTN | Q76MU0 | 2 |
| pinin, desmosome associated protein | PNN | Q7KYL1 | 2 |
| MAP7 domain containing 1 | MAP7D1 | Q7L8J5 | 2 |
| oligodendrocyte transcription factor 3 | OLIG3 | Q7RTU3 | 2 |
| mitogen-activated protein kinase kinase 2 pseudogene; mitogen-activated protein kinase kinase 2 | MAP2K2 | Q7Z370 | 2 |
| methyl CpG binding protein 2 (Rett syndrome) | MECP2 | Q7Z384 | 2 |
| ankyrin 2, neuronal | ANK2 | Q7Z3L5 | 2 |
| tumor protein p53 binding protein 1 | TP53BP1 | Q7Z3U4 | 2 |
| piwi-like 3 (Drosophila) | PIWIL3 | Q7Z3Z3 | 2 |
| eukaryotic translation initiation factor 2A, 65kDa | EIF2A | Q7Z4E9 | 2 |
| NADH dehydrogenase (ubiquinone) 1 alpha subcomplex, 10, 42kDa | NDUFA10 | Q7Z518 | 2 |
| immunoglobulin-like domain containing receptor 1 | ILDR1 | Q7Z578 | 2 |
| sphingosine-1-phosphate receptor 3 | S1PR3 | Q7Z5I2 | 2 |
| tripartite motif-containing 28 | TRIM28 | Q7Z632 | 2 |
| kelch repeat and BTB (POZ) domain containing 12 | KLHDC6 | Q7Z665 | 2 |
| kinesin family member 21A | KIF21A | Q7Z668 | 2 |
| hypothetical protein LOC387763 | AG2 | Q7Z7L8 | 2 |
| taxilin alpha | TXLNA | Q86T86 | 2 |
| pleckstrin homology domain containing, family A (phosphoinositide binding specific) member 3 | PLEKHA3 | Q86TQ1 | 2 |
| bromodomain adjacent to zinc finger domain, 1B | BAZ1B | Q86UJ6 | 2 |
| euchromatic histone-lysine N-methyltransferase 1 | EHMT1 | Q86X08 | 2 |
| chromosome 17 open reading frame 82 | C17orf82 | Q86X59 | 2 |
| regulation of nuclear pre-mRNA domain containing 2 | RPRD2 | Q86XD2 | 2 |
| growth arrest-specific 2 like 3 | GAS2L3 | Q86XJ1 | 2 |
| microtubule-associated protein 4 | MAP4 | Q86Y04 | 2 |
| DEAD (Asp-Glu-Ala-Asp) box polypeptide 54 | DDX54 | Q86YT8 | 2 |
| EPH receptor A8 | EPHA8 | Q8IUX6 | 2 |
| DEAD (Asp-Glu-Ala-Asp) box polypeptide 6 | DDX6 | Q8IV96 | 2 |
| FCH domain only 1 | FCHO1 | Q8IW22 | 2 |
| glutamate receptor, ionotropic, N-methyl D-aspartate 2C | GRIN2C | Q8IW23 | 2 |
| DEAD (Asp-Glu-Ala-Asp) box polypeptide 51 | DDX51 | Q8IXK5 | 2 |
| chromodomain helicase DNA binding protein 4 | CHD4 | Q8IXZ5 | 2 |
| suppressor of var1, 3-like 1 (S. cerevisiae) | SUPV3L1 | Q8IYB8 | 2 |
| cartilage intermediate layer protein, nucleotide pyrophosphohydrolase | CILP | Q8IYI5 | 2 |
| family with sequence similarity 154, member A | FAM154A | Q8IYX7 | 2 |
| zinc finger protein 683 | ZNF683 | Q8IZ20 | 2 |
| zinc finger protein 687 | ZNF687 | Q8N1G0 | 2 |
| Rho-guanine nucleotide exchange factor | RGNEF | Q8N1W1 | 2 |
| zinc finger, C3HC-type containing 1 | ZC3HC1 | Q8N330 | 2 |
| SEC16 homolog A (S. cerevisiae) | SEC16A | Q8N347 | 2 |
| YSK4 Sps1/Ste20-related kinase homolog (S. cerevisiae) | YSK4 | Q8N4E9 | 2 |
| chromosome 1 open reading frame 92 | C1orf92 | Q8N4P6 | 2 |
| collagen, type VI, alpha 3 | COL6A3 | Q8N4Z1 | 2 |
| ARP8 actin-related protein 8 homolog (yeast) | ACTR8 | Q8N566 | 2 |
| chromosome 6 open reading frame 223 | C6orf223 | Q8N575 | 2 |
| cytoplasmic linker associated protein 1 | CLASP1 | Q8N5B8 | 2 |
| elastin microfibril interfacer 2 | EMILIN2 | Q8N5L1 | 2 |
| hypothetical MGC50722 | MGC50722 | Q8N5P7 | 2 |
| lactation elevated 1 | LACE1 | Q8N6A3 | 2 |
| similar to RNA binding motif protein, X-linked; similar to hCG2011544; RNA binding motif protein, X-linked | RBMX | Q8N8Y7 | 2 |
| centrosomal protein 120kDa | CCDC100 | Q8N960.2 | 2 |
| LSM11, U7 small nuclear RNA associated | LSM11 | Q8N975 | 2 |
| dihydropyrimidinase-like 2 | DPYSL2 | Q8NAN9 | 2 |
| tudor domain containing 5 | TDRD5 | Q8NAT2 | 2 |
| PRP38 pre-mRNA processing factor 38 (yeast) domain containing A | PRPF38A | Q8NAV1 | 2 |
| nucleolin | NCL | Q8NB06 | 2 |
| pygopus homolog 2 (Drosophila) | PYGO2 | Q8NBG9 | 2 |
| solute carrier family 35, member E1 | SLC35E1 | Q8NBQ2 | 2 |
| death effector domain containing 2 | DEDD2 | Q8NBR2 | 2 |
| HEG homolog 1 (zebrafish) | HEG1 | Q8NC40 | 2 |
| protein interacting with cyclin A1 | PROCA1 | Q8NCQ7 | 2 |
| chromosome 14 open reading frame 48 | C14orf48 | Q8NCU1 | 2 |
| ankyrin repeat domain 17 | ANKRD17 | Q8NDR5 | 2 |
| catenin (cadherin-associated protein), beta 1, 88kDa | CTNNB1 | Q8NEW9 | 2 |
| olfactory receptor, family 5, subfamily AR, member 1 | OR5AR1 | Q8NGP9 | 2 |
| sphingomyelin synthase 2 | SGMS2 | Q8NHU3 | 2 |
| WD repeat domain 43 | WDR43 | Q8TB67 | 2 |
| prospero homeobox 1 | PROX1 | Q8TB91 | 2 |
| glutamate receptor, metabotropic 3 | GRM3 | Q8TBH9 | 2 |
| cytochrome P450, family 19, subfamily A, polypeptide 1 | CYP19A1 | Q8TCA4 | 2 |
| mutS homolog 6 (E. coli) | MSH6 | Q8TCX4 | 2 |
| dynein, axonemal, heavy chain 1 | DNAH1 | Q8TEJ4 | 2 |
| peptidylprolyl isomerase (cyclophilin)-like 4 | PPIL4 | Q8WUA2 | 2 |
| kinesin family member 23 | KIF23 | Q8WVP0 | 2 |
| Fc receptor, IgA, IgM, high affinity | FCAMR | Q8WWV6 | 2 |
| centrosomal protein 350kDa | CEP350 | Q8WY20 | 2 |
| telomeric repeat binding factor 2, interacting protein | TERF2IP | Q8WYZ3 | 2 |
| damage-specific DNA binding protein 2, 48kDa | DDB2 | Q92466 | 2 |
| H1 histone family, member X | H1FX | Q92522 | 2 |
| TBC1 domain family, member 5 | TBC1D5 | Q92609 | 2 |
| bromodomain containing 3 | BRD3 | Q92645 | 2 |
| bridging integrator 1 | BIN1 | Q92944 | 2 |
| SECIS binding protein 2-like | KIAA0256 | Q93073.3 | 2 |
| ATPase, class V, type 10A | ATP10A | Q969I4 | 2 |
| eukaryotic translation elongation factor 1 delta (guanine nucleotide exchange protein) | EEF1D | Q969J1 | 2 |
| minichromosome maintenance complex component 2 | MCM2 | Q969W7 | 2 |
| vacuolar protein sorting 26 homolog B (S. pombe) | VPS26B | Q96A55 | 2 |
| transmembrane protein 86A | TMEM86A | Q96AJ0 | 2 |
| scribbled homolog (Drosophila) | SCRIB | Q96C69 | 2 |
| septin 2 | 40788 | Q96CB0 | 2 |
| serine/threonine kinase 11 interacting protein | STK11IP | Q96CN3 | 2 |
| coiled-coil domain containing 124 | CCDC124 | Q96CT7 | 2 |
| C-type lectin domain family 7, member A | CLEC7A | Q96D32 | 2 |
| ligase III, DNA, ATP-dependent | LIG3 | Q96DF0 | 2 |
| zinc finger CCCH-type containing 18 | ZC3H18 | Q96DG4 | 2 |
| chromosome 3 open reading frame 54 | C3orf54 | Q96EL1 | 2 |
| cofactor of BRCA1 | COBRA1 | Q96EW5 | 2 |
| cytoplasmic linker associated protein 2 | CLASP2 | Q96F87 | 2 |
| zinc finger with KRAB and SCAN domains 1 | ZKSCAN1 | Q96FA2 | 2 |
| glucocorticoid induced transcript 1 | GLCCI1 | Q96FD0 | 2 |
| leucine-rich repeats and WD repeat domain containing 1 | LRWD1 | Q96GJ2 | 2 |
| SWI/SNF related, matrix associated, actin dependent regulator of chromatin, subfamily c, member 2 | SMARCC2 | Q96GY4 | 2 |
| microtubule-actin crosslinking factor 1 | MACF1 | Q96IQ1 | 2 |
| zinc finger protein 828 | ZNF828 | Q96JM3 | 2 |
| chromosome 12 open reading frame 42 | C12orf42 | Q96LP6 | 2 |
| beta-1,4-N-acetyl-galactosaminyl transferase 4 | B4GALNT4 | Q96LV2 | 2 |
| similar to dynein cytoplasmic 1 intermediate chain 2; dynein, cytoplasmic 1, intermediate chain 2 | DYNC1I2 | Q96NG7 | 2 |
| H2A histone family, member B1 | H2AFB1 | Q96PR7 | 2 |
| G protein regulated inducer of neurite outgrowth 1 | GPRIN1 | Q96PZ4 | 2 |
| insulinoma-associated 2 | INSM2 | Q96Q84 | 2 |
| checkpoint with forkhead and ring finger domains | CHFR | Q96SL3 | 2 |
| family with sequence similarity 40, member A | FAM40A | Q96SN2 | 2 |
| protein tyrosine phosphatase-like A domain containing 1 | PTPLAD1 | Q96T12 | 2 |
| remodeling and spacing factor 1 | RSF1 | Q96T23 | 2 |
| AT rich interactive domain 1A (SWI-like) | ARID1A | Q96T89 | 2 |
| myosin, heavy chain 9, non-muscle | MYH9 | Q99529 | 2 |
| HIV-1 Tat specific factor 1 | HTATSF1 | Q99730 | 2 |
| A kinase (PRKA) anchor protein 12 | AKAP12 | Q99970 | 2 |
| microtubule-associated protein 2 | MAP2 | Q99976 | 2 |
| splicing factor, arginine/serine-rich 2B | SFRS2B | Q9BRL6 | 2 |
| metastasis associated 1 | MTA1 | Q9BRL8 | 2 |
| anaphase promoting complex subunit 1; similar to anaphase promoting complex subunit 1 | ANAPC1 | Q9BSE6 | 2 |
| ubiquitin-conjugating enzyme E2O | UBE2O | Q9BSW1 | 2 |
| potassium voltage-gated channel, subfamily H (eag-related), member 2 | KCNH2 | Q9BT72 | 2 |
| neural proliferation, differentiation and control, 1 | NPDC1 | Q9BTD6 | 2 |
| single stranded DNA binding protein 3; hypothetical LOC100131851 | SSBP3 | Q9BTM0 | 2 |
| chromosome 11 open reading frame 84 | C11orf84 | Q9BUA3 | 2 |
| transgelin 2 | TAGLN2 | Q9BUH5 | 2 |
| phosphatidylserine synthase 2 | PTDSS2 | Q9BVG9 | 2 |
| HIRA interacting protein 3 | HIRIP3 | Q9BW71 | 2 |
| TRAF interacting protein | TRAIP | Q9BWF2 | 2 |
| thioredoxin interacting protein | TXNIP | Q9BXG9 | 2 |
| tripartite motif-containing 29 | TRIM29 | Q9BZY7 | 2 |
| dedicator of cytokinesis 7 | DOCK7 | Q9C092 | 2 |
| tet oncogene 1 | TET1 | Q9C0I7 | 2 |
| 5'-3' exoribonuclease 2 | XRN2 | Q9H0D6 | 2 |
| phosphoglucomutase 1 | PGM1 | Q9H1D2 | 2 |
| nuclear casein kinase and cyclin-dependent kinase substrate 1 | NUCKS1 | Q9H1E3 | 2 |
| SAPS domain family, member 3 | SAPS3 | Q9H2K6 | 2 |
| hematological and neurological expressed 1 | HN1 | Q9H3K0 | 2 |
| SAM domain and HD domain 1 | SAMHD1 | Q9H3U9 | 2 |
| DnaJ (Hsp40) homolog, subfamily C, member 5 | DNAJC5 | Q9H3Z5 | 2 |
| histidyl-tRNA synthetase 2, mitochondrial (putative); D-tyrosyl-tRNA deacylase 1 homolog (S. cerevisiae) | HARS2 | Q9H464 | 2 |
| pericentriolar material 1 | PCM1 | Q9H4A2 | 2 |
| centromere protein K | CENPK | Q9H4L0 | 2 |
| mitochondrial antiviral signaling protein | MAVS | Q9H4Y1 | 2 |
| retinoblastoma binding protein 6 | RBBP6 | Q9H5M5 | 2 |
| coiled-coil domain containing 86 | CCDC86 | Q9H6F5 | 2 |
| transforming growth factor, beta receptor associated protein 1 | TGFBRAP1 | Q9H6G8 | 2 |
| ring finger protein 34 | RNF34 | Q9H6W8 | 2 |
| erythrocyte membrane protein band 4.1 like 4B | EPB41L4B | Q9H709 | 2 |
| NKF3 kinase family member | SGK269 | Q9H792 | 2 |
| solute carrier family 35, member F5 | SLC35F5 | Q9H7D8 | 2 |
| arginine/serine-rich coiled-coil 2 | RSRC2 | Q9H864 | 2 |
| kinesin family member 9 | KIF9 | Q9H8A4 | 2 |
| myelin expression factor 2 | MYEF2 | Q9H922 | 2 |
| RAB24, member RAS oncogene family | RAB24 | Q9HAG2 | 2 |
| STIP1 homology and U-box containing protein 1 | STUB1 | Q9HBT1 | 2 |
| otoferlin | OTOF | Q9HC10 | 2 |
| peter pan homolog (Drosophila) | PPAN | Q9NQ55 | 2 |
| exosome component 5 | EXOSC5 | Q9NQT4 | 2 |
| DEAD (Asp-Glu-Ala-Asp) box polypeptide 21 | DDX21 | Q9NR30 | 2 |
| eukaryotic translation initiation factor 4E nuclear import factor 1 | EIF4ENIF1 | Q9NRA8 | 2 |
| centrosomal protein 170kDa | CEP170 | Q9NSN9 | 2 |
| Bardet-Biedl syndrome 7 | BBS7 | Q9NVI4 | 2 |
| anillin, actin binding protein | ANLN | Q9NVP0 | 2 |
| trichorhinophalangeal syndrome I | TRPS1 | Q9NWE1 | 2 |
| SAFB-like, transcription modulator | SLTM | Q9NWH9 | 2 |
| periphilin 1 | PPHLN1 | Q9NXL4 | 2 |
| kinesin family member 4B; kinesin family member 4A | KIF4A | Q9NY24 | 2 |
| serine/arginine repetitive matrix 2; hypothetical LOC100132779 | SRRM2 | Q9P0G1 | 2 |
| Rho guanine nucleotide exchange factor (GEF) 12 | ARHGEF12 | Q9P149 | 2 |
| similar to ABT1-associated protein; ESF1, nucleolar pre-rRNA processing protein, homolog (S. cerevisiae) | ESF1 | Q9P1S6 | 2 |
| KIAA1539 | KIAA1539 | Q9P1Y9 | 2 |
| syntaxin 18 | STX18 | Q9P2W9 | 2 |
| heat shock 27kDa protein-like 2 pseudogene; heat shock 27kDa protein 1 | HSPB1 | Q9UC31 | 2 |
| transcription factor CP2 | TFCP2 | Q9UD75 | 2 |
| similar to hCG1820375; PRP4 pre-mRNA processing factor 4 homolog B (yeast) | PRPF4B | Q9UEE6 | 2 |
| Fc fragment of IgA, receptor for | FCAR | Q9UEK0 | 2 |
| Treacher Collins-Franceschetti syndrome 1 | TCOF1 | Q9UFD4 | 2 |
| RAB11 family interacting protein 5 (class I) | RAB11FIP5 | Q9UFM0 | 2 |
| G-protein signaling modulator 1 (AGS3-like, C. elegans) | GPSM1 | Q9UFS8 | 2 |
| drebrin 1 | DBN1 | Q9UFZ5 | 2 |
| like-glycosyltransferase | LARGE | Q9UGG3 | 2 |
| progesterone receptor membrane component 1 | PGRMC1 | Q9UGJ9 | 2 |
| ubiquitin associated protein 2-like | UBAP2L | Q9UGL5 | 2 |
| transcription factor 20 (AR1) | TCF20 | Q9UGU0 | 2 |
| nucleoporin 98kDa | NUP98 | Q9UHX0 | 2 |
| cyclin L1 | CCNL1 | Q9UK58 | 2 |
| synaptopodin 2 | SYNPO2 | Q9UK89 | 2 |
| CDC42 effector protein (Rho GTPase binding) 3 | CDC42EP3 | Q9UKI2 | 2 |
| G patch domain containing 8 | GPATCH8 | Q9UKJ3 | 2 |
| SON DNA binding protein | SON | Q9UKP9 | 2 |
| nucleoporin 50kDa | NUP50 | Q9UKX7 | 2 |
| microtubule-associated protein 1A | MAP1A | Q9UL09 | 2 |
| zinc finger protein 644 | ZNF644 | Q9ULJ9 | 2 |
| vang-like 2 (van gogh, Drosophila) | VANGL2 | Q9ULK5 | 2 |
| KIAA1211 | KIAA1211 | Q9ULK9 | 2 |
| GATA zinc finger domain containing 2B | GATAD2B | Q9ULS0 | 2 |
| apolipoprotein B (including Ag(x) antigen) | APOB | Q9UMN0 | 2 |
| nuclear mitotic apparatus protein 1 | NUMA1 | Q9UNL7 | 2 |
| regulator of G-protein signaling 7 | RGS7 | Q9UNU7 | 2 |
| CD22 molecule | CD22 | Q9UQB1 | 2 |
| GRB2-associated binding protein 2 | GAB2 | Q9UQC2 | 2 |
| PDZ and LIM domain 4 | PDLIM4 | Q9Y292 | 2 |
| pleckstrin homology domain containing, family A member 6 | PLEKHA6 | Q9Y2H5 | 2 |
| PDS5, regulator of cohesion maintenance, homolog B (S. cerevisiae) | PDS5B | Q9Y2I5 | 2 |
| inhibitor of Bruton agammaglobulinemia tyrosine kinase | IBTK | Q9Y3T8 | 2 |
| torsin A interacting protein 1 | TOR1AIP1 | Q9Y3X5 | 2 |
| ribosomal L1 domain containing 1 | RSL1D1 | Q9Y3Z9 | 2 |
| interferon regulatory factor 2 binding protein 1 | IRF2BP1 | Q9Y4P4 | 2 |
| zinc finger protein 608 | ZNF608 | Q9Y5A1 | 2 |
| origin recognition complex, subunit 6 like (yeast) | ORC6L | Q9Y5N6 | 2 |
| capicua homolog (Drosophila) | CIC | Q9Y6T1 | 2 |
| SET binding protein 1 | SETBP1 | Q9Y6X0 | 2 |
