# Supplementary material for: VENNTURE–A Novel Venn Diagram Investigational Tool for Multiple Pharmacological Dataset Analysis
Source: PLoS One. 2012 May 14;7(5):e36911. doi: 10.1371/journal.pone.0036911 (PMC3351456; doi:10.1371/journal.pone.0036911)
Supplement: Table S12 — Phosphoproteins extracted from 100 µM MeCh-stimulated chronic minimal peroxide (CMP)-state human neuroblastoma SH-SY5Y cells. For each successfully identified protein official symbol, Uniprot accession code and number of peptides recovered are indicated. (DOC) [file pone.0036911.s013.doc]

**Table S12.** Phosphoproteins extracted from 100µM MeCh-stimulated chronic minimal peroxide (CMP)-state human neuroblastoma SH-SY5Y cells. For each successfully identified protein official symbol, Uniprot accession code and number of peptides recovered are indicated.

| **Protein Identification** | **Symbol** | **Accession** | **Peptide** |
| --- | --- | --- | --- |
| v-yes-1 Yamaguchi sarcoma viral related oncogene homolog | LYN | A0AVQ5 | 33 |
| U2-associated SR140 protein | SR140 | A0PJ60 | 26 |
| cysteine-rich protein 2 | CRIP2 | A1A4U1 | 25 |
| nuclear fragile X mental retardation protein interacting protein 2 | NUFIP2 | A1L3A7 | 23 |
| stathmin 1 | STMN1 | A2A2D1 | 23 |
| chromosome 7 open reading frame 47 | C7orf47 | A4D2C5 | 20 |
| synaptopodin 2-like | SYNPO2L | A5PKV9 | 18 |
| RAP1 interacting factor homolog (yeast) | RIF1 | A6NC27 | 15 |
| family with sequence similarity 54, member B | FAM54B | A6NCB4 | 12 |
| tolloid-like 2 | TLL2 | A6NDK0 | 12 |
| family with sequence similarity 186, member A | FAM186A | A6NE01 | 12 |
| chromosome 12 open reading frame 24 | C12orf24 | A6NH30 | 10 |
| ribonucleoprotein, PTB-binding 1 | RAVER1 | A6NMU4 | 10 |
| inter-alpha (globulin) inhibitor H5-like | ITIH5L | A6NN03 | 10 |
| KIAA0467; chromosome 1 open reading frame 84 | KIAA0467 | A7E2X4 | 9 |
| ataxin 2-like | ATXN2L | A8K1R6 | 9 |
| phosphogluconate dehydrogenase | PGD | A8K2Y9 | 9 |
| leucine rich repeat containing 41 | LRRC41 | A8K5G8 | 9 |
| potassium channel tetramerisation domain containing 15 | KCTD15 | A8K600 | 9 |
| epsin 3 | EPN3 | A8K6J3 | 8 |
| enoyl-Coenzyme A, hydratase/3-hydroxyacyl Coenzyme A dehydrogenase | EHHADH | A8K6Y3 | 8 |
| D4, zinc and double PHD fingers family 2 | DPF2 | A8K7C9 | 8 |
| neural cell adhesion molecule 1 | NCAM1 | A8K8T8 | 8 |
| alkaline phosphatase, placental-like 2 | ALPPL2 | A8KAF2 | 7 |
| ubiquitin interaction motif containing 1 | UIMC1 | A8MSA1 | 7 |
| ubiquitin fusion degradation 1 like (yeast) | UFD1L | A8MW31 | 7 |
| doublecortin | DCX | A9Z1V8 | 7 |
| HLA-B associated transcript 3 | BAT3 | B0UX84 | 7 |
| interferon regulatory factor 2 binding protein 2 | IRF2BP2 | B1AM36 | 7 |
| adenosine deaminase, RNA-specific | ADAR | B1AQQ9 | 7 |
| ribosomal protein S3 pseudogene 3; ribosomal protein S3 | RPS3 | B2R7N5 | 6 |
| flavin containing monooxygenase 3 | FMO3 | B2R816 | 6 |
| serine/threonine kinase 10 | STK10 | B2R8F5 | 6 |
| RNA binding motif protein 25 | RBM25 | B2RNA8 | 6 |
| NOL1/NOP2/Sun domain family, member 2 | NSUN2 | B2RNR4 | 6 |
| CD164 sialomucin-like 2 | CD164L2 | B2RPJ0 | 6 |
| zinc finger CCCH-type containing 12B | ZC3H12B | B2RTQ3 | 6 |
| SWI/SNF related, matrix associated, actin dependent regulator of chromatin, subfamily a, member 4 | SMARCA4 | B3KNW7 | 6 |
| nudE nuclear distribution gene E homolog (A. nidulans)-like 1 | NDEL1 | B3KP93 | 6 |
| sushi, von Willebrand factor type A, EGF and pentraxin domain containing 1 | SVEP1 | B3KQM1 | 5 |
| ADAM metallopeptidase domain 19 (meltrin beta) | ADAM19 | B3KRF5 | 5 |
| kinesin light chain 4 | KLC4 | B3KSQ3 | 5 |
| chromosome 1 open reading frame 64 | C1orf64 | B3KXI9 | 5 |
| fibrillin 2 | FBN2 | B4DU01 | 5 |
| ADP-ribosylation factor interacting protein 1 | ARFIP1 | B4E273 | 5 |
| ets variant 2 | ETV2 | B5MD42 | 5 |
| PHD and ring finger domains 1 | PHRF1 | B7ZM64 | 5 |
| myosin, heavy chain 16 | MYH16 | BAB15219.1 | 5 |
| proprotein convertase subtilisin/kexin type 9 | PCSK9 | C0JYY9 | 5 |
| chromosome 17 open reading frame 49 | C17orf49 | C9J4G0 | 5 |
| PIP5K1A and PSMD4-like, pseudogene | PIPSL | CAM19360.1 | 5 |
| similar to Dual specificity protein kinase CLK2 (CDC like kinase 2) | LOC402468 | CH236948.1 | 5 |
| suppressor of Ty 5 homolog (S. cerevisiae) | SUPT5H | O00267 | 5 |
| CD93 molecule | CD93 | O00274 | 5 |
| protein phosphatase 1, regulatory (inhibitor) subunit 10 | PPP1R10 | O00405 | 5 |
| B-cell CLL/lymphoma 9 | BCL9 | O00512 | 5 |
| TRAF-type zinc finger domain containing 1 | TRAFD1 | O14545 | 5 |
| glutaryl-Coenzyme A dehydrogenase | GCDH | O14719 | 5 |
| paired-like homeobox 2a | PHOX2A | O14813 | 5 |
| glycogen synthase kinase 3 alpha | GSK3A | O14959 | 5 |
| NAC alpha domain containing | NACAD | O15069 | 5 |
| API5-like 1; apoptosis inhibitor 5 | API5 | O15441 | 5 |
| squamous cell carcinoma antigen recognized by T cells | SART1 | O43290 | 5 |
| eukaryotic translation initiation factor 4 gamma, 3 | EIF4G3 | O43432 | 4 |
| poly(A) binding protein, nuclear 1 | PABPN1 | O43484 | 4 |
| phospholipase D2 | PLD2 | O43580 | 4 |
| CAP-GLY domain containing linker protein 2 | CLIP2 | O43611 | 4 |
| GLI family zinc finger 2 | GLI2 | O60252 | 4 |
| dyskeratosis congenita 1, dyskerin | DKC1 | O60832 | 4 |
| apoptotic chromatin condensation inducer 1 | ACIN1 | O75158 | 4 |
| tripartite motif-containing 3 | TRIM3 | O75382 | 4 |
| SEC22 vesicle trafficking protein homolog B (S. cerevisiae) | SEC22B | O75396 | 4 |
| serpin peptidase inhibitor, clade I (pancpin), member 2 | SERPINI2 | O75830 | 4 |
| protein kinase D3 | PRKD3 | O94806 | 4 |
| kelch repeat and BTB (POZ) domain containing 11 | KBTBD11 | O94819 | 4 |
| EPM2A (laforin) interacting protein 1 | EPM2AIP1 | O94866 | 4 |
| latrophilin 3 | LPHN3 | O94867 | 4 |
| ADAM metallopeptidase with thrombospondin type 1 motif, 2 | ADAMTS2 | O95450 | 4 |
| follistatin-like 3 (secreted glycoprotein) | FSTL3 | O95633 | 4 |
| structural maintenance of chromosomes 4 | SMC4 | O95752 | 4 |
| eukaryotic translation initiation factor 5B | EIF5B | O95805 | 4 |
| 2',5'-oligoadenylate synthetase 1, 40/46kDa | OAS1 | P00973 | 4 |
| neurofilament, medium polypeptide | NEFM | P07197 | 4 |
| ribosomal protein S17 | RPS17 | P08708 | 4 |
| thymopoietin | TMPO | P08919 | 4 |
| zinc finger protein 679; zinc finger protein 735 | ZNF735 | P0CB33 | 4 |
| GLI family zinc finger 3 | GLI3 | P10071 | 4 |
| microtubule-associated protein tau | MAPT | P10636 | 4 |
| integrin, beta 5 | ITGB5 | P18084 | 4 |
| transcription elongation factor A (SII), 1 pseudogene 2; transcription elongation factor A (SII), 1 | TCEA1 | P23193 | 4 |
| proteasome (prosome, macropain) subunit, alpha type, 3 | PSMA3 | P25788 | 4 |
| zinc finger protein 36, C3H type, homolog (mouse) | ZFP36 | P26651 | 4 |
| IQ motif containing GTPase activating protein 1 | IQGAP1 | P46940 | 4 |
| SWI/SNF related, matrix associated, actin dependent regulator of chromatin, subfamily a, member 2 | SMARCA2 | P51531 | 4 |
| glycoprotein 2 (zymogen granule membrane) | GP2 | P55259 | 4 |
| TPI1 pseudogene; triosephosphate isomerase 1 | TPI1 | P60174 | 4 |
| v-myb myeloblastosis viral oncogene homolog (avian) | MYB | P78525 | 4 |
| glutamyl-prolyl-tRNA synthetase | EPRS | Q05BP6 | 3 |
| host cell factor C1 (VP16-accessory protein) | HCFC1 | Q05C05 | 3 |
| Dmx-like 1 | DMXL1 | Q05C95 | 3 |
| NFKB activating protein | NKAP | Q05D22 | 3 |
| KIAA1704 | KIAA1704 | Q05D87 | 3 |
| nuclear cap binding protein subunit 1, 80kDa | NCBP1 | Q09161 | 3 |
| PCF11, cleavage and polyadenylation factor subunit, homolog (S. cerevisiae) | PCF11 | Q0D2H7 | 3 |
| interleukin enhancer binding factor 3, 90kDa | ILF3 | Q12906 | 3 |
| chromatin assembly factor 1, subunit B (p60) | CHAF1B | Q13112 | 3 |
| p21 protein (Cdc42/Rac)-activated kinase 2 | PAK2 | Q13154 | 3 |
| proteasome (prosome, macropain) 26S subunit, non-ATPase, 2 | PSMD2 | Q13200 | 3 |
| forkhead box K2 | FOXK2 | Q13622 | 3 |
| c-abl oncogene 1, receptor tyrosine kinase | ABL1 | Q13688 | 3 |
| heterogeneous nuclear ribonucleoprotein D (AU-rich element RNA binding protein 1, 37kDa) | HNRNPD | Q14100 | 3 |
| similar to RNA binding motif protein 39; RNA binding motif protein 39 | RBM39 | Q14498 | 3 |
| integrin, alpha 9 | ITGA9 | Q14638 | 3 |
| phosphoprotein enriched in astrocytes 15 | PEA15 | Q14801 | 3 |
| poly(rC) binding protein 1 | PCBP1 | Q14975 | 3 |
| prostaglandin E synthase 3 (cytosolic) | PTGES3 | Q15185 | 3 |
| non-POU domain containing, octamer-binding | NONO | Q15233 | 3 |
| bromodomain containing 2 | BRD2 | Q15310 | 3 |
| telomeric repeat binding factor 2 | TERF2 | Q15554 | 3 |
| nuclear receptor coactivator 2 | NCOA2 | Q15596 | 3 |
| protein tyrosine phosphatase, receptor type, S | PTPRS | Q15718 | 3 |
| adducin 1 (alpha) | ADD1 | Q16156 | 3 |
| ELAV (embryonic lethal, abnormal vision, Drosophila)-like 4 (Hu antigen D) | ELAVL4 | Q16234 | 3 |
| diacylglycerol kinase, delta 130kDa | DGKD | Q16760 | 3 |
| kallikrein B, plasma (Fletcher factor) 1 | KLKB1 | Q17RE8 | 3 |
| KIAA0528 | KIAA0528 | Q17RY7 | 3 |
| transferrin receptor 2 | TFR2 | Q1HE13 | 3 |
| phosphoprotein associated with glycosphingolipid microdomains 1 | PAG1 | Q2M1Z9 | 3 |
| chromosome 15 open reading frame 59 | C15orf59 | Q2T9L4 | 3 |
| LIM domain and actin binding 1 | LIMA1 | Q2TAN7 | 3 |
| heparan sulfate proteoglycan 2 | HSPG2 | Q2VPA1 | 3 |
| nestin | NES | Q2YDX4 | 3 |
| calcium regulated heat stable protein 1, 24kDa | CARHSP1 | Q2YDX5 | 3 |
| protein kinase, cAMP-dependent, catalytic, alpha | PRKACA | Q32P54 | 3 |
| HECT, UBA and WWE domain containing 1 | HUWE1 | Q3B7K0 | 3 |
| ubiquitin specific peptidase 42 | USP42 | Q3C166 | 3 |
| reticulon 4 | RTN4 | Q3LIF4 | 3 |
| heterogeneous nuclear ribonucleoprotein A1-like 3 | HNRPA1L3 | Q3MI39 | 3 |
| POM121-like protein | POM121L1 | Q3SYA9 | 3 |
| suppressor of defective silencing 3 homolog (S. cerevisiae) | SUDS3 | Q4KMQ5 | 2 |
| mucin 6, oligomeric mucus/gel-forming | MUC6 | Q4L207 | 2 |
| afamin | AFM | Q4W5C5 | 2 |
| SWI/SNF related, matrix associated, actin dependent regulator of chromatin, subfamily a, member 5 | SMARCA5 | Q4W5G3 | 2 |
| AP2 associated kinase 1 | AAK1 | Q4ZFZ3 | 2 |
| splicing factor, arginine/serine-rich 9 | SFRS9 | Q52LD1 | 2 |
| solute carrier family 35, member C2 | SLC35C2 | Q53GK3 | 2 |
| Wolf-Hirschhorn syndrome candidate 2 | WHSC2 | Q53GS8 | 2 |
| general transcription factor IIIC, polypeptide 2, beta 110kDa | GTF3C2 | Q53QN0 | 2 |
| ArfGAP with FG repeats 1 | AGFG1 | Q53R11 | 2 |
| spectrin, beta, non-erythrocytic 1 | SPTBN1 | Q53R99 | 2 |
| activating transcription factor 2 | ATF2 | Q53RY2 | 2 |
| endothelial PAS domain protein 1 | EPAS1 | Q53SM6 | 2 |
| thyroid hormone receptor interactor 12 | TRIP12 | Q53TE7 | 2 |
| gamma-aminobutyric acid (GABA) A receptor, alpha 5 | GABRA5 | Q53XL6 | 2 |
| centromere protein J | CENPJ | Q569I1 | 2 |
| zinc finger protein 638 | ZNF638 | Q57Z90 | 2 |
| sodium channel, voltage-gated, type I, alpha subunit | SCN1A | Q585T7 | 2 |
| heat shock protein 90kDa alpha (cytosolic), class B member 2 (pseudogene) | HSP90AB2P | Q58FF8 | 2 |
| insulin-like growth factor 2 receptor | IGF2R | Q59EZ3 | 2 |
| drebrin-like | DBNL | Q59FH4 | 2 |
| cyclin K | CCNK | Q59FT6 | 2 |
| exosome component 10 | EXOSC10 | Q59G73 | 2 |
| topoisomerase (DNA) II beta 180kDa | TOP2B | Q59H80 | 2 |
| sorbin and SH3 domain containing 3 | SORBS3 | Q5BJE4 | 2 |
| matrin 3 | MATR3 | Q5CZA7 | 2 |
| motile sperm domain containing 1 | MOSPD1 | Q5H9C5 | 2 |
| family with sequence similarity 76, member B | FAM76B | Q5HYJ3 | 2 |
| lamin A/C | LMNA | Q5I6Y6 | 2 |
| SH2 domain containing 3C | SH2D3C | Q5JU30 | 2 |
| 5'-nucleotidase, cytosolic II | NT5C2 | Q5JUV5 | 2 |
| FERM, RhoGEF (ARHGEF) and pleckstrin domain protein 1 (chondrocyte-derived) | FARP1 | Q5JV94 | 2 |
| NSFL1 (p97) cofactor (p47) | NSFL1C | Q5JXA5 | 2 |
| septin 7 | 40793 | Q5JXL7 | 2 |
| calcium channel, voltage-dependent, beta 2 subunit | CACNB2 | Q5QJA0 | 2 |
| heterogeneous nuclear ribonucleoprotein U (scaffold attachment factor A) | HNRNPU | Q5RI19 | 2 |
| death-domain associated protein | DAXX | Q5STR5 | 2 |
| ankyrin repeat and sterile alpha motif domain containing 1A | ANKS1A | Q5SYR2 | 2 |
| hepatoma-derived growth factor (high-mobility group protein 1-like) | HDGF | Q5SZ07 | 2 |
| leucine rich repeat containing 27 | LRRC27 | Q5SZH9 | 2 |
| HORMA domain containing 1 | HORMAD1 | Q5T5I4 | 2 |
| caspase 8 associated protein 2 | CASP8AP2 | Q5T791 | 2 |
| bystin-like | BYSL | Q5T8J2 | 2 |
| LEM domain containing 2 | LEMD2 | Q5T972 | 2 |
| colipase, pancreatic | CLPS | Q5T9G7 | 2 |
| nucleoporin 153kDa | NUP153 | Q5T9I7 | 2 |
| proteasome (prosome, macropain) subunit, beta type, 7 | PSMB7 | Q5TBG6 | 2 |
| outer dense fiber of sperm tails 2-like | ODF2L | Q5TBX3 | 2 |
| nardilysin (N-arginine dibasic convertase) | NRD1 | Q5TFB9 | 2 |
| AT hook, DNA binding motif, containing 1 | AHDC1 | Q5TGY4 | 2 |
| myeloid leukemia factor 2 | MLF2 | Q5U0N1 | 2 |
| GTPase activating protein (SH3 domain) binding protein 1 | G3BP1 | Q5U0Q1 | 2 |
| wings apart-like homolog (Drosophila) | WAPAL | Q5VSK5 | 2 |
| serine/arginine repetitive matrix 1 | SRRM1 | Q5VVN4 | 2 |
| antigen identified by monoclonal antibody Ki-67 | MKI67 | Q5VWH2 | 2 |
| sorting nexin family member 30 | SNX30 | Q5VWJ9 | 2 |
| DAB2 interacting protein | DAB2IP | Q5VWQ8 | 2 |
| zinc finger, MYM-type 4 | ZMYM4 | Q5VZL5 | 2 |
| soc-2 suppressor of clear homolog (C. elegans) | SHOC2 | Q5VZS9 | 2 |
| ankyrin repeat domain 30A | ANKRD30A | Q5W025 | 2 |
| ribonucleotide reductase M2 polypeptide | RRM2 | Q5WRU7 | 2 |
| WWC family member 3 | WWC3 | Q659C1 | 2 |
| phytanoyl-CoA 2-hydroxylase interacting protein-like | PHYHIPL | Q68DF3 | 2 |
| heterogeneous nuclear ribonucleoprotein H1 (H) | HNRNPH1 | Q68DG4 | 2 |
| apolipoprotein O-like | APOOL | Q68DW4 | 2 |
| ATP-binding cassette, sub-family F (GCN20), member 1 | ABCF1 | Q69YP6 | 2 |
| epidermal growth factor receptor pathway substrate 15-like 1 | EPS15L1 | Q69YZ4 | 2 |
| MDN1, midasin homolog (yeast) | MDN1 | Q6AI22 | 2 |
| similar to Bcl-2-associated transcription factor 1 (Btf); BCL2-associated transcription factor 1 | BCLAF1 | Q6DCA8 | 2 |
| fumarylacetoacetate hydrolase domain containing 1 | FAHD1 | Q6FIC7 | 2 |
| ADAM metallopeptidase domain 20 | ADAM20 | Q6GTZ1 | 2 |
| StAR-related lipid transfer (START) domain containing 7 | STARD7 | Q6GU43 | 2 |
| mitogen-activated protein kinase associated protein 1 | MAPKAP1 | Q6GVJ2 | 2 |
| eukaryotic translation initiation factor 3, subunit G | EIF3G | Q6IAM0 | 2 |
| heterogeneous nuclear ribonucleoprotein K; similar to heterogeneous nuclear ribonucleoprotein K | HNRNPK | Q6IBN1 | 2 |
| GTP binding protein 1 | GTPBP1 | Q6IC67 | 2 |
| RAB12, member RAS oncogene family | RAB12 | Q6IQ22 | 2 |
| Nipped-B homolog (Drosophila) | NIPBL | Q6KCD6 | 2 |
| LIM and calponin homology domains 1 | LIMCH1 | Q6N054 | 2 |
| solute carrier family 25 (mitochondrial carrier; phosphate carrier), member 24 | SLC25A24 | Q6NUK1 | 2 |
| cartilage intermediate layer protein 2 | CILP2 | Q6NV88 | 2 |
| myristoylated alanine-rich protein kinase C substrate | MARCKS | Q6NVI1 | 2 |
| histone cluster 1, H3 | HIST2H3A | Q6NWP9 | 2 |
| MARCKS-like 1 | MARCKSL1 | Q6NXS5 | 2 |
| thyroid hormone receptor associated protein 3 | THRAP3 | Q6P0P7 | 2 |
| UV radiation resistance associated gene | UVRAG | Q6P1X0 | 2 |
| NIMA (never in mitosis gene a)-related kinase 5 | NEK5 | Q6P3R8 | 2 |
| SAM and SH3 domain containing 1 | SASH1 | Q6P4R9 | 2 |
| solute carrier family 5 (sodium/glucose cotransporter), member 10 | SLC5A10 | Q6P5X0 | 2 |
| sterile alpha motif domain containing 1 | SAMD1 | Q6PIS7 | 2 |
| microtubule-associated protein 1B | MAP1B | Q6PJD3 | 2 |
| pleckstrin homology domain containing, family H (with MyTH4 domain) member 1 | PLEKHH1 | Q6PJL4 | 2 |
| KH domain containing, RNA binding, signal transduction associated 1 | KHDRBS1 | Q6PJX7 | 2 |
| splicing factor, arginine/serine-rich 11 | SFRS11 | Q6PJY9 | 2 |
| chromosome 14 open reading frame 43 | C14orf43 | Q6PK59 | 2 |
| RNA binding motif protein 10 | RBM10 | Q6PKH5 | 2 |
| zinc finger CCCH-type containing 14 | ZC3H14 | Q6PUI8 | 2 |
| solute carrier family 25, member 47 | HDMCP | Q6Q0C1.1 | 2 |
| enhancer of zeste homolog 2 (Drosophila) | EZH2 | Q6R125 | 2 |
| estrogen receptor binding site associated, antigen, 9 | EBAG9 | Q6R3F1 | 2 |
| chromosome 7 open reading frame 51 | C7orf51 | Q6U9Y3 | 2 |
| chromosome 6 open reading frame 89 | C6orf89 | Q6UWU4 | 2 |
| ring finger and SPRY domain containing 1 | RSPRY1 | Q6UX21 | 2 |
| regulatory factor X, 4 (influences HLA class II expression) | RFX4 | Q6YM53 | 2 |
| sodium leak channel, non-selective | NALCN | Q6ZMI7 | 2 |
| hypothetical LOC100130009; high mobility group AT-hook 1 | HMGA1 | Q6ZP45 | 2 |
| FRY-like | FRYL | Q6ZR29 | 2 |
| coiled-coil domain containing 144C | CCDC144C | Q6ZU57 | 2 |
| Rho GTPase activating protein 17 | ARHGAP17 | Q6ZUS4 | 2 |
| keratin 3 | KRT3 | Q701L8 | 2 |
| synaptopodin | SYNPO | Q71HJ6 | 2 |
| nucleoporin 214kDa | NUP214 | Q75R47 | 2 |
| ligase I, DNA, ATP-dependent | LIG1 | Q76GR4 | 2 |
| cortactin | CTTN | Q76MU0 | 2 |
| HECT, C2 and WW domain containing E3 ubiquitin protein ligase 1 | HECW1 | Q76N89 | 2 |
| pinin, desmosome associated protein | PNN | Q7KYL1 | 2 |
| prolyl-tRNA synthetase 2, mitochondrial (putative) | PARS2 | Q7L3T8 | 2 |
| similar to U5 snRNP-specific protein, 200 kDa; small nuclear ribonucleoprotein 200kDa (U5) | SNRNP200 | Q7L5W4 | 2 |
| alpha thalassemia/mental retardation syndrome X-linked (RAD54 homolog, S. cerevisiae) | ATRX | Q7Z2J1 | 2 |
| titin | TTN | Q7Z2X3 | 2 |
| mitogen-activated protein kinase kinase 2 pseudogene; mitogen-activated protein kinase kinase 2 | MAP2K2 | Q7Z370 | 2 |
| methyl CpG binding protein 2 (Rett syndrome) | MECP2 | Q7Z384 | 2 |
| tumor protein p53 binding protein 1 | TP53BP1 | Q7Z3U4 | 2 |
| eukaryotic translation initiation factor 2A, 65kDa | EIF2A | Q7Z4E9 | 2 |
| sperm associated antigen 1 | SPAG1 | Q7Z5G1 | 2 |
| mucin 19, oligomeric | MUC19 | Q7Z5P9 | 2 |
| tripartite motif-containing 28 | TRIM28 | Q7Z632 | 2 |
| kinesin family member 21A | KIF21A | Q7Z668 | 2 |
| hypothetical protein LOC387763 | AG2 | Q7Z7L8 | 2 |
| chromodomain helicase DNA binding protein 7 | CHD7 | Q7Z7Q2 | 2 |
| zinc finger and BTB domain containing 1 | ZBTB1 | Q86SW8 | 2 |
| Hypothetical protein | DKFZp451F173 | Q86T62 | 2 |
| taxilin alpha | TXLNA | Q86T86 | 2 |
| filamin A, alpha (actin binding protein 280) | FLNA | Q86TQ3 | 2 |
| bromodomain adjacent to zinc finger domain, 1B | BAZ1B | Q86UJ6 | 2 |
| kinectin 1 (kinesin receptor) | KTN1 | Q86W57 | 2 |
| sperm associated antigen 9 | SPAG9 | Q86WC7 | 2 |
| chromosome 17 open reading frame 82 | C17orf82 | Q86X59 | 2 |
| regulation of nuclear pre-mRNA domain containing 2 | RPRD2 | Q86XD2 | 2 |
| DENN/MADD domain containing 2A | DENND2A | Q86XY0 | 2 |
| microtubule-associated protein 4 | MAP4 | Q86Y04 | 2 |
| DEAD (Asp-Glu-Ala-Asp) box polypeptide 54 | DDX54 | Q86YT8 | 2 |
| nuclear receptor co-repressor 1 | NCOR1 | Q86YY0 | 2 |
| poly (ADP-ribose) polymerase 1 | PARP1 | Q8IUZ9 | 2 |
| calcineurin binding protein 1 | CABIN1 | Q8IVX9 | 2 |
| RAB37, member RAS oncogene family | RAB37 | Q8IWA7 | 2 |
| ATPase family, AAA domain containing 2B | ATAD2B | Q8IWJ3 | 2 |
| DEAD (Asp-Glu-Ala-Asp) box polypeptide 51 | DDX51 | Q8IXK5 | 2 |
| chromodomain helicase DNA binding protein 4 | CHD4 | Q8IXZ5 | 2 |
| cartilage intermediate layer protein, nucleotide pyrophosphohydrolase | CILP | Q8IYI5 | 2 |
| zinc finger protein 683 | ZNF683 | Q8IZ20 | 2 |
| G protein-coupled receptor 116 | GPR116 | Q8IZF2 | 2 |
| chromosome 5 open reading frame 41 | C5orf41 | Q8IZG1 | 2 |
| serpin peptidase inhibitor, clade B (ovalbumin), member 8 | SERPINB8 | Q8N178 | 2 |
| zinc finger protein 687 | ZNF687 | Q8N1G0 | 2 |
| prominin 2 | PROM2 | Q8N271 | 2 |
| BMP2 inducible kinase | BMP2K | Q8N2G7 | 2 |
| SEC16 homolog A (S. cerevisiae) | SEC16A | Q8N347 | 2 |
| transmembrane protein 132C | TMEM132C | Q8N3T6 | 2 |
| microtubule-associated protein 1S | MAP1S | Q8N3W5 | 2 |
| chromosome 6 open reading frame 223 | C6orf223 | Q8N575 | 2 |
| cytoplasmic linker associated protein 1 | CLASP1 | Q8N5B8 | 2 |
| testis expressed 9 | TEX9 | Q8N6V9 | 2 |
| cyclin Y-like 1 | CCNYL1 | Q8N7R7 | 2 |
| similar to RNA binding motif protein, X-linked; similar to hCG2011544 | RBMX | Q8N8Y7 | 2 |
| nucleolin | NCL | Q8NB06 | 2 |
| pygopus homolog 2 (Drosophila) | PYGO2 | Q8NBG9 | 2 |
| solute carrier family 35, member E1 | SLC35E1 | Q8NBQ2 | 2 |
| zinc finger protein 318 | ZNF318 | Q8NEM6 | 2 |
| catenin (cadherin-associated protein), beta 1, 88kDa | CTNNB1 | Q8NEW9 | 2 |
| olfactory receptor, family 10, subfamily J, member 6 pseudogene | OR10J6P | Q8NGY7 | 2 |
| WD repeat domain 43 | WDR43 | Q8TB67 | 2 |
| prospero homeobox 1 | PROX1 | Q8TB91 | 2 |
| glutamate receptor, metabotropic 3 | GRM3 | Q8TBH9 | 2 |
| family with sequence similarity 71, member B | FAM71B | Q8TEW9 | 2 |
| peptidylprolyl isomerase (cyclophilin)-like 4 | PPIL4 | Q8WUA2 | 2 |
| short chain dehydrogenase/reductase family 42E, member 1 | HSPC105 | Q8WUS8.2 | 2 |
| zinc finger protein 296 | ZNF296 | Q8WUU4 | 2 |
| myosin IXB | MYO9B | Q8WVD2 | 2 |
| kinesin family member 23 | KIF23 | Q8WVP0 | 2 |
| smoothelin | SMTN | Q8WWW1 | 2 |
| ATPase, class VI, type 11C | ATP11C | Q8WX24 | 2 |
| glutamine-fructose-6-phosphate transaminase 1 | GFPT1 | Q8WYR5 | 2 |
| telomeric repeat binding factor 2, interacting protein | TERF2IP | Q8WYZ3 | 2 |
| damage-specific DNA binding protein 2, 48kDa | DDB2 | Q92466 | 2 |
| twist homolog 1 (Drosophila) | TWIST1 | Q92487 | 2 |
| H1 histone family, member X | H1FX | Q92522 | 2 |
| TBC1 domain family, member 5 | TBC1D5 | Q92609 | 2 |
| bromodomain containing 3 | BRD3 | Q92645 | 2 |
| eukaryotic translation elongation factor 1 delta (guanine nucleotide exchange protein) | EEF1D | Q969J1 | 2 |
| minichromosome maintenance complex component 2 | MCM2 | Q969W7 | 2 |
| hypothetical LOC642975; chromosome 20 open reading frame 30 | C20orf30 | Q96A57 | 2 |
| PWP2 periodic tryptophan protein homolog (yeast) | PWP2 | Q96A77 | 2 |
| Janus kinase and microtubule interacting protein 2 | JAKMIP2 | Q96AA8 | 2 |
| ATPase, class II, type 9A | ATP9A | Q96B35 | 2 |
| scribbled homolog (Drosophila) | SCRIB | Q96C69 | 2 |
| septin 2 | 40788 | Q96CB0 | 2 |
| similar to chromobox homolog 3; chromobox homolog 3 (HP1 gamma homolog, Drosophila) | CBX3 | Q96CD7 | 2 |
| serine/threonine kinase 11 interacting protein | STK11IP | Q96CN3 | 2 |
| coiled-coil domain containing 124 | CCDC124 | Q96CT7 | 2 |
| ligase III, DNA, ATP-dependent | LIG3 | Q96DF0 | 2 |
| zinc finger CCCH-type containing 18 | ZC3H18 | Q96DG4 | 2 |
| nexilin (F actin binding protein) | NEXN | Q96DL0 | 2 |
| ring finger protein 31 | RNF31 | Q96EP0 | 2 |
| cofactor of BRCA1 | COBRA1 | Q96EW5 | 2 |
| cytoplasmic linker associated protein 2 | CLASP2 | Q96F87 | 2 |
| zinc finger with KRAB and SCAN domains 1 | ZKSCAN1 | Q96FA2 | 2 |
| glucocorticoid induced transcript 1 | GLCCI1 | Q96FD0 | 2 |
| SLU7 splicing factor homolog (S. cerevisiae) | SLU7 | Q96FM9 | 2 |
| secretory carrier membrane protein 3 | SCAMP3 | Q96FR8 | 2 |
| heterogeneous nuclear ribonucleoprotein U-like 1 | HNRNPUL1 | Q96G37 | 2 |
| hepatoma-derived growth factor-related protein 2 | HDGFRP2 | Q96GI5 | 2 |
| leucine-rich repeats and WD repeat domain containing 1 | LRWD1 | Q96GJ2 | 2 |
| SWI/SNF related, matrix associated, actin dependent regulator of chromatin, subfamily c, member 2 | SMARCC2 | Q96GY4 | 2 |
| zinc finger protein 828 | ZNF828 | Q96JM3 | 2 |
| zinc finger protein 41 | ZNF41 | Q96LE8 | 2 |
| beta-1,4-N-acetyl-galactosaminyl transferase 4 | B4GALNT4 | Q96LV2 | 2 |
| leucine-rich repeats and guanylate kinase domain containing | LRGUK | Q96M69 | 2 |
| Leo1, Paf1/RNA polymerase II complex component, homolog (S. cerevisiae) | LEO1 | Q96N99 | 2 |
| similar to dynein cytoplasmic 1 intermediate chain 2; dynein, cytoplasmic 1, intermediate chain 2 | DYNC1I2 | Q96NG7 | 2 |
| A kinase (PRKA) anchor protein 13 | AKAP13 | Q96P79 | 2 |
| G protein regulated inducer of neurite outgrowth 1 | GPRIN1 | Q96PZ4 | 2 |
| arginine/serine-rich coiled-coil 1 | RSRC1 | Q96QK2 | 2 |
| CTF18, chromosome transmission fidelity factor 18 homolog (S. cerevisiae) | CHTF18 | Q96S08 | 2 |
| peroxisomal biogenesis factor 1 | PEX1 | Q96S72 | 2 |
| nuclear fragile X mental retardation protein interacting protein 1 | NUFIP1 | Q96SG1 | 2 |
| family with sequence similarity 40, member A | FAM40A | Q96SN2 | 2 |
| protein tyrosine phosphatase-like A domain containing 1 | PTPLAD1 | Q96T12 | 2 |
| remodeling and spacing factor 1 | RSF1 | Q96T23 | 2 |
| AT rich interactive domain 1A (SWI-like) | ARID1A | Q96T89 | 2 |
| myosin, heavy chain 9, non-muscle | MYH9 | Q99529 | 2 |
| HIV-1 Tat specific factor 1 | HTATSF1 | Q99730 | 2 |
| microtubule-associated protein 2 | MAP2 | Q99976 | 2 |
| superkiller viralicidic activity 2-like (S. cerevisiae) | SKIV2L | Q9BQJ5 | 2 |
| RD RNA binding protein | RDBP | Q9BQJ6 | 2 |
| leucine zipper protein 3, pseudogene | LUZPP1 | Q9BQQ8 | 2 |
| ubiquitin-conjugating enzyme E2O | UBE2O | Q9BSW1 | 2 |
| potassium voltage-gated channel, subfamily H (eag-related), member 2 | KCNH2 | Q9BT72 | 2 |
| neural proliferation, differentiation and control, 1 | NPDC1 | Q9BTD6 | 2 |
| single stranded DNA binding protein 3; hypothetical LOC100131851 | SSBP3 | Q9BTM0 | 2 |
| chromosome 11 open reading frame 84 | C11orf84 | Q9BUA3 | 2 |
| zinc finger, CW type with PWWP domain 1 | ZCWPW1 | Q9BUD0 | 2 |
| transgelin 2 | TAGLN2 | Q9BUH5 | 2 |
| HIRA interacting protein 3 | HIRIP3 | Q9BW71 | 2 |
| cytochrome b-561 | CYB561 | Q9BWR9 | 2 |
| synaptonemal complex protein 2 | SYCP2 | Q9BX26 | 2 |
| chromosome 22 open reading frame 23 | C22orf23 | Q9BZE7 | 2 |
| testis specific, 10 | TSGA10 | Q9BZW7 | 2 |
| dedicator of cytokinesis 7 | DOCK7 | Q9C092 | 2 |
| zinc finger protein 518B | ZNF518B | Q9C0D4 | 2 |
| tetratricopeptide repeat, ankyrin repeat and coiled-coil containing 1 | TANC1 | Q9C0D5 | 2 |
| zinc finger, MYM-type 2 | ZMYM2 | Q9H0V5 | 2 |
| phosphoglucomutase 1 | PGM1 | Q9H1D2 | 2 |
| nuclear casein kinase and cyclin-dependent kinase substrate 1 | NUCKS1 | Q9H1E3 | 2 |
| SAPS domain family, member 3 | SAPS3 | Q9H2K6 | 2 |
| hematological and neurological expressed 1 | HN1 | Q9H3K0 | 2 |
| SAM domain and HD domain 1 | SAMHD1 | Q9H3U9 | 2 |
| DnaJ (Hsp40) homolog, subfamily C, member 5 | DNAJC5 | Q9H3Z5 | 2 |
| tumor protein D52-like 2 | TPD52L2 | Q9H3Z6 | 2 |
| pericentriolar material 1 | PCM1 | Q9H4A2 | 2 |
| mitochondrial antiviral signaling protein | MAVS | Q9H4Y1 | 2 |
| zinc finger protein 671 | ZNF671 | Q9H5E9 | 2 |
| chromosome 6 open reading frame 97 | C6orf97 | Q9H5M3 | 2 |
| retinoblastoma binding protein 6 | RBBP6 | Q9H5M5 | 2 |
| coiled-coil domain containing 86 | CCDC86 | Q9H6F5 | 2 |
| fidgetin | FIGN | Q9H6M5 | 2 |
| DENN/MADD domain containing 5A | DENND5A | Q9H6U7 | 2 |
| arginine/serine-rich coiled-coil 2 | RSRC2 | Q9H864 | 2 |
| dehydrogenase/reductase (SDR family) member 12 | DHRS12 | Q9H8H1 | 2 |
| H2A histone family, member Y | H2AFY | Q9H8P3 | 2 |
| centromere protein T | CENPT | Q9H901 | 2 |
| myelin expression factor 2 | MYEF2 | Q9H922 | 2 |
| M-phase phosphoprotein 9 | MPHOSPH9 | Q9H976 | 2 |
| coiled-coil domain containing 71 | CCDC71 | Q9H9F1 | 2 |
| ring finger protein 20 | RNF20 | Q9H9Y7 | 2 |
| chromosome 12 open reading frame 43 | C12orf43 | Q9H9Z7 | 2 |
| pumilio homolog 2 (Drosophila) | PUM2 | Q9HAN2 | 2 |
| STIP1 homology and U-box containing protein 1 | STUB1 | Q9HBT1 | 2 |
| otoferlin | OTOF | Q9HC10 | 2 |
| peter pan homolog (Drosophila) | PPAN | Q9NQ55 | 2 |
| regulation of nuclear pre-mRNA domain containing 1B | RPRD1B | Q9NQG5 | 2 |
| exosome component 5 | EXOSC5 | Q9NQT4 | 2 |
| DEAD (Asp-Glu-Ala-Asp) box polypeptide 21 | DDX21 | Q9NR30 | 2 |
| eukaryotic translation initiation factor 4E nuclear import factor 1 | EIF4ENIF1 | Q9NRA8 | 2 |
| spectrin, beta, non-erythrocytic 5 | SPTBN5 | Q9NRC6 | 2 |
| ST6 (alpha-N-acetyl-neuraminyl-2,3-beta-galactosyl-1,3)-N-acetylgalactosaminide alpha-2,6-sialyltransferase 1 | ST6GALNAC1 | Q9NSC6 | 2 |
| Holliday junction recognition protein | HJURP | Q9NSL8 | 2 |
| centrosomal protein 170kDa | CEP170 | Q9NSN9 | 2 |
| protein phosphatase 1, regulatory (inhibitor) subunit 12C | PPP1R12C | Q9NT00 | 2 |
| NFS1 nitrogen fixation 1 homolog (S. cerevisiae) | NFS1 | Q9NTZ5 | 2 |
| anillin, actin binding protein | ANLN | Q9NVP0 | 2 |
| transmembrane protein 39B | TMEM39B | Q9NW51 | 2 |
| kinesin family member 26B | KIF26B | Q9NWB4 | 2 |
| SAFB-like, transcription modulator | SLTM | Q9NWH9 | 2 |
| tetratricopeptide repeat domain 27 | TTC27 | Q9NWR4 | 2 |
| ubiquitin specific peptidase 24 | USP24 | Q9NXD1 | 2 |
| kinesin family member 4B; kinesin family member 4A | KIF4A | Q9NY24 | 2 |
| serine/arginine repetitive matrix 2; hypothetical LOC100132779 | SRRM2 | Q9P0G1 | 2 |
| Rho guanine nucleotide exchange factor (GEF) 12 | ARHGEF12 | Q9P149 | 2 |
| ash1 (absent, small, or homeotic)-like (Drosophila) | ASH1L | Q9P2C7 | 2 |
| phosphatidylinositol 4-kinase, catalytic, beta | PI4KB | Q9UBF8 | 2 |
| heat shock 27kDa protein-like 2 pseudogene; heat shock 27kDa protein 1 | HSPB1 | Q9UC31 | 2 |
| membrane associated guanylate kinase, WW and PDZ domain containing 2 | MAGI2 | Q9UDU1 | 2 |
| similar to hCG1820375; PRP4 pre-mRNA processing factor 4 homolog B (yeast) | PRPF4B | Q9UEE6 | 2 |
| dopey family member 2 | DOPEY2 | Q9UEZ3 | 2 |
| Treacher Collins-Franceschetti syndrome 1 | TCOF1 | Q9UFD4 | 2 |
| La ribonucleoprotein domain family, member 1 | LARP1 | Q9UFD7 | 2 |
| G-protein signaling modulator 1 (AGS3-like, C. elegans) | GPSM1 | Q9UFS8 | 2 |
| phospholipase C, gamma 1 | PLCG1 | Q9UFY1 | 2 |
| drebrin 1 | DBN1 | Q9UFZ5 | 2 |
| like-glycosyltransferase | LARGE | Q9UGG3 | 2 |
| progesterone receptor membrane component 1 | PGRMC1 | Q9UGJ9 | 2 |
| ubiquitin associated protein 2-like | UBAP2L | Q9UGL5 | 2 |
| mediator complex subunit 13 | MED13 | Q9UHV7 | 2 |
| nucleoporin 98kDa | NUP98 | Q9UHX0 | 2 |
| zinc finger protein 91 homolog (mouse); ZFP91-CNTF readthrough transcript; ciliary neurotrophic factor | ZFP91 | Q9UI87 | 2 |
| midline 2 | MID2 | Q9UJR9 | 2 |
| cyclin L1 | CCNL1 | Q9UK58 | 2 |
| synaptopodin 2 | SYNPO2 | Q9UK89 | 2 |
| CDC42 effector protein (Rho GTPase binding) 3 | CDC42EP3 | Q9UKI2 | 2 |
| G patch domain containing 8 | GPATCH8 | Q9UKJ3 | 2 |
| SON DNA binding protein | SON | Q9UKP9 | 2 |
| nucleoporin 50kDa | NUP50 | Q9UKX7 | 2 |
| microtubule-associated protein 1A | MAP1A | Q9UL09 | 2 |
| KIAA1211 | KIAA1211 | Q9ULK9 | 2 |
| apolipoprotein B (including Ag(x) antigen) | APOB | Q9UMN0 | 2 |
| nuclear mitotic apparatus protein 1 | NUMA1 | Q9UNL7 | 2 |
| ataxin 7 | ATXN7 | Q9UPD8 | 2 |
| thrombospondin, type I, domain containing 7A | THSD7A | Q9UPZ6 | 2 |
| GRB2-associated binding protein 2 | GAB2 | Q9UQC2 | 2 |
| PDZ and LIM domain 4 | PDLIM4 | Q9Y292 | 2 |
| A kinase (PRKA) anchor protein 2; paralemmin 2; PALM2-AKAP2 readthrough transcript | AKAP2 | Q9Y2D5 | 2 |
| PDS5, regulator of cohesion maintenance, homolog B (S. cerevisiae) | PDS5B | Q9Y2I5 | 2 |
| myosin, heavy chain 15 | MYH15 | Q9Y2K3 | 2 |
| N-acetylated alpha-linked acidic dipeptidase 2 | NAALAD2 | Q9Y3Q0 | 2 |
| inhibitor of Bruton agammaglobulinemia tyrosine kinase | IBTK | Q9Y3T8 | 2 |
| ribosomal L1 domain containing 1 | RSL1D1 | Q9Y3Z9 | 2 |
| PDS5, regulator of cohesion maintenance, homolog A (S. cerevisiae) | PDS5A | Q9Y4D4 | 2 |
| KIAA0284 | KIAA0284 | Q9Y4F5 | 2 |
| interferon regulatory factor 2 binding protein 1 | IRF2BP1 | Q9Y4P4 | 2 |
| similar to poly (ADP-ribose) glycohydrolase; poly (ADP-ribose) glycohydrolase | PARG | Q9Y4W7 | 2 |
| pogo transposable element with ZNF domain | POGZ | Q9Y4X7 | 2 |
| leucine rich repeat (in FLII) interacting protein 2 | LRRFIP2 | Q9Y608 | 2 |
| dynein, cytoplasmic 1, light intermediate chain 1 | DYNC1LI1 | Q9Y6G9 | 2 |
