# Supplementary material for: VENNTURE–A Novel Venn Diagram Investigational Tool for Multiple Pharmacological Dataset Analysis
Source: PLoS One. 2012 May 14;7(5):e36911. doi: 10.1371/journal.pone.0036911 (PMC3351456; doi:10.1371/journal.pone.0036911)
Supplement: Table S13 — Dose-dependent acetyl-β-methylcholine-stimulated phosphoproteins in control-state human neuroblastoma SH-SY5Y cells. Ligand stimulation with acetyl-β-methylcholine (MeCh) was for 15 minutes before cell lysate protein extraction and titanium dioxide-mediated purification. (DOC) [file pone.0036911.s014.doc]

**Table S13.** Dose-dependent acetyl-β-methylcholine-stimulated phosphoproteins in control-state human neuroblastoma SH-SY5Y cells. Ligand stimulation with acetyl-β-methylcholine (MeCh) was for 15 minutes before cell lysate protein extraction and titanium dioxide-mediated purification.

| **non-stimulated** | **10nM MeCh** | **100nM MeCh** | **1μM MeCh** | **10μM MeCh** | **100μM MeCh** |
| --- | --- | --- | --- | --- | --- |
| *Protein ID* | *Protein ID* | *Protein ID* | *Protein ID* | *Protein ID* | *Protein ID* |
| AAK1 | AAK1 | ADAM22 | ABI2 | AAK1 | AAK1 |
| ABI2 | ABCC6 | ADAMTS9 | ABL1 | ABCA2 | ABI2 |
| ABL1 | ABI2 | AG2 | ADAMTS13 | ABCC6 | ABL1 |
| AG2 | ACIN1 | ANAPC1 | ADD1 | ADD1 | ABL2 |
| AKAP4 | ADD1 | ARHGAP25 | AG2 | AG2 | ACIN1 |
| AMD1 | AG2 | ARID1A | ANAPC1 | ANAPC1 | AG2 |
| ANAPC1 | ANAPC1 | ATP10A | ARID1A | ANKRD24 | AKAP12 |
| APOB | ANK3 | BCLAF1 | ARL6IP4 | ARID1A | ANAPC1 |
| ARHGEF12 | AP1M1 | BRD3 | ATM | ATP2C2 | ANKRD26 |
| ARID1A | ARHGAP15 | BYSL | B1 | AVGR5 | ANP32B |
| ARL6IP4 | ARID1A | C13orf29 | B4GALNT4 | BAZ1B | ARID1A |
| ATP10A | ARL6IP4 | C17orf49 | BAZ1B | BCLAF1 | ARL6IP4 |
| ATXN2L | ARMC3 | C6orf223 | BCLAF1 | BLM | ATRX |
| B4GALNT4 | BAZ1B | CCDC86 | BRD3 | BRD3 | BAZ1B |
| BAZ1B | BCLAF1 | CCL14 | BYSL | BYSL | BCAS3 |
| BCLAF1 | BMPER | CCNY | C16orf71 | C17orf49 | BCLAF1 |
| BDP1 | BRD3 | CCNYL1 | C17orf97 | C1orf83 | BNIP3 |
| BLZF1 | BYSL | CDC2L1 | C20orf26 | C21orf70 | BRD2 |
| BRD3 | C4orf37 | CDC42EP3 | C6orf223 | C4BPB | BRD3 |
| BYSL | CARS | CDC42EP4 | CARHSP1 | C6orf223 | BYSL |
| C15orf39 | CCDC86 | CEP170 | CBX8 | C7orf50 | C11orf84 |
| C17orf49 | CCNY | CHD7 | CCDC124 | CCDC86 | C14orf145 |
| C19orf15 | CCNYL1 | CLASP2 | CCDC86 | CCNY | C5orf30 |
| C1orf26 | CD2BP2 | CLNS1A | CCNL1 | CCNYL1 | C7orf50 |
| C6orf134 | CDC2 | COBRA1 | CCNY | CDC2L1 | CAMKV |
| C6orf223 | CDC42EP4 | CSMD2 | CDC2 | CDCA2 | CCDC6 |
| C7orf50 | CDR2 | CTNNA1 | CDC42EP4 | CDKN2AIP | CCDC86 |
| CARHSP1 | CEP170 | CTR9 | CEP170 | CFHR3 | CCNL1 |
| CBX3 | CHAF1B | CYTIP | CLNS1A | CHD7 | CCNY |
| CCDC86 | CHD7 | DDX21 | CNTNAP5 | CLASP2 | CCNYL1 |
| CCDC88B | CHD8 | DDX25 | COBRA1 | CLNS1A | CDC2 |
| CCNL1 | COBRA1 | DDX6 | CRIP2 | COBRA1 | CDC2L1 |
| CCNY | CRKRS | DKC1 | CRKRS | CP | CDC42EP3 |
| CCNYL1 | CSPP1 | DKFZp779J2370 | CTR9 | CRIP2 | CDH23 |
| CDC2 | CTTN | DNAJC5 | CTTN | CSPP1 | CEP170 |
| CDC42EP3 | DBN1 | DPF2 | DBN1 | CTNNA1 | CFH |
| CDC42EP4 | DDB2 | DYNC1LI1 | DDX21 | CTR9 | COBRA1 |
| CENPC1 | DDX21 | EAP1 | DDX51 | CTTN | CRIP2 |
| CEP170 | DDX51 | EBAG9 | DDX60 | DAXX | CRKRS |
| CEP350 | DGKD | EBF1 | DKC1 | DBN1 | CTNNA2 |
| CLNS1A | DKC1 | EIF3G | DLC1 | DDX21 | CTR9 |
| COBRA1 | DNAJC5 | EIF4ENIF1 | DNAJC5 | DDX3X | CTTN |
| COL6A3 | DNMT1 | ELAVL4 | DOCK7 | DEFB132 | DBN1 |
| COMMD5 | DPF2 | ESR2 | DPF2 | DKC1 | DBNL |
| CRIP2 | DPYSL2 | ETS1 | DPYSL2 | DMAP1 | DCTD |
| CTR9 | EEF1D | EXOSC10 | DPYSL2 | DNAJC5 | DDB2 |
| CTTN | EIF3G | FAM40A | EAP1 | DNMT1 | DDX21 |
| CXorf58 | EIF4ENIF1 | FAM76B | EBAG9 | DOCK4 | DDX51 |
| DACT1 | EIF5B | FAM83H | EBF1 | DPF2 | DIDO1 |
| DBN1 | ELAVL4 | G3BP1 | EEF1D | DUSP4 | DKC1 |
| DBNL | EPN2 | GHRL | EIF3G | DYNC1LI2 | DKFZp686O16217 |
| DCDC2 | EPN3 | GRIN2C | EIF5B | EEF1D | DKFZp779J2370 |
| DDX21 | ERCC5 | HDGF | ELAVL4 | EIF3G | DMRTA2 |
| DKC1 | FAM40A | HIVEP1 | EPB41L2 | EIF5B | DNAH1 |
| DKFZp686P13170 | FAM76B | HN1 | EPM2AIP1 | ELAVL4 | DNAJC5 |
| DKFZp779J2370 | FARP1 | HNRNPH1 | EPRS | ELK1 | DOCK7 |
| DNAH1 | FGL2 | HNRPA1L3 | ERCC5 | EPRS | DPF2 |
| DNAJC5 | FIP1L1 | IBTK | FAM40A | FAM178A | DPYSL2 |
| DPF2 | FMO3 | IDH3B | FARP1 | FAM40A | DYNC1LI1 |
| DPP7 | FOXA2 | IGF2R | FHAD1 | FAM76B | EAP1 |
| DPYSL2 | G3BP1 | IGFL2 | FIP1L1 | FIP1L1 | EIF3G |
| DPYSL2 | GFPT1 | KCTD15 | GARNL1 | FRMD1 | EIF5B |
| DTD1 | GIT1 | KIAA1429 | gdf7 | G3BP1 | ELAVL4 |
| EAP1 | GLIPR2 | KIF23 | GPR110 | GBF1 | EPRS |
| EIF3G | GORASP2 | KIF4A | GPR18 | GPR110 | FAM40A |
| EIF4G3 | GRIN2C | KPNA3 | GPRIN1 | GPSM1 | FAM76B |
| EIF5B | GRLF1 | KRI1 | GRIN2C | GRLF1 | FIP1L1 |
| ELA1 | H1FX | LIMCH1 | GRLF1 | GSK3A | G3BP1 |
| ELAVL4 | HDGF | LPHN3 | H1FX | GTF3C2 | GART |
| EPB41L4B | HN1 | LRRC41 | HDGF | GYPA | GFRA4 |
| EPM2AIP1 | HNRNPD | MAP1B | HN1 | hCG_2026193 | GIT1 |
| FAM40A | HNRNPH1 | MAP2 | HNRNPC | HCN3 | GLCCI1 |
| FAM76B | HNRNPK | MAP4 | HNRNPH1 | HN1 | GPR110 |
| FARP1 | HNRNPU | MARCKS | HNRNPK | HNRNPK | GPRIN1 |
| FBN1 | HNRNPUL2 | MARCKSL1 | HNRNPU | HNRPA1L3 | GPSM1 |
| FIP1L1 | HSP90AB2P | MCM2 | HSF1 | HSP90AB2P | GSK3A |
| FRMD1 | HSPB1 | MCRS1 | HSP90AB2P | HSPB1 | GTF3C2 |
| G3BP1 | HUWE1 | MDC1 | HSPB1 | HUWE1 | H1FX |
| GAB1 | IBTK | MED1 | HUWE1 | IBTK | HDGF |
| GIT1 | IGF2R | MKI67 | IBTK | IGF2R | HN1 |
| GLCCI1 | IRF2BP2 | MTA1 | IGF2R | KCNMB1 | HNRNPD |
| GLUD1 | KCTD15 | MYEF2 | KANK2 | KCTD15 | HNRNPH1 |
| GPRIN1 | KHDRBS1 | MYLC2B | KCNH7 | KHDRBS1 | HNRNPK |
| GRIN2C | KIAA0947 | NCAM1 | KHDRBS1 | KIAA0528 | HNRNPUL2 |
| GRLF1 | KIAA1211 | NIPBL | KIAA0947 | KIAA0947 | HSP90AB2P |
| GRM3 | KIAA1429 | NKAP | KIF4A | KIAA1377 | HSPB1 |
| GSK3A | KIAA1641 | NOLC1 | KPNA3 | KIAA2030 | HSPG2 |
| GTF3C2 | KIF4A | NPDC1 | LDB1 | KIF4A | HUWE1 |
| hCG_2026193 | KLHL4 | NUCKS1 | LEMD2 | KLC4 | IBTK |
| HDGF | KNDC1 | NUMA1 | LIMCH1 | KPG_008 | IGF2R |
| HN1 | KPNA3 | NUP98 | LMNA | KPNA3 | ILF3 |
| HNRNPD | LEO1 | OR5AR1 | LRRC41 | LHCGR | IRF2BP2 |
| HNRNPH1 | LIMCH1 | PA1 | LYN | LIMCH1 | JUN |
| HNRNPU | LMNA | PARC | MAP1B | LRRC1 | KBTBD11 |
| HSP90AB2P | LSP1 | PARP1 | MAP2 | LYN | KCTD1 |
| HSPB1 | LYN | PCBP1 | MAP2K2 | MAP1B | KHDRBS1 |
| HSPG2 | MACF1 | PDLIM4 | MAP4 | MAP4 | KIAA0947 |
| HUWE1 | MAP1B | PDS5B | MAPT | MAPKBP1 | KIAA1324 |
| IBTK | MAP2 | PEA15 | MARCKS | MARCKS | KIAA1641 |
| IGF2R | MAP4 | PGM1 | MARCKSL1 | MARCKSL1 | KIAA1704 |
| KCNMB1 | MARCKS | PHOX2A | MATR3 | MCM2 | KIF21A |
| KCTD15 | MCM2 | PI4KAP2 | MCM2 | MCRS1 | KIF4A |
| KIAA0913 | MKI67 | PPAN | MDC1 | MICAL3 | KPNA3 |
| KIAA0947 | MLF2 | PRCC | MKI67 | MKI67 | LARP1 |
| KIAA1641 | MYEF2 | PROM2 | MLF2 | MRPS12 | LARP7 |
| KIAA1641 | MYH9 | PRPF4B | MST081 | MSH6 | LIMCH1 |
| KIAA2030 | MYLC2B | PTGES3 | MTIF2 | MST4 | LMNA |
| KIF21A | MYLK | PTPLAD1 | MTMR4 | MYEF2 | LYN |
| KIF4A | NCAPD3 | RB1 | MUC20 | MYLC2B | MAGEB16 |
| KPNA3 | NCL | RBM25 | MYCL1 | MYO9A | MAP1B |
| LAMC1 | NEFM | RBM39 | MYEF2 | NCAPD3 | MAP2 |
| LBR | NKAP | RCNC2 | MYH9 | NDC80 | MAP4 |
| LDB1 | NOL5A | RNF20 | MYLC2B | NEK5 | MARCKS |
| LIMCH1 | NPDC1 | RNF222 | NCAM1 | NKAP | MARCKSL1 |
| LPHN3 | NUCKS1 | RPL10 | NCAPD3 | NPDC1 | MBD4 |
| LPIN3 | NUMA1 | RPS3 | NEK5 | NUCKS1 | MCM2 |
| LRRC41 | NUP98 | RRM2 | NFIB | NUMA1 | MCRS1 |
| LRRFIP1 | OTOF | RSF1 | NKAP | NUP98 | MGC50722 |
| LYN | PAK1 | RSL1D1 | NOL5A | OR5AR1 | MKI67 |
| LZTS1 | PCTK1 | SAPS2 | NPDC1 | PAK2 | MLF2 |
| MACF1 | PDS5B | SAPS3 | NSUN2 | PCF11 | MTA1 |
| MAG | PEA15 | SEPT2 | NUCKS1 | PDZK1 | MTA2 |
| MAP1A | PGM1 | SIPA1L3 | NUMA1 | PEA15 | MYEF2 |
| MAP1B | PGRMC1 | SLC25A30 | NUP98 | PGRMC1 | MYH9 |
| MAP2 | PHOX2A | SLC35C2 | OR5AR1 | PHOX2A | MYLC2B |
| MAP2K2 | PI4KB | SMAP | PAK1 | PIGA | NCAM1 |
| MAP3K12 | PLEKHA6 | SMARCC2 | PBRM1 | PLEKHA6 | NEK4 |
| MAP4 | PPAN | SMC4 | PCBP1 | PLEKHN1 | NES |
| MAPT | PPHLN1 | SON | PCF11 | PPAN | NKAP |
| MARCKS | PRKD3 | SORBS3 | PDS5B | PRKD3 | NKX6-3 |
| MARCKSL1 | PRPF4B | SRRM1 | PEA15 | PRPF4B | NOL5A |
| MCM2 | PTPLAD1 | SSBP3 | PGM1 | PTGES3 | NPDC1 |
| MKI67 | PTPN12 | STMN1 | PGRMC1 | PTPLAD1 | NSUN2 |
| MLF2 | RAB24 | SUPT5H | PHOX2A | PTPN12 | NUCKS1 |
| MTA1 | RB1 | SYNPO | PIK3R3 | RAD9A | NUMA1 |
| MUC12 | RBM25 | SYNPO2 | PLEKHA6 | RAG1 | NUP50 |
| MUC16 | RBM39 | TERF2 | PM20D1 | RALY | NUP98 |
| MYEF2 | RBMX2 | THRAP3 | POLN | RB1 | OTOF |
| MYH9 | RIMBP2 | THSD1 | POLR1B | RBM25 | PBRM1 |
| MYLC2B | RIPK2 | TJP3 | PPHLN1 | RIPK2 | PCBP1 |
| MYO9B | RNF20 | TMPO | PROX1 | RNF20 | PDLIM4 |
| NEFM | RPS3 | TP53BP1 | PRPF4B | RNF222 | PDS5B |
| NEK5 | RRM2 | TRIP12 | PSMD2 | RPL10 | PEA15 |
| NKAP | RSF1 | TSHR | PTPLAD1 | RPS3 | PGRMC1 |
| NOL5A | RSL1D1 | TTN | PTPN12 | RRM2 | PHOX2A |
| NPDC1 | RSPRY1 | TUBA4A | PTPRF | RSF1 | PLEKHA6 |
| NUCKS1 | SAPS3 | UHRF2 | PUM2 | RSL1D1 | POLN |
| NUMA1 | SEC24A | USP42 | RAD18 | RSRC1 | POP1 |
| NUP98 | SENP7 | UVRAG | RAPTOR | SAPS2 | PPAN |
| PAF | SLTM | VAMP4 | RB1 | SAPS3 | PPHLN1 |
| PAK1 | SMAP | WDR79 | RBBP6 | SMAP | PRKD3 |
| PAPOLG | SMC4 | WHRN | RBM25 | SMARCA4 | PROX1 |
| PCNT | SNX16 | ZFC3H1 | REXO1L2P | SMARCC2 | PRPF4B |
| PDLIM4 | SON | ZNF683 | RIC3 | SMC4 | PSD3 |
| PDS5A | SORBS3 |  | RNF20 | SNRNP200 | PSIP1 |
| PDS5B | SPTBN1 |  | RPS3 | SON | PTBP1 |
| PGM1 | SRRM1 |  | RRM2 | SORBS3 | PTGES3 |
| PGRMC1 | SRRM2 |  | RSF1 | SRRM1 | PTPLAD1 |
| PHOX2A | SSBP3 |  | RSL1D1 | SRRM2 | PTPN12 |
| PIK3AP1 | STMN1 |  | SAPS3 | SSBP3 | PWWP2A |
| PIP5K3 | SUPT5H |  | SEC23A | STMN1 | RAD18 |
| PLEKHO2 | SVEP1 |  | SERPING1 | SUPT5H | RAPTOR |
| PNRC1 | SYNPO2 |  | SERPINI2 | SYNPO2 | RB1 |
| PPAN | TBC1D15 |  | SFRS11 | TBC1D5 | RBBP6 |
| PPFIBP2 | TBC1D5 |  | SGMS2 | TERF2 | RBM15 |
| PPHLN1 | TCOF1 |  | SLC35C2 | TERF2IP | RBM25 |
| PPWD1 | TERF2 |  | SLTM | THRAP3 | RBM39 |
| PRCC | TFCP2 |  | SMAP | TLE3 | RBM4 |
| PROCA1 | THRAP3 |  | SMC4 | TMPO | RBMX |
| PRPF4B | TMPO |  | SNRNP200 | TOP2B | RBMX2 |
| PTGES3 | TOP2B |  | SON | TP53BP1 | RDBP |
| PTPLAD1 | TP53BP1 |  | SORBS3 | TPI1 | RER1 |
| PTPN12 | TRAFD1 |  | SPNS2 | TPR | RFX7 |
| PTPRS | TRHR |  | SPTBN1 | TRAFD1 | RIF1 |
| PWWP2A | TRIP12 |  | SRRM1 | TRIP12 | RNF20 |
| RBBP6 | TWIST1 |  | SRRM2 | ULK2 | RPS3 |
| RBM25 | TXLNA |  | SSBP3 | URG4 | RRM2 |
| RBM39 | TXNDC11 |  | STMN1 | VAMP4 | RSF1 |
| RBMX | USP42 |  | SUPT5H | VNRL4 | RSL1D1 |
| RNF20 | WAPAL |  | SYNPO | WDR79 | RSRC2 |
| RPS3 | WHRN |  | SYNPO2 | WNT2B | RWDD3 |
| RRM2 | YBX1 |  | TCOF1 | ZFC3H1 | SAPS3 |
| RSF1 | ZC3H14 |  | TERF2 | ZNF354A | SART1 |
| RSL1D1 | ZC3H18 |  | THRAP3 |  | SEC23IP |
| RSRC2 | ZNF687 |  | TJAP1 |  | SEPT2 |
| SAMSN1 |  |  | TMEM200A |  | SLC35C2 |
| SAPS3 |  |  | TMPO |  | SLC4A4 |
| SCAF1 |  |  | TOP2B |  | SLTM |
| SCMH1 |  |  | TP53BP1 |  | SMAP |
| SEC16A |  |  | TPI1 |  | SMARCC2 |
| SEC62 |  |  | TRIP12 |  | SMC4 |
| SENP7 |  |  | TTN |  | SNRNP200 |
| SEPT2 |  |  | TUBA4A |  | SON |
| SFRS11 |  |  | TXLNA |  | SORBS3 |
| SHROOM3 |  |  | UNC13C |  | SPECC1L |
| SLC35C2 |  |  | USP24 |  | SPTBN1 |
| SLC5A10 |  |  | VAMP4 |  | SRRM1 |
| SLTM |  |  | VWA5B2 |  | SRRM2 |
| SMAP |  |  | WAPAL |  | STH |
| SMARCA4 |  |  | WHRN |  | STMN1 |
| SMARCC2 |  |  | ZC3H18 |  | STUB1 |
| SMC4 |  |  | ZC3H4 |  | SUDS3 |
| SNRNP200 |  |  | ZFHX4 |  | SUPT5H |
| SNX16 |  |  | ZFYVE19 |  | SYNPO2 |
| SON |  |  | ZNF609 |  | TBC1D15 |
| SORBS3 |  |  |  |  | TBC1D5 |
| SP6 |  |  |  |  | TCF12 |
| SPECC1L |  |  |  |  | TCOF1 |
| SPIRE1 |  |  |  |  | TERF2 |
| SPTAN1 |  |  |  |  | TFCP2 |
| SPTBN1 |  |  |  |  | THRAP3 |
| SRRM1 |  |  |  |  | THYN1 |
| SRRM2 |  |  |  |  | TMPO |
| SSBP3 |  |  |  |  | TOP2B |
| STMN1 |  |  |  |  | TP53BP1 |
| STUB1 |  |  |  |  | TPI1 |
| STX1A |  |  |  |  | TRAFD1 |
| SUPT5H |  |  |  |  | TRIM28 |
| SYNPO2 |  |  |  |  | TRIP12 |
| TBC1D15 |  |  |  |  | TTN |
| TCOF1 |  |  |  |  | TXLNA |
| TERF2 |  |  |  |  | UBAP2L |
| TERF2IP |  |  |  |  | UFD1L |
| TFCP2 |  |  |  |  | USP31 |
| THRAP3 |  |  |  |  | VAMP4 |
| TMPO |  |  |  |  | VPS33B |
| TOP2B |  |  |  |  | VSIG2 |
| TP53BP1 |  |  |  |  | WDR43 |
| TP53BP2 |  |  |  |  | WHRN |
| TPI1 |  |  |  |  | WTAP |
| TPO |  |  |  |  | ZC3H14 |
| TRAFD1 |  |  |  |  | ZC3H18 |
| TRIP12 |  |  |  |  | ZNF683 |
| TWIST1 |  |  |  |  |  |
| TXNDC11 |  |  |  |  |  |
| USP24 |  |  |  |  |  |
| USP42 |  |  |  |  |  |
| VAMP4 |  |  |  |  |  |
| WAPAL |  |  |  |  |  |
| WDR43 |  |  |  |  |  |
| WDR79 |  |  |  |  |  |
| WHRN |  |  |  |  |  |
| YBX1 |  |  |  |  |  |
| YTHDC1 |  |  |  |  |  |
| ZC3H13 |  |  |  |  |  |
| ZC3H18 |  |  |  |  |  |
| ZNF618 |  |  |  |  |  |
| ZNF683 |  |  |  |  |  |
| ZNF687 |  |  |  |  |  |
| ZNF8 |  |  |  |  |  |
| ZNF828 |  |  |  |  |  |
