# Supplementary material for: VENNTURE–A Novel Venn Diagram Investigational Tool for Multiple Pharmacological Dataset Analysis
Source: PLoS One. 2012 May 14;7(5):e36911. doi: 10.1371/journal.pone.0036911 (PMC3351456; doi:10.1371/journal.pone.0036911)
Supplement: Table S14 — Dose-dependent acetyl-β-methylcholine-stimulated phosphoproteins in CMP-state human neuroblastoma SH-SY5Y cells. Ligand stimulation, performed after pre-treatment for seven days with 10 nM continuous hydrogen peroxide treatment (CMP protocol), with acetyl-β-methylcholine (MeCh) was for 15 minutes before cell lysate protein extraction and titanium dioxide-mediated purification. (DOC) [file pone.0036911.s015.doc]

**Table S14.** Dose-dependent acetyl-β-methylcholine-stimulated phosphoproteins in CMP-state human neuroblastoma SH-SY5Y cells. Ligand stimulation, performed after pre-treatment for seven days with 10nM continuous hydrogen peroxide treatment (CMP protocol), with acetyl-β-methylcholine (MeCh) was for 15 minutes before cell lysate protein extraction and titanium dioxide-mediated purification.

| **non-stimulated** | **10nM MeCh** | **100nM MeCh** | **1μM MeCh** | **10μM MeCh** | **100μM MeCh** |
| --- | --- | --- | --- | --- | --- |
| *Protein ID* | *Protein ID* | *Protein ID* | *Protein ID* | *Protein ID* | *Protein ID* |
| AAK1 | AAK1 | AAK1 | A26C1A | AAK1 | AAK1 |
| ABCA9 | ABCF1 | AFF2 | AAK1 | ABCF1 | ABCF1 |
| ABCF3 | ABL1 | AG2 | ABCF1 | ACIN1 | ABL1 |
| ABI2 | ACIN1 | AHNAK | ABI2 | ACLY | ACIN1 |
| ABL1 | ADD1 | ANAPC1 | ACIN1 | ACTR8 | ADAM19 |
| ACACB | AG2 | ANK2 | ACLY | ADAM20 | ADAM20 |
| ACIN1 | AKAP12 | ANKRA2 | ADAM22 | ADAR | ADAMTS2 |
| ACSL3 | ARHGAP21 | AR | ADD1 | ADD1 | ADAR |
| ADD1 | ARID1A | ARID1A | ADNP | AG2 | ADD1 |
| ADFP | ATF2 | ASCC3 | AG2 | AGFG1 | AFM |
| AG2 | ATRX | ATF2 | AHDC1 | AHDC1 | AG2 |
| AKAP12 | BASP1 | ATR | AKAP12 | AKAP12 | AGFG1 |
| AKAP13 | BAT3 | BAT3 | ANAPC1 | ALOX12B | AHDC1 |
| AKT1S1 | BAZ1B | BAZ1B | APOB | ANAPC1 | AKAP13 |
| ALK | BCLAF1 | BAZ2A | ARD1A | ANK2 | AKAP2 |
| ANK1 | BRD3 | BCLAF1 | ARHGAP17 | ANKRD17 | ALPPL2 |
| ANK3 | BYSL | BIN1 | ARHGEF12 | ANLN | ANKRD30A |
| ANP32B | CAGE1 | BMS1 | ARID1A | API5 | ANKS1A |
| API5 | CAMSAP1L1 | BRD2 | ARNT | APOB | ANLN |
| APOB | CBX1 | BRD3 | ARS2 | ARHGAP25 | API5 |
| ARHGAP18 | CBX3 | BRIP1 | ASAP1 | ARHGEF12 | APOB |
| ARHGAP25 | CCDC86 | C13orf18 | ATF2 | ARID1A | APOOL |
| ARHGEF12 | CCDC88A | C14orf38 | ATP9A | ARS2 | ARFIP1 |
| ARID1A | CCNK | C17orf49 | ATRX | ATF2 | ARHGAP17 |
| ARVCF | CDC2 | C7orf47 | BAZ1B | ATP10A | ARHGEF12 |
| ASCL5 | CDC42EP4 | CAD | BCLAF1 | B4GALNT4 | ARID1A |
| ASPSCR1 | CDON | CALD1 | BMP2 | BASP1 | ARMS |
| ATF2 | CEP170 | CCDC71 | BRD3 | BAZ1B | ASH1L |
| ATOH8 | CHD3 | CCDC86 | BYSL | BBS7 | ATAD2B |
| ATP2C2 | CLASP1 | CCL14 | C11orf84 | BCLAF1 | ATF2 |
| ATRX | CRIP2 | CCNL1 | C12orf49 | BIN1 | ATP11C |
| ATXN2L | CSTF2 | CDC2 | C14orf145 | BRD3 | ATP9A |
| BAZ1B | CTTN | CEP170 | C17orf49 | BRF1 | ATRX |
| BCL11A | DBN1 | CGN | C17orf82 | BTBD8 | ATRX |
| BCLAF1 | DBNL | CHD7 | C1orf62 | C11orf84 | ATXN2L |
| BRD3 | DCX | CLASP2 | C20orf117 | C12orf42 | ATXN7 |
| BRWD1 | DDX23 | COBRA1 | C22orf30 | C14orf48 | B4GALNT4 |
| BYSL | DDX51 | CRIP2 | C2orf49 | C17orf49 | BAT3 |
| C11orf84 | DDX54 | CTTN | C3orf30 | C17orf82 | BAZ1B |
| C12orf11 | DKC1 | DAXX | C6orf174 | C1orf92 | BCL9 |
| C14orf81 | DMAP1 | DBN1 | C7orf27 | C3orf54 | BCLAF1 |
| C16orf62 | DMXL1 | DBNL | C7orf50 | C4orf47 | BMP2K |
| C17orf49 | DNAJC16 | DDX6 | C7orf54 | C6orf223 | BRD2 |
| C19orf28 | DNAJC5 | DEDD2 | C9orf84 | C7orf47 | BRD3 |
| C1orf144 | DOCK7 | DENND4A | CACHD1 | C9orf82 | BYSL |
| C1orf173 | DOS | DNTTIP2 | CACNB2 | CALD1 | C11orf84 |
| C20orf132 | DPF2 | DOC2A | CARD10 | CARHSP1 | C12orf24 |
| C21orf59 | DPP8 | DOCK7 | CARHSP1 | CBX1 | C12orf43 |
| C21orf82 | DPYSL2 | DPF2 | CCDC124 | CCDC100 | C14orf43 |
| C22orf23 | DPYSL2 | DVL2 | CCDC86 | CCDC124 | C15orf59 |
| C3orf54 | E2F8 | DYNC1LI1 | CCNK | CCDC132 | C17orf49 |
| C3orf63 | EAP1 | EAP1 | CCNY | CCDC142 | C17orf82 |
| C5orf42 | EEF1D | EIF3G | CD2BP2 | CCDC86 | C1orf64 |
| C5orf45 | EIF2A | EIF4ENIF1 | CDC2 | CCNK | C20orf30 |
| C6orf199 | EIF2AK4 | EIF4G3 | CEP170 | CCNL1 | C22orf23 |
| C6orf223 | EIF3G | EIF5B | CEP350 | CD22 | C5orf41 |
| C7orf47 | EIF4G3 | ELAVL4 | CHAF1A | CDC2 | C6orf223 |
| CAMSAP1 | EIF5B | EPB41L5 | CHAF1B | CDC42EP3 | C6orf89 |
| CAMSAP1L1 | ELAVL4 | EPRS | CHD1L | CDON | C6orf97 |
| CARHSP1 | EPM2AIP1 | ERCC5 | CHD3 | CECR2 | C7orf47 |
| CATSPER1 | EPN3 | ERRFI1 | CHD9 | CENPK | C7orf51 |
| CCDC126 | EPRS | FAM40A | CHFR | CEP170 | CABIN1 |
| CCDC86 | FAM179B | FAM54B | CHGA | CEP350 | CACNB2 |
| CCDC88C | FAM40A | FAM76B | CILP | CFL1 | CARHSP1 |
| CCNL1 | FAM54B | FNDC1 | CISH | CHAF1B | CASP8AP2 |
| CD209 | FAM76B | FZR1 | CLASP1 | CHD4 | CBX3 |
| CDC2 | FDXR | G3BP1 | CLASP2 | CHFR | CCDC124 |
| CDC2L5 | FIP1L1 | GABRA5 | CLDN15 | CHST13 | CCDC144C |
| CDC42EP4 | FMN1 | GARNL1 | COBRA1 | CIC | CCDC71 |
| CDK4 | G3BP1 | GATA1 | COIL | CILP | CCDC86 |
| CDKL3 | GJA10 | GATA3 | CPSF7 | CLASP1 | CCNK |
| CEACAM21 | GLCCI1 | GATAD2B | CRIP2 | CLASP2 | CCNL1 |
| CEACAM5 | GPR18 | GFPT1 | CSTF2 | CLEC7A | CCNYL1 |
| CEP110 | GPR52 | GINS3 | CTR9 | CNTNAP5 | CD164L2 |
| CEP170 | GPRIN1 | GLCCI1 | CTTN | COBRA1 | CD93 |
| CEP250 | GPSM1 | GPLD1 | DACT2 | COL6A3 | CDC2 |
| CHAF1B | GSK3A | GSK3A | DAXX | CRB1 | CDC42EP3 |
| CHD4 | H1FX | GTPBP1 | DBN1 | CRIP2 | CENPJ |
| CHD7 | HDGF | GUCY2C | DBNL | CRKRS | CENPT |
| CHSY3 | HIVEP3 | H1FX | DCHS1 | CSPP1 | CEP170 |
| CHTF18 | HN1 | H2AFY | DCX | CTNNB1 | CHAF1B |
| CIR | HNRNPD | HDGF | DDB2 | CTTN | CHD4 |
| CLASP1 | HNRNPH1 | HN1 | DDX21 | CYP19A1 | CHD7 |
| CLASP2 | HNRNPK | HNRNPD | DDX51 | DAXX | CHTF18 |
| CLIC1 | HNRNPUL2 | HNRNPH1 | DDX54 | DBF4 | CILP |
| CNPY2 | HSP90AB2P | HNRPA1L3 | DENND4A | DBN1 | CILP2 |
| COBRA1 | HUWE1 | HORMAD1 | DHRS3 | DBNL | CLASP1 |
| COL6A3 | IGF2R | HSP90AB2P | DHX57 | DCX | CLASP2 |
| CPS1 | ILDR1 | HSPB1 | DISP1 | DDB2 | CLIP2 |
| CRIP2 | IRF2BP2 | HSPG2 | DKC1 | DDX21 | CLPS |
| CRIPAK | IRF2BP2 | ILF3 | DKFZp686L2367 | DDX23 | COBRA1 |
| CRKRS | JMJD2B | IMPA1 | DKFZp779J2370 | DDX51 | CRIP2 |
| CSMD2 | KBTBD11 | IRF2BP2 | DMAP1 | DDX54 | CRKRS |
| CTR9 | KCNH2 | ITGA1 | DMXL1 | DDX6 | CTNNB1 |
| CTTN | KCTD15 | ITPR2 | DNAJC21 | DEDD2 | CTTN |
| DACT1 | KIAA0528 | KBTBD11 | DNAJC5 | DENND1B | CYB561 |
| DAXX | KIAA0614 | KCNN1 | DOCK7 | DGKZ | DAB2IP |
| DBN1 | KIAA0947 | KCTD15 | DPF2 | DIDO1 | DAXX |
| DBNL | KIAA1211 | KIAA0528 | DPYSL2 | DKC1 | DBN1 |
| DCHS2 | KIAA1641 | KIAA0614 | DTD1 | DKFZp686K09128 | DBNL |
| DCX | KIF21A | KIAA1641 | DUSP15 | DKFZp686L2367 | DCX |
| DDB2 | KPNA3 | KIF21A | EAP1 | DKK1 | DDB2 |
| DDX21 | LARP1 | KLC1 | EBAG9 | DLGAP2 | DDX21 |
| DDX3Y | LBR | KLC3 | EDC3 | DMXL1 | DDX51 |
| DDX51 | LCA5 | KLC4 | EEF1D | DNAH1 | DDX54 |
| DDX54 | LIG1 | LIG3 | EIF2A | DNAJC5 | DENND2A |
| DGCR14 | LIG3 | LIMA1 | EIF3G | DOCK7 | DENND5A |
| DHX57 | LIMCH1 | LIMCH1 | EIF4ENIF1 | DPF2 | DGKD |
| DIP2C | LRRC41 | LRP1 | EIF4G3 | DPYSL2 | DHRS12 |
| DKC1 | LRWD1 | LRRC41 | EIF5B | DPYSL2 | DKC1 |
| DKFZp686P13218 | MAGED2 | LRWD1 | ELAVL4 | DYNC1I2 | DKFZp451F173 |
| DMXL1 | MAP1A | MAP1A | EPB41 | EAP1 | DMXL1 |
| DNAJC5 | MAP1B | MAP1B | EPB41L4B | EBAG9 | DNAJC5 |
| DOCK7 | MAP2 | MAP2 | EPHA8 | EEF1D | DOCK7 |
| DPF2 | MAP2K2 | MAP3K2 | EPM2AIP1 | EHMT1 | DOPEY2 |
| DPYSL2 | MAP4 | MAP4 | EPN3 | EIF2A | DOS |
| DPYSL2 | MAPT | MAPT | EPRS | EIF3G | DPF2 |
| EBAG9 | MARCKS | MARCKS | ERBB2IP | EIF4ENIF1 | DYNC1I2 |
| ECM29 | MARCKSL1 | MARCKSL1 | ERCC5 | EIF5B | DYNC1LI1 |
| EDC4 | MCM2 | MASP1 | ERRFI1 | ELAVL4 | EAP1 |
| EEA1 | MECP2 | MCM2 | EXOSC5 | EMILIN2 | EBAG9 |
| EGFL7 | MPDZ | MECP2 | FAM186B | EPB41L4B | EEF1D |
| EIF2A | MST120 | MED11 | FAM40A | EPHA8 | EHHADH |
| EIF3G | MUC19 | MICALL1 | FAM48A | EPM2AIP1 | EIF2A |
| EIF4E2 | MYEF2 | MICALL2 | FAM54B | EPRS | EIF3G |
| EIF4ENIF1 | MYH9 | MKI67 | FAM62C | EPS15L1 | EIF4ENIF1 |
| EIF4G3 | MYLC2B | MSH6 | FAM76B | ESF1 | EIF4G3 |
| EIF5B | MYO9B | MTA1 | FARP1 | EXOSC10 | EIF5B |
| ELAVL4 | NCAM1 | MYEF2 | FIP1L1 | EXOSC5 | ELAVL4 |
| EPB41L5 | NCL | MYLC2B | FMN1 | FAM110B | EPAS1 |
| EPM2AIP1 | NES | MYOM1 | FOXN3 | FAM154A | EPM2AIP1 |
| EPN3 | NFATC2 | MYST2 | FYTTD1 | FAM178A | EPN3 |
| EPR1 | NHSL1 | NADK | G3BP1 | FAM40A | EPRS |
| EPRS | NKAP | NCAM1 | GAB2 | FAM54B | EPS15L1 |
| ESF1 | NOL5 | NCL | GABRA4 | FAM76B | ETV2 |
| FAM110B | NOL5A | NIPBL | GALR1 | FARP1 | EXOSC10 |
| FAM178A | NRBP1 | NKAP | GLCCI1 | FBN1 | EXOSC5 |
| FAM186A | NSFL1C | NOL5A | GPATCH8 | FCAMR | EZH2 |
| FAM40A | NSUN2 | NONO | GPRIN1 | FCAR | FAHD1 |
| FAM54B | NUCKS1 | NOP2 | GPSM1 | FCHO1 | FAM186A |
| FAM65B | NUMA1 | NPDC1 | GRM3 | FMN1 | FAM40A |
| FAM76B | NUP214 | NR1H4 | GSK3A | G3BP1 | FAM54B |
| FARP1 | OR4D2 | NUMA1 | GTF3C2 | G3BP2 | FAM71B |
| FBXL17 | PBRM1 | NXF1 | GUCY2C | GAB2 | FAM76B |
| FBXO34 | PCBP1 | PAK4 | GZMK | GAS2L3 | FARP1 |
| FBXO40 | PCM1 | PALB2 | H1FX | GATAD2B | FBN2 |
| FHAD1 | PDS5A | PALM | HBA1 | GIGYF2 | FIGN |
| FIP1L1 | PDS5B | PALM3 | HDGF | GJA1 | FLNA |
| FLJ00397 | PGM1 | PCM1 | HN1 | GLA | FMO3 |
| FLJ36046 | PHOX2A | PDS5A | HNRNPD | GLCCI1 | FOXK2 |
| FMNL3 | PI4KB | PDS5B | HNRNPH1 | GON4L | FRYL |
| FNBP4 | PLCB4 | PGM1 | HNRNPK | GP2 | FSTL3 |
| FNDC3B | PPHLN1 | PHOX2A | HNRNPUL2 | GPATCH8 | G3BP1 |
| G3BP1 | PPIL4 | PLA2G6 | HNRPA1L3 | GPR126 | GAB2 |
| GAPDH | PRKACA | PLEKHA5 | HORMAD1 | GPRIN1 | GABRA5 |
| GAPVD1 | PROX1 | PLEKHH2 | HSP90AB2P | GPSM1 | GCDH |
| GARNL1 | PRPF38A | PPP1R10 | HSPB1 | GRIN2C | GFPT1 |
| GARNL2P | PRPF4B | PRKD3 | HSPG2 | GRM3 | GLCCI1 |
| gdf7 | PSIP1 | PRPF4B | HSPH1 | GSE1 | GLI2 |
| GGCT | PTDSS2 | PRPSAP1 | HUWE1 | GSK3A | GLI3 |
| GIGYF2 | PTPLAD1 | PSMC1 | IBTK | GTF3C2 | GP2 |
| GLCCI1 | PUM2 | PTBP1 | IGF2R | H1FX | GPATCH8 |
| GPATCH8 | RAB12 | PTPLAD1 | ILF3 | H2AFB1 | GPR116 |
| GPR116 | RAI1 | RAB12 | INE1 | HARS2 | GPRIN1 |
| GPR82 | RBBP6 | RAPTOR | IRF2BP1 | HCFC1 | GPSM1 |
| GPRIN1 | RBM25 | RBBP6 | IRF2BP2 | hCG_2015407 | GRM3 |
| GPSM1 | RBM33 | RBM33 | JMJD1C | hCG_2026193 | GSK3A |
| GRLF1 | RBM39 | RBM39 | JMJD2C | hCG_2044975 | GTF3C2 |
| GRM3 | RBM6 | RBMX2 | KCNQ5 | HCRTR2 | GTPBP1 |
| GSK3A | RBMX | RGS12 | KHDRBS1 | HDGF | H1FX |
| GTF2I | RBMX2 | RNF20 | KIAA0528 | HEG1 | H2AFY |
| GTF3C2 | RP13 | RPS17 | KIAA0614 | HIRIP3 | HCFC1 |
| GTF3C4 | RPN2 | RPS3 | KIAA1211 | HLA-C | HDGF |
| HDGF | RPS3 | RRM2 | KIAA1429 | HMGA1 | HDGFRP2 |
| HIGD1B | RRM2 | RSF1 | KIF21A | HN1 | HDMCP |
| HIRIP3 | RSF1 | RTN4 | KIF25 | HNRNPC | HECW1 |
| HIST2H3A | RSL1D1 | SAMD1 | KIF4A | HNRNPD | HIRIP3 |
| HIVEP1 | RSRC2 | SAMHD1 | KLC4 | HNRNPH1 | HIST2H3A |
| HN1 | SAMD1 | SCRIB | KLF3 | HNRNPK | HJURP |
| HNRNPD | SCRIB | SEC16A | KPNA3 | HNRPA1L3 | HMGA1 |
| HNRNPH1 | SENP7 | SEC24D | KTN1 | HSP90AB2P | HN1 |
| HNRNPK | SEPT2 | SEPT2 | LARP5 | HSPA12A | HNRNPD |
| HNRNPUL2 | SERBP1 | SEPT5 | LARP7 | HSPB1 | HNRNPH1 |
| HNRPA1L3 | SFRS11 | SIPA1L1 | LBA1 | HSPG2 | HNRNPK |
| HORMAD1 | SLC35C2 | SLC35C2 | LBR | HTATSF1 | HNRNPU |
| HPN | SLTM | SLC44A1 | LDB1 | HUWE1 | HNRNPUL1 |
| HPS6 | SMAP | SLTM | LIG1 | IBTK | HNRPA1L3 |
| HRH4 | SMARCC2 | SMAP | LIMA1 | IGF2R | HORMAD1 |
| HSP90AB2P | SMC4 | SMARCC2 | LIMCH1 | IGFL2 | HSP90AB2P |
| HSPA12A | SMOC1 | SON | LMNA | ILDR1 | HSPB1 |
| HSPB1 | SNRNP200 | SORBS3 | LOXHD1 | ILF3 | HSPC105 |
| HUWE1 | SNTG1 | SPAG9 | LQK1 | INSM2 | HSPG2 |
| IBTK | SON | SPTAN1 | LRRC37A | IRF2BP1 | HTATSF1 |
| IGF2BP3 | SPTBN1 | SPTBN1 | LRRC41 | IRF2BP2 | HUWE1 |
| IGF2R | SRRM1 | SRRM1 | LRRFIP2 | IRF2BP2 | IBTK |
| ILDR1 | SRRM2 | SRRM2 | LRWD1 | JUN | IGF2R |
| IQGAP1 | ST5 | STK11IP | LY6H | KARP-1 | ILF3 |
| IRF2BP1 | STMN1 | STMN1 | LYN | KBTBD11 | INE1 |
| IRF2BP2 | STUB1 | STUB1 | MAP1A | KCNH2 | IQGAP1 |
| ITGB3 | SUDS3 | SUPT5H | MAP1B | KCNH7 | IRF2BP1 |
| JMJD2C | SUPT5H | SYNPO | MAP2 | KCNT1 | IRF2BP2 |
| KBTBD11 | SYDE2 | SYNPO2 | MAP4 | KCTD1 | IRF2BP2 |
| KCNH2 | SYNPO | TACC2 | MAPKAP1 | KHDRBS1 | ITGA9 |
| KCNN1 | SYNPO2 | TBC1D5 | MAPT | KIAA0256 | ITGB5 |
| KCNT1 | TAF3 | TCOF1 | MARCKS | KIAA0528 | ITIH5L |
| KCTD15 | TCOF1 | TERF2 | MARCKSL1 | KIAA0614 | JAKMIP2 |
| KHDRBS1 | TERF2 | THRAP3 | MARK4 | KIAA1211 | KBTBD11 |
| KIAA0355 | TFCP2 | TMEM45A | MCM2 | KIAA1539 | KCNH2 |
| KIAA0528 | THRAP3 | TMPO | MECP2 | KIAA1641 | KCTD15 |
| KIAA0947 | TMEM44 | TOP2B | MFF | KIF21A | KHDRBS1 |
| KIAA1109 | TMPO | TP53BP1 | MKI67 | KIF23 | KIAA0284 |
| KIAA1211 | TNKS1BP1 | TP53BP2 | MMP10 | KIF4A | KIAA0467 |
| KIAA1370 | TOP2B | TPI1 | MMP14 | KIF9 | KIAA0528 |
| KIAA1641 | TP53BP1 | TRAFD1 | MNDA | KLHDC6 | KIAA0614 |
| KIAA1656 | TPD52L2 | TTN | MPHOSPH1 | KPNA3 | KIAA1211 |
| KIAA1704 | TPI1 | TUBA4A | MRPS2 | KRT26 | KIAA1641 |
| KIAA2030 | TRAFD1 | USP42 | MTA1 | LACE1 | KIAA1704 |
| KIF21A | TRIM3 | VPS13C | MUC19 | LARGE | KIF21A |
| KIF23 | TRIP12 | VWA3B | MYBBP1A | LHX2 | KIF23 |
| KIF4A | TTBK2 | WAPAL | MYEF2 | LIG3 | KIF26B |
| KIF5A | TTN | ZC3H13 | MYH9 | LIMA1 | KIF4A |
| KLC4 | TUBA4A | ZC3H18 | MYLC2B | LIMCH1 | KLC4 |
| KLF11 | TWIST1 | ZMYND8 | MYO9B | LMNA | KLKB1 |
| KLHL13 | TXLNA | ZNF236 | NCAM1 | LRRC41 | KRT3 |
| KLHL9 | UBAP2L | ZNF391 | NCL | LRWD1 | KTN1 |
| KPNA3 | USP24 | ZNF683 | NCM | LSM11 | LARGE |
| KRTAP5-4 | USP42 | ZNF687 | NDEL1 | LYN | LARP1 |
| LARP1 | VAX2 | ZNF828 | NECAB2 | MACF1 | LEMD2 |
| LCORL | WAPAL |  | NEFH | MAP1A | LEO1 |
| LEO1 | WDR17 |  | NEFM | MAP1B | LIG1 |
| LIMCH1 | WDR43 |  | NES | MAP2 | LIG3 |
| LKAP | XPO5 |  | NIPBL | MAP2K2 | LIMA1 |
| LMNA | YRDC |  | NKAP | MAP3K15 | LIMCH1 |
| LPHN3 | ZC3H18 |  | NLRP9 | MAP4 | LMNA |
| LRRC41 | ZFP91 |  | NOL5 | MAP7D1 | LOC402468 |
| LRRC9 | ZFPM2 |  | NOL5A | MAPKAP1 | LPHN3 |
| LRRFIP2 | ZKSCAN1 |  | NONO | MAPT | LRGUK |
| LSM14A | ZNF534 |  | NOP2 | MARCKS | LRRC27 |
| LST8 | ZNF608 |  | NPDC1 | MARCKSL1 | LRRC41 |
| LYN | ZNF683 |  | NPS | MARE | LRRFIP2 |
| MACF1 | ZNF828 |  | NSFL1C | MATR3 | LRWD1 |
| MAGEB18 |  |  | NSUN2 | MAVS | LUZPP1 |
| MAP1A |  |  | NUCKS1 | MCM2 | LYN |
| MAP1B |  |  | NUMA1 | MECP2 | MAGI2 |
| MAP1S |  |  | NUP153 | MGC50722 | MAP1A |
| MAP2 |  |  | NUP214 | MKI67 | MAP1B |
| MAP2K2 |  |  | NUP50 | MLF2 | MAP1S |
| MAP3K15 |  |  | NUP98 | MPHOSPH1 | MAP2 |
| MAP4 |  |  | ODF2L | MSH5 | MAP2K2 |
| MAPT |  |  | PALM3 | MSH6 | MAP4 |
| MARCKS |  |  | PCBP1 | MTA1 | MAPKAP1 |
| MARCKSL1 |  |  | PCM1 | MUC16 | MAPT |
| MARK3 |  |  | PDGFC | MXRA7 | MARCKS |
| MCM2 |  |  | PDLIM4 | MYCBP2 | MARCKSL1 |
| MCOLN3 |  |  | PDS5A | MYEF2 | MATR3 |
| MED12L |  |  | PDS5B | MYH9 | MAVS |
| MGC50722 |  |  | PEA15 | MYLC2B | MCM2 |
| MID2 |  |  | PGK1 | MYST2 | MDN1 |
| MKI67 |  |  | PGM1 | NARS | MECP2 |
| MLF2 |  |  | PGRMC1 | Nav1.5 | MED13 |
| MPP7 |  |  | PHC1 | NCAM1 | MID2 |
| MTA1 |  |  | PHIP | NCAPD3 | MKI67 |
| MUC1 |  |  | PHOX2A | NCL | MLF2 |
| MXRA5 |  |  | PI4KB | NDUFA10 | MOSPD1 |
| MYEF2 |  |  | PIGA | NEFM | MPHOSPH9 |
| MYH9 |  |  | PLEKHA6 | NES | MUC19 |
| MYLC2B |  |  | PNMAL2 | NFATC2 | MUC6 |
| MYO5B |  |  | PNN | NGLY1 | MYB |
| NACAD |  |  | pp14450 | NKAP | MYEF2 |
| NARG2 |  |  | PPAN | NOL5A | MYH15 |
| NCAM1 |  |  | PPHLN1 | NOL8 | MYH16 |
| NCL |  |  | PPP1R10 | NONO | MYH9 |
| NCM |  |  | PPP1R12C | NOP2 | MYLC2B |
| NCOR2 |  |  | PPP1R16A | NPDC1 | MYO9B |
| NDUFA10 |  |  | PRKD3 | NUCKS1 | NAALAD2 |
| NEFM |  |  | PROX1 | NUMA1 | NACAD |
| NEK5 |  |  | PRPF38A | NUP153 | NALCN |
| NES |  |  | PRPF38B | NUP214 | NCAM1 |
| NET1 |  |  | PRPF4B | NUP50 | NCBP1 |
| NFX1 |  |  | PSRC1 | NUP98 | NCL |
| NKAP |  |  | PTDSS2 | OLIG3 | NCM |
| NKAPL |  |  | PTPLAD1 | OR5AR1 | NCOA2 |
| NOL5A |  |  | PTPN4 | ORC6L | NCOR1 |
| NOL8 |  |  | PUM2 | OTOF | NDEL1 |
| NOP2 |  |  | PYGO2 | OXCT2 | NEFM |
| NOTCH3 |  |  | RAB24 | PA1 | NEK5 |
| NPDC1 |  |  | RAD18 | PAK4 | NES |
| NRAP |  |  | RAI1 | PCBP1 | NEXN |
| NRG2 |  |  | RALY | PCM1 | NFS1 |
| NSUN2 |  |  | RANBP2 | PCTK1 | NIPBL |
| NUCKS1 |  |  | RASAL2 | PDLIM4 | NKAP |
| NUMA1 |  |  | RBBP6 | PDS5B | NOL5 |
| NUP160 |  |  | RBM25 | PEA15 | NOL5A |
| NUP98 |  |  | RBM26 | PEX26T35insC | NONO |
| ODZ1 |  |  | RBM39 | PGM1 | NPDC1 |
| OTOF |  |  | RBMX | PGRMC1 | NRD1 |
| PA1 |  |  | RCNC2 | PHIP | NSFL1C |
| PAEP |  |  | RDBP | PHOX2A | NSUN2 |
| PAK1 |  |  | RER1 | PIK3C2A | NT5C2 |
| PARP1 |  |  | RFX5 | PIWIL3 | NUCKS1 |
| PCBP1 |  |  | RIF1 | PLEKHA3 | NUFIP1 |
| PCF11 |  |  | RIOK3 | PLEKHA6 | NUFIP2 |
| PCM1 |  |  | RIPK2 | PNN | NUMA1 |
| PDE8B |  |  | RNF20 | pp10472 | NUP153 |
| PDLIM4 |  |  | RNF40 | PPAN | NUP214 |
| PDS5B |  |  | ROCK1 | PPHLN1 | NUP50 |
| PEA15 |  |  | RPN2 | PPIL4 | NUP98 |
| PECAM1 |  |  | RPS17 | PPP1R10 | OAS1 |
| PEX6 |  |  | RPS3 | PRKACA | ODF2L |
| PGM1 |  |  | RRM2 | PRKD3 | OR10J6P |
| PGRMC1 |  |  | RRP12 | PRKRA | OTOF |
| PHACTR2 |  |  | RSF1 | PROCA1 | PABPN1 |
| PHLDB1 |  |  | RSL1D1 | PROX1 | PAG1 |
| PHOX2A |  |  | RSRC1 | PRPF38A | PAK2 |
| PI4KB |  |  | SAMHD1 | PRPF4B | PARG |
| PIAS1 |  |  | SAPS3 | PSIP1 | PARP1 |
| PKN1 |  |  | SART1 | PSMD2 | PARS2 |
| PLCB4 |  |  | SCRIB | PTCH2 | PCBP1 |
| PLCG1 |  |  | SEMA6D | PTDSS2 | PCF11 |
| PLEKHA6 |  |  | SEPT2 | PTMAP4 | PCM1 |
| PLEKHO2 |  |  | SEPT7 | PTPLAD1 | PCSK9 |
| PNN |  |  | SERBP1 | PTPN13 | PDLIM4 |
| PPAN |  |  | SERPINB8 | PTPRS | PDS5A |
| PPIL4 |  |  | SFRS11 | PYGO2 | PDS5B |
| PPP2R4 |  |  | SFRS17A | RAB11FIP5 | PEA15 |
| PROM2 |  |  | SGPP1 | RAB24 | PEX1 |
| PROX1 |  |  | SHISA2 | RANBP10 | PGD |
| PRPF4B |  |  | SHROOM4 | RAPTOR | PGM1 |
| PSIP1 |  |  | SIPA1L1 | RBBP6 | PGRMC1 |
| PTGES3 |  |  | SIPA1L3 | RBM25 | PHOX2A |
| PTPLAD1 |  |  | SKIV2L | RBM33 | PHRF1 |
| PTPN12 |  |  | SLC1A4 | RBM39 | PHYHIPL |
| PUM2 |  |  | SLC25A2 | RBM4 | PI4KB |
| R3HDM1 |  |  | SLC35C2 | RBM6 | PIPSL |
| RAB12 |  |  | SLC9A5 | RBMX | PLCG1 |
| RAB24 |  |  | SLTM | RBMX2 | PLD2 |
| RAB4B |  |  | SMAP | RCNC2 | PLEKHH1 |
| RALY |  |  | SMARCA4 | REXO1L2P | PNN |
| RANBP2 |  |  | SMARCC1 | RGNEF | POGZ |
| RBBP6 |  |  | SMARCC2 | RGS7 | POM121L1 |
| RBM23 |  |  | SMC4 | RNF34 | PPAN |
| RBM25 |  |  | SNRNP200 | RPL23A | PPIL4 |
| RBM39 |  |  | SNRNP70 | RPRD2 | PPP1R10 |
| RBM44 |  |  | SON | RPS17 | PPP1R12C |
| RBMX |  |  | SORBS3 | RPS3 | PRKACA |
| RBMX2 |  |  | SPECC1L | RRM2 | PRKD3 |
| RECQL4 |  |  | SPTBN1 | RSF1 | PROM2 |
| RER1 |  |  | SRRM1 | RSL1D1 | PROX1 |
| RGS18 |  |  | SRRM2 | RSPRY1 | PRPF4B |
| RHBDF2 |  |  | SSBP3 | RSRC2 | PSMA3 |
| RIPK2 |  |  | STK10 | RTN4 | PSMB7 |
| RNF20 |  |  | STK11IP | RUFY4 | PSMD2 |
| ROCK1 |  |  | STMN1 | S1PR3 | PTGES3 |
| RP11-191L9.1 |  |  | STUB1 | SAMHD1 | PTPLAD1 |
| RPS17 |  |  | SUPT5H | SAPS3 | PTPRS |
| RPS3 |  |  | SUPV3L1 | SART1 | PUM2 |
| RRM2 |  |  | SYNE2 | SCEL | PWP2 |
| RSF1 |  |  | SYNPO | SCRIB | PYGO2 |
| RSL1D1 |  |  | SYNPO2 | SCUBE2 | RAB12 |
| RSRC2 |  |  | TAGLN2 | SEC16A | RAB37 |
| RTN4 |  |  | TCEA1 | SEPT2 | RAPTOR |
| SAMHD1 |  |  | TCOF1 | SEPT7 | RAVER1 |
| SAMSN1 |  |  | TERF2 | SETBP1 | RBBP6 |
| SCAF1 |  |  | TERF2IP | SFRS11 | RBM10 |
| SCN9A |  |  | TEX14 | SFRS2B | RBM25 |
| SCRIB |  |  | TFCP2 | SFRS9 | RBM39 |
| SDCCAG8 |  |  | THRAP3 | SGK269 | RBMX |
| SEC16A |  |  | TJAP1 | SGMS2 | RDBP |
| SEC23A |  |  | TLL2 | SLC31A2 | RFX4 |
| SEPHS2 |  |  | TMEM95 | SLC35E1 | RIF1 |
| SEPT2 |  |  | TMPO | SLC35F5 | RNF20 |
| SERBP1 |  |  | TNRC6A | SLTM | RNF31 |
| SF3B1 |  |  | TOP2B | SMAP | RPRD1B |
| SFRS11 |  |  | TOR1B | SMARCA5 | RPRD2 |
| SGIP1 |  |  | TP53BP1 | SMARCC2 | RPS17 |
| SIPA1L1 |  |  | TPD52L2 | SMC4 | RPS3 |
| SLC35C2 |  |  | TPI1 | SON | RRM2 |
| SLC5A1 |  |  | TRAFD1 | SORBS3 | RSF1 |
| SLTM |  |  | TRIP12 | SPTBN1 | RSL1D1 |
| SMAD5 |  |  | TSPY2 | SR140 | RSPRY1 |
| SMAP |  |  | TWIST1 | SRFBP1 | RSRC1 |
| SMARCA4 |  |  | TXLNA | SRRM1 | RSRC2 |
| SMARCC2 |  |  | U2AF2 | SRRM2 | RTN4 |
| SMC4 |  |  | UBAP2L | SSBP3 | SAMD1 |
| SNRNP200 |  |  | UIMC1 | STK11IP | SAMHD1 |
| SNRNP70 |  |  | USP42 | STMN1 | SAPS3 |
| SON |  |  | USP6 | STUB1 | SART1 |
| SORBS3 |  |  | VN1R5 | STX18 | SASH1 |
| SPAG9 |  |  | VPS11 | SUPT5H | SCAMP3 |
| SPTAN1 |  |  | WAPAL | SUPT7L | SCN1A |
| SPTBN1 |  |  | WDR43 | SUPV3L1 | SCRIB |
| SR140 |  |  | WDR44 | SYNPO | SEC16A |
| SRRM1 |  |  | WDR79 | SYNPO2 | SEC22B |
| SRRM2 |  |  | WHRN | TAGLN2 | SEPT2 |
| SSBP3 |  |  | XRN1 | TBC1D5 | SEPT7 |
| STK10 |  |  | YRDC | TCEA1 | SERPINB8 |
| STMN1 |  |  | ZC3H13 | TCF20 | SERPINI2 |
| STUB1 |  |  | ZC3H14 | TCF3 | SFRS11 |
| SUDS3 |  |  | ZC3H18 | TCOF1 | SFRS9 |
| SUPT5H |  |  | ZDHHC5 | TDRD5 | SH2D3C |
| SYNE2 |  |  | ZFR | TERF2 | SHOC2 |
| SYNPO |  |  | ZKSCAN1 | TERF2IP | SKIV2L |
| SYNPO2 |  |  | ZMYM4 | TET1 | SLC25A24 |
| SYTL2 |  |  | ZNF318 | TFCP2 | SLC35C2 |
| TAF5 |  |  | ZNF474 | TGFBRAP1 | SLC35E1 |
| TAGLN2 |  |  | ZNF609 | THRAP3 | SLC5A10 |
| TBC1D10B |  |  | ZNF663 | TLL2 | SLTM |
| TBC1D15 |  |  | ZNF683 | TMEM86A | SLU7 |
| TCEA1 |  |  | ZNF687 | TMPO | SMAP |
| TCF20 |  |  | ZNF828 | TNKS1BP1 | SMARCA2 |
| TCOF1 |  |  |  | TNRC6A | SMARCA4 |
| TERF2 |  |  |  | TOP2B | SMARCA5 |
| TFCP2 |  |  |  | TOR1AIP1 | SMARCC2 |
| TFG |  |  |  | TP53BP1 | SMC4 |
| THRAP3 |  |  |  | TPI1 | SMTN |
| TJP2 |  |  |  | TPR | SNRNP200 |
| TLE3 |  |  |  | TRAFD1 | SNX30 |
| TM9SF4 |  |  |  | TRAIP | SON |
| TMEM119 |  |  |  | TRIM28 | SORBS3 |
| TMEM131 |  |  |  | TRIM29 | SPAG1 |
| TMPO |  |  |  | TRIM3 | SPAG9 |
| TNC |  |  |  | TRIP12 | SPTBN1 |
| TOM1L2 |  |  |  | TRPS1 | SPTBN5 |
| TOP2A |  |  |  | TUBA4A | SR140 |
| TOP2B |  |  |  | TXLNA | SRRM1 |
| TP53BP1 |  |  |  | TXNIP | SRRM2 |
| TPI1 |  |  |  | UBAP2L | SSBP3 |
| TRAFD1 |  |  |  | UBE2O | ST6GALNAC1 |
| TRDN |  |  |  | UFD1L | STARD7 |
| TRIP12 |  |  |  | UHRF2 | STK10 |
| TRMT5 |  |  |  | ULK1 | STK11IP |
| TSC2 |  |  |  | USP42 | STMN1 |
| TTC17 |  |  |  | VANGL2 | STUB1 |
| TTN |  |  |  | VPS26B | SUDS3 |
| TUBA4A |  |  |  | WAPAL | SUPT5H |
| TWIST1 |  |  |  | WDR43 | SVEP1 |
| TXLNA |  |  |  | WDR77 | SYCP2 |
| U2AF2 |  |  |  | WHRN | SYNPO |
| UBAP2L |  |  |  | XRN2 | SYNPO2 |
| ULK2 |  |  |  | YSK4 | SYNPO2L |
| UPF3B |  |  |  | ZC3H18 | TAGLN2 |
| USP24 |  |  |  | ZC3HC1 | TANC1 |
| USP31 |  |  |  | ZFAT | TBC1D5 |
| USP42 |  |  |  | ZFC3H1 | TCEA1 |
| USP45 |  |  |  | ZKSCAN1 | TCOF1 |
| VAMP4 |  |  |  | ZMYM4 | TERF2 |
| VILL |  |  |  | ZNF141 | TERF2IP |
| WAPAL |  |  |  | ZNF509 | TEX9 |
| WDR43 |  |  |  | ZNF608 | TFR2 |
| WDR79 |  |  |  | ZNF609 | THRAP3 |
| WHRN |  |  |  | ZNF644 | THSD7A |
| XPO1 |  |  |  | ZNF655 | TLL2 |
| YARS |  |  |  | ZNF683 | TMEM132C |
| YBX1 |  |  |  | ZNF687 | TMEM39B |
| ZC3H12B |  |  |  | ZNF73 | TMPO |
| ZFHX2 |  |  |  | ZNF828 | TMPO |
| ZKSCAN1 |  |  |  | ZNF841 | TOP2B |
| ZMYM4 |  |  |  |  | TP53BP1 |
| ZNF174 |  |  |  |  | TPD52L2 |
| ZNF185 |  |  |  |  | TPI1 |
| ZNF518B |  |  |  |  | TRAFD1 |
| ZNF652 |  |  |  |  | TRIM28 |
| ZNF683 |  |  |  |  | TRIM3 |
| ZNF828 |  |  |  |  | TRIP12 |
| ZNF831 |  |  |  |  | TSGA10 |
| ZNF91 |  |  |  |  | TTC27 |
|  |  |  |  |  | TTN |
|  |  |  |  |  | TWIST1 |
|  |  |  |  |  | TXLNA |
|  |  |  |  |  | UBAP2L |
|  |  |  |  |  | UBE2O |
|  |  |  |  |  | UFD1L |
|  |  |  |  |  | UIMC1 |
|  |  |  |  |  | USP24 |
|  |  |  |  |  | USP42 |
|  |  |  |  |  | UVRAG |
|  |  |  |  |  | WAPAL |
|  |  |  |  |  | WDR43 |
|  |  |  |  |  | WDR79 |
|  |  |  |  |  | WHRN |
|  |  |  |  |  | WHSC2 |
|  |  |  |  |  | WWC3 |
|  |  |  |  |  | ZBTB1 |
|  |  |  |  |  | ZC3H12B |
|  |  |  |  |  | ZC3H14 |
|  |  |  |  |  | ZC3H18 |
|  |  |  |  |  | ZCWPW1 |
|  |  |  |  |  | ZFP36 |
|  |  |  |  |  | ZFP91 |
|  |  |  |  |  | ZKSCAN1 |
|  |  |  |  |  | ZMYM2 |
|  |  |  |  |  | ZMYM4 |
|  |  |  |  |  | ZNF296 |
|  |  |  |  |  | ZNF318 |
|  |  |  |  |  | ZNF41 |
|  |  |  |  |  | ZNF518B |
|  |  |  |  |  | ZNF638 |
|  |  |  |  |  | ZNF671 |
|  |  |  |  |  | ZNF683 |
|  |  |  |  |  | ZNF687 |
|  |  |  |  |  | ZNF735 |
|  |  |  |  |  | ZNF828 |
