# Supplementary material for: VENNTURE–A Novel Venn Diagram Investigational Tool for Multiple Pharmacological Dataset Analysis
Source: PLoS One. 2012 May 14;7(5):e36911. doi: 10.1371/journal.pone.0036911 (PMC3351456; doi:10.1371/journal.pone.0036911)
Supplement: Table S15 — Dose-unique MeCh-stimulated protein phosphorylation in control-state SH-SY5Y cells. The proteins uniquely phosphorylated at the specified MeCh dose only in control-state SH-SY5Y cells are indicated by their protein symbol as well as Uniprot accession number. (DOC) [file pone.0036911.s016.doc]

**Table S15.** Dose-unique MeCh-stimulated protein phosphorylation in control-state SH-SY5Y. The proteins uniquely phosphorylated at the specified MeCh dose only in control-state SH-SY5Y cells are indicated by their protein symbol as well as Uniprot accession number.

| **Protein Identification** | **Symbol** | **Accession** |
| --- | --- | --- |
| **10nM MeCh** |  |  |
| ankyrin 3, node of Ranvier (ankyrin G) | ANK3 | Q12955 |
| Rho GTPase activating protein 15 | ARHGAP15 | Q8IXX1 |
| armadillo repeat containing 3 | ARMC3 | Q8N7B0 |
| BMP binding endothelial regulator | BMPER | Q8N8U9 |
| chromosome 4 open reading frame 37 | C4orf37 | Q8N412 |
| cysteinyl-tRNA synthetase | CARS | Q5HYE4 |
| CD2 (cytoplasmic tail) binding protein 2 | CD2BP2 | Q9ULP2 |
| cerebellar degeneration-related protein 2, 62kDa | CDR2 | Q01850 |
| chromatin assembly factor 1, subunit B (p60) | CHAF1B | Q13112 |
| chromodomain helicase DNA binding protein 8 | CHD8 | Q6DKH9 |
| diacylglycerol kinase, delta 130kDa | DGKD | Q16760 |
| epsin 2 | EPN2 | Q9H7Z2 |
| epsin 3 | EPN3 | A8K6J3 |
| fibrinogen-like 2 | FGL2 | Q8WWE4 |
| flavin containing monooxygenase 3 | FMO3 | B2R816 |
| forkhead box A2 | FOXA2 | Q96DF7 |
| glutamine-fructose-6-phosphate transaminase 1 | GFPT1 | Q8WYR5 |
| GLI pathogenesis-related 2 | GLIPR2 | Q8N2S6 |
| golgi reassembly stacking protein 2, 55kDa | GORASP2 | Q9UFW4 |
| KIAA1211 | KIAA1211 | Q9ULK9 |
| kelch-like 4 (Drosophila) | KLHL4 | Q9Y3J5 |
| kinase non-catalytic C-lobe domain (KIND) containing 1 | KNDC1 | Q8TEE5 |
| Leo1, Paf1/RNA polymerase II complex component, homolog (S. cerevisiae) | LEO1 | Q96N99 |
| lymphocyte-specific protein 1 | LSP1 | Q16004 |
| myosin light chain kinase | MYLK | Q6LAL4 |
| nucleolin | NCL | Q8NB06 |
| PCTK1 protein | PCTK1 | BC009852 |
| RAB24, member RAS oncogene family | RAB24 | Q9HAG2 |
| RIMS binding protein 2 | RIMBP2 | Q96ID2 |
| ring finger and SPRY domain containing 1 | RSPRY1 | Q6UX21 |
| sushi, von Willebrand factor type A, EGF and pentraxin domain containing 1 | SVEP1 | B3KQM1 |
| thyrotropin-releasing hormone receptor | TRHR | Q2M339 |
|  |  |  |
| **100nM MeCh** |  |  |
| ADAM metallopeptidase domain 22 | ADAM22 | Q75MS7 |
| ADAM metallopeptidase with thrombospondin type 1 motif, 9 | ADAMTS9 | A1L4L0 |
| Rho GTPase activating protein 25 | ARHGAP25 | Q53QF7 |
| chromosome 13 open reading frame 29 | C13orf29 | Q8IVM7 |
| chemokine (C-C motif) ligand 14; chemokine (C-C motif) ligand 15 | CCL14 | B2RU34 |
| CUB and Sushi multiple domains 2 | CSMD2 | Q53TY4 |
| cytohesin 1 interacting protein | CYTIP | Q15630 |
| DEAD (Asp-Glu-Ala-Asp) box polypeptide 25 | DDX25 | Q9UHL0 |
| DEAD (Asp-Glu-Ala-Asp) box polypeptide 6 | DDX6 | Q8IV96 |
| estrogen receptor 2 (ER beta) | ESR2 | Q9UHD3 |
| v-ets erythroblastosis virus E26 oncogene homolog 1 (avian) | ETS1 | Q14278 |
| exosome component 10 | EXOSC10 | Q59G73 |
| family with sequence similarity 83, member H | FAM83H | Q6ZRV2 |
| ghrelin/obestatin prepropeptide | GHRL | A8CF38 |
| human immunodeficiency virus type I enhancer binding protein 1 | HIVEP1 | Q5VW60 |
| isocitrate dehydrogenase 3 (NAD+) beta | IDH3B | O95106 |
| IGF-like family member 2 | IGFL2 | Q6B9Z3 |
| kinesin family member 23 | KIF23 | Q8WVP0 |
| KRI1 homolog (S. cerevisiae) | KRI1 | Q9BU50 |
| mediator complex subunit 1 | MED1 | Q9HD39 |
| Nipped-B homolog (Drosophila) | NIPBL | Q6KCD6 |
| nucleolar and coiled-body phosphoprotein 1 | NOLC1 | Q5VV70 |
| chromosome 16 open reading frame 53 | PA1 | Q9BTK6.1 |
| chemokine (C-C motif) ligand 18 (pulmonary and activation-regulated) | PARC | P55774.1 |
| poly (ADP-ribose) polymerase 1 | PARP1 | Q8IUZ9 |
| phosphatidylinositol 4-kinase, catalytic, alpha pseudogene 2 | PI4KAP2 | A4QPH2.2 |
| prominin 2 | PROM2 | Q8N271 |
| CNGB1 cyclic nucleotide gated channel beta 1 | RCNC2 | Q14028.2 |
| signal-induced proliferation-associated 1 like 3 | SIPA1L3 | Q8IUV1 |
| solute carrier family 25, member 30 | SLC25A30 | Q5SVS4 |
| thrombospondin, type I, domain containing 1 | THSD1 | B2RCF5 |
| tight junction protein 3 (zona occludens 3) | TJP3 | Q96KB4 |
| thyroid stimulating hormone receptor | TSHR | Q9UPH3 |
| ubiquitin-like with PHD and ring finger domains 2 | UHRF2 | Q5VYR1 |
| UV radiation resistance associated gene | UVRAG | Q6P1X0 |
|  |  |  |
| **1μM MeCh** |  |  |
| ADAM metallopeptidase with thrombospondin type 1 motif, 13 | ADAMTS13 | Q6QNA7 |
| similar to Serine-protein kinase ATM (Ataxia telangiectasia mutated) | ATM | Q6P7P1 |
| B1 protein | B1 | X84838.1 |
| chromosome 16 open reading frame 71 | C16orf71 | Q8NCV0 |
| chromosome 17 open reading frame 97 | C17orf97 | Q6PFW9 |
| chromosome 20 open reading frame 26 | C20orf26 | Q9UFV8 |
| chromobox homolog 8 (Pc class homolog, Drosophila) | CBX8 | Q9NR07 |
| coiled-coil domain containing 124 | CCDC124 | Q96CT7 |
| contactin associated protein-like 5 | CNTNAP5 | Q53RX1 |
| DEAD (Asp-Glu-Ala-Asp) box polypeptide 60 | DDX60 | Q6PK35 |
| deleted in liver cancer 1 | DLC1 | Q45XF9 |
| erythrocyte membrane protein band 4.1-like 2 | EPB41L2 | Q59FD8 |
| forkhead-associated (FHA) phosphopeptide binding domain 1 | FHAD1 | Q5JYW1 |
| Ral GTPase activating protein, alpha subunit 1 (catalytic) | GARNL1 | Q9H984 |
| growth differentiation factor 7 | gdf7 | Q75RY1 |
| G protein-coupled receptor 18 | GPR18 | Q9H2L2 |
| heterogeneous nuclear ribonucleoprotein C (C1/C2) | HNRNPC | P22628 |
| heat shock transcription factor 1 | HSF1 | Q3KQR6 |
| KN motif and ankyrin repeat domains 2 | KANK2 | Q9P210 |
| potassium voltage-gated channel, subfamily H (eag-related), member 7 | KCNH7 | Q53QU4 |
| LEM domain containing 2 | LEMD2 | Q5T972 |
| matrin 3 | MATR3 | Q5CZA7 |
| ras homolog gene family, member B | MST081 | Q7Z4F6 |
| mitochondrial translational initiation factor 2 | MTIF2 | Q05DA1 |
| myotubularin related protein 4 | MTMR4 | Q9Y4D5 |
| mucin 20, cell surface associated | MUC20 | Q8NBY6 |
| v-myc myelocytomatosis viral oncogene homolog 1, lung carcinoma derived (avian) | MYCL1 | Q9NUE9 |
| nuclear factor I/B | NFIB | Q96J45 |
| phosphoinositide-3-kinase, regulatory subunit 3 (gamma) | PIK3R3 | Q8N381 |
| peptidase M20 domain containing 1 | PM20D1 | Q96DM4 |
| polymerase (RNA) I polypeptide B, 128kDa | POLR1B | Q9H9Y6 |
| proteasome (prosome, macropain) 26S subunit, non-ATPase, 2 | PSMD2 | Q13200 |
| protein tyrosine phosphatase, receptor type, F | PTPRF | Q7Z3X4 |
| pumilio homolog 2 (Drosophila) | PUM2 | Q9HAN2 |
| REX1, RNA exonuclease 1 homolog (S. cerevisiae)-like 2 (pseudogene) | REXO1L2P | A0PJM3.2 |
| resistance to inhibitors of cholinesterase 3 homolog (C. elegans) | RIC3 | B2RD25 |
| Sec23 homolog A (S. cerevisiae) | SEC23A | Q15436 |
| serpin peptidase inhibitor, clade G (C1 inhibitor), member 1 | SERPING1 | Q16304 |
| serpin peptidase inhibitor, clade I (pancpin), member 2 | SERPINI2 | O75830 |
| sphingomyelin synthase 2 | SGMS2 | Q8NHU3 |
| spinster homolog 2 (Drosophila) | SPNS2 | Q8IVW8 |
| tight junction associated protein 1 (peripheral) | TJAP1 | Q5JTD1 |
| transmembrane protein 200A | TMEM200A | A8K2A1 |
| unc-13 homolog C (C. elegans) | UNC13C | Q8ND48 |
| von Willebrand factor A domain containing 5B2 | VWA5B2 | Q9BVH8 |
| zinc finger CCCH-type containing 4 | ZC3H4 | Q9Y420 |
| zinc finger homeobox 4 | ZFHX4 | Q5U3C1 |
| zinc finger, FYVE domain containing 19 | ZFYVE19 | Q59G85 |
| zinc finger protein 609 | ZNF609 | O15014 |
|  |  |  |
| **10μM MeCh** |  |  |
| ATP-binding cassette, sub-family A (ABC1), member 2 | ABCA2 | Q5SPZ4 |
| ankyrin repeat domain 24 | ANKRD24 | O75268 |
| ATPase, Ca++ transporting, type 2C, member 2 | ATP2C2 | O75185 |
| autogenous vein graft remodeling associated protein 5 | AVGR5 | AM233521.1 |
| Bloom syndrome, RecQ helicase-like | BLM | Q52M96 |
| chromosome 1 open reading frame 83 | C1orf83 | Q96MN5 |
| chromosome 21 open reading frame 70 | C21orf70 | Q9NSI2 |
| complement component 4 binding protein, beta | C4BPB | Q5VVR0 |
| cell division cycle associated 2 | CDCA2 | Q69YH5 |
| CDKN2A interacting protein | CDKN2AIP | Q9NXV6 |
| complement factor H-related 3 | CFHR3 | Q6NSD3 |
| ceruloplasmin (ferroxidase) | CP | Q14063 |
| death-domain associated protein | DAXX | Q5STR5 |
| DEAD (Asp-Glu-Ala-Asp) box polypeptide 3, X-linked | DDX3X | A8K538 |
| defensin, beta 132 | DEFB132 | B2RP72 |
| DNA methyltransferase 1 associated protein 1 | DMAP1 | Q5TG40 |
| dedicator of cytokinesis 4 | DOCK4 | Q8NB45 |
| dual specificity phosphatase 4 | DUSP4 | Q13649 |
| dynein, cytoplasmic 1, light intermediate chain 2 | DYNC1LI2 | Q8TAT3 |
| ELK1, member of ETS oncogene family | ELK1 | Q86SR6 |
| family with sequence similarity 178, member A | FAM178A | Q05BG6 |
| golgi-specific brefeldin A resistant guanine nucleotide exchange factor 1 | GBF1 | Q05BW6 |
| glycophorin A (MNS blood group) | GYPA | Q03867 |
| hyperpolarization activated cyclic nucleotide-gated potassium channel 3 | HCN3 | Q4VX12 |
| KIAA0528 | KIAA0528 | Q17RY7 |
| KIAA1377 | KIAA1377 | Q4G0U6 |
| G-protein coupled receptor KPG_008 | KPG_008 | AB041941.1 |
| luteinizing hormone/choriogonadotropin receptor | LHCGR | Q15996 |
| leucine rich repeat containing 1 | LRRC1 | Q9BTT6 |
| mitogen-activated protein kinase binding protein 1 | MAPKBP1 | Q14CD8 |
| microtubule associated monoxygenase, calponin and LIM domain containing 3 | MICAL3 | Q96DF2 |
| mitochondrial ribosomal protein S12 | MRPS12 | Q53X98 |
| mutS homolog 6 (E. coli) | MSH6 | Q8TCX4 |
| serine/threonine protein kinase MST4 | MST4 | Q9BXC3 |
| myosin IXA | MYO9A | Q9UNJ2 |
| NDC80 homolog, kinetochore complex component (S. cerevisiae) | NDC80 | Q6PJX2 |
| p21 protein (Cdc42/Rac)-activated kinase 2 | PAK2 | Q13154 |
| PDZ domain containing 1 | PDZK1 | Q5T2W1 |
| phosphatidylinositol glycan anchor biosynthesis, class A | PIGA | P37287 |
| pleckstrin homology domain containing, family N member 1 | PLEKHN1 | Q494U1 |
| RAD9 homolog A (S. pombe) | RAD9A | Q99638 |
| recombination activating gene 1 | RAG1 | Q9BYY2 |
| RNA binding protein, autoantigenic (hnRNP-associated with lethal yellow homolog | RALY | Q53GL6 |
| arginine/serine-rich coiled-coil 1 | RSRC1 | Q96QK2 |
| transducin-like enhancer of split 3 (E(sp1) homolog, Drosophila) | TLE3 | B3KUA2 |
| translocated promoter region (to activated MET oncogene) | TPR | P12270 |
| unc-51-like kinase 2 (C. elegans) | ULK2 | Q8IYT8 |
| up-regulated gene 4 isoform 1 | URG4 | AY078404.1 |
| vesicle-associated membrane protein 4 | VAMP4 | Q96J20 |
| wingless-type MMTV integration site family, member 2B | WNT2B | Q5TEH9 |
| zinc finger protein 354A | ZNF354A | O60765 |
|  |  |  |
| **100μM MeCh** |  |  |
| v-abl Abelson murine leukemia viral oncogene homolog 2 (arg, Abelson-related gene) | ABL2 | Q5W0C5 |
| A kinase (PRKA) anchor protein 12 | AKAP12 | Q99970 |
| ankyrin repeat domain 26 | ANKRD26 | Q9NSK9 |
| similar to Acidic leucine-rich nuclear phosphoprotein 32 family member B | ANP32B | P78459 |
| alpha thalassemia/mental retardation syndrome X-linked (RAD54 homolog) | ATRX | Q7Z2J1 |
| breast carcinoma amplified sequence 3 | BCAS3 | Q8NDR8 |
| BCL2/adenovirus E1B 19kDa interacting protein 3 | BNIP3 | Q96GP0 |
| bromodomain containing 2 | BRD2 | Q15310 |
| chromosome 11 open reading frame 84 | C11orf84 | Q9BUA3 |
| chromosome 14 open reading frame 145 | C14orf145 | Q96ML4 |
| chromosome 5 open reading frame 30 | C5orf30 | Q96GV9 |
| CaM kinase-like vesicle-associated | CAMKV | Q8NDU4 |
| coiled-coil domain containing 6 | CCDC6 | Q15250 |
| cadherin-like 23 | CDH23 | Q9H4K9 |
| complement factor H | CFH | Q9NU86 |
| catenin (cadherin-associated protein), alpha 2 | CTNNA2 | B7Z898 |
| dCMP deaminase | DCTD | Q9BVD8 |
| death inducer-obliterator 1 | DIDO1 | Q4VXS2 |
| Hypothetical protein | DKFZp686O16217 | BX640710.1 |
| DMRT-like family A2 | DMRTA2 | Q5TFQ3 |
| phosphoribosylglycinamide formyltransferase | GART | Q3B7A7 |
| GDNF family receptor alpha 4 | GFRA4 | Q5JT74 |
| interleukin enhancer binding factor 3, 90kDa | ILF3 | Q12906 |
| jun oncogene | JUN | Q6FHM7 |
| kelch repeat and BTB (POZ) domain containing 11 | KBTBD11 | O94819 |
| potassium channel tetramerisation domain containing 1 | KCTD1 | Q719H9 |
| KIAA1324 | KIAA1324 | Q6UXG2 |
| KIAA1704 | KIAA1704 | Q05D87 |
| La ribonucleoprotein domain family, member 1 | LARP1 | Q9UFD7 |
| La ribonucleoprotein domain family, member 7 | LARP7 | Q9Y3Z8 |
| melanoma antigen family B, 16 | MAGEB16 | A2A368 |
| methyl-CpG binding domain protein 4 | MBD4 | Q7Z4T3 |
| hypothetical MGC50722 | MGC50722 | Q8N5P7 |
| metastasis associated 1 family, member 2 | MTA2 | Q9UQB5 |
| NIMA (never in mitosis gene a)-related kinase 4 | NEK4 | Q6P576 |
| nestin | NES | Q2YDX4 |
| NK6 homeobox 3 | NKX6-3 | Q96LR0 |
| nucleoporin 50kDa | NUP50 | Q9UKX7 |
| processing of precursor 1, ribonuclease P/MRP subunit (S. cerevisiae) | POP1 | A8K5W9 |
| pleckstrin and Sec7 domain containing 3 | PSD3 | Q6B003 |
| PC4 and SFRS1 interacting protein 1 | PSIP1 | Q6P391 |
| polypyrimidine tract binding protein 1 | PTBP1 | Q9BUQ0 |
| RNA binding motif protein 15 | RBM15 | Q9BRA5 |
| RNA binding motif protein 14; RNA binding motif protein 4 | RBM4 | Q53GV1 |
| RD RNA binding protein | RDBP | Q9BQJ6 |
| RER1 retention in endoplasmic reticulum 1 homolog (S. cerevisiae) | RER1 | O15258 |
| regulatory factor X, 7 | RFX7 | Q8N3J0 |
| RAP1 interacting factor homolog (yeast) | RIF1 | A6NC27 |
| RWD domain containing 3 | RWDD3 | A8K9F0 |
| squamous cell carcinoma antigen recognized by T cells | SART1 | O43290 |
| SEC23 interacting protein | SEC23IP | B3KM47 |
| solute carrier family 4, sodium bicarbonate cotransporter, member 4 | SLC4A4 | Q9Y6R1 |
| saitohin | STH | A1L3X7 |
| suppressor of defective silencing 3 homolog (S. cerevisiae) | SUDS3 | Q4KMQ5 |
| transcription factor 12 | TCF12 | Q9NQY9 |
| thymocyte nuclear protein 1 | THYN1 | Q9P016 |
| tripartite motif-containing 28 | TRIM28 | Q7Z632 |
| ubiquitin associated protein 2-like | UBAP2L | Q9UGL5 |
| ubiquitin fusion degradation 1 like (yeast) | UFD1L | A8MW31 |
| ubiquitin specific peptidase 31 | USP31 | Q9ULL7 |
| vacuolar protein sorting 33 homolog B (yeast) | VPS33B | Q96K14 |
| V-set and immunoglobulin domain containing 2 | VSIG2 | O95791 |
| Wilms tumor 1 associated protein | WTAP | Q9BZS4 |
