# Supplementary material for: VENNTURE–A Novel Venn Diagram Investigational Tool for Multiple Pharmacological Dataset Analysis
Source: PLoS One. 2012 May 14;7(5):e36911. doi: 10.1371/journal.pone.0036911 (PMC3351456; doi:10.1371/journal.pone.0036911)
Supplement: Table S16 — Dose-unique MeCh-stimulated protein phosphorylation in CMP-state SH-SY5Y cells. The proteins uniquely phosphorylated at the specified MeCh dose only in CMP-state SH-SY5Y cells are indicated by their protein symbol as well as Uniprot accession number. (DOC) [file pone.0036911.s017.doc]

**Table S16.** Dose-unique MeCh-stimulated protein phosphorylation in CMP-state SH-SY5Y. The proteins uniquely phosphorylated at the specified MeCh dose only in CMP-state SH-SY5Y cells are indicated by their protein symbol as well as Uniprot accession number.

| **Protein Identification** | **Symbol** | **Accession** |
| --- | --- | --- |
| **10nM MeCh** |  |  |
| Rho GTPase activating protein 21 | ARHGAP21 | Q0VF98 |
| cancer antigen 1 | CAGE1 | Q8TC20 |
| coiled-coil domain containing 88A | CCDC88A | Q9ULK8 |
| DnaJ (Hsp40) homolog, subfamily C, member 16 | DNAJC16 | Q68D57 |
| dipeptidyl-peptidase 8 | DPP8 | Q9NXF4 |
| E2F transcription factor 8 | E2F8 | Q9H5M0 |
| eukaryotic translation initiation factor 2 alpha kinase 4 | EIF2AK4 | Q69YL7 |
| family with sequence similarity 179, member B | FAM179B | Q9Y4F4 |
| ferredoxin reductase | FDXR | Q4PJI0 |
| gap junction protein, alpha 10, 62kDa | GJA10 | Q969M2 |
| G protein-coupled receptor 18 | GPR18 | Q9H2L2 |
| G protein-coupled receptor 52 | GPR52 | Q4VBL6 |
| human immunodeficiency virus type I enhancer binding protein 3 | HIVEP3 | Q99302 |
| JMJD2B protein | JMJD2B | BC144292.1 |
| Leber congenital amaurosis 5 | LCA5 | Q9BWX7 |
| melanoma antigen family D, 2 | MAGED2 | O76058 |
| multiple PDZ domain protein | MPDZ | Q4LE30 |
| oxysterol-binding protein-related protein 8 isoform a | MST120 | AF392452.1 |
| NHS-like 1 | NHSL1 | Q9P2J0 |
| nuclear receptor binding protein 1 | NRBP1 | Q96SU3 |
| olfactory receptor, family 4, subfamily D, member 2 | OR4D2 | P58180 |
| Novel protein | RP13-347D8.5-001 | AL772284.5 |
| SUMO1/sentrin specific peptidase 7 | SENP7 | Q9C0F6 |
| SPARC related modular calcium binding 1 | SMOC1 | B2R7P5 |
| syntrophin, gamma 1 | SNTG1 | Q2M3Q0 |
| suppression of tumorigenicity 5 | ST5 | P78524 |
| synapse defective 1, Rho GTPase, homolog 2 (C. elegans) | SYDE2 | Q5VT97 |
| TAF3 RNA polymerase II, TATA box binding protein (TBP)-associated factor, 140kDa | TAF3 | Q6P6B5 |
| transmembrane protein 44 | TMEM44 | B7ZLZ5 |
| tau tubulin kinase 2 | TTBK2 | Q8IWY7 |
| ventral anterior homeobox 2 | VAX2 | Q9UIW0 |
| WD repeat domain 17 | WDR17 | Q0QD35 |
| exportin 5 | XPO5 | Q5JTE7 |
| zinc finger protein, multitype 2 | ZFPM2 | Q32MA6 |
| zinc finger protein 534; zinc finger protein 528 | ZNF534 | Q3MIS6 |
|  |  |  |
| **100nM MeCh** |  |  |
| AF4/FMR2 family, member 2 | AFF2 | A2RTY4 |
| AHNAK nucleoprotein | AHNAK | Q96EC4 |
| ankyrin repeat, family A (RFXANK-like), 2 | ANKRA2 | Q9H9E1 |
| androgen receptor | AR | P10275 |
| activating signal cointegrator 1 complex subunit 3 | ASCC3 | Q9H1I9 |
| ataxia telangiectasia and Rad3 related; similar to ataxia telangiectasia and Rad3 related protein | ATR | Q13535 |
| bromodomain adjacent to zinc finger domain, 2A | BAZ2A | Q68DI8 |
| BMS1 homolog, ribosome assembly protein (yeast) pseudogene | BMS1 | Q5QPT5 |
| BRCA1 interacting protein C-terminal helicase 1 | BRIP1 | Q9BX63 |
| similar to protein phosphatase 1, regulatory subunit 2; protein phosphatase 1, regulatory (inhibitor) | C13orf18 | Q6ZU68 |
| chromosome 14 open reading frame 38 | C14orf38 | P0C221 |
| carbamoyl-phosphate synthetase 2, aspartate transcarbamylase, and dihydroorotase | CAD | P27708 |
| chemokine (C-C motif) ligand 14; chemokine (C-C motif) ligand 15 | CCL14 | B2RU34 |
| cingulin | CGN | Q9P2M7 |
| deoxynucleotidyltransferase, terminal, interacting protein 2 | DNTTIP2 | Q5QJE6 |
| double C2-like domains, alpha | DOC2A | Q7Z5G0 |
| dishevelled, dsh homolog 2 (Drosophila) | DVL2 | Q53XM0 |
| fibronectin type III domain containing 1 | FNDC1 | Q4ZHG4 |
| fizzy/cell division cycle 20 related 1 (Drosophila) | FZR1 | Q96NW8 |
| GATA binding protein 1 (globin transcription factor 1) | GATA1 | P15976 |
| GATA binding protein 3 | GATA3 | P23771 |
| GINS complex subunit 3 (Psf3 homolog) | GINS3 | Q9H870 |
| glycosylphosphatidylinositol specific phospholipase D1 | GPLD1 | Q9H167 |
| inositol(myo)-1(or 4)-monophosphatase 1 | IMPA1 | B4DLN3 |
| integrin, alpha 1 | ITGA1 | P56199 |
| inositol 1,4,5-triphosphate receptor, type 2 | ITPR2 | Q14571 |
| kinesin light chain 1 | KLC1 | Q7RTQ4 |
| kinesin light chain 3 | KLC3 | Q8WWJ9 |
| low density lipoprotein-related protein 1 (alpha-2-macroglobulin receptor) | LRP1 | Q2PP12 |
| mitogen-activated protein kinase kinase kinase 2 | MAP3K2 | Q53S75 |
| mannan-binding lectin serine peptidase 1 (C4/C2 activating component of Ra-reactive factor) | MASP1 | Q6MZL2 |
| mediator complex subunit 11 | MED11 | Q6NS89 |
| MICAL-like 1 | MICALL1 | Q7RTP5 |
| MICAL-like 2 | MICALL2 | Q7RTP4 |
| myomesin 1, 185kDa | MYOM1 | Q9NZL8 |
| NAD kinase | NADK | Q9H931 |
| nuclear receptor subfamily 1, group H, member 4 | NR1H4 | B7Z412 |
| nuclear RNA export factor 1 | NXF1 | Q9UQL2 |
| partner and localizer of BRCA2 | PALB2 | Q8N7Y6 |
| paralemmin | PALM | Q9UQS3 |
| phospholipase A2, group VI (cytosolic, calcium-independent) | PLA2G6 | Q9UIT0 |
| pleckstrin homology domain containing, family A member 5 | PLEKHA5 | Q86ST7 |
| pleckstrin homology domain containing, family H (with MyTH4 domain) member 2 | PLEKHH2 | Q8N3Q3 |
| phosphoribosyl pyrophosphate synthetase-associated protein 1 | PRPSAP1 | Q14558 |
| proteasome (prosome, macropain) 26S subunit, ATPase, 1 | PSMC1 | Q6IAW0 |
| polypyrimidine tract binding protein 1 | PTBP1 | Q9BUQ0 |
| regulator of G-protein signaling 12 | RGS12 | O75338 |
| SEC24 family, member D (S. cerevisiae) | SEC24D | Q4W5D3 |
| septin 5 | SEPT5 | Q96MY5 |
| solute carrier family 44, member 1 | SLC44A1 | Q96KU3 |
| transforming, acidic coiled-coil containing protein 2 | TACC2 | Q9BVQ1 |
| transmembrane protein 45A | TMEM45A | Q9NWC5 |
| tumor protein p53 binding protein, 2 | TP53BP2 | Q13625 |
| vacuolar protein sorting 13 homolog C (S. cerevisiae) | VPS13C | Q9NXN8 |
| von Willebrand factor A domain containing 3B | VWA3B | Q86T73 |
| zinc finger, MYND-type containing 8 | ZMYND8 | Q2HXV9 |
| zinc finger protein 236 | ZNF236 | Q9UL37 |
| zinc finger protein 391 | ZNF391 | B4DH77 |
|  |  |  |
| **1μM MeCh** |  |  |
| POTE ankyrin domain family, member E | A26C1A | Q562Q2 |
| ADAM metallopeptidase domain 22 | ADAM22 | Q75MS7 |
| activity-dependent neuroprotector homeobox | ADNP | Q5BKU2 |
| ARD1A protein | ARD1A | BC063377.1 |
| Rho GTPase activating protein 17 | ARHGAP17 | Q6ZUS4 |
| Rho guanine nucleotide exchange factor (GEF) 12 | ARHGEF12 | Q9P149 |
| AT rich interactive domain 1A (SWI-like) | ARID1A | Q96T89 |
| aryl hydrocarbon receptor nuclear translocator | ARNT | Q59ED4 |
| ArfGAP with SH3 domain, ankyrin repeat and PH domain 1 | ASAP1 | Q9ULH1 |
| bone morphogenetic protein 2 | BMP2 | C8C060 |
| chromosome 12 open reading frame 49 | C12orf49 | Q53GE8 |
| chromosome 14 open reading frame 145 | C14orf145 | Q96ML4 |
| chromosome 17 open reading frame 82 | C17orf82 | Q86X59 |
| chromosome 20 open reading frame 117 | C20orf117 | Q14DB2 |
| chromosome 22 open reading frame 30 | C22orf30 | Q5THK4 |
| chromosome 2 open reading frame 49 | C2orf49 | B3KXN3 |
| chromosome 3 open reading frame 30 | C3orf30 | Q96M34 |
| KIAA0408; chromosome 6 open reading frame 174 | C6orf174 | A5PLQ8 |
| chromosome 7 open reading frame 27 | C7orf27 | Q8IW85 |
| chromosome 7 open reading frame 50 | C7orf50 | Q9BRJ6 |
| chromosome 7 open reading frame 54 | C7orf54 | Q9HBX3 |
| chromosome 9 open reading frame 84 | C9orf84 | Q96M73 |
| cache domain containing 1 | CACHD1 | Q9H7W4 |
| caspase recruitment domain family, member 10 | CARD10 | Q9UGR6 |
| cyclin Y | CCNY | Q8TEX3 |
| CD2 (cytoplasmic tail) binding protein 2 | CD2BP2 | Q9ULP2 |
| chromatin assembly factor 1, subunit A (p150) | CHAF1A | Q9UJY8 |
| chromodomain helicase DNA binding protein 1-like | CHD1L | Q9BVJ1 |
| chromodomain helicase DNA binding protein 9 | CHD9 | Q461N2 |
| chromogranin A (parathyroid secretory protein 1) | CHGA | Q53FA8 |
| cytokine inducible SH2-containing protein | CISH | Q71V34 |
| claudin 15 | CLDN15 | Q96FX9 |
| coilin | COIL | B2R931 |
| cleavage and polyadenylation specific factor 7, 59kDa | CPSF7 | Q7Z3H9 |
| dapper, antagonist of beta-catenin, homolog 2 (Xenopus laevis) | DACT2 | Q569G0 |
| dachsous 1 (Drosophila) | DCHS1 | Q96JQ0 |
| dehydrogenase/reductase (SDR family) member 3 | DHRS3 | Q0QD44 |
| dispatched homolog 1 (Drosophila) | DISP1 | Q8N7C2 |
| hypothetical protein | DKFZp779J2370 | BX640928.1 |
| DnaJ (Hsp40) homolog, subfamily C, member 21 | DNAJC21 | Q86VC6 |
| histidyl-tRNA synthetase 2, mitochondrial (putative); D-tyrosyl-tRNA deacylase 1 homolog (S. cerevisiae) | DTD1 | Q9H464 |
| dual specificity phosphatase 15; chromosome 20 open reading frame 57 | DUSP15 | Q9BX24 |
| enhancer of mRNA decapping 3 homolog (S. cerevisiae) | EDC3 | Q9H797 |
| erythrocyte membrane protein band 4.1 (elliptocytosis 1, RH-linked) | EPB41 | Q8IXV9 |
| erbb2 interacting protein | ERBB2IP | Q86W38 |
| family with sequence similarity 186, member B | FAM186B | Q9H0L3 |
| family with sequence similarity 48, member A | FAM48A | Q8WYR6 |
| family with sequence similarity 62, member C | FAM62C | BC037292.1 |
| forkhead box N3 | FOXN3 | Q9P1I8 |
| forty-two-three domain containing 1 | FYTTD1 | C9J7P6 |
| gamma-aminobutyric acid (GABA) A receptor, alpha 4 | GABRA4 | P48169 |
| galanin receptor 1 | GALR1 | P47211 |
| granzyme K (granzyme 3; tryptase II) | GZMK | B2R563 |
| hemoglobin, alpha 2; hemoglobin, alpha 1 | HBA1 | Q96KF1 |
| heat shock 105kDa/110kDa protein 1 | HSPH1 | O95739 |
| jumonji domain containing 1C | JMJD1C | Q8N3U0 |
| potassium voltage-gated channel, KQT-like subfamily, member 5 | KCNQ5 | Q9NR82 |
| KIAA1429 | KIAA1429 | Q9BTH4 |
| kinesin family member 25 | KIF25 | Q9UIL4 |
| Kruppel-like factor 3 (basic) | KLF3 | Q6PIR1 |
| La ribonucleoprotein domain family, member 5 | LARP5 | Q92615 |
| La ribonucleoprotein domain family, member 7 | LARP7 | Q9Y3Z8 |
| tetratricopeptide repeat and ankyrin repeat containing 1 | LBA1 | O15050.4 |
| LIM domain binding 1 | LDB1 | Q9UGM4 |
| lipoxygenase homology domains 1 | LOXHD1 | Q8IVV2 |
| hypothetical LOC642946 | LQK1 | Q8TAF5 |
| leucine rich repeat containing 37A | LRRC37A | A6NMS7 |
| lymphocyte antigen 6 complex, locus H | LY6H | Q6IAX0 |
| MAP/microtubule affinity-regulating kinase 4 | MARK4 | Q96JG7 |
| mitochondrial fission factor | MFF | Q9BVZ1 |
| matrix metallopeptidase 10 (stromelysin 2) | MMP10 | B2R9X9 |
| matrix metallopeptidase 14 (membrane-inserted) | MMP14 | Q6GSF3 |
| myeloid cell nuclear differentiation antigen | MNDA | Q5VUU6 |
| mitochondrial ribosomal protein S2 | MRPS2 | Q9BSQ4 |
| MYB binding protein (P160) 1a | MYBBP1A | Q9UF99 |
| N-terminal EF-hand calcium binding protein 2 | NECAB2 | O75547 |
| neurofilament, heavy polypeptide | NEFH | Q9UJS7 |
| NLR family, pyrin domain containing 9 | NLRP9 | B2RN12 |
| neuropeptide S | NPS | P0C0P6 |
| platelet derived growth factor C | PDGFC | B9EGR8 |
| phosphoglycerate kinase 1 | PGK1 | Q5J7W1 |
| polyhomeotic homolog 1B (Drosophila); polyhomeotic homolog 1 (Drosophila) | PHC1 | Q9BU63 |
| phosphatidylinositol glycan anchor biosynthesis, class A | PIGA | P37287 |
| PNMA-like 2 | PNMAL2 | Q08E79 |
| Unknown protein | pp14450 | AF318341.1 |
| protein phosphatase 1, regulatory (inhibitor) subunit 16A | PPP1R16A | Q96I34 |
| PRP38 pre-mRNA processing factor 38 (yeast) domain containing B | PRPF38B | Q9NW40 |
| proline/serine-rich coiled-coil 1 | PSRC1 | Q9BV77 |
| protein tyrosine phosphatase, non-receptor type 4 (megakaryocyte) | PTPN4 | Q580X3 |
| RAD18 homolog (S. cerevisiae) | RAD18 | Q53H10 |
| RAS protein activator like 2 | RASAL2 | Q6P4F9 |
| RNA binding motif protein 26 | RBM26 | Q2NKM2 |
| regulatory factor X, 5 (influences HLA class II expression) | RFX5 | Q9UG77 |
| RIO kinase 3 (yeast) | RIOK3 | B0YJ89 |
| ring finger protein 40 | RNF40 | Q6AHZ6 |
| ribosomal RNA processing 12 homolog (S. cerevisiae) | RRP12 | Q9Y4C7 |
| sema domain, transmembrane domain (TM), and cytoplasmic domain, (semaphorin) 6D | SEMA6D | Q9P249 |
| splicing factor, arginine/serine-rich 17A | SFRS17A | Q8N6U9 |
| sphingosine-1-phosphate phosphatase 1 | SGPP1 | B2RAH0 |
| shisa homolog 2 (Xenopus laevis) | SHISA2 | Q6UWI4 |
| shroom family member 4 | SHROOM4 | Q9ULL8 |
| signal-induced proliferation-associated 1 like 3 | SIPA1L3 | Q8IUV1 |
| solute carrier family 1 (glutamate/neutral amino acid transporter), member 4 | SLC1A4 | P43007 |
| solute carrier family 25 (mitochondrial carrier; ornithine transporter) member 2 | SLC25A2 | Q9BXI2 |
| solute carrier family 9 (sodium/hydrogen exchanger), member 5 | SLC9A5 | Q9Y626 |
| SWI/SNF related, matrix associated, actin dependent regulator of chromatin, subfamily c, member 1 | SMARCC1 | Q17RS0 |
| sperm antigen with calponin homology and coiled-coil domains 1-like | SPECC1L | Q59GT7 |
| testis expressed 14 | TEX14 | Q8ND97 |
| tight junction associated protein 1 (peripheral) | TJAP1 | Q5JTD1 |
| transmembrane protein 95 | TMEM95 | Q3KNT9 |
| torsin family 1, member B (torsin B) | TOR1B | Q9BR69 |
| testis specific protein, Y-linked 3; testis specific protein, Y-linked 2; testis specific protein, Y-linked 1; testis specific protein, Y-linked pseudogene 7 | TSPY2 | Q0VAD3 |
| ubiquitin specific peptidase 6 (Tre-2 oncogene) | USP6 | Q9UDD3 |
| vomeronasal 1 receptor 5 | VN1R5 | A0AVG1 |
| vacuolar protein sorting 11 homolog (S. cerevisiae) | VPS11 | Q8WY89 |
| WD repeat domain 44 | WDR44 | Q7Z3P6 |
| 5'-3' exoribonuclease 1 | XRN1 | Q8IZH2 |
| zinc finger, DHHC-type containing 5 | ZDHHC5 | Q9H923 |
| zinc finger RNA binding protein | ZFR | Q9Y687 |
| zinc finger protein 474 | ZNF474 | Q96M07 |
| zinc finger protein 663 | ZNF663 | Q8NDT4 |
|  |  |  |
| **10μM MeCh** |  |  |
| ARP8 actin-related protein 8 homolog (yeast) | ACTR8 | Q8N566 |
| arachidonate 12-lipoxygenase, 12R type | ALOX12B | O75342 |
| ankyrin repeat domain 17 | ANKRD17 | Q8NDR5 |
| ATPase, class V, type 10A | ATP10A | Q969I4 |
| Bardet-Biedl syndrome 7 | BBS7 | Q9NVI4 |
| BRF1 homolog, subunit of RNA polymerase III transcription initiation factor IIIB (S. cerevisiae) | BRF1 | Q6IQ02 |
| BTB (POZ) domain containing 8 | BTBD8 | Q6V9S5 |
| chromosome 12 open reading frame 42 | C12orf42 | Q96LP6 |
| chromosome 14 open reading frame 48 | C14orf48 | Q8NCU1 |
| chromosome 1 open reading frame 92 | C1orf92 | Q8N4P6 |
| chromosome 4 open reading frame 47 | C4orf47 | A7E2U8 |
| chromosome 9 open reading frame 82 | C9orf82 | Q6IPE6 |
| centrosomal protein 120kDa | CCDC100 | Q8N960.2 |
| coiled-coil domain containing 132 | CCDC132 | Q75N11 |
| coiled-coil domain containing 142 | CCDC142 | B7ZKV5 |
| CD22 molecule | CD22 | Q9UQB1 |
| cat eye syndrome chromosome region, candidate 2 | CECR2 | A8MS90 |
| centromere protein K | CENPK | Q9H4L0 |
| cofilin 1 (non-muscle) | CFL1 | B3KUQ1 |
| carbohydrate (chondroitin 4) sulfotransferase 13 | CHST13 | Q3SYA5 |
| capicua homolog (Drosophila) | CIC | Q9Y6T1 |
| C-type lectin domain family 7, member A | CLEC7A | Q96D32 |
| contactin associated protein-like 5 | CNTNAP5 | Q53RX1 |
| crumbs homolog 1 (Drosophila) | CRB1 | Q59H36 |
| centrosome and spindle pole associated protein 1 | CSPP1 | A6ND63 |
| cytochrome P450, family 19, subfamily A, polypeptide 1 | CYP19A1 | Q8TCA4 |
| DBF4 homolog (S. cerevisiae) | DBF4 | Q75MS6 |
| DENN/MADD domain containing 1B | DENND1B | Q5T3B9 |
| diacylglycerol kinase, zeta 104kDa | DGKZ | Q6ZWA5 |
| death inducer-obliterator 1 | DIDO1 | Q4VXS2 |
| Hypothetical protein | DKFZp686K09128 | CR749605.1 |
| dickkopf homolog 1 (Xenopus laevis) | DKK1 | B2RC19 |
| discs, large (Drosophila) homolog-associated protein 2 | DLGAP2 | A1QCF8 |
| dynein, axonemal, heavy chain 1 | DNAH1 | Q8TEJ4 |
| euchromatic histone-lysine N-methyltransferase 1 | EHMT1 | Q86X08 |
| elastin microfibril interfacer 2 | EMILIN2 | Q8N5L1 |
| family with sequence similarity 154, member A | FAM154A | Q8IYX7 |
| fibrillin 1 | FBN1 | Q15972 |
| Fc receptor, IgA, IgM, high affinity | FCAMR | Q8WWV6 |
| Fc fragment of IgA, receptor for | FCAR | Q9UEK0 |
| FCH domain only 1 | FCHO1 | Q8IW22 |
| GTPase activating protein (SH3 domain) binding protein 2 | G3BP2 | O60606 |
| growth arrest-specific 2 like 3 | GAS2L3 | Q86XJ1 |
| gap junction protein, alpha 1, 43kDa | GJA1 | Q6FHU1 |
| galactosidase, alpha | GLA | Q53Y83 |
| YY1 associated protein 1; gon-4-like (C. elegans) | GON4L | Q1ED43 |
| G protein-coupled receptor 126 | GPR126 | Q6MZU7 |
| glutamate receptor, ionotropic, N-methyl D-aspartate 2C | GRIN2C | Q8IW23 |
| KIAA0182 protein | GSE1 | Q59GZ0 |
| H2A histone family, member B1 | H2AFB1 | Q96PR7 |
| histidyl-tRNA synthetase 2, mitochondrial (putative) | HARS2 | Q9H464 |
| hCG_2015407 | hCG_2015407 | CH471054.1 |
| hCG_2026193 | hCG_2026193 | CH471083.1 |
| hCG_2044975 | hCG_2044975 | CH471066.2 |
| hypocretin (orexin) receptor 2 | HCRTR2 | O43614 |
| HEG homolog 1 (zebrafish) | HEG1 | Q8NC40 |
| major histocompatibility complex, class I, C; major histocompatibility complex, class I, B | HLA-C | Q6V4Z4 |
| heterogeneous nuclear ribonucleoprotein C (C1/C2) | HNRNPC | P22628 |
| IGF-like family member 2 | IGFL2 | Q6B9Z3 |
| insulinoma-associated 2 | INSM2 | Q96Q84 |
| jun oncogene | JUN | Q6FHM7 |
| Ku86 autoantigen related protein 1 | KARP-1 | AAC52087.1 |
| potassium voltage-gated channel, subfamily H (eag-related), member 7 | KCNH7 | Q53QU4 |
| potassium channel tetramerisation domain containing 1 | KCTD1 | Q719H9 |
| SECIS binding protein 2-like | KIAA0256 | Q93073.3 |
| KIAA1539 | KIAA1539 | Q9P1Y9 |
| kinesin family member 9 | KIF9 | Q9H8A4 |
| kelch repeat and BTB (POZ) domain containing 12 | KLHDC6 | Q7Z665 |
| keratin 26 | KRT26 | A2RUL2 |
| lactation elevated 1 | LACE1 | Q8N6A3 |
| LIM homeobox 2 | LHX2 | Q52M57 |
| LSM11, U7 small nuclear RNA associated | LSM11 | Q8N975 |
| MAP7 domain containing 1 | MAP7D1 | Q7L8J5 |
| nitrogen permease regulator-like 3 (S. cerevisiae) | MARE | P78384 |
| mutS homolog 5 (E. coli) | MSH5 | Q59EC5 |
| mucin 16, cell surface associated | MUC16 | B3KY81 |
| matrix-remodelling associated 7 | MXRA7 | Q0P5W3 |
| MYC binding protein 2 | MYCBP2 | Q6PIB6 |
| asparaginyl-tRNA synthetase | NARS | O43776 |
| sodium channel, voltage-gated, type V, alpha subunit | Nav1.5 | Q14524.2 |
| non-SMC condensin II complex, subunit D3 | NCAPD3 | A6NFS2 |
| N-glycanase 1 | NGLY1 | Q59FB1 |
| oligodendrocyte transcription factor 3 | OLIG3 | Q7RTU3 |
| olfactory receptor, family 5, subfamily AR, member 1 | OR5AR1 | Q8NGP9 |
| origin recognition complex, subunit 6 like (yeast) | ORC6L | Q9Y5N6 |
| 3-oxoacid CoA transferase 2 | OXCT2 | Q6INA3 |
| PCTAIRE protein kinase 1 | PCTK1 | Q00536 |
| PEX26T35insC protein | PEX26T35insC | AB103104.1 |
| phosphoinositide-3-kinase, class 2, alpha polypeptide | PIK3C2A | O00443 |
| piwi-like 3 (Drosophila) | PIWIL3 | Q7Z3Z3 |
| pleckstrin homology domain containing, family A (phosphoinositide binding specific) member 3 | PLEKHA3 | Q86TQ1 |
| Unknown protein | pp10472 | AF318321.1 |
| protein kinase, interferon-inducible double stranded RNA dependent activator | PRKRA | A8K3I6 |
| protein interacting with cyclin A1 | PROCA1 | Q8NCQ7 |
| patched homolog 2 (Drosophila) | PTCH2 | Q5QP87 |
| hypothetical LOC728026; prothymosin, alpha | PTMAP4 | Q15204 |
| protein tyrosine phosphatase, non-receptor type 13 (APO-1/CD95 (Fas)-associated phosphatase) | PTPN13 | Q4W5F5 |
| RAB11 family interacting protein 5 (class I) | RAB11FIP5 | Q9UFM0 |
| RAN binding protein 10 | RANBP10 | A4FTY2 |
| RNA binding motif protein 14; RNA binding motif protein 4 | RBM4 | Q53GV1 |
| REX1, RNA exonuclease 1 homolog (S. cerevisiae)-like 2 (pseudogene) | REXO1L2P | A0PJM3.2 |
| Rho-guanine nucleotide exchange factor | RGNEF | Q8N1W1 |
| regulator of G-protein signaling 7 | RGS7 | Q9UNU7 |
| ring finger protein 34 | RNF34 | Q9H6W8 |
| ribosomal protein L23a pseudogene 63 | RPL23A | P29316 |
| RUN and FYVE domain containing 4 | RUFY4 | Q6ZR96 |
| sphingosine-1-phosphate receptor 3 | S1PR3 | Q7Z5I2 |
| sciellin | SCEL | B7Z797 |
| signal peptide, CUB domain, EGF-like 2 | SCUBE2 | Q6ZWI1 |
| SET binding protein 1 | SETBP1 | Q9Y6X0 |
| splicing factor, arginine/serine-rich 2B | SFRS2B | Q9BRL6 |
| NKF3 kinase family member | SGK269 | Q9H792 |
| sphingomyelin synthase 2 | SGMS2 | Q8NHU3 |
| solute carrier family 31 (copper transporters), member 2 | SLC31A2 | Q53X94 |
| solute carrier family 35, member F5 | SLC35F5 | Q9H7D8 |
| serum response factor binding protein 1 | SRFBP1 | Q5QFI2 |
| syntaxin 18 | STX18 | Q9P2W9 |
| suppressor of Ty 7 (S. cerevisiae)-like | SUPT7L | Q6IB21 |
| transcription factor 3 (E2A immunoglobulin enhancer binding factors E12/E47) | TCF3 | Q14208 |
| tudor domain containing 5 | TDRD5 | Q8NAT2 |
| tet oncogene 1 | TET1 | Q9C0I7 |
| transforming growth factor, beta receptor associated protein 1 | TGFBRAP1 | Q9H6G8 |
| transmembrane protein 86A | TMEM86A | Q96AJ0 |
| torsin A interacting protein 1 | TOR1AIP1 | Q9Y3X5 |
| translocated promoter region (to activated MET oncogene) | TPR | P12270 |
| TRAF interacting protein | TRAIP | Q9BWF2 |
| tripartite motif-containing 29 | TRIM29 | Q9BZY7 |
| trichorhinophalangeal syndrome I | TRPS1 | Q9NWE1 |
| thioredoxin interacting protein | TXNIP | Q9BXG9 |
| ubiquitin-like with PHD and ring finger domains 2 | UHRF2 | Q5VYR1 |
| unc-51-like kinase 1 (C. elegans) | ULK1 | O75385 |
| vang-like 2 (van gogh, Drosophila) | VANGL2 | Q9ULK5 |
| vacuolar protein sorting 26 homolog B (S. pombe) | VPS26B | Q96A55 |
| WD repeat domain 77 | WDR77 | Q6JZZ5 |
| 5'-3' exoribonuclease 2 | XRN2 | Q9H0D6 |
| YSK4 Sps1/Ste20-related kinase homolog (S. cerevisiae) | YSK4 | Q8N4E9 |
| zinc finger, C3HC-type containing 1 | ZC3HC1 | Q8N330 |
| zinc finger and AT hook domain containing | ZFAT | Q75PJ7 |
| zinc finger, C3H1-type containing | ZFC3H1 | Q6ZV36 |
| zinc finger and BTB domain containing 49 | ZNF509 | Q32MK9 |
| zinc finger protein 608 | ZNF608 | Q9Y5A1 |
| zinc finger protein 609 | ZNF609 | O15014 |
| zinc finger protein 644 | ZNF644 | Q9ULJ9 |
| zinc finger protein 655 | ZNF655 | A6NGD3 |
| zinc finger protein 73 | ZNF73 | O43830.1 |
| zinc finger protein 841 | ZNF841 | Q6ZN82 |
|  |  |  |
| **100μM MeCh** |  |  |
| ADAM metallopeptidase domain 19 (meltrin beta) | ADAM19 | B3KRF5 |
| ADAM metallopeptidase with thrombospondin type 1 motif, 2 | ADAMTS2 | O95450 |
| afamin | AFM | Q4W5C5 |
| A kinase (PRKA) anchor protein 2; paralemmin 2; PALM2-AKAP2 readthrough transcript | AKAP2 | Q9Y2D5 |
| alkaline phosphatase, placental-like 2 | ALPPL2 | A8KAF2 |
| ankyrin repeat domain 30A | ANKRD30A | Q5W025 |
| ankyrin repeat and sterile alpha motif domain containing 1A | ANKS1A | Q5SYR2 |
| apolipoprotein O-like | APOOL | Q68DW4 |
| ADP-ribosylation factor interacting protein 1 | ARFIP1 | B4E273 |
| AT rich interactive domain 1A (SWI-like) | ARID1A | Q96T89 |
| ash1 (absent, small, or homeotic)-like (Drosophila) | ASH1L | Q9P2C7 |
| ATPase family, AAA domain containing 2B | ATAD2B | Q8IWJ3 |
| ATPase, class VI, type 11C | ATP11C | Q8WX24 |
| ataxin 7 | ATXN7 | Q9UPD8 |
| B-cell CLL/lymphoma 9 | BCL9 | O00512 |
| BMP2 inducible kinase | BMP2K | Q8N2G7 |
| chromosome 12 open reading frame 24 | C12orf24 | A6NH30 |
| chromosome 12 open reading frame 43 | C12orf43 | Q9H9Z7 |
| chromosome 14 open reading frame 43 | C14orf43 | Q6PK59 |
| chromosome 15 open reading frame 59 | C15orf59 | Q2T9L4 |
| chromosome 1 open reading frame 64 | C1orf64 | B3KXI9 |
| hypothetical LOC642975; chromosome 20 open reading frame 30 | C20orf30 | Q96A57 |
| chromosome 5 open reading frame 41 | C5orf41 | Q8IZG1 |
| chromosome 6 open reading frame 89 | C6orf89 | Q6UWU4 |
| chromosome 6 open reading frame 97 | C6orf97 | Q9H5M3 |
| chromosome 7 open reading frame 51 | C7orf51 | Q6U9Y3 |
| calcineurin binding protein 1 | CABIN1 | Q8IVX9 |
| caspase 8 associated protein 2 | CASP8AP2 | Q5T791 |
| coiled-coil domain containing 144C | CCDC144C | Q6ZU57 |
| cyclin Y-like 1 | CCNYL1 | Q8N7R7 |
| CD164 sialomucin-like 2 | CD164L2 | B2RPJ0 |
| CD93 molecule | CD93 | O00274 |
| centromere protein J | CENPJ | Q569I1 |
| centromere protein T | CENPT | Q9H901 |
| cartilage intermediate layer protein 2 | CILP2 | Q6NV88 |
| CAP-GLY domain containing linker protein 2 | CLIP2 | O43611 |
| colipase, pancreatic | CLPS | Q5T9G7 |
| cytochrome b-561 | CYB561 | Q9BWR9 |
| DAB2 interacting protein | DAB2IP | Q5VWQ8 |
| DENN/MADD domain containing 2A | DENND2A | Q86XY0 |
| DENN/MADD domain containing 5A | DENND5A | Q9H6U7 |
| diacylglycerol kinase, delta 130kDa | DGKD | Q16760 |
| dehydrogenase/reductase (SDR family) member 12 | DHRS12 | Q9H8H1 |
| Hypothetical protein | DKFZp451F173 | Q86T62 |
| dopey family member 2 | DOPEY2 | Q9UEZ3 |
| enoyl-Coenzyme A, hydratase/3-hydroxyacyl Coenzyme A dehydrogenase | EHHADH | A8K6Y3 |
| endothelial PAS domain protein 1 | EPAS1 | Q53SM6 |
| ets variant 2 | ETV2 | B5MD42 |
| enhancer of zeste homolog 2 (Drosophila) | EZH2 | Q6R125 |
| fumarylacetoacetate hydrolase domain containing 1 | FAHD1 | Q6FIC7 |
| family with sequence similarity 71, member B | FAM71B | Q8TEW9 |
| fibrillin 2 | FBN2 | B4DU01 |
| fidgetin | FIGN | Q9H6M5 |
| filamin A, alpha (actin binding protein 280) | FLNA | Q86TQ3 |
| flavin containing monooxygenase 3 | FMO3 | B2R816 |
| forkhead box K2 | FOXK2 | Q13622 |
| FRY-like | FRYL | Q6ZR29 |
| follistatin-like 3 (secreted glycoprotein) | FSTL3 | O95633 |
| glutaryl-Coenzyme A dehydrogenase | GCDH | O14719 |
| GLI family zinc finger 2 | GLI2 | O60252 |
| GLI family zinc finger 3 | GLI3 | P10071 |
| hepatoma-derived growth factor-related protein 2 | HDGFRP2 | Q96GI5 |
| solute carrier family 25, member 47 | HDMCP | Q6Q0C1.1 |
| HECT, C2 and WW domain containing E3 ubiquitin protein ligase 1 | HECW1 | Q76N89 |
| Holliday junction recognition protein | HJURP | Q9NSL8 |
| heterogeneous nuclear ribonucleoprotein U (scaffold attachment factor A) | HNRNPU | Q5RI19 |
| heterogeneous nuclear ribonucleoprotein U-like 1 | HNRNPUL1 | Q96G37 |
| short chain dehydrogenase/reductase family 42E, member 1 | HSPC105 | Q8WUS8.2 |
| integrin, alpha 9 | ITGA9 | Q14638 |
| integrin, beta 5 | ITGB5 | P18084 |
| inter-alpha (globulin) inhibitor H5-like | ITIH5L | A6NN03 |
| Janus kinase and microtubule interacting protein 2 | JAKMIP2 | Q96AA8 |
| KIAA0284 | KIAA0284 | Q9Y4F5 |
| KIAA0467; chromosome 1 open reading frame 84 | KIAA0467 | A7E2X4 |
| kinesin family member 26B | KIF26B | Q9NWB4 |
| kallikrein B, plasma (Fletcher factor) 1 | KLKB1 | Q17RE8 |
| keratin 3 | KRT3 | Q701L8 |
| LEM domain containing 2 | LEMD2 | Q5T972 |
| similar to Dual specificity protein kinase CLK2 (CDC like kinase 2) | LOC402468 | CH236948.1 |
| leucine-rich repeats and guanylate kinase domain containing | LRGUK | Q96M69 |
| leucine rich repeat containing 27 | LRRC27 | Q5SZH9 |
| leucine zipper protein 3, pseudogene | LUZPP1 | Q9BQQ8 |
| membrane associated guanylate kinase, WW and PDZ domain containing 2 | MAGI2 | Q9UDU1 |
| MDN1, midasin homolog (yeast) | MDN1 | Q6AI22 |
| mediator complex subunit 13 | MED13 | Q9UHV7 |
| motile sperm domain containing 1 | MOSPD1 | Q5H9C5 |
| M-phase phosphoprotein 9 | MPHOSPH9 | Q9H976 |
| mucin 6, oligomeric mucus/gel-forming | MUC6 | Q4L207 |
| v-myb myeloblastosis viral oncogene homolog (avian) | MYB | P78525 |
| myosin, heavy chain 15 | MYH15 | Q9Y2K3 |
| myosin, heavy chain 16 | MYH16 | BAB15219.1 |
| N-acetylated alpha-linked acidic dipeptidase 2 | NAALAD2 | Q9Y3Q0 |
| sodium leak channel, non-selective | NALCN | Q6ZMI7 |
| nuclear cap binding protein subunit 1, 80kDa | NCBP1 | Q09161 |
| nuclear receptor coactivator 2 | NCOA2 | Q15596 |
| nuclear receptor co-repressor 1 | NCOR1 | Q86YY0 |
| nexilin (F actin binding protein) | NEXN | Q96DL0 |
| NFS1 nitrogen fixation 1 homolog (S. cerevisiae) | NFS1 | Q9NTZ5 |
| nardilysin (N-arginine dibasic convertase) | NRD1 | Q5TFB9 |
| 5'-nucleotidase, cytosolic II | NT5C2 | Q5JUV5 |
| nuclear fragile X mental retardation protein interacting protein 1 | NUFIP1 | Q96SG1 |
| nuclear fragile X mental retardation protein interacting protein 2 | NUFIP2 | A1L3A7 |
| 2',5'-oligoadenylate synthetase 1, 40/46kDa | OAS1 | P00973 |
| olfactory receptor, family 10, subfamily J, member 6 pseudogene | OR10J6P | Q8NGY7 |
| poly(A) binding protein, nuclear 1 | PABPN1 | O43484 |
| phosphoprotein associated with glycosphingolipid microdomains 1 | PAG1 | Q2M1Z9 |
| p21 protein (Cdc42/Rac)-activated kinase 2 | PAK2 | Q13154 |
| similar to poly (ADP-ribose) glycohydrolase; poly (ADP-ribose) glycohydrolase | PARG | Q9Y4W7 |
| prolyl-tRNA synthetase 2, mitochondrial (putative) | PARS2 | Q7L3T8 |
| proprotein convertase subtilisin/kexin type 9 | PCSK9 | C0JYY9 |
| peroxisomal biogenesis factor 1 | PEX1 | Q96S72 |
| phosphogluconate dehydrogenase | PGD | A8K2Y9 |
| PHD and ring finger domains 1 | PHRF1 | B7ZM64 |
| phytanoyl-CoA 2-hydroxylase interacting protein-like | PHYHIPL | Q68DF3 |
| PIP5K1A and PSMD4-like, pseudogene | PIPSL | CAM19360.1 |
| phospholipase D2 | PLD2 | O43580 |
| pleckstrin homology domain containing, family H (with MyTH4 domain) member 1 | PLEKHH1 | Q6PJL4 |
| pogo transposable element with ZNF domain | POGZ | Q9Y4X7 |
| POM121-like protein | POM121L1 | Q3SYA9 |
| proteasome (prosome, macropain) subunit, alpha type, 3 | PSMA3 | P25788 |
| proteasome (prosome, macropain) subunit, beta type, 7 | PSMB7 | Q5TBG6 |
| PWP2 periodic tryptophan protein homolog (yeast) | PWP2 | Q96A77 |
| RAB37, member RAS oncogene family | RAB37 | Q8IWA7 |
| ribonucleoprotein, PTB-binding 1 | RAVER1 | A6NMU4 |
| RNA binding motif protein 10 | RBM10 | Q6PKH5 |
| regulatory factor X, 4 (influences HLA class II expression) | RFX4 | Q6YM53 |
| ring finger protein 31 | RNF31 | Q96EP0 |
| regulation of nuclear pre-mRNA domain containing 1B | RPRD1B | Q9NQG5 |
| SAM and SH3 domain containing 1 | SASH1 | Q6P4R9 |
| secretory carrier membrane protein 3 | SCAMP3 | Q96FR8 |
| sodium channel, voltage-gated, type I, alpha subunit | SCN1A | Q585T7 |
| SEC22 vesicle trafficking protein homolog B (S. cerevisiae) | SEC22B | O75396 |
| serpin peptidase inhibitor, clade I (pancpin), member 2 | SERPINI2 | O75830 |
| SH2 domain containing 3C | SH2D3C | Q5JU30 |
| soc-2 suppressor of clear homolog (C. elegans) | SHOC2 | Q5VZS9 |
| solute carrier family 25 (mitochondrial carrier; phosphate carrier), member 24 | SLC25A24 | Q6NUK1 |
| solute carrier family 5 (sodium/glucose cotransporter), member 10 | SLC5A10 | Q6P5X0 |
| SLU7 splicing factor homolog (S. cerevisiae) | SLU7 | Q96FM9 |
| SWI/SNF related, matrix associated, actin dependent regulator of chromatin, subfamily a, member 2 | SMARCA2 | P51531 |
| smoothelin | SMTN | Q8WWW1 |
| sorting nexin family member 30 | SNX30 | Q5VWJ9 |
| sperm associated antigen 1 | SPAG1 | Q7Z5G1 |
| spectrin, beta, non-erythrocytic 5 | SPTBN5 | Q9NRC6 |
| ST6 (alpha-N-acetyl-neuraminyl-2,3-beta-galactosyl-1,3)-N-acetylgalactosaminide alpha-2,6-sialyltransferase 1 | ST6GALNAC1 | Q9NSC6 |
| StAR-related lipid transfer (START) domain containing 7 | STARD7 | Q6GU43 |
| sushi, von Willebrand factor type A, EGF and pentraxin domain containing 1 | SVEP1 | B3KQM1 |
| synaptonemal complex protein 2 | SYCP2 | Q9BX26 |
| synaptopodin 2-like | SYNPO2L | A5PKV9 |
| tetratricopeptide repeat, ankyrin repeat and coiled-coil containing 1 | TANC1 | Q9C0D5 |
| testis expressed 9 | TEX9 | Q8N6V9 |
| transferrin receptor 2 | TFR2 | Q1HE13 |
| thrombospondin, type I, domain containing 7A | THSD7A | Q9UPZ6 |
| transmembrane protein 132C | TMEM132C | Q8N3T6 |
| transmembrane protein 39B | TMEM39B | Q9NW51 |
| testis specific, 10 | TSGA10 | Q9BZW7 |
| tetratricopeptide repeat domain 27 | TTC27 | Q9NWR4 |
| UV radiation resistance associated gene | UVRAG | Q6P1X0 |
| Wolf-Hirschhorn syndrome candidate 2 | WHSC2 | Q53GS8 |
| WWC family member 3 | WWC3 | Q659C1 |
| zinc finger and BTB domain containing 1 | ZBTB1 | Q86SW8 |
| zinc finger, CW type with PWWP domain 1 | ZCWPW1 | Q9BUD0 |
| zinc finger protein 36, C3H type, homolog (mouse) | ZFP36 | P26651 |
| zinc finger, MYM-type 2 | ZMYM2 | Q9H0V5 |
| zinc finger protein 296 | ZNF296 | Q8WUU4 |
| zinc finger protein 41 | ZNF41 | Q96LE8 |
| zinc finger protein 638 | ZNF638 | Q57Z90 |
| zinc finger protein 671 | ZNF671 | Q9H5E9 |
| zinc finger protein 679; zinc finger protein 735 | ZNF735 | P0CB33 |
