# Supplementary material for: VENNTURE–A Novel Venn Diagram Investigational Tool for Multiple Pharmacological Dataset Analysis
Source: PLoS One. 2012 May 14;7(5):e36911. doi: 10.1371/journal.pone.0036911 (PMC3351456; doi:10.1371/journal.pone.0036911)
Supplement: Table S17 — GO term groups populated by extracted phosphoproteins in non-stimulated control-state SH-SY5Y cells. GO term groups were considered enriched only if at least two proteins were present in each group and with a probability of ≤0.05. Hybrid GO term group scores were generated by multiplication of the GO term group enrichment score with the negative log10 of the probability result. (DOC) [file pone.0036911.s018.doc]

**Table S17**. GO term groups populated by extracted phosphoproteins in non-stimulated control-state SH-SY5Y cells. GO term groups were considered enriched only if at least two proteins were present in each group and with a probability of ≤0.05. Hybrid GO term group scores were generated by multiplication of the GO term group enrichment score with the negative log_10_ of the probability result.

| **GO term** | **GO term ID** | **Enrichment** | **Probability** | **Hybrid** |
| --- | --- | --- | --- | --- |
| ATP-dependent protein binding | GO:0043008 | 51.99 | 0.008 | 109.01835 |
| Mre11 complex | GO:0030870 | 39.33 | 0.0042 | 93.477605 |
| microtubule depolymerization | GO:0007019 | 20.33 | 0.0002 | 75.20006 |
| histone acetyl-lysine binding | GO:0070577 | 38.99 | 0.0129 | 73.668107 |
| telomerase activity | GO:0003720 | 38.99 | 0.0129 | 73.668107 |
| heterogeneous nuclear ribonucleoprotein complex | GO:0030530 | 18.51 | 0.0003 | 65.208486 |
| telomeric DNA binding | GO:0042162 | 20.8 | 0.001 | 62.4 |
| negative regulation of microtubule depolymerization | GO:0007026 | 20.6 | 0.001 | 61.8 |
| regulation of microtubule depolymerization | GO:0031114 | 20.6 | 0.001 | 61.8 |
| negative regulation of microtubule polymerization or depolymerization | GO:0031111 | 19.31 | 0.0011 | 57.130707 |
| lamin binding | GO:0005521 | 31.2 | 0.0193 | 53.490612 |
| intracellular non-membrane-bounded organelle | GO:0043232 | 2.72 | 1.15E-18 | 48.794902 |
| non-membrane-bounded organelle | GO:0043228 | 2.72 | 1.15E-18 | 48.794902 |
| protein depolymerization | GO:0051261 | 11.76 | 0.0001 | 47.04 |
| negative regulation of protein complex disassembly | GO:0043242 | 11.59 | 0.0005 | 38.258938 |
| microtubule polymerization or depolymerization | GO:0031109 | 12.88 | 0.0011 | 38.106862 |
| SWI/SNF complex | GO:0016514 | 15.73 | 0.0038 | 38.070004 |
| SWI/SNF-type complex | GO:0070603 | 15.73 | 0.0038 | 38.070004 |
| actin-dependent ATPase activity | GO:0030898 | 22.28 | 0.0285 | 34.426057 |
| chromatin remodeling | GO:0006338 | 9.32 | 0.0004 | 31.668801 |
| cytoskeleton | GO:0005856 | 2.97 | 2.25E-11 | 31.624018 |
| nuclear part | GO:0044428 | 2.69 | 6.06E-12 | 30.175149 |
| RNA-directed DNA polymerase activity | GO:0003964 | 19.5 | 0.034 | 28.636161 |
| regulation of cell morphogenesis | GO:0022604 | 6.64 | 8.33E-05 | 27.086917 |
| regulation of protein complex disassembly | GO:0043244 | 9.09 | 0.0011 | 26.89374 |
| cellular protein complex disassembly | GO:0043624 | 8.45 | 0.0007 | 26.658922 |
| protein complex disassembly | GO:0043241 | 8.19 | 0.0008 | 25.363693 |
| regulation of cell shape | GO:0008360 | 8.58 | 0.0014 | 24.486221 |
| negative regulation of organelle organization | GO:0010639 | 7.36 | 0.0005 | 24.295581 |
| negative regulation of cytoskeleton organization | GO:0051494 | 8.43 | 0.0014 | 24.058141 |
| RNA splicing | GO:0008380 | 4.76 | 1.35E-05 | 23.179611 |
| nucleus | GO:0005634 | 1.82 | 6.74E-13 | 22.151839 |
| cellular macromolecular complex disassembly | GO:0034623 | 7.41 | 0.0011 | 21.92328 |
| mRNA processing | GO:0006397 | 4.43 | 1.35E-05 | 21.572621 |
| macromolecular complex disassembly | GO:0032984 | 7.21 | 0.0011 | 21.331559 |
| ADP binding | GO:0043531 | 12.31 | 0.0193 | 21.10479 |
| chromatin remodeling complex | GO:0016585 | 6.74 | 0.0015 | 19.033145 |
| cellular component disassembly | GO:0022411 | 6.18 | 0.0011 | 18.284193 |
| nuclear mRNA splicing, via spliceosome | GO:0000398 | 5.31 | 0.0004 | 18.043061 |
| RNA splicing, via transesterification reactions with bulged adenosine as nucleophile | GO:0000377 | 5.31 | 0.0004 | 18.043061 |
| RNA splicing, via transesterification reactions | GO:0000375 | 5.31 | 0.0004 | 18.043061 |
| regulation of cell projection organization | GO:0031344 | 6.36 | 0.0017 | 17.614345 |
| nuclear lumen | GO:0031981 | 2.48 | 1.22E-07 | 17.145828 |
| chromosome | GO:0005694 | 3.47 | 1.63E-05 | 16.613709 |
| spindle | GO:0005819 | 4.99 | 0.0005 | 16.47214 |
| intracellular part | GO:0044424 | 1.35 | 6.74E-13 | 16.431309 |
| mRNA metabolic process | GO:0016071 | 3.86 | 6.89E-05 | 16.064474 |
| intracellular | GO:0005622 | 1.33 | 8.65E-13 | 16.043769 |
| intracellular organelle part | GO:0044446 | 1.81 | 1.69E-09 | 15.877525 |
| organelle part | GO:0044422 | 1.8 | 2.22E-09 | 15.576565 |
| nuclear speck | GO:0016607 | 5.35 | 0.0015 | 15.107912 |
| chromosomal part | GO:0044427 | 3.54 | 5.49E-05 | 15.081914 |
| intracellular organelle | GO:0043229 | 1.42 | 5.25E-11 | 14.597374 |
| organelle | GO:0043226 | 1.42 | 5.45E-11 | 14.574317 |
| structural constituent of cytoskeleton | GO:0005200 | 6.32 | 0.007 | 13.61898 |
| microtubule cytoskeleton | GO:0015630 | 3.09 | 3.94E-05 | 13.609917 |
| microtubule | GO:0005874 | 3.83 | 0.0003 | 13.492626 |
| spliceosomal complex | GO:0005681 | 4.73 | 0.0015 | 13.357088 |
| cytoskeletal protein binding | GO:0008092 | 3.32 | 0.0001 | 13.28 |
| cell cortex | GO:0005938 | 4.7 | 0.0015 | 13.272371 |
| negative regulation of cellular component organization | GO:0051129 | 4.76 | 0.0017 | 13.183063 |
| nucleolus | GO:0005730 | 2.83 | 2.39E-05 | 13.079134 |
| RNA processing | GO:0006396 | 3.2 | 8.33E-05 | 13.053936 |
| membrane-enclosed lumen | GO:0031974 | 2.15 | 1.42E-06 | 12.57258 |
| phosphoprotein binding | GO:0051219 | 8.67 | 0.0356 | 12.558929 |
| regulation of cellular component organization | GO:0051128 | 3.37 | 0.0002 | 12.465529 |
| intracellular organelle lumen | GO:0070013 | 2.16 | 2.39E-06 | 12.142661 |
| nuclear body | GO:0016604 | 4.27 | 0.0015 | 12.05809 |
| cytoskeletal part | GO:0044430 | 2.55 | 2.11E-05 | 11.92308 |
| organelle organization | GO:0006996 | 2.42 | 1.35E-05 | 11.784592 |
| histone binding | GO:0042393 | 7.09 | 0.0232 | 11.58869 |
| organelle lumen | GO:0043233 | 2.11 | 4.46E-06 | 11.289903 |
| protein binding | GO:0005515 | 1.43 | 1.68E-08 | 11.117808 |
| regulation of anatomical structure morphogenesis | GO:0022603 | 3.99 | 0.0017 | 11.050509 |
| RNA binding | GO:0003723 | 2.73 | 0.0002 | 10.098188 |
| condensed chromosome | GO:0000793 | 4.37 | 0.005 | 10.055501 |
| actin filament binding | GO:0051015 | 6.5 | 0.0285 | 10.043508 |
| calmodulin binding | GO:0005516 | 4.46 | 0.007 | 9.6108627 |
| cellular component organization | GO:0016043 | 1.9 | 1.71E-05 | 9.0573074 |
| enzyme binding | GO:0019899 | 2.93 | 0.001 | 8.79 |
| cytoskeleton organization | GO:0007010 | 2.97 | 0.0011 | 8.7870637 |
| chromosome organization | GO:0051276 | 2.83 | 0.0014 | 8.0764577 |
| negative regulation of nucleobase, nucleoside, nucleotide and nucleic acid metabolic process | GO:0045934 | 2.83 | 0.0014 | 8.0764577 |
| macromolecular complex | GO:0032991 | 1.71 | 2.65E-05 | 7.8262496 |
| negative regulation of nitrogen compound metabolic process | GO:0051172 | 2.79 | 0.0016 | 7.8005052 |
| nucleoplasm | GO:0005654 | 2.29 | 0.0006 | 7.3780336 |
| cytosol | GO:0005829 | 2.08 | 0.0003 | 7.3275878 |
| actin binding | GO:0003779 | 3.15 | 0.0064 | 6.9105331 |
| intracellular membrane-bounded organelle | GO:0043231 | 1.33 | 2.01E-05 | 6.2467492 |
| membrane-bounded organelle | GO:0043227 | 1.33 | 2.01E-05 | 6.2467492 |
| nucleic acid binding | GO:0003676 | 1.66 | 0.0002 | 6.1402902 |
| cellular macromolecule metabolic process | GO:0044260 | 1.43 | 0.0001 | 5.72 |
| protein domain specific binding | GO:0019904 | 2.94 | 0.0119 | 5.6578919 |
| protein complex | GO:0043234 | 1.68 | 0.0005 | 5.5457304 |
| protein binding, bridging | GO:0030674 | 4.15 | 0.0463 | 5.5378389 |
| nucleobase, nucleoside, nucleotide and nucleic acid metabolic process | GO:0006139 | 1.52 | 0.0005 | 5.0175656 |
| isomerase activity | GO:0016853 | 3.66 | 0.0426 | 5.0163609 |
| protein C-terminus binding | GO:0008022 | 3.63 | 0.0426 | 4.9752432 |
| gene expression | GO:0010467 | 1.51 | 0.0011 | 4.467497 |
| kinase binding | GO:0019900 | 3.23 | 0.0426 | 4.427007 |
| cell part | GO:0044464 | 1.08 | 0.0001 | 4.32 |
| cell | GO:0005623 | 1.08 | 0.0001 | 4.32 |
| nitrogen compound metabolic process | GO:0006807 | 1.45 | 0.0012 | 4.2351872 |
| macromolecule metabolic process | GO:0043170 | 1.34 | 0.001 | 4.02 |
| structural molecule activity | GO:0005198 | 2.2 | 0.0203 | 3.7235087 |
| DNA binding | GO:0003677 | 1.63 | 0.0064 | 3.5759266 |
| binding | GO:0005488 | 1.12 | 0.0028 | 2.859183 |
| nucleotide binding | GO:0000166 | 1.55 | 0.0193 | 2.6573862 |
| ATP binding | GO:0005524 | 1.66 | 0.0285 | 2.5649575 |
| adenyl ribonucleotide binding | GO:0032559 | 1.64 | 0.0338 | 2.4125766 |
