# Supplementary material for: VENNTURE–A Novel Venn Diagram Investigational Tool for Multiple Pharmacological Dataset Analysis
Source: PLoS One. 2012 May 14;7(5):e36911. doi: 10.1371/journal.pone.0036911 (PMC3351456; doi:10.1371/journal.pone.0036911)
Supplement: Table S18 — GO term groups populated by extracted phosphoproteins in 10 nM MeCh-stimulated control-state SH-SY5Y cells. GO term groups were considered enriched only if at least two proteins were present in each group and with a probability of ≤0.05. Hybrid GO term group scores were generated by multiplication of the GO term group enrichment score with the negative log10 of the probability result. (DOC) [file pone.0036911.s019.doc]

**Table S18.** GO term groups populated by extracted phosphoproteins in 10nM MeCh-stimulated control-state SH-SY5Y cells. GO term groups were considered enriched only if at least two proteins were present in each group and with a probability of ≤0.05. Hybrid GO term group scores were generated by multiplication of the GO term group enrichment score with the negative log_10_ of the probability result.

| **GO term** | **GO term ID** | **Enrichment** | **Probability** | **Hybrid** |
| --- | --- | --- | --- | --- |
| establishment of mitotic spindle orientation | GO:0000132 | 34.96 | 0.0257 | 55.588738 |
| establishment of spindle orientation | GO:0051294 | 34.96 | 0.0257 | 55.588738 |
| positive regulation of protein binding | GO:0032092 | 29.97 | 0.0281 | 46.4922716 |
| regulation of centrosome cycle | GO:0046605 | 29.97 | 0.0281 | 46.4922716 |
| establishment of mitotic spindle localization | GO:0040001 | 26.22 | 0.0343 | 38.4045882 |
| axonemal dynein complex | GO:0005858 | 18.67 | 0.024 | 30.2414561 |
| kinetochore | GO:0000776 | 8.84 | 0.0007 | 27.8893333 |
| tRNA binding | GO:0000049 | 15.68 | 0.0274 | 24.4960712 |
| cytoplasmic microtubule | GO:0005881 | 14 | 0.035 | 20.3830474 |
| growth cone | GO:0030426 | 9.33 | 0.007 | 20.1052353 |
| site of polarized growth | GO:0030427 | 9.14 | 0.007 | 19.6958039 |
| axoneme part | GO:0044447 | 13.18 | 0.0373 | 18.8248776 |
| heterogeneous nuclear ribonucleoprotein complex | GO:0030530 | 13.18 | 0.0373 | 18.8248776 |
| dynein complex | GO:0030286 | 9.88 | 0.02 | 16.7858236 |
| chromosome | GO:0005694 | 3.95 | 8.88E-05 | 16.0037688 |
| spliceosomal complex | GO:0005681 | 5.89 | 0.002 | 15.8969333 |
| RNA modification | GO:0009451 | 8.93 | 0.0224 | 14.7322852 |
| RNA splicing | GO:0008380 | 4.67 | 0.001 | 14.01 |
| mRNA processing | GO:0006397 | 4.44 | 0.001 | 13.32 |
| regulation of microtubule cytoskeleton organization | GO:0070507 | 9.83 | 0.0461 | 13.1358199 |
| nuclear part | GO:0044428 | 2.44 | 5.58E-06 | 12.8182126 |
| nuclear mRNA splicing, via spliceosome | GO:0000398 | 5.24 | 0.004 | 12.5652056 |
| RNA splicing, via transesterification reactions with bulged adenosine as nucleophile | GO:0000377 | 5.24 | 0.004 | 12.5652056 |
| RNA splicing, via transesterification reactions | GO:0000375 | 5.24 | 0.004 | 12.5652056 |
| chromosome, centromeric region | GO:0000775 | 5.6 | 0.0061 | 12.4021529 |
| microtubule associated complex | GO:0005875 | 5.77 | 0.0126 | 10.960862 |
| chromosomal part | GO:0044427 | 3.56 | 0.001 | 10.68 |
| macromolecular complex | GO:0032991 | 2.03 | 5.58E-06 | 10.6643326 |
| mRNA metabolic process | GO:0016071 | 3.86 | 0.0021 | 10.3362335 |
| RNA processing | GO:0006396 | 3.4 | 0.001 | 10.2 |
| organelle localization | GO:0051640 | 6.1 | 0.0266 | 9.60822202 |
| ribonucleoprotein complex | GO:0030529 | 3.16 | 0.001 | 9.48 |
| intracellular non-membrane-bounded organelle | GO:0043232 | 2.02 | 3.66E-05 | 8.96176821 |
| non-membrane-bounded organelle | GO:0043228 | 2.02 | 3.66E-05 | 8.96176821 |
| establishment of organelle localization | GO:0051656 | 6.56 | 0.0445 | 8.86675833 |
| nucleosome organization | GO:0034728 | 5.64 | 0.0288 | 8.68902637 |
| chromatin assembly or disassembly | GO:0006333 | 4.88 | 0.0266 | 7.68657761 |
| response to DNA damage stimulus | GO:0006974 | 3.43 | 0.0064 | 7.52480269 |
| protein-DNA complex | GO:0032993 | 5.09 | 0.0336 | 7.50093308 |
| condensed chromosome | GO:0000793 | 4.44 | 0.0258 | 7.05240851 |
| RNA binding | GO:0003723 | 2.92 | 0.004 | 7.00198483 |
| macromolecular complex assembly | GO:0065003 | 2.72 | 0.004 | 6.52239682 |
| microtubule-based process | GO:0007017 | 3.66 | 0.0187 | 6.32505972 |
| nucleus | GO:0005634 | 1.58 | 0.0001 | 6.32 |
| macromolecular complex subunit organization | GO:0043933 | 2.61 | 0.004 | 6.25862342 |
| cellular macromolecular complex assembly | GO:0034622 | 3.26 | 0.0163 | 5.82826841 |
| microtubule | GO:0005874 | 3.36 | 0.0187 | 5.8066122 |
| protein complex | GO:0043234 | 1.84 | 0.0007 | 5.80501961 |
| cellular component biogenesis | GO:0044085 | 2.32 | 0.004 | 5.56322082 |
| cellular macromolecular complex subunit organization | GO:0034621 | 3.03 | 0.0163 | 5.41707156 |
| nuclear lumen | GO:0031981 | 2.04 | 0.0028 | 5.20779762 |
| cellular component assembly | GO:0022607 | 2.37 | 0.0064 | 5.19935346 |
| DNA repair | GO:0006281 | 3.3 | 0.0281 | 5.11926914 |
| intracellular organelle part | GO:0044446 | 1.6 | 0.0007 | 5.04784314 |
| organelle part | GO:0044422 | 1.59 | 0.0007 | 5.01629412 |
| cellular response to DNA damage stimulus | GO:0034984 | 3.15 | 0.0257 | 5.00871066 |
| chromosome organization | GO:0051276 | 2.78 | 0.0187 | 4.80428033 |
| nuclear chromosome | GO:0000228 | 3.59 | 0.0466 | 4.78049456 |
| microtubule cytoskeleton | GO:0015630 | 2.51 | 0.0196 | 4.28643726 |
| nucleoplasm | GO:0005654 | 2.21 | 0.0125 | 4.20582887 |
| intracellular | GO:0005622 | 1.23 | 0.0004 | 4.17946621 |
| intracellular part | GO:0044424 | 1.23 | 0.0007 | 3.88052941 |
| gene expression | GO:0010467 | 1.6 | 0.004 | 3.83670401 |
| nucleolus | GO:0005730 | 2.26 | 0.0201 | 3.83477691 |
| cellular component organization | GO:0016043 | 1.72 | 0.0064 | 3.77337044 |
| cellular macromolecule metabolic process | GO:0044260 | 1.43 | 0.0028 | 3.65056402 |
| organelle organization | GO:0006996 | 1.98 | 0.0151 | 3.60562564 |
| macromolecule metabolic process | GO:0043170 | 1.38 | 0.004 | 3.30915721 |
| nucleotide binding | GO:0000166 | 1.78 | 0.0152 | 3.23631841 |
| cytosol | GO:0005829 | 1.88 | 0.0201 | 3.18999141 |
| cytoskeleton | GO:0005856 | 1.82 | 0.0223 | 3.00608515 |
| intracellular organelle lumen | GO:0070013 | 1.73 | 0.0196 | 2.954397 |
| cytoskeletal part | GO:0044430 | 1.93 | 0.035 | 2.80994867 |
| organelle lumen | GO:0043233 | 1.69 | 0.0223 | 2.79136478 |
| membrane-enclosed lumen | GO:0031974 | 1.65 | 0.0258 | 2.62082749 |
| nucleobase, nucleoside, nucleotide and nucleic acid metabolic process | GO:0006139 | 1.47 | 0.018 | 2.56474942 |
| RNA metabolic process | GO:0016070 | 1.6 | 0.0266 | 2.52018938 |
| protein binding | GO:0005515 | 1.28 | 0.0152 | 2.32724021 |
| nucleic acid binding | GO:0003676 | 1.53 | 0.0355 | 2.21815062 |
| nitrogen compound metabolic process | GO:0006807 | 1.39 | 0.0309 | 2.09895771 |
| regulation of macromolecule metabolic process | GO:0060255 | 1.46 | 0.0464 | 1.94688375 |
| cellular metabolic process | GO:0044237 | 1.25 | 0.0281 | 1.9391171 |
| intracellular organelle | GO:0043229 | 1.19 | 0.0258 | 1.89017255 |
| organelle | GO:0043226 | 1.19 | 0.0259 | 1.88817328 |
| primary metabolic process | GO:0044238 | 1.23 | 0.0464 | 1.64018288 |
