# Supplementary material for: VENNTURE–A Novel Venn Diagram Investigational Tool for Multiple Pharmacological Dataset Analysis
Source: PLoS One. 2012 May 14;7(5):e36911. doi: 10.1371/journal.pone.0036911 (PMC3351456; doi:10.1371/journal.pone.0036911)
Supplement: Table S19 — GO term groups populated by extracted phosphoproteins in 100 nM MeCh-stimulated control-state SH-SY5Y cells. GO term groups were considered enriched only if at least two proteins were present in each group and with a probability of ≤0.05. Hybrid GO term group scores were generated by multiplication of the GO term group enrichment score with the negative log10 of the probability result. (DOC) [file pone.0036911.s020.doc]

**Table S19.** GO term groups populated by extracted phosphoproteins in 100nM MeCh-stimulated control-state SH-SY5Y cells. GO term groups were considered enriched only if at least two proteins were present in each group and with a probability of ≤0.05. Hybrid GO term group scores were generated by multiplication of the GO term group enrichment score with the negative log_10_ of the probability result.

| **GO term** | **GO term ID** | **Enrichment** | **Probability** | **Hybrid** |
| --- | --- | --- | --- | --- |
| protection from non-homologous end joining at telomere | GO:0031848 | 82.59 | 0.0076 | 175.0236054 |
| telomere maintenance in response to DNA damage | GO:0043247 | 82.59 | 0.0076 | 175.0236054 |
| Mre11 complex | GO:0030870 | 63 | 0.0027 | 161.8240829 |
| heterogeneous nuclear ribonucleoprotein complex | GO:0030530 | 29.65 | 6.96E-05 | 123.266636 |
| CRD-mediated mRNA stability complex | GO:0070937 | 50.4 | 0.0037 | 122.5626331 |
| telomeric DNA binding | GO:0042162 | 32.78 | 0.0002 | 121.2522367 |
| telomere capping | GO:0016233 | 61.94 | 0.0112 | 120.8314357 |
| CRD-mediated mRNA stabilization | GO:0070934 | 49.55 | 0.0143 | 91.40309934 |
| protein localization to chromosome, telomeric region | GO:0070198 | 41.29 | 0.0187 | 71.35566007 |
| determination of adult lifespan | GO:0008340 | 35.39 | 0.0215 | 59.0150029 |
| protein localization to chromosome | GO:0034502 | 35.39 | 0.0215 | 59.0150029 |
| telomere maintenance via telomerase | GO:0007004 | 26.55 | 0.0076 | 56.26439912 |
| telomere maintenance via telomere lengthening | GO:0010833 | 26.55 | 0.0076 | 56.26439912 |
| nuclear telomere cap complex | GO:0000783 | 28 | 0.0113 | 54.51380358 |
| telomere cap complex | GO:0000782 | 28 | 0.0113 | 54.51380358 |
| nuclear mRNA splicing, via spliceosome | GO:0000398 | 8.52 | 1.13E-05 | 42.14777166 |
| RNA splicing, via transesterification reactions with bulged adenosine as nucleophile | GO:0000377 | 8.52 | 1.13E-05 | 42.14777166 |
| RNA splicing, via transesterification reactions | GO:0000375 | 8.52 | 1.13E-05 | 42.14777166 |
| RNA-dependent DNA replication | GO:0006278 | 20.65 | 0.0112 | 40.28364783 |
| double-stranded RNA binding | GO:0003725 | 14.9 | 0.0024 | 39.0348525 |
| Rac GTPase binding | GO:0048365 | 22.35 | 0.0374 | 31.89631969 |
| RNA binding | GO:0003723 | 4.3 | 7.41E-08 | 30.65978171 |
| RNA splicing | GO:0008380 | 5.94 | 1.13E-05 | 29.38471405 |
| nuclear part | GO:0044428 | 3.03 | 2.78E-10 | 28.95454427 |
| intracellular non-membrane-bounded organelle | GO:0043232 | 2.62 | 1.26E-10 | 25.93702917 |
| non-membrane-bounded organelle | GO:0043228 | 2.62 | 1.26E-10 | 25.93702917 |
| negative regulation of DNA metabolic process | GO:0051053 | 12.71 | 0.0097 | 25.58813126 |
| microtubule-based transport | GO:0010970 | 14.29 | 0.0215 | 23.82945441 |
| cytoplasmic microtubule | GO:0005881 | 15.75 | 0.0309 | 23.78315395 |
| NF-kappaB binding | GO:0051059 | 17.56 | 0.0443 | 23.76915057 |
| mRNA processing | GO:0006397 | 5.24 | 4.04E-05 | 23.02256165 |
| organelle part | GO:0044422 | 2.18 | 3.37E-11 | 22.82976682 |
| intracellular organelle part | GO:0044446 | 2.17 | 4.52E-11 | 22.4483496 |
| beta-tubulin binding | GO:0048487 | 16.39 | 0.0443 | 22.18544293 |
| protein kinase C activity | GO:0004697 | 16.39 | 0.0443 | 22.18544293 |
| RNA processing | GO:0006396 | 4.23 | 1.13E-05 | 20.92547818 |
| nuclear lumen | GO:0031981 | 2.91 | 1.96E-07 | 19.51953483 |
| maintenance of location in cell | GO:0051651 | 10.32 | 0.0143 | 19.03693209 |
| nucleolus | GO:0005730 | 3.63 | 5.78E-06 | 19.01420195 |
| p53 binding | GO:0002039 | 14.46 | 0.0489 | 18.9525939 |
| intracellular organelle lumen | GO:0070013 | 2.66 | 1.96E-07 | 17.84259885 |
| membrane-enclosed lumen | GO:0031974 | 2.62 | 1.96E-07 | 17.57428909 |
| organelle lumen | GO:0043233 | 2.6 | 3.11E-07 | 16.91882299 |
| mRNA metabolic process | GO:0016071 | 4.56 | 0.0002 | 16.86730322 |
| nuclear chromosome, telomeric region | GO:0000784 | 12 | 0.0498 | 15.63324789 |
| ubiquitin protein ligase binding | GO:0031625 | 10.24 | 0.0369 | 14.67365001 |
| microtubule-based movement | GO:0007018 | 6.58 | 0.0097 | 13.24704199 |
| ribonucleoprotein complex | GO:0030529 | 3.56 | 0.0003 | 12.54144833 |
| nucleus | GO:0005634 | 1.77 | 1.96E-07 | 11.87270675 |
| damaged DNA binding | GO:0003684 | 8.38 | 0.0443 | 11.34313677 |
| intracellular organelle | GO:0043229 | 1.46 | 3.90E-08 | 10.81704567 |
| maintenance of location | GO:0051235 | 6.45 | 0.0215 | 10.75577193 |
| organelle | GO:0043226 | 1.45 | 3.90E-08 | 10.74295632 |
| regulation of cellular response to stress | GO:0080135 | 6.26 | 0.0215 | 10.43893524 |
| intracellular part | GO:0044424 | 1.36 | 2.15E-08 | 10.42788369 |
| cytosol | GO:0005829 | 2.52 | 0.0001 | 10.08 |
| structural constituent of cytoskeleton | GO:0005200 | 6.64 | 0.0369 | 9.514944929 |
| macromolecular complex | GO:0032991 | 1.95 | 1.46E-05 | 9.429511931 |
| gene expression | GO:0010467 | 1.89 | 1.13E-05 | 9.349681742 |
| regulation of response to stress | GO:0080134 | 4.32 | 0.0076 | 9.154885281 |
| structure-specific DNA binding | GO:0043566 | 5.8 | 0.027 | 9.098090168 |
| intracellular | GO:0005622 | 1.31 | 1.98E-07 | 8.781368601 |
| spliceosomal complex | GO:0005681 | 4.74 | 0.0196 | 8.094706222 |
| double-stranded DNA binding | GO:0003690 | 5.4 | 0.0443 | 7.309419878 |
| tubulin binding | GO:0015631 | 5.29 | 0.0455 | 7.099119712 |
| cytoskeleton | GO:0005856 | 2.23 | 0.0007 | 7.035431371 |
| protein binding | GO:0005515 | 1.42 | 2.24E-05 | 6.602647814 |
| protein C-terminus binding | GO:0008022 | 4.76 | 0.0419 | 6.558261251 |
| microtubule-based process | GO:0007017 | 3.84 | 0.0215 | 6.403436314 |
| nucleoplasm | GO:0005654 | 2.49 | 0.0032 | 6.212176554 |
| intracellular membrane-bounded organelle | GO:0043231 | 1.4 | 4.99E-05 | 6.022659236 |
| membrane-bounded organelle | GO:0043227 | 1.4 | 4.99E-05 | 6.022659236 |
| response to DNA damage stimulus | GO:0006974 | 3.38 | 0.0172 | 5.963913849 |
| structural molecule activity | GO:0005198 | 2.85 | 0.0082 | 5.945630521 |
| nuclear chromosome | GO:0000228 | 4.04 | 0.0348 | 5.892019854 |
| protein kinase binding | GO:0019901 | 4.33 | 0.0443 | 5.861071865 |
| guanyl-nucleotide exchange factor activity | GO:0005085 | 4.12 | 0.046 | 5.509437733 |
| cellular response to DNA damage stimulus | GO:0034984 | 3.35 | 0.023 | 5.488211749 |
| enzyme binding | GO:0019899 | 2.92 | 0.0143 | 5.386418771 |
| cellular response to stress | GO:0033554 | 2.88 | 0.0143 | 5.312632212 |
| nucleic acid binding | GO:0003676 | 1.75 | 0.001 | 5.25 |
| binding | GO:0005488 | 1.18 | 3.79E-05 | 5.217205732 |
| chromosome | GO:0005694 | 2.78 | 0.0159 | 5.000115994 |
| negative regulation of macromolecule metabolic process | GO:0010605 | 2.7 | 0.0161 | 4.841570135 |
| protein complex | GO:0043234 | 1.77 | 0.0027 | 4.546486137 |
| negative regulation of metabolic process | GO:0009892 | 2.52 | 0.0215 | 4.202255081 |
| cellular response to stimulus | GO:0051716 | 2.4 | 0.0202 | 4.067156713 |
| cytoskeletal part | GO:0044430 | 2.17 | 0.0134 | 4.064182588 |
| cytoskeletal protein binding | GO:0008092 | 2.74 | 0.0329 | 4.06288324 |
| cellular component organization | GO:0016043 | 1.79 | 0.0076 | 3.79334367 |
| nucleobase, nucleoside, nucleotide and nucleic acid metabolic process | GO:0006139 | 1.61 | 0.0048 | 3.733201608 |
| regulation of macromolecule metabolic process | GO:0060255 | 1.69 | 0.0065 | 3.696176427 |
| cell projection | GO:0042995 | 2.32 | 0.0263 | 3.665702664 |
| regulation of gene expression | GO:0010468 | 1.71 | 0.0097 | 3.442620334 |
| nitrogen compound metabolic process | GO:0006807 | 1.53 | 0.0076 | 3.242355204 |
| cytoplasm | GO:0005737 | 1.3 | 0.0071 | 2.793364147 |
| regulation of metabolic process | GO:0019222 | 1.56 | 0.0172 | 2.752575623 |
| cellular macromolecule metabolic process | GO:0044260 | 1.39 | 0.0112 | 2.711586948 |
| macromolecule metabolic process | GO:0043170 | 1.36 | 0.0134 | 2.547137474 |
| cell part | GO:0044464 | 1.07 | 0.0057 | 2.401213904 |
| cell | GO:0005623 | 1.07 | 0.0057 | 2.401213904 |
