# Supplementary material for: VENNTURE–A Novel Venn Diagram Investigational Tool for Multiple Pharmacological Dataset Analysis
Source: PLoS One. 2012 May 14;7(5):e36911. doi: 10.1371/journal.pone.0036911 (PMC3351456; doi:10.1371/journal.pone.0036911)
Supplement: Table S20 — GO term groups populated by extracted phosphoproteins in 1 µM MeCh-stimulated control-state SH-SY5Y cells. GO term groups were considered enriched only if at least two proteins were present in each group and with a probability of ≤0.05. Hybrid GO term group scores were generated by multiplication of the GO term group enrichment score with the negative log10 of the probability result. (DOC) [file pone.0036911.s021.doc]

**Table S20.** GO term groups populated by extracted phosphoproteins in 1µM MeCh-stimulated control-state SH-SY5Y cells. GO term groups were considered enriched only if at least two proteins were present in each group and with a probability of ≤0.05. Hybrid GO term group scores were generated by multiplication of the GO term group enrichment score with the negative log_10_ of the probability result.

| **GO term** | **GO term ID** | **Enrichment** | **Probability** | **Hybrid** |
| --- | --- | --- | --- | --- |
| alpha-actinin binding | GO:0051393 | 41.03 | 0.045 | 55.25869056 |
| PcG protein complex | GO:0031519 | 27.1 | 0.0128 | 51.29460982 |
| histone ubiquitination | GO:0016574 | 21.97 | 0.0161 | 39.3960355 |
| chromosome, telomeric region | GO:0000781 | 14.02 | 0.0077 | 29.63140003 |
| kinesin complex | GO:0005871 | 15.94 | 0.0328 | 23.65697093 |
| translational initiation | GO:0006413 | 9.55 | 0.012 | 18.3438191 |
| nuclear chromosome, telomeric region | GO:0000784 | 12.91 | 0.0383 | 18.29090383 |
| synaptic vesicle membrane | GO:0030672 | 12.91 | 0.0383 | 18.29090383 |
| nuclear mRNA splicing, via spliceosome | GO:0000398 | 6.59 | 0.0021 | 17.64657485 |
| RNA splicing, via transesterification reactions with bulged adenosine as nucleophile | GO:0000377 | 6.59 | 0.0021 | 17.64657485 |
| RNA splicing, via transesterification reactions | GO:0000375 | 6.59 | 0.0021 | 17.64657485 |
| histone deacetylase complex | GO:0000118 | 9.92 | 0.0182 | 17.26009183 |
| RNA splicing | GO:0008380 | 5.42 | 0.001 | 16.26 |
| chromatin remodeling complex | GO:0016585 | 7.74 | 0.0105 | 15.31599483 |
| nuclear part | GO:0044428 | 2.58 | 5.63E-06 | 13.54368834 |
| mRNA processing | GO:0006397 | 4.78 | 0.0019 | 13.00755779 |
| RNA binding | GO:0003723 | 3.61 | 0.0003 | 12.71759227 |
| nuclear chromosome part | GO:0044454 | 5.79 | 0.0104 | 11.48137697 |
| nuclear chromatin | GO:0000790 | 7.53 | 0.0331 | 11.14569521 |
| mRNA metabolic process | GO:0016071 | 4.16 | 0.0021 | 11.13956773 |
| nuclear chromosome | GO:0000228 | 5.21 | 0.0074 | 11.10130274 |
| macromolecular complex | GO:0032991 | 2.1 | 5.63E-06 | 11.02393237 |
| chromosome | GO:0005694 | 3.58 | 0.0009 | 10.90381182 |
| RNA processing | GO:0006396 | 3.56 | 0.0021 | 9.532899311 |
| nucleus | GO:0005634 | 1.75 | 5.63E-06 | 9.186610309 |
| chromosomal part | GO:0044427 | 3.59 | 0.0037 | 8.730155811 |
| ribonucleoprotein complex | GO:0030529 | 3.28 | 0.0024 | 8.592907127 |
| nuclear speck | GO:0016607 | 5.26 | 0.0328 | 7.806503582 |
| protein complex | GO:0043234 | 1.96 | 0.0006 | 6.314823549 |
| intracellular non-membrane-bounded organelle | GO:0043232 | 1.92 | 0.0006 | 6.185949599 |
| non-membrane-bounded organelle | GO:0043228 | 1.92 | 0.0006 | 6.185949599 |
| cytosol | GO:0005829 | 2.27 | 0.0024 | 5.946920481 |
| organelle part | GO:0044422 | 1.7 | 0.0005 | 5.611750993 |
| intracellular organelle part | GO:0044446 | 1.67 | 0.0006 | 5.380487412 |
| nuclear lumen | GO:0031981 | 2.09 | 0.0049 | 4.827490193 |
| intracellular membrane-bounded organelle | GO:0043231 | 1.37 | 0.0006 | 4.413932787 |
| membrane-bounded organelle | GO:0043227 | 1.37 | 0.0006 | 4.413932787 |
| intracellular organelle | GO:0043229 | 1.32 | 0.0006 | 4.252840349 |
| organelle | GO:0043226 | 1.32 | 0.0006 | 4.252840349 |
| intracellular | GO:0005622 | 1.24 | 0.0006 | 3.99509245 |
| intracellular part | GO:0044424 | 1.24 | 0.0008 | 3.840168416 |
| nucleoplasm | GO:0005654 | 2.21 | 0.0227 | 3.633182856 |
| cytoskeletal part | GO:0044430 | 2.05 | 0.0337 | 3.018358703 |
| membrane-enclosed lumen | GO:0031974 | 1.71 | 0.0328 | 2.537855727 |
| intracellular organelle lumen | GO:0070013 | 1.7 | 0.0343 | 2.489999996 |
| organelle lumen | GO:0043233 | 1.66 | 0.0406 | 2.309846784 |
| protein binding | GO:0005515 | 1.32 | 0.0203 | 2.23410523 |
| cell part | GO:0044464 | 1.06 | 0.0337 | 1.560712305 |
| cell | GO:0005623 | 1.06 | 0.0337 | 1.560712305 |
