# Supplementary material for: VENNTURE–A Novel Venn Diagram Investigational Tool for Multiple Pharmacological Dataset Analysis
Source: PLoS One. 2012 May 14;7(5):e36911. doi: 10.1371/journal.pone.0036911 (PMC3351456; doi:10.1371/journal.pone.0036911)
Supplement: Table S21 — GO term groups populated by extracted phosphoproteins in 10 µM MeCh-stimulated control-state SH-SY5Y cells. GO term groups were considered enriched only if at least two proteins were present in each group and with a probability of ≤0.05. Hybrid GO term group scores were generated by multiplication of the GO term group enrichment score with the negative log10 of the probability result. (DOC) [file pone.0036911.s022.doc]

**Table S21**. GO term groups populated by extracted phosphoproteins in 10µM MeCh-stimulated control-state SH-SY5Y cells. GO term groups were considered enriched only if at least two proteins were present in each group and with a probability of ≤0.05. Hybrid GO term group scores were generated by multiplication of the GO term group enrichment score with the negative log_10_ of the probability result.

| **GO term** | **GO term ID** | **Enrichment** | **Probability** | **Hybrid** |
| --- | --- | --- | --- | --- |
| ATP-dependent polydeoxyribonucleotide 5'-hydroxyl-kinase activity | GO:0046404 | 63.23 | 0.0057 | 141.8960329 |
| ATP-dependent polynucleotide kinase activity | GO:0051734 | 63.23 | 0.0057 | 141.8960329 |
| polydeoxyribonucleotide kinase activity | GO:0051733 | 63.23 | 0.0057 | 141.8960329 |
| polynucleotide kinase activity | GO:0051731 | 63.23 | 0.0057 | 141.8960329 |
| telomerase activity | GO:0003720 | 47.42 | 0.01 | 94.84 |
| CRD-mediated mRNA stability complex | GO:0070937 | 38.86 | 0.0061 | 86.06208261 |
| telomerase holoenzyme complex | GO:0005697 | 32.38 | 0.0077 | 68.43543032 |
| CRD-mediated mRNA stabilization | GO:0070934 | 39.37 | 0.0371 | 56.32374918 |
| telomeric DNA binding | GO:0042162 | 18.97 | 0.0078 | 39.98696539 |
| regulation of centrosome cycle | GO:0046605 | 28.12 | 0.0427 | 38.51236815 |
| actin monomer binding | GO:0003785 | 23.71 | 0.0323 | 35.3468082 |
| RNA-directed DNA polymerase activity | GO:0003964 | 23.71 | 0.0323 | 35.3468082 |
| phosphoprotein binding | GO:0051219 | 14.05 | 0.0057 | 31.52995828 |
| actinin binding | GO:0042805 | 18.97 | 0.0425 | 26.019462 |
| telomere maintenance | GO:0000723 | 12.7 | 0.0126 | 24.12529408 |
| telomere organization | GO:0032200 | 12.3 | 0.0126 | 23.3654423 |
| nucleus | GO:0005634 | 1.93 | 1.69E-12 | 22.72017866 |
| nucleotide-excision repair, DNA damage removal | GO:0000718 | 14.06 | 0.0379 | 19.98433271 |
| regulation of mRNA stability | GO:0043488 | 13.42 | 0.0379 | 19.0746618 |
| chromosome | GO:0005694 | 3.85 | 1.39E-05 | 18.69939302 |
| growth cone | GO:0030426 | 8.1 | 0.0077 | 17.11942513 |
| regulation of RNA stability | GO:0043487 | 12.3 | 0.0421 | 16.92133022 |
| intracellular non-membrane-bounded organelle | GO:0043232 | 2.21 | 2.57E-08 | 16.7740478 |
| non-membrane-bounded organelle | GO:0043228 | 2.21 | 2.57E-08 | 16.7740478 |
| nuclear part | GO:0044428 | 2.45 | 1.49E-07 | 16.72569364 |
| site of polarized growth | GO:0030427 | 7.93 | 0.008 | 16.6284964 |
| RNA binding | GO:0003723 | 3.32 | 1.06E-05 | 16.51598453 |
| chromosome condensation | GO:0030261 | 11.81 | 0.0427 | 16.1746468 |
| I band | GO:0031674 | 7.62 | 0.0089 | 15.62564815 |
| damaged DNA binding | GO:0003684 | 8.62 | 0.0159 | 15.50395679 |
| RNA splicing | GO:0008380 | 4.72 | 0.001 | 14.16 |
| negative regulation of organelle organization | GO:0010639 | 7.03 | 0.0097 | 14.15299471 |
| actin filament binding | GO:0051015 | 7.9 | 0.0197 | 13.47371681 |
| macromolecular complex | GO:0032991 | 1.98 | 2.81E-07 | 12.97156149 |
| chromosomal part | GO:0044427 | 3.6 | 0.0003 | 12.68236348 |
| protein C-terminus binding | GO:0008022 | 5.15 | 0.0069 | 11.12992718 |
| intracellular organelle | GO:0043229 | 1.42 | 1.92E-08 | 10.95771226 |
| organelle | GO:0043226 | 1.41 | 1.92E-08 | 10.88054527 |
| intracellular part | GO:0044424 | 1.32 | 1.92E-08 | 10.18604238 |
| nuclear mRNA splicing, via spliceosome | GO:0000398 | 4.92 | 0.0097 | 9.905083067 |
| RNA splicing, via transesterification reactions with bulged adenosine as nucleophile | GO:0000377 | 4.92 | 0.0097 | 9.905083067 |
| RNA splicing, via transesterification reactions | GO:0000375 | 4.92 | 0.0097 | 9.905083067 |
| negative regulation of cytoskeleton organization | GO:0051494 | 7.16 | 0.0454 | 9.615480094 |
| mRNA processing | GO:0006397 | 3.87 | 0.0035 | 9.504456668 |
| organelle part | GO:0044422 | 1.71 | 3.94E-06 | 9.241701461 |
| microtubule associated complex | GO:0005875 | 5.01 | 0.0146 | 9.196592193 |
| intracellular | GO:0005622 | 1.29 | 1.00E-07 | 9.03 |
| mRNA metabolic process | GO:0016071 | 3.63 | 0.0035 | 8.915032999 |
| intracellular organelle part | GO:0044446 | 1.69 | 6.37E-06 | 8.781004359 |
| protein complex | GO:0043234 | 1.91 | 3.16E-05 | 8.595597672 |
| RNA processing | GO:0006396 | 3.19 | 0.0022 | 8.477671648 |
| cytosol | GO:0005829 | 2.25 | 0.0003 | 7.926477177 |
| nucleic acid binding | GO:0003676 | 1.79 | 3.74E-05 | 7.924559832 |
| cytoskeletal part | GO:0044430 | 2.41 | 0.0006 | 7.764655487 |
| regulation of cytoskeleton organization | GO:0051493 | 5 | 0.0379 | 7.10680395 |
| nuclear lumen | GO:0031981 | 2.11 | 0.0005 | 6.965173291 |
| regulation of Rho protein signal transduction | GO:0035023 | 5.24 | 0.0473 | 6.943727623 |
| protein binding | GO:0005515 | 1.39 | 1.06E-05 | 6.914824847 |
| contractile fiber | GO:0043292 | 4.3 | 0.0264 | 6.787103114 |
| microtubule | GO:0005874 | 3.27 | 0.0087 | 6.737772044 |
| cytoskeleton | GO:0005856 | 2.08 | 0.0007 | 6.562196077 |
| ribonucleoprotein complex | GO:0030529 | 2.74 | 0.0041 | 6.540972233 |
| Rho protein signal transduction | GO:0007266 | 4.58 | 0.0427 | 6.272640332 |
| intracellular membrane-bounded organelle | GO:0043231 | 1.36 | 3.16E-05 | 6.120425568 |
| membrane-bounded organelle | GO:0043227 | 1.36 | 3.16E-05 | 6.120425568 |
| nuclear chromosome | GO:0000228 | 3.74 | 0.0241 | 6.051256261 |
| anchoring junction | GO:0070161 | 3.55 | 0.0285 | 5.485300747 |
| microtubule cytoskeleton | GO:0015630 | 2.54 | 0.0074 | 5.412151432 |
| nuclear body | GO:0016604 | 3.51 | 0.0298 | 5.355500913 |
| guanyl-nucleotide exchange factor activity | GO:0005085 | 3.82 | 0.043 | 5.2201505 |
| binding | GO:0005488 | 1.16 | 3.74E-05 | 5.135468941 |
| striated muscle tissue development | GO:0014706 | 3.76 | 0.0473 | 4.982522111 |
| regulation of small GTPase mediated signal transduction | GO:0051056 | 3.49 | 0.0379 | 4.960549157 |
| gene expression | GO:0010467 | 1.66 | 0.0012 | 4.848559132 |
| nucleoplasm | GO:0005654 | 2.15 | 0.0077 | 4.544044941 |
| nucleolus | GO:0005730 | 2.24 | 0.01 | 4.48 |
| cellular macromolecule metabolic process | GO:0044260 | 1.46 | 0.0012 | 4.264395381 |
| response to DNA damage stimulus | GO:0006974 | 2.95 | 0.0379 | 4.193014331 |
| cellular response to DNA damage stimulus | GO:0034984 | 2.96 | 0.0427 | 4.05393349 |
| intracellular organelle lumen | GO:0070013 | 1.78 | 0.0057 | 3.994542757 |
| membrane-enclosed lumen | GO:0031974 | 1.75 | 0.0057 | 3.927219003 |
| chromosome organization | GO:0051276 | 2.61 | 0.0379 | 3.709751662 |
| organelle lumen | GO:0043233 | 1.73 | 0.0074 | 3.686229125 |
| cell part | GO:0044464 | 1.08 | 0.0005 | 3.565112395 |
| cell | GO:0005623 | 1.08 | 0.0005 | 3.565112395 |
| GTPase regulator activity | GO:0030695 | 2.56 | 0.0405 | 3.564915141 |
| DNA metabolic process | GO:0006259 | 2.5 | 0.0379 | 3.553401975 |
| nucleoside-triphosphatase regulator activity | GO:0060589 | 2.5 | 0.0425 | 3.429027675 |
| cellular response to stress | GO:0033554 | 2.46 | 0.0413 | 3.404762873 |
| macromolecule metabolic process | GO:0043170 | 1.37 | 0.0041 | 3.270486116 |
| nucleobase, nucleoside, nucleotide and nucleic acid metabolic process | GO:0006139 | 1.52 | 0.0079 | 3.195606821 |
| RNA metabolic process | GO:0016070 | 1.62 | 0.0316 | 2.430506926 |
| organelle organization | GO:0006996 | 1.79 | 0.0495 | 2.336656694 |
| cellular process | GO:0009987 | 1.11 | 0.0097 | 2.234683375 |
| cellular component organization | GO:0016043 | 1.53 | 0.0473 | 2.027462455 |
| nitrogen compound metabolic process | GO:0006807 | 1.39 | 0.0379 | 1.975691498 |
| regulation of macromolecule metabolic process | GO:0060255 | 1.47 | 0.0473 | 1.947954123 |
| regulation of cellular metabolic process | GO:0031323 | 1.44 | 0.0498 | 1.875989746 |
