# Supplementary material for: VENNTURE–A Novel Venn Diagram Investigational Tool for Multiple Pharmacological Dataset Analysis
Source: PLoS One. 2012 May 14;7(5):e36911. doi: 10.1371/journal.pone.0036911 (PMC3351456; doi:10.1371/journal.pone.0036911)
Supplement: Table S22 — GO term groups populated by extracted phosphoproteins in 100 µM MeCh-stimulated control-state SH-SY5Y cells. GO term groups were considered enriched only if at least two proteins were present in each group and with a probability of ≤0.05. Hybrid GO term group scores were generated by multiplication of the GO term group enrichment score with the negative log10 of the probability result. (DOC) [file pone.0036911.s023.doc]

**Table S22**. GO term groups populated by extracted phosphoproteins in 100µM MeCh-stimulated control-state SH-SY5Y cells. GO term groups were considered enriched only if at least two proteins were present in each group and with a probability of ≤0.05. Hybrid GO term group scores were generated by multiplication of the GO term group enrichment score with the negative log_10_ of the probability result.

| **GO term** | **GO term ID** | **Enrichment** | **Probability** | **Hybrid** |
| --- | --- | --- | --- | --- |
| heterogeneous nuclear ribonucleoprotein complex | GO:0030530 | 17.73 | 0.037 | 25.38578343 |
| stress fiber | GO:0001725 | 13.7 | 0.0468 | 18.21763181 |
| actin filament bundle | GO:0032432 | 12.56 | 0.0497 | 16.37376376 |
| actomyosin | GO:0042641 | 12.06 | 0.0497 | 15.72194195 |
| RNA binding | GO:0003723 | 3.55 | 0.0004 | 12.06268703 |
| proteasome complex | GO:0000502 | 7.54 | 0.0433 | 10.28088126 |
| establishment of RNA localization | GO:0051236 | 7.11 | 0.0409 | 9.87064728 |
| nucleic acid transport | GO:0050657 | 7.11 | 0.0409 | 9.87064728 |
| RNA transport | GO:0050658 | 7.11 | 0.0409 | 9.87064728 |
| RNA splicing | GO:0008380 | 4.87 | 0.0097 | 9.804421654 |
| RNA localization | GO:0006403 | 6.91 | 0.0409 | 9.592991942 |
| mRNA processing | GO:0006397 | 4.73 | 0.0097 | 9.522569697 |
| nuclear speck | GO:0016607 | 5.85 | 0.037 | 8.376019914 |
| nuclear pore | GO:0005643 | 6.37 | 0.0497 | 8.304209804 |
| mRNA metabolic process | GO:0016071 | 4.12 | 0.012 | 7.913773266 |
| nuclear mRNA splicing, via spliceosome | GO:0000398 | 5.34 | 0.0409 | 7.413397535 |
| RNA splicing, via transesterification reactions with bulged adenosine as nucleophile | GO:0000377 | 5.34 | 0.0409 | 7.413397535 |
| RNA splicing, via transesterification reactions | GO:0000375 | 5.34 | 0.0409 | 7.413397535 |
| nuclear part | GO:0044428 | 2.36 | 0.0012 | 6.893132259 |
| chromosome, centromeric region | GO:0000775 | 5.02 | 0.0447 | 6.775456234 |
| chromosomal part | GO:0044427 | 3.59 | 0.0132 | 6.747139587 |
| RNA processing | GO:0006396 | 3.33 | 0.0125 | 6.337289657 |
| spliceosomal complex | GO:0005681 | 4.53 | 0.0497 | 5.905505559 |
| protein binding | GO:0005515 | 1.41 | 0.0004 | 4.791095412 |
| chromosome | GO:0005694 | 2.99 | 0.0312 | 4.502477764 |
| ribonucleoprotein complex | GO:0030529 | 2.73 | 0.037 | 3.908809293 |
| nucleus | GO:0005634 | 1.58 | 0.0035 | 3.88037249 |
| macromolecular complex | GO:0032991 | 1.78 | 0.0078 | 3.752071607 |
| intracellular non-membrane-bounded organelle | GO:0043232 | 1.78 | 0.0132 | 3.345378402 |
| non-membrane-bounded organelle | GO:0043228 | 1.78 | 0.0132 | 3.345378402 |
| intracellular organelle part | GO:0044446 | 1.61 | 0.0088 | 3.309382898 |
| organelle part | GO:0044422 | 1.6 | 0.0094 | 3.242995434 |
| nuclear lumen | GO:0031981 | 2.01 | 0.0298 | 3.066825309 |
| nucleoplasm | GO:0005654 | 2.1 | 0.0497 | 2.737651584 |
| membrane-enclosed lumen | GO:0031974 | 1.81 | 0.0351 | 2.632994119 |
| cytosol | GO:0005829 | 1.93 | 0.0447 | 2.60490648 |
| protein complex | GO:0043234 | 1.7 | 0.0298 | 2.593832351 |
| intracellular organelle lumen | GO:0070013 | 1.81 | 0.037 | 2.591554879 |
| organelle lumen | GO:0043233 | 1.77 | 0.0406 | 2.462908921 |
| intracellular | GO:0005622 | 1.18 | 0.0298 | 1.800424808 |
| intracellular part | GO:0044424 | 1.18 | 0.037 | 1.689521966 |
