# Supplementary material for: VENNTURE–A Novel Venn Diagram Investigational Tool for Multiple Pharmacological Dataset Analysis
Source: PLoS One. 2012 May 14;7(5):e36911. doi: 10.1371/journal.pone.0036911 (PMC3351456; doi:10.1371/journal.pone.0036911)
Supplement: Table S23 — GO term groups populated by extracted phosphoproteins in non-stimulated CMP-state SH-SY5Y cells. GO term groups were considered enriched only if at least two proteins were present in each group and with a probability of ≤0.05. Hybrid GO term group scores were generated by multiplication of the GO term group enrichment score with the negative log10 of the probability result. (DOC) [file pone.0036911.s024.doc]

**Table S23.** GO term groups populated by extracted phosphoproteins in non-stimulated CMP-state SH-SY5Y cells. GO term groups were considered enriched only if at least two proteins were present in each group and with a probability of ≤0.05. Hybrid GO term group scores were generated by multiplication of the GO term group enrichment score with the negative log_10_ of the probability result.

| **GO term** | **GO term ID** | **Enrichment** | **Probability** | **Hybrid** |
| --- | --- | --- | --- | --- |
| negative regulation of microtubule depolymerization | GO:0007026 | 16.91 | 3.54E-05 | 75.26635484 |
| regulation of microtubule depolymerization | GO:0031114 | 16.91 | 3.54E-05 | 75.26635484 |
| microtubule depolymerization | GO:0007019 | 15.57 | 1.64E-05 | 74.50488129 |
| negative regulation of microtubule polymerization or depolymerization | GO:0031111 | 15.85 | 5.02E-05 | 68.14384608 |
| negative regulation of protein complex disassembly | GO:0043242 | 10.57 | 5.20E-06 | 55.85184466 |
| protein depolymerization | GO:0051261 | 10.11 | 3.38E-06 | 55.31265216 |
| heterogeneous nuclear ribonucleoprotein complex | GO:0030530 | 13 | 0.0002 | 48.08661006 |
| regulation of microtubule cytoskeleton organization | GO:0070507 | 10.57 | 3.26E-05 | 47.42528997 |
| nuclear part | GO:0044428 | 2.55 | 1.20E-17 | 43.14808782 |
| regulation of microtubule polymerization or depolymerization | GO:0031110 | 11.53 | 0.0003 | 40.61879193 |
| regulation of protein complex disassembly | GO:0043244 | 8.29 | 2.14E-05 | 38.71086982 |
| intracellular non-membrane-bounded organelle | GO:0043232 | 2.2 | 1.20E-17 | 37.22580126 |
| non-membrane-bounded organelle | GO:0043228 | 2.2 | 1.20E-17 | 37.22580126 |
| microtubule polymerization or depolymerization | GO:0031109 | 9.86 | 0.0002 | 36.47184424 |
| regulation of microtubule-based process | GO:0032886 | 8.9 | 9.50E-05 | 35.79825991 |
| DNA topoisomerase (ATP-hydrolyzing) activity | GO:0003918 | 21.1 | 0.0219 | 35.01662918 |
| histone acetyl-lysine binding | GO:0070577 | 21.1 | 0.0219 | 35.01662918 |
| telomerase activity | GO:0003720 | 21.1 | 0.0219 | 35.01662918 |
| negative regulation of cytoskeleton organization | GO:0051494 | 7.68 | 3.26E-05 | 34.45848883 |
| cellular protein complex disassembly | GO:0043624 | 7.26 | 2.14E-05 | 33.90119601 |
| protein complex disassembly | GO:0043241 | 7.04 | 2.57E-05 | 32.31407081 |
| regulation of cytoskeleton organization | GO:0051493 | 5.73 | 5.20E-06 | 30.27730084 |
| nuclear lumen | GO:0031981 | 2.42 | 3.13E-12 | 27.8407827 |
| cellular macromolecular complex disassembly | GO:0034623 | 6.37 | 5.02E-05 | 27.38651732 |
| telomeric DNA binding | GO:0042162 | 11.26 | 0.0037 | 27.38204859 |
| RNA binding | GO:0003723 | 2.95 | 6.93E-10 | 27.01983696 |
| macromolecular complex disassembly | GO:0032984 | 6.2 | 5.83E-05 | 26.25285496 |
| nucleus | GO:0005634 | 1.66 | 9.12E-15 | 23.30640857 |
| nucleolus | GO:0005730 | 2.8 | 8.61E-09 | 22.58199118 |
| spliceosomal complex | GO:0005681 | 4.65 | 1.65E-05 | 22.23869966 |
| spindle microtubule | GO:0005876 | 7.89 | 0.0021 | 21.12768976 |
| cytoskeleton | GO:0005856 | 2.29 | 7.14E-10 | 20.9450311 |
| negative regulation of organelle organization | GO:0010639 | 5.53 | 0.0002 | 20.45530412 |
| microtubule associated complex | GO:0005875 | 5.01 | 8.94E-05 | 20.28379903 |
| intracellular organelle part | GO:0044446 | 1.7 | 1.50E-12 | 20.10064486 |
| organelle part | GO:0044422 | 1.69 | 1.99E-12 | 19.7749383 |
| cytosol | GO:0005829 | 2.3 | 3.20E-09 | 19.53815505 |
| microtubule cytoskeleton | GO:0015630 | 2.89 | 2.14E-07 | 19.2751042 |
| nuclear speck | GO:0016607 | 4.72 | 0.0001 | 18.88 |
| establishment or maintenance of cell polarity | GO:0007163 | 6.72 | 0.0018 | 18.44456877 |
| microtubule | GO:0005874 | 3.48 | 7.57E-06 | 17.82074634 |
| nuclear body | GO:0016604 | 3.99 | 4.37E-05 | 17.39447907 |
| intracellular organelle lumen | GO:0070013 | 2.04 | 3.42E-09 | 17.27058674 |
| intracellular | GO:0005622 | 1.27 | 2.60E-14 | 17.25298385 |
| membrane-enclosed lumen | GO:0031974 | 2.01 | 4.50E-09 | 16.77704285 |
| regulation of organelle organization | GO:0033043 | 3.82 | 5.23E-05 | 16.35532355 |
| mRNA processing | GO:0006397 | 3.32 | 1.20E-05 | 16.33711826 |
| organelle lumen | GO:0043233 | 2 | 8.61E-09 | 16.1299937 |
| nucleoplasm | GO:0005654 | 2.41 | 2.14E-07 | 16.07370281 |
| protein binding | GO:0005515 | 1.37 | 4.66E-12 | 15.52431129 |
| cytoskeletal part | GO:0044430 | 2.34 | 2.78E-07 | 15.34093518 |
| microtubule cytoskeleton organization | GO:0000226 | 4.09 | 0.0002 | 15.12878732 |
| RNA splicing | GO:0008380 | 3.33 | 3.03E-05 | 15.04679605 |
| intracellular part | GO:0044424 | 1.27 | 1.55E-12 | 14.99827874 |
| chromatin remodeling complex | GO:0016585 | 5.05 | 0.0012 | 14.75013471 |
| cellular component disassembly | GO:0022411 | 4.65 | 0.0007 | 14.67029411 |
| nuclear mRNA splicing, via spliceosome | GO:0000398 | 3.96 | 0.0002 | 14.64792122 |
| RNA splicing, via transesterification reactions with bulged adenosine as nucleophile | GO:0000377 | 3.96 | 0.0002 | 14.64792122 |
| RNA splicing, via transesterification reactions | GO:0000375 | 3.96 | 0.0002 | 14.64792122 |
| cyclin-dependent protein kinase activity | GO:0004693 | 6.81 | 0.0076 | 14.43165944 |
| cytoskeleton organization | GO:0007010 | 2.88 | 1.20E-05 | 14.17195801 |
| cell cortex | GO:0005938 | 3.96 | 0.0003 | 13.95059983 |
| RNA processing | GO:0006396 | 2.74 | 8.16E-06 | 13.94196897 |
| mRNA metabolic process | GO:0016071 | 3 | 2.57E-05 | 13.77020063 |
| organelle organization | GO:0006996 | 2.15 | 4.05E-07 | 13.7439717 |
| nucleotide binding | GO:0000166 | 1.85 | 4.85E-08 | 13.53137778 |
| intracellular organelle | GO:0043229 | 1.31 | 9.96E-11 | 13.10228027 |
| organelle | GO:0043226 | 1.31 | 1.12E-10 | 13.03552439 |
| chromosome | GO:0005694 | 2.63 | 4.05E-05 | 11.55239329 |
| macromolecular complex | GO:0032991 | 1.64 | 1.69E-07 | 11.1062658 |
| actin filament binding | GO:0051015 | 5.28 | 0.009 | 10.80159955 |
| microtubule-based process | GO:0007017 | 3.11 | 0.0004 | 10.56759343 |
| cytoskeletal protein binding | GO:0008092 | 2.56 | 7.90E-05 | 10.50207465 |
| helicase activity | GO:0004386 | 3.67 | 0.0014 | 10.47371011 |
| calmodulin binding | GO:0005516 | 3.62 | 0.0014 | 10.33101651 |
| nucleoplasm part | GO:0044451 | 2.39 | 0.0001 | 9.56 |
| protein C-terminus binding | GO:0008022 | 3.6 | 0.0027 | 9.247090449 |
| nuclear envelope | GO:0005635 | 3.16 | 0.0012 | 9.229787262 |
| microtubule binding | GO:0008017 | 4.48 | 0.009 | 9.164993558 |
| nucleic acid binding | GO:0003676 | 1.58 | 1.59E-06 | 9.161792544 |
| chromosomal part | GO:0044427 | 2.57 | 0.0003 | 9.053798375 |
| ATP-dependent helicase activity | GO:0008026 | 3.96 | 0.0056 | 8.917175413 |
| purine NTP-dependent helicase activity | GO:0070035 | 3.96 | 0.0056 | 8.917175413 |
| enzyme binding | GO:0019899 | 2.42 | 0.0003 | 8.525366564 |
| ATP binding | GO:0005524 | 1.85 | 3.73E-05 | 8.192338661 |
| negative regulation of cellular component organization | GO:0051129 | 3.47 | 0.0048 | 8.046092906 |
| adenyl ribonucleotide binding | GO:0032559 | 1.83 | 5.00E-05 | 7.870884892 |
| cellular component organization | GO:0016043 | 1.63 | 1.69E-05 | 7.778544671 |
| histone binding | GO:0042393 | 4.8 | 0.024 | 7.77498604 |
| protein complex | GO:0043234 | 1.6 | 2.00E-05 | 7.518352007 |
| actin binding | GO:0003779 | 2.62 | 0.0014 | 7.477144547 |
| structural constituent of cytoskeleton | GO:0005200 | 3.99 | 0.0161 | 7.154764755 |
| binding | GO:0005488 | 1.13 | 4.91E-07 | 7.129077914 |
| tubulin binding | GO:0015631 | 3.63 | 0.0148 | 6.641949973 |
| intracellular membrane-bounded organelle | GO:0043231 | 1.26 | 5.43E-06 | 6.634152215 |
| membrane-bounded organelle | GO:0043227 | 1.26 | 5.58E-06 | 6.619240909 |
| ribonucleoprotein complex | GO:0030529 | 2.23 | 0.0012 | 6.513425821 |
| cellular macromolecule metabolic process | GO:0044260 | 1.34 | 1.56E-05 | 6.441213038 |
| adenyl nucleotide binding | GO:0030554 | 1.74 | 0.0002 | 6.436207808 |
| regulation of cellular component organization | GO:0051128 | 2.33 | 0.0025 | 6.06279978 |
| purine nucleoside binding | GO:0001883 | 1.71 | 0.0003 | 6.024122654 |
| nucleoside binding | GO:0001882 | 1.7 | 0.0003 | 5.988893867 |
| ATPase activity | GO:0016887 | 2.45 | 0.0037 | 5.957905776 |
| chromosome organization | GO:0051276 | 2.24 | 0.0025 | 5.828614381 |
| purine ribonucleotide binding | GO:0032555 | 1.65 | 0.0003 | 5.81274993 |
| ribonucleotide binding | GO:0032553 | 1.65 | 0.0003 | 5.81274993 |
| gene expression | GO:0010467 | 1.44 | 9.54E-05 | 5.78945034 |
| cell part | GO:0044464 | 1.06 | 1.63E-05 | 5.075081139 |
| cell | GO:0005623 | 1.06 | 1.63E-05 | 5.075081139 |
| purine nucleotide binding | GO:0017076 | 1.58 | 0.0009 | 4.812296835 |
| macromolecule metabolic process | GO:0043170 | 1.26 | 0.0004 | 4.281404411 |
| GTPase activator activity | GO:0005096 | 2.44 | 0.0207 | 4.109032357 |
| nucleobase, nucleoside, nucleotide and nucleic acid metabolic process | GO:0006139 | 1.35 | 0.0013 | 3.896176474 |
| nucleoside-triphosphatase activity | GO:0017111 | 1.82 | 0.0096 | 3.672266356 |
| enzyme activator activity | GO:0008047 | 2.14 | 0.0206 | 3.608324148 |
| protein serine/threonine kinase activity | GO:0004674 | 1.98 | 0.0216 | 3.297781573 |
| protein kinase activity | GO:0004672 | 1.84 | 0.017 | 3.255973985 |
| pyrophosphatase activity | GO:0016462 | 1.75 | 0.0164 | 3.124023266 |
| hydrolase activity, acting on acid anhydrides, in phosphorus-containing anhydrides | GO:0016818 | 1.74 | 0.0167 | 3.09247334 |
| hydrolase activity, acting on acid anhydrides | GO:0016817 | 1.73 | 0.0173 | 3.048180242 |
