# Supplementary material for: VENNTURE–A Novel Venn Diagram Investigational Tool for Multiple Pharmacological Dataset Analysis
Source: PLoS One. 2012 May 14;7(5):e36911. doi: 10.1371/journal.pone.0036911 (PMC3351456; doi:10.1371/journal.pone.0036911)
Supplement: Table S24 — GO term groups populated by extracted phosphoproteins in 10 nM MeCh-stimulated CMP-state SH-SY5Y cells. GO term groups were considered enriched only if at least two proteins were present in each group and with a probability of ≤0.05. Hybrid GO term group scores were generated by multiplication of the GO term group enrichment score with the negative log10 of the probability result. (DOC) [file pone.0036911.s025.doc]

**Table S24**. GO term groups populated by extracted phosphoproteins in 10nM MeCh-stimulated CMP-state SH-SY5Y cells. GO term groups were considered enriched only if at least two proteins were present in each group and with a probability of ≤0.05. Hybrid GO term group scores were generated by multiplication of the GO term group enrichment score with the negative log_10_ of the probability result.

| **GO term** | **GO term ID** | **Enrichment** | **Probability** | **Hybrid** |
| --- | --- | --- | --- | --- |
| DNA ligase (ATP) activity | GO:0003910 | 165.2 | 0.0019 | 449.5499051 |
| DNA ligase activity | GO:0003909 | 165.2 | 0.0019 | 449.5499051 |
| ligase activity, forming phosphoric ester bonds | GO:0016886 | 99.12 | 0.0054 | 224.7651305 |
| heterochromatin | GO:0000792 | 26.68 | 0.0004 | 90.65703943 |
| nuclear heterochromatin | GO:0005720 | 27.87 | 0.0026 | 72.04469279 |
| nuclear inner membrane | GO:0005637 | 23.65 | 0.0139 | 43.91769997 |
| chromatin | GO:0000785 | 9.15 | 0.0004 | 31.09115108 |
| nuclear chromatin | GO:0000790 | 14.45 | 0.0101 | 28.83755615 |
| lamellipodium | GO:0030027 | 11.48 | 0.0127 | 21.76833328 |
| chromosomal part | GO:0044427 | 6.19 | 0.0004 | 21.03324865 |
| chromatin binding | GO:0003682 | 8.66 | 0.0061 | 19.17904363 |
| chromosome | GO:0005694 | 5.16 | 0.0009 | 15.71610865 |
| nuclear chromosome | GO:0000228 | 6.67 | 0.0138 | 12.40700649 |
| nuclear chromosome part | GO:0044454 | 6.67 | 0.0402 | 9.309812226 |
| chromosome, centromeric region | GO:0000775 | 6.5 | 0.0409 | 9.023798498 |
| nucleus | GO:0005634 | 1.8 | 0.0007 | 5.678823528 |
| nuclear part | GO:0044428 | 2.18 | 0.0127 | 4.133707888 |
| intracellular non-membrane-bounded organelle | GO:0043232 | 1.94 | 0.0127 | 3.678620781 |
| non-membrane-bounded organelle | GO:0043228 | 1.94 | 0.0127 | 3.678620781 |
| intracellular organelle | GO:0043229 | 1.37 | 0.0068 | 2.96946279 |
| organelle | GO:0043226 | 1.37 | 0.0068 | 2.96946279 |
| intracellular membrane-bounded organelle | GO:0043231 | 1.38 | 0.0127 | 2.616750865 |
| membrane-bounded organelle | GO:0043227 | 1.38 | 0.0127 | 2.616750865 |
| intracellular | GO:0005622 | 1.26 | 0.0101 | 2.514555069 |
| nucleic acid binding | GO:0003676 | 1.76 | 0.0437 | 2.392752671 |
| intracellular part | GO:0044424 | 1.23 | 0.0283 | 1.904302684 |
