# Supplementary material for: VENNTURE–A Novel Venn Diagram Investigational Tool for Multiple Pharmacological Dataset Analysis
Source: PLoS One. 2012 May 14;7(5):e36911. doi: 10.1371/journal.pone.0036911 (PMC3351456; doi:10.1371/journal.pone.0036911)
Supplement: Table S25 — GO term groups populated by extracted phosphoproteins in 100 nM MeCh-stimulated CMP-state SH-SY5Y cells. GO term groups were considered enriched only if at least two proteins were present in each group and with a probability of ≤0.05. Hybrid GO term group scores were generated by multiplication of the GO term group enrichment score with the negative log10 of the probability result. (DOC) [file pone.0036911.s026.doc]

**Table S25.** GO term groups populated by extracted phosphoproteins in 100nM MeCh-stimulated CMP-state SH-SY5Y cells. GO term groups were considered enriched only if at least two proteins were present in each group and with a probability of ≤0.05. Hybrid GO term group scores were generated by multiplication of the GO term group enrichment score with the negative log_10_ of the probability result.

| **GO term** | **GO term ID** | **Enrichment** | **Probability** | **Hybrid** |
| --- | --- | --- | --- | --- |
| M band | GO:0031430 | 45.3 | 0.0051 | 103.847071 |
| MutLalpha complex binding | GO:0032405 | 57.54 | 0.0183 | 99.9785643 |
| mismatch repair complex binding | GO:0032404 | 43.16 | 0.0248 | 69.29546546 |
| nuclear chromatin | GO:0000790 | 16.78 | 0.0002 | 62.06871667 |
| DNA secondary structure binding | GO:0000217 | 38.36 | 0.0264 | 60.54727337 |
| heterochromatin | GO:0000792 | 18.58 | 0.0008 | 57.54058804 |
| A band | GO:0031672 | 25.89 | 0.0128 | 49.00433389 |
| sex chromatin | GO:0001739 | 25.89 | 0.0128 | 49.00433389 |
| sex chromosome | GO:0000803 | 24.16 | 0.013 | 45.56712861 |
| kinesin complex | GO:0005871 | 21.32 | 0.0162 | 38.17313989 |
| chromatin | GO:0000785 | 7.28 | 0.0002 | 26.92850163 |
| actin filament bundle | GO:0032432 | 15.1 | 0.0256 | 24.03557652 |
| nuclear chromosome | GO:0000228 | 6.97 | 0.0015 | 19.68264392 |
| nuclear heterochromatin | GO:0005720 | 12.94 | 0.0304 | 19.63161583 |
| nuclear chromosome part | GO:0044454 | 7.74 | 0.0036 | 18.91421864 |
| intracellular non-membrane-bounded organelle | GO:0043232 | 2.42 | 1.34E-05 | 11.79240639 |
| non-membrane-bounded organelle | GO:0043228 | 2.42 | 1.34E-05 | 11.79240639 |
| synaptic vesicle | GO:0008021 | 7.35 | 0.0256 | 11.69943625 |
| coated vesicle | GO:0030135 | 5.77 | 0.0097 | 11.61632709 |
| double-stranded DNA binding | GO:0003690 | 7.59 | 0.0348 | 11.06941354 |
| clathrin-coated vesicle | GO:0030136 | 5.58 | 0.0224 | 9.205616058 |
| structure-specific DNA binding | GO:0043566 | 6.51 | 0.0394 | 9.143319596 |
| chromosomal part | GO:0044427 | 3.83 | 0.0063 | 8.428525696 |
| chromosome | GO:0005694 | 3.59 | 0.0054 | 8.140706402 |
| actin cytoskeleton | GO:0015629 | 4.21 | 0.013 | 7.940298487 |
| microtubule associated complex | GO:0005875 | 5.6 | 0.0445 | 7.569183939 |
| nuclear part | GO:0044428 | 2.33 | 0.0008 | 7.21580033 |
| nucleus | GO:0005634 | 1.72 | 0.0002 | 6.362228407 |
| synapse part | GO:0044456 | 3.74 | 0.031 | 5.642307265 |
| neuron projection | GO:0043005 | 3.42 | 0.0256 | 5.443819319 |
| nuclear lumen | GO:0031981 | 2.29 | 0.0047 | 5.330895905 |
| intracellular organelle part | GO:0044446 | 1.71 | 0.0009 | 5.208245309 |
| intracellular part | GO:0044424 | 1.32 | 0.0002 | 4.882640406 |
| organelle part | GO:0044422 | 1.7 | 0.0015 | 4.80064486 |
| intracellular | GO:0005622 | 1.29 | 0.0002 | 4.771671306 |
| nucleolus | GO:0005730 | 2.61 | 0.0192 | 4.480583793 |
| protein binding | GO:0005515 | 1.44 | 0.0008 | 4.459550419 |
| intracellular organelle | GO:0043229 | 1.36 | 0.0008 | 4.211797618 |
| organelle | GO:0043226 | 1.36 | 0.0008 | 4.211797618 |
| cytoskeleton | GO:0005856 | 2.14 | 0.013 | 4.036161226 |
| nucleoside-triphosphatase activity | GO:0017111 | 2.64 | 0.0394 | 3.707889974 |
| pyrophosphatase activity | GO:0016462 | 2.54 | 0.0394 | 3.567439597 |
| hydrolase activity, acting on acid anhydrides, in phosphorus-containing anhydrides | GO:0016818 | 2.53 | 0.0394 | 3.553394559 |
| hydrolase activity, acting on acid anhydrides | GO:0016817 | 2.51 | 0.0394 | 3.525304483 |
| cytoskeletal part | GO:0044430 | 2.15 | 0.0357 | 3.111763335 |
| intracellular organelle lumen | GO:0070013 | 1.86 | 0.023 | 3.047186225 |
| organelle lumen | GO:0043233 | 1.82 | 0.0256 | 2.897003263 |
| membrane-enclosed lumen | GO:0031974 | 1.78 | 0.0304 | 2.700485021 |
| DNA binding | GO:0003677 | 1.73 | 0.044 | 2.34682687 |
| nucleic acid binding | GO:0003676 | 1.6 | 0.0394 | 2.247206045 |
| intracellular membrane-bounded organelle | GO:0043231 | 1.28 | 0.0227 | 2.104286903 |
| membrane-bounded organelle | GO:0043227 | 1.28 | 0.0227 | 2.104286903 |
| binding | GO:0005488 | 1.16 | 0.0165 | 2.067718625 |
