# Supplementary material for: VENNTURE–A Novel Venn Diagram Investigational Tool for Multiple Pharmacological Dataset Analysis
Source: PLoS One. 2012 May 14;7(5):e36911. doi: 10.1371/journal.pone.0036911 (PMC3351456; doi:10.1371/journal.pone.0036911)
Supplement: Table S26 — GO term groups populated by extracted phosphoproteins in 1 µM MeCh-stimulated CMP-state SH-SY5Y cells. GO term groups were considered enriched only if at least two proteins were present in each group and with a probability of ≤0.05. Hybrid GO term group scores were generated by multiplication of the GO term group enrichment score with the negative log10 of the probability result. (DOC) [file pone.0036911.s027.doc]

**Table S26.** GO term groups populated by extracted phosphoproteins in 1µM MeCh-stimulated CMP-state SH-SY5Y cells. GO term groups were considered enriched only if at least two proteins were present in each group and with a probability of ≤0.05. Hybrid GO term group scores were generated by multiplication of the GO term group enrichment score with the negative log_10_ of the probability result.

| **GO term** | **GO term ID** | **Enrichment** | **Probability** | **Hybrid** |
| --- | --- | --- | --- | --- |
| neurofilament cytoskeleton | GO:0060053 | 24.29 | 0.0243 | 39.21362361 |
| stress fiber | GO:0001725 | 13.25 | 0.0177 | 23.21435422 |
| filamentous actin | GO:0031941 | 16.19 | 0.0476 | 21.40954344 |
| actin filament bundle | GO:0032432 | 12.14 | 0.0192 | 20.84072308 |
| actomyosin | GO:0042641 | 11.66 | 0.0209 | 19.5870943 |
| actin filament | GO:0005884 | 7.88 | 0.0476 | 10.42045721 |
| chromosomal part | GO:0044427 | 3.34 | 0.0052 | 7.628548832 |
| nuclear pore | GO:0005643 | 5.47 | 0.0476 | 7.233489969 |
| chromatin | GO:0000785 | 3.91 | 0.0153 | 7.097856506 |
| chromosome | GO:0005694 | 3 | 0.0052 | 6.851989969 |
| nuclear part | GO:0044428 | 1.85 | 0.0052 | 4.225393814 |
| intracellular non-membrane-bounded organelle | GO:0043232 | 1.68 | 0.0052 | 3.837114383 |
| non-membrane-bounded organelle | GO:0043228 | 1.68 | 0.0052 | 3.837114383 |
| organelle organization | GO:0006996 | 2.2 | 0.0183 | 3.822607603 |
| intracellular organelle part | GO:0044446 | 1.48 | 0.0052 | 3.380315051 |
| organelle part | GO:0044422 | 1.47 | 0.0052 | 3.357475085 |
| nucleus | GO:0005634 | 1.44 | 0.0052 | 3.288955185 |
| macromolecular complex | GO:0032991 | 1.53 | 0.0153 | 2.777422111 |
| protein binding | GO:0005515 | 1.29 | 0.0094 | 2.614665069 |
| intracellular | GO:0005622 | 1.15 | 0.0192 | 1.974203587 |
| intracellular part | GO:0044424 | 1.16 | 0.0232 | 1.896033938 |
