# Supplementary material for: VENNTURE–A Novel Venn Diagram Investigational Tool for Multiple Pharmacological Dataset Analysis
Source: PLoS One. 2012 May 14;7(5):e36911. doi: 10.1371/journal.pone.0036911 (PMC3351456; doi:10.1371/journal.pone.0036911)
Supplement: Table S27 — GO term groups populated by extracted phosphoproteins in 10 µM MeCh-stimulated CMP-state SH-SY5Y cells. GO term groups were considered enriched only if at least two proteins were present in each group and with a probability of ≤0.05. Hybrid GO term group scores were generated by multiplication of the GO term group enrichment score with the negative log10 of the probability result. (DOC) [file pone.0036911.s028.doc]

**Table S27**. GO term groups populated by extracted phosphoproteins in 10µM MeCh-stimulated CMP-state SH-SY5Y cells. GO term groups were considered enriched only if at least two proteins were present in each group and with a probability of ≤0.05. Hybrid GO term group scores were generated by multiplication of the GO term group enrichment score with the negative log_10_ of the probability result.

| **GO term** | **GO term ID** | **Enrichment** | **Probability** | **Hybrid** |
| --- | --- | --- | --- | --- |
| nuclear chromatin | GO:0000790 | 9.24 | 0.0007 | 29.15129411 |
| mismatched DNA binding | GO:0030983 | 13.98 | 0.0215 | 23.31251033 |
| nuclear chromosome part | GO:0044454 | 6.39 | 0.0003 | 22.51119518 |
| exosome (RNase complex) | GO:0000178 | 13.85 | 0.0426 | 18.98267705 |
| heterochromatin | GO:0000792 | 8.53 | 0.0103 | 16.95049847 |
| nuclear chromosome | GO:0000228 | 4.8 | 0.001 | 14.4 |
| nuclear heterochromatin | GO:0005720 | 8.91 | 0.0246 | 14.3367682 |
| gene silencing by RNA | GO:0031047 | 9.79 | 0.0354 | 14.20525806 |
| chromosomal part | GO:0044427 | 3.52 | 0.0003 | 12.40053318 |
| chromatin | GO:0000785 | 4.18 | 0.0018 | 11.47296093 |
| nuclear pore | GO:0005643 | 5.85 | 0.0124 | 11.15348314 |
| gene silencing | GO:0016458 | 7.08 | 0.0354 | 10.2730569 |
| RNA binding | GO:0003723 | 2.77 | 0.0002 | 10.24614691 |
| structure-specific DNA binding | GO:0043566 | 5.23 | 0.0119 | 10.06488939 |
| chromosome | GO:0005694 | 3.11 | 0.0006 | 10.01994961 |
| nucleic acid binding | GO:0003676 | 1.81 | 5.96E-06 | 9.45680427 |
| nucleus | GO:0005634 | 1.62 | 2.50E-06 | 9.075337186 |
| double-stranded DNA binding | GO:0003690 | 5.22 | 0.0215 | 8.704671239 |
| nuclear part | GO:0044428 | 2.05 | 9.96E-05 | 8.203568356 |
| pore complex | GO:0046930 | 4.89 | 0.0223 | 8.07678922 |
| mRNA transport | GO:0051028 | 5.5 | 0.0375 | 7.842828027 |
| intracellular non-membrane-bounded organelle | GO:0043232 | 1.86 | 6.20E-05 | 7.826151458 |
| non-membrane-bounded organelle | GO:0043228 | 1.86 | 6.20E-05 | 7.826151458 |
| nuclear lumen | GO:0031981 | 1.92 | 0.0018 | 5.26987679 |
| M phase | GO:0000279 | 3.05 | 0.0188 | 5.26381856 |
| zinc ion binding | GO:0008270 | 1.75 | 0.0014 | 4.994275938 |
| cell cycle phase | GO:0022403 | 2.81 | 0.0188 | 4.849616444 |
| mRNA metabolic process | GO:0016071 | 2.97 | 0.0246 | 4.778922732 |
| actin cytoskeleton | GO:0015629 | 2.9 | 0.0244 | 4.676569504 |
| cell cycle process | GO:0022402 | 2.66 | 0.0188 | 4.590740121 |
| intracellular organelle part | GO:0044446 | 1.49 | 0.0009 | 4.538178661 |
| RNA processing | GO:0006396 | 2.61 | 0.0188 | 4.504448013 |
| intracellular | GO:0005622 | 1.21 | 0.0002 | 4.475753705 |
| organelle part | GO:0044422 | 1.48 | 0.001 | 4.44 |
| intracellular part | GO:0044424 | 1.21 | 0.0003 | 4.262683282 |
| binding | GO:0005488 | 1.14 | 0.0002 | 4.216825805 |
| mRNA processing | GO:0006397 | 2.93 | 0.0394 | 4.11519607 |
| intracellular organelle | GO:0043229 | 1.25 | 0.0008 | 3.871137516 |
| organelle | GO:0043226 | 1.25 | 0.0008 | 3.871137516 |
| DNA binding | GO:0003677 | 1.65 | 0.0072 | 3.535401381 |
| extrinsic to membrane | GO:0019898 | 2.21 | 0.0315 | 3.318733676 |
| cell cycle | GO:0007049 | 2.13 | 0.0292 | 3.268734526 |
| nucleoplasm | GO:0005654 | 1.93 | 0.0223 | 3.187771614 |
| intracellular organelle lumen | GO:0070013 | 1.66 | 0.0133 | 3.114406276 |
| membrane-enclosed lumen | GO:0031974 | 1.64 | 0.0134 | 3.071548131 |
| transition metal ion binding | GO:0046914 | 1.54 | 0.0128 | 2.914896647 |
| macromolecular complex | GO:0032991 | 1.48 | 0.0114 | 2.87578082 |
| organelle lumen | GO:0043233 | 1.62 | 0.0175 | 2.846278361 |
| gene expression | GO:0010467 | 1.47 | 0.0188 | 2.536987962 |
| nucleobase, nucleoside, nucleotide and nucleic acid metabolic process | GO:0006139 | 1.47 | 0.0188 | 2.536987962 |
| cytoskeleton | GO:0005856 | 1.66 | 0.0315 | 2.492804481 |
| cellular macromolecule metabolic process | GO:0044260 | 1.35 | 0.0188 | 2.329886903 |
| protein binding | GO:0005515 | 1.21 | 0.0215 | 2.017749464 |
| nitrogen compound metabolic process | GO:0006807 | 1.38 | 0.0354 | 2.002375498 |
| intracellular membrane-bounded organelle | GO:0043231 | 1.19 | 0.0246 | 1.914787223 |
| membrane-bounded organelle | GO:0043227 | 1.19 | 0.0246 | 1.914787223 |
| macromolecule metabolic process | GO:0043170 | 1.28 | 0.0354 | 1.857275825 |
