# Supplementary material for: VENNTURE–A Novel Venn Diagram Investigational Tool for Multiple Pharmacological Dataset Analysis
Source: PLoS One. 2012 May 14;7(5):e36911. doi: 10.1371/journal.pone.0036911 (PMC3351456; doi:10.1371/journal.pone.0036911)
Supplement: Table S28 — GO term groups populated by extracted phosphoproteins in 100 µM MeCh-stimulated CMP-state SH-SY5Y cells. GO term groups were considered enriched only if at least two proteins were present in each group and with a probability of ≤0.05. Hybrid GO term group scores were generated by multiplication of the GO term group enrichment score with the negative log10 of the probability result. (DOC) [file pone.0036911.s029.doc]

**Table S28.** GO term groups populated by extracted phosphoproteins in 100µM MeCh-stimulated CMP-state SH-SY5Y cells. GO term groups were considered enriched only if at least two proteins were present in each group and with a probability of ≤0.05. Hybrid GO term group scores were generated by multiplication of the GO term group enrichment score with the negative log_10_ of the probability result.

| **GO term** | **GO term ID** | **Enrichment** | **Probability** | **Hybrid** |
| --- | --- | --- | --- | --- |
| cell cycle process | GO:0022402 | 2.29 | 0.0292 | 3.51427327 |
| cell cycle | GO:0007049 | 2.04 | 0.0292 | 3.130618983 |
| cellular component assembly | GO:0022607 | 2 | 0.0292 | 3.069234297 |
| cellular component biogenesis | GO:0044085 | 1.93 | 0.0292 | 2.961811097 |
| cellular component organization | GO:0016043 | 1.63 | 0.0286 | 2.516123366 |
| cellular macromolecular complex assembly | GO:0034622 | 2.62 | 0.0393 | 3.682691518 |
| cellular macromolecular complex subunit organization | GO:0034621 | 2.61 | 0.0297 | 3.986105667 |
| chondrocyte differentiation | GO:0002062 | 15.45 | 0.0106 | 30.50902438 |
| chromatin modification | GO:0016568 | 3.33 | 0.0286 | 5.14030111 |
| chromatin organization | GO:0006325 | 2.64 | 0.0346 | 3.856839099 |
| chromatin remodeling | GO:0006338 | 6.66 | 0.0292 | 10.22055021 |
| chromosome organization | GO:0051276 | 2.52 | 0.0292 | 3.867235214 |
| establishment of RNA localization | GO:0051236 | 4.64 | 0.0367 | 6.659949462 |
| gene expression | GO:0010467 | 1.45 | 0.0286 | 2.238269252 |
| hindgut morphogenesis | GO:0007442 | 30.9 | 0.0346 | 45.14254855 |
| macromolecular complex assembly | GO:0065003 | 2.23 | 0.0292 | 3.422196241 |
| macromolecular complex subunit organization | GO:0043933 | 2.12 | 0.0292 | 3.253388355 |
| mRNA metabolic process | GO:0016071 | 2.85 | 0.0286 | 4.399356806 |
| mRNA processing | GO:0006397 | 3.03 | 0.0286 | 4.67721092 |
| mRNA transport | GO:0051028 | 5.27 | 0.0297 | 8.048573512 |
| negative regulation of chondrocyte differentiation | GO:0032331 | 30.9 | 0.0346 | 45.14254855 |
| nuclear export | GO:0051168 | 5.77 | 0.0357 | 8.351104393 |
| nuclear transport | GO:0051169 | 3.64 | 0.0292 | 5.586006421 |
| nucleic acid transport | GO:0050657 | 4.64 | 0.0367 | 6.659949462 |
| nucleobase, nucleoside, nucleotide and nucleic acid metabolic process | GO:0006139 | 1.39 | 0.0292 | 2.133117836 |
| nucleocytoplasmic transport | GO:0006913 | 3.66 | 0.0292 | 5.616698764 |
| organelle organization | GO:0006996 | 1.91 | 0.0286 | 2.948340877 |
| regulation of chondrocyte differentiation | GO:0032330 | 19.31 | 0.0286 | 29.8075719 |
| regulation of gene expression | GO:0010468 | 1.45 | 0.0393 | 2.038130802 |
| RNA localization | GO:0006403 | 4.5 | 0.0393 | 6.325233523 |
| RNA processing | GO:0006396 | 2.36 | 0.0292 | 3.621696471 |
| RNA splicing | GO:0008380 | 2.91 | 0.0346 | 4.251288553 |
| RNA transport | GO:0050658 | 4.64 | 0.0367 | 6.659949462 |
| smoothened signaling pathway involved in spinal cord motor neuron cell fate specification | GO:0021776 | 77.26 | 0.0286 | 119.2611603 |
| smoothened signaling pathway involved in ventral spinal cord interneuron specification | GO:0021775 | 77.26 | 0.0286 | 119.2611603 |
| smoothened signaling pathway involved in ventral spinal cord patterning | GO:0021910 | 30.9 | 0.0346 | 45.14254855 |
| spinal cord motor neuron cell fate specification | GO:0021520 | 30.9 | 0.0346 | 45.14254855 |
| ventral spinal cord interneuron differentiation | GO:0021514 | 30.9 | 0.0346 | 45.14254855 |
| ventral spinal cord interneuron fate commitment | GO:0060579 | 30.9 | 0.0346 | 45.14254855 |
| ventral spinal cord interneuron specification | GO:0021521 | 30.9 | 0.0346 | 45.14254855 |
| actin cytoskeleton | GO:0015629 | 2.87 | 0.0147 | 5.259799249 |
| cell cortex | GO:0005938 | 3.31 | 0.0436 | 4.503299721 |
| chromatin | GO:0000785 | 3.35 | 0.0097 | 6.74431469 |
| chromosomal part | GO:0044427 | 3.13 | 0.001 | 9.39 |
| chromosome | GO:0005694 | 2.77 | 0.0026 | 7.160523826 |
| condensed chromosome kinetochore | GO:0000777 | 5.1 | 0.0356 | 7.38760501 |
| condensed chromosome, centromeric region | GO:0000779 | 5.69 | 0.0101 | 11.35541138 |
| condensed chromosome | GO:0000793 | 4.7 | 0.0032 | 11.7257951 |
| euchromatin | GO:0000791 | 36.99 | 0.0074 | 78.81711869 |
| exosome (RNase complex) | GO:0000178 | 12.33 | 0.0477 | 16.29386839 |
| heterochromatin | GO:0000792 | 7.59 | 0.0101 | 15.14720077 |
| intracellular membrane-bounded organelle | GO:0043231 | 1.25 | 0.0026 | 3.231283315 |
| intracellular non-membrane-bounded organelle | GO:0043232 | 1.54 | 0.0059 | 3.432887902 |
| intracellular organelle lumen | GO:0070013 | 1.73 | 0.0032 | 4.316090538 |
| intracellular organelle part | GO:0044446 | 1.59 | 2.34E-05 | 7.362946787 |
| intracellular organelle | GO:0043229 | 1.26 | 0.0002 | 4.660702205 |
| intracellular part | GO:0044424 | 1.23 | 2.34E-05 | 5.695864495 |
| intracellular | GO:0005622 | 1.2 | 0.0002 | 4.438764005 |
| macromolecular complex | GO:0032991 | 1.41 | 0.0153 | 2.559585083 |
| membrane-bounded organelle | GO:0043227 | 1.25 | 0.0026 | 3.231283315 |
| membrane-enclosed lumen | GO:0031974 | 1.66 | 0.0061 | 3.676352474 |
| non-membrane-bounded organelle | GO:0043228 | 1.54 | 0.0059 | 3.432887902 |
| nuclear chromatin | GO:0000790 | 6.85 | 0.0061 | 15.17049063 |
| nuclear chromosome part | GO:0044454 | 4.43 | 0.0074 | 9.439303482 |
| nuclear chromosome | GO:0000228 | 3.32 | 0.0256 | 5.284643315 |
| nuclear envelope | GO:0005635 | 3.4 | 0.0088 | 6.988758915 |
| nuclear euchromatin | GO:0005719 | 49.32 | 0.0047 | 114.8121336 |
| nuclear heterochromatin | GO:0005720 | 7.93 | 0.0291 | 12.1813286 |
| nuclear inner membrane | GO:0005637 | 10.09 | 0.0159 | 18.14790302 |
| nuclear lumen | GO:0031981 | 1.97 | 0.0007 | 6.215156861 |
| nuclear matrix | GO:0016363 | 6.61 | 0.0066 | 14.41281459 |
| nuclear part | GO:0044428 | 2.19 | 1.88E-06 | 12.53959431 |
| nuclear periphery | GO:0034399 | 6.16 | 0.0085 | 12.75477942 |
| nucleoplasm | GO:0005654 | 2.15 | 0.0032 | 5.363927547 |
| nucleus | GO:0005634 | 1.6 | 9.06E-07 | 9.668594884 |
| organelle lumen | GO:0043233 | 1.69 | 0.0047 | 3.93415462 |
| organelle part | GO:0044422 | 1.58 | 2.50E-05 | 7.271254786 |
| organelle | GO:0043226 | 1.26 | 0.0002 | 4.660702205 |
| protein complex | GO:0043234 | 1.46 | 0.0179 | 2.550834575 |
| alkaline phosphatase activity | GO:0004035 | 35.73 | 0.0033 | 88.66349693 |
| binding | GO:0005488 | 1.09 | 0.0466 | 1.451459351 |
| DNA ligase (ATP) activity | GO:0003910 | 47.64 | 0.0143 | 87.87979118 |
| DNA ligase activity | GO:0003909 | 47.64 | 0.0143 | 87.87979118 |
| enzyme binding | GO:0019899 | 2.55 | 0.0095 | 5.156804807 |
| ligase activity, forming phosphoric ester bonds | GO:0016886 | 28.58 | 0.0361 | 41.22644417 |
| nucleic acid binding | GO:0003676 | 1.48 | 0.0095 | 2.992969064 |
| phosphatase binding | GO:0019902 | 9.74 | 0.003 | 24.57283898 |
| protein binding | GO:0005515 | 1.19 | 0.0361 | 1.71656643 |
| protein phosphatase binding | GO:0019903 | 9.16 | 0.0095 | 18.52405178 |
| RNA binding | GO:0003723 | 2.5 | 0.003 | 6.307196863 |
