# Supplementary material for: VENNTURE–A Novel Venn Diagram Investigational Tool for Multiple Pharmacological Dataset Analysis
Source: PLoS One. 2012 May 14;7(5):e36911. doi: 10.1371/journal.pone.0036911 (PMC3351456; doi:10.1371/journal.pone.0036911)
Supplement: Table S29 — Cumulated significantly populated Gene Ontology term groups generated from dose-dependent acetyl-β-methylcholine-stimulated phosphoproteins in control-state human neuroblastoma SH-SY5Y cells. Ligand stimulation with acetyl-β-methylcholine (MeCh: 10 nM–100 µM) was for 15 minutes before cell lysate protein extraction and titanium dioxide-mediated purification. (DOC) [file pone.0036911.s030.doc]

**Table S29.** Cumulated significantly populated Gene Ontology term groups generated from dose-dependent acetyl-β-methylcholine-stimulated phosphoproteins in control-state human neuroblastoma SH-SY5Y cells. Ligand stimulation with acetyl-β-methylcholine (MeCh: 10nM-100μM) was for 15 minutes before cell lysate protein extraction and titanium dioxide-mediated purification.

| **non-stiumulated** | **10nM MeCh** | **100nM MeCh** | **1μM MeCh** | **10μM MeCh** | **100μM MeCh** |
| --- | --- | --- | --- | --- | --- |
| *GO term ID* | *GO term ID* | *GO term ID* | *GO term ID* | *GO term ID* | *GO term ID* |
| GO:0022411 | GO:0005858 | GO:0016043 | GO:0008380 | GO:0008380 | GO:0008380 |
| GO:0016043 | GO:0044447 | GO:0044260 | GO:0006397 | GO:0010467 | GO:0006397 |
| GO:0034623 | GO:0022607 | GO:0034984 | GO:0000377 | GO:0044260 | GO:0016071 |
| GO:0044260 | GO:0044085 | GO:0051716 | GO:0006396 | GO:0006396 | GO:0006396 |
| GO:0043624 | GO:0016043 | GO:0033554 | GO:0016071 | GO:0006397 | GO:0051236 |
| GO:0006338 | GO:0034622 | GO:0070934 | GO:0000375 | GO:0016071 | GO:0000375 |
| GO:0051276 | GO:0034621 | GO:0008340 | GO:0000398 | GO:0043170 | GO:0000377 |
| GO:0007010 | GO:0044260 | GO:0010467 | GO:0006413 | GO:0006139 | GO:0050658 |
| GO:0010467 | GO:0044237 | GO:0043170 | GO:0016574 | GO:0000375 | GO:0000398 |
| GO:0032984 | GO:0034984 | GO:0051651 | GO:0005634 | GO:0009987 | GO:0006403 |
| GO:0043170 | GO:0006333 | GO:0051235 | GO:0032991 | GO:0010639 | GO:0050657 |
| GO:0007019 | GO:0044427 | GO:0007018 | GO:0044428 | GO:0000377 | GO:0044428 |
| GO:0031109 | GO:0005694 | GO:0007017 | GO:0044422 | GO:0000398 | GO:0005634 |
| GO:0016071 | GO:0051276 | GO:0010970 | GO:0043229 | GO:0032200 | GO:0032991 |
| GO:0006397 | GO:0000775 | GO:0016071 | GO:0005622 | GO:0000723 | GO:0044446 |
| GO:0051129 | GO:0000793 | GO:0006397 | GO:0043231 | GO:0016070 | GO:0044422 |
| GO:0051494 | GO:0005881 | GO:0051053 | GO:0044446 | GO:0070934 | GO:0044427 |
| GO:0007026 | GO:0044430 | GO:0010605 | GO:0043227 | GO:0006974 | GO:0043232 |
| GO:0031111 | GO:0005856 | GO:0009892 | GO:0043234 | GO:0051056 | GO:0043228 |
| GO:0051172 | GO:0005829 | GO:0006807 | GO:0043228 | GO:0051493 | GO:0005622 |
| GO:0045934 | GO:0006281 | GO:0000398 | GO:0043232 | GO:0051276 | GO:0043234 |
| GO:0010639 | GO:0030286 | GO:0006139 | GO:0043226 | GO:0000718 | GO:0031981 |
| GO:0043242 | GO:0040001 | GO:0031848 | GO:0044424 | GO:0006259 | GO:0005694 |
| GO:0006807 | GO:0000132 | GO:0070198 | GO:0005694 | GO:0006807 | GO:0031974 |
| GO:0000398 | GO:0051656 | GO:0034502 | GO:0030529 | GO:0043488 | GO:0030530 |
| GO:0006139 | GO:0051294 | GO:0080135 | GO:0005829 | GO:0033554 | GO:0016607 |
| GO:0006996 | GO:0010467 | GO:0010468 | GO:0044427 | GO:0043487 | GO:0044424 |
| GO:0043241 | GO:0030426 | GO:0060255 | GO:0031981 | GO:0046605 | GO:0070013 |
| GO:0051261 | GO:0030530 | GO:0019222 | GO:0000228 | GO:0034984 | GO:0030529 |
| GO:0022603 | GO:0005622 | GO:0080134 | GO:0000781 | GO:0030261 | GO:0043233 |
| GO:0022604 | GO:0043232 | GO:0006974 | GO:0044454 | GO:0007266 | GO:0000502 |
| GO:0031344 | GO:0043229 | GO:0006396 | GO:0016585 | GO:0051494 | GO:0000775 |
| GO:0008360 | GO:0070013 | GO:0000377 | GO:0031519 | GO:0016043 | GO:0005829 |
| GO:0051128 | GO:0044446 | GO:0000375 | GO:0000118 | GO:0060255 | GO:0001725 |
| GO:0031114 | GO:0044424 | GO:0008380 | GO:0005654 | GO:0035023 | GO:0042641 |
| GO:0043244 | GO:0000776 | GO:0006278 | GO:0016607 | GO:0014706 | GO:0005643 |
| GO:0006396 | GO:0032991 | GO:0016233 | GO:0005871 | GO:0006996 | GO:0005654 |
| GO:0000377 | GO:0065003 | GO:0043247 | GO:0031974 | GO:0031323 | GO:0005681 |
| GO:0000375 | GO:0043933 | GO:0007004 | GO:0000790 | GO:0005634 | GO:0032432 |
| GO:0008380 | GO:0043170 | GO:0010833 | GO:0044464 | GO:0043226 | GO:0003723 |
| GO:0005938 | GO:0031974 | GO:0044464 | GO:0005623 | GO:0044424 | GO:0005515 |
| GO:0044464 | GO:0005874 | GO:0042995 | GO:0044430 | GO:0043229 |  |
| GO:0005623 | GO:0005875 | GO:0005623 | GO:0070013 | GO:0043228 |  |
| GO:0016585 | GO:0007017 | GO:0005694 | GO:0000784 | GO:0043232 |  |
| GO:0044427 | GO:0015630 | GO:0070937 | GO:0030672 | GO:0005622 |  |
| GO:0005694 | GO:0016071 | GO:0005737 | GO:0043233 | GO:0044428 |  |
| GO:0000793 | GO:0006397 | GO:0005881 | GO:0003723 | GO:0032991 |  |
| GO:0044430 | GO:0006807 | GO:0044430 | GO:0005515 | GO:0044422 |  |
| GO:0005856 | GO:0043228 | GO:0005856 | GO:0051393 | GO:0044446 |  |
| GO:0005829 | GO:0000228 | GO:0005829 |  | GO:0005694 |  |
| GO:0030530 | GO:0031981 | GO:0030530 |  | GO:0043231 |  |
| GO:0043231 | GO:0000398 | GO:0043231 |  | GO:0043234 |  |
| GO:0043232 | GO:0044428 | GO:0043232 |  | GO:0043227 |  |
| GO:0070013 | GO:0003676 | GO:0070013 |  | GO:0005829 |  |
| GO:0044446 | GO:0006139 | GO:0044446 |  | GO:0044427 |  |
| GO:0043229 | GO:0005730 | GO:0043229 |  | GO:0031981 |  |
| GO:0044424 | GO:0005654 | GO:0044424 |  | GO:0005623 |  |
| GO:0005622 | GO:0034728 | GO:0005622 |  | GO:0044464 |  |
| GO:0032991 | GO:0000166 | GO:0032991 |  | GO:0044430 |  |
| GO:0043227 | GO:0005634 | GO:0043227 |  | GO:0005856 |  |
| GO:0031974 | GO:0043226 | GO:0031974 |  | GO:0030529 |  |
| GO:0015630 | GO:0051640 | GO:0030870 |  | GO:0031974 |  |
| GO:0005874 | GO:0043233 | GO:0043228 |  | GO:0070013 |  |
| GO:0030870 | GO:0006996 | GO:0000784 |  | GO:0070937 |  |
| GO:0043228 | GO:0044422 | GO:0000228 |  | GO:0015630 |  |
| GO:0016604 | GO:0032092 | GO:0031981 |  | GO:0043233 |  |
| GO:0031981 | GO:0044238 | GO:0044428 |  | GO:0030426 |  |
| GO:0044428 | GO:0005515 | GO:0000783 |  | GO:0005697 |  |
| GO:0016607 | GO:0043234 | GO:0005730 |  | GO:0005654 |  |
| GO:0005730 | GO:0032993 | GO:0005654 |  | GO:0030427 |  |
| GO:0005654 | GO:0046605 | GO:0005634 |  | GO:0005874 |  |
| GO:0005634 | GO:0060255 | GO:0043233 |  | GO:0031674 |  |
| GO:0043233 | GO:0070507 | GO:0044422 |  | GO:0005730 |  |
| GO:0044422 | GO:0006974 | GO:0043226 |  | GO:0005875 |  |
| GO:0043226 | GO:0030529 | GO:0043234 |  | GO:0000228 |  |
| GO:0043234 | GO:0003723 | GO:0030529 |  | GO:0043292 |  |
| GO:0005819 | GO:0016070 | GO:0005681 |  | GO:0070161 |  |
| GO:0005681 | GO:0009451 | GO:0000782 |  | GO:0016604 |  |
| GO:0016514 | GO:0006396 | GO:0048487 |  | GO:0005515 |  |
| GO:0070603 | GO:0008380 | GO:0005488 |  | GO:0003723 |  |
| GO:0003779 | GO:0000375 | GO:0008092 |  | GO:0005488 |  |
| GO:0051015 | GO:0000377 | GO:0003684 |  | GO:0003676 |  |
| GO:0030898 | GO:0030427 | GO:0003690 |  | GO:0051734 |  |
| GO:0032559 | GO:0005681 | GO:0003725 |  | GO:0051219 |  |
| GO:0043531 | GO:0000049 | GO:0019899 |  | GO:0051731 |  |
| GO:0005524 | GO:0003697 | GO:0005085 |  | GO:0051733 |  |
| GO:0043008 |  | GO:0051059 |  | GO:0046404 |  |
| GO:0005488 |  | GO:0003676 |  | GO:0008022 |  |
| GO:0005516 |  | GO:0002039 |  | GO:0042162 |  |
| GO:0008092 |  | GO:0005515 |  | GO:0003720 |  |
| GO:0003677 |  | GO:0008022 |  | GO:0003684 |  |
| GO:0019899 |  | GO:0019901 |  | GO:0051015 |  |
| GO:0070577 |  | GO:0004697 |  | GO:0003785 |  |
| GO:0042393 |  | GO:0048365 |  | GO:0003964 |  |
| GO:0016853 |  | GO:0003723 |  | GO:0030695 |  |
| GO:0019900 |  | GO:0005200 |  | GO:0042805 |  |
| GO:0005521 |  | GO:0005198 |  | GO:0060589 |  |
| GO:0003676 |  | GO:0043566 |  | GO:0005085 |  |
| GO:0000166 |  | GO:0042162 |  |  |  |
| GO:0051219 |  | GO:0015631 |  |  |  |
| GO:0030674 |  | GO:0031625 |  |  |  |
| GO:0005515 |  |  |  |  |  |
| GO:0008022 |  |  |  |  |  |
| GO:0019904 |  |  |  |  |  |
| GO:0003723 |  |  |  |  |  |
| GO:0003964 |  |  |  |  |  |
| GO:0005200 |  |  |  |  |  |
| GO:0005198 |  |  |  |  |  |
| GO:0003720 |  |  |  |  |  |
| GO:0042162 |  |  |  |  |  |
