# Supplementary material for: VENNTURE–A Novel Venn Diagram Investigational Tool for Multiple Pharmacological Dataset Analysis
Source: PLoS One. 2012 May 14;7(5):e36911. doi: 10.1371/journal.pone.0036911 (PMC3351456; doi:10.1371/journal.pone.0036911)
Supplement: Table S30 — Cumulated significantly populated Gene Ontology term groups generated from dose-dependent acetyl-β-methylcholine-stimulated phosphoproteins in peroxide (CMP)-treated-state human neuroblastoma SH-SY5Y cells. Ligand stimulation with acetyl-β-methylcholine (MeCh: 10 nM–100 µM) was for 15 minutes before cell lysate protein extraction and titanium dioxide-mediated purification. (DOC) [file pone.0036911.s031.doc]

**Table S30.** Cumulated significantly populated Gene Ontology term groups generated from dose-dependent acetyl-β-methylcholine-stimulated phosphoproteins in peroxide (CMP)-treated-state human neuroblastoma SH-SY5Y cells. Ligand stimulation with acetyl-β-methylcholine (MeCh: 10nM-100μM) was for 15 minutes before cell lysate protein extraction and titanium dioxide-mediated purification.

| **non-stimulated** | **10nM** | **100nM** | **1μM** | **10μM** | **100μM** |
| --- | --- | --- | --- | --- | --- |
| *GO term ID* | *GO term ID* | *GO term ID* | *GO term ID* | *GO term ID* | *GO term ID* |
| GO:0022411 | GO:0000792 | GO:0043232 | GO:0006996 | GO:0044260 | GO:0002062 |
| GO:0016043 | GO:0000785 | GO:0043228 | GO:0043232 | GO:0010467 | GO:0006996 |
| GO:0034623 | GO:0044427 | GO:0000785 | GO:0044446 | GO:0006396 | GO:0006397 |
| GO:0044260 | GO:0005634 | GO:0005634 | GO:0043228 | GO:0022402 | GO:0032330 |
| GO:0043624 | GO:0005694 | GO:0044424 | GO:0005634 | GO:0022403 | GO:0016071 |
| GO:0051276 | GO:0005720 | GO:0005622 | GO:0044427 | GO:0006139 | GO:0021776 |
| GO:0007010 | GO:0043229 | GO:0000790 | GO:0044422 | GO:0000279 | GO:0021775 |
| GO:0007163 | GO:0043226 | GO:0000792 | GO:0044428 | GO:0016071 | GO:0010467 |
| GO:0010467 | GO:0000790 | GO:0043226 | GO:0005694 | GO:0007049 | GO:0016043 |
| GO:0032984 | GO:0005622 | GO:0043229 | GO:0032991 | GO:0043170 | GO:0016568 |
| GO:0043170 | GO:0030027 | GO:0044428 | GO:0000785 | GO:0006807 | GO:0044085 |
| GO:0000226 | GO:0043227 | GO:0044446 | GO:0001725 | GO:0016458 | GO:0051276 |
| GO:0007019 | GO:0043228 | GO:0000228 | GO:0005622 | GO:0031047 | GO:0006338 |
| GO:0031109 | GO:0043231 | GO:0044422 | GO:0032432 | GO:0051028 | GO:0006396 |
| GO:0007017 | GO:0044428 | GO:0044454 | GO:0042641 | GO:0006397 | GO:0051169 |
| GO:0016071 | GO:0043232 | GO:0031981 | GO:0044424 | GO:0005634 | GO:0022607 |
| GO:0006397 | GO:0000228 | GO:0031430 | GO:0060053 | GO:0043228 | GO:0006139 |
| GO:0051129 | GO:0005637 | GO:0005694 | GO:0031941 | GO:0043232 | GO:0043933 |
| GO:0051494 | GO:0044424 | GO:0044427 | GO:0005643 | GO:0044428 | GO:0007049 |
| GO:0007026 | GO:0044454 | GO:0030135 | GO:0005884 | GO:0005622 | GO:0006913 |
| GO:0031111 | GO:0000775 | GO:0001739 | GO:0005515 | GO:0044427 | GO:0065003 |
| GO:0010639 | GO:0003909 | GO:0031672 |  | GO:0044424 | GO:0022402 |
| GO:0043242 | GO:0003910 | GO:0000803 |  | GO:0044454 | GO:0051028 |
| GO:0000398 | GO:0016886 | GO:0015629 |  | GO:0005694 | GO:0034621 |
| GO:0006139 | GO:0003682 | GO:0005856 |  | GO:0000790 | GO:0060579 |
| GO:0006996 | GO:0003676 | GO:0005871 |  | GO:0043226 | GO:0021520 |
| GO:0043241 |  | GO:0005730 |  | GO:0043229 | GO:0021910 |
| GO:0051261 |  | GO:0030136 |  | GO:0044446 | GO:0021514 |
| GO:0051128 |  | GO:0043231 |  | GO:0044422 | GO:0032331 |
| GO:0051493 |  | GO:0043227 |  | GO:0000228 | GO:0007442 |
| GO:0070507 |  | GO:0070013 |  | GO:0031981 | GO:0006325 |
| GO:0031114 |  | GO:0043005 |  | GO:0000785 | GO:0008380 |
| GO:0031110 |  | GO:0008021 |  | GO:0000792 | GO:0021521 |
| GO:0032886 |  | GO:0043233 |  | GO:0032991 | GO:0051168 |
| GO:0033043 |  | GO:0032432 |  | GO:0005643 | GO:0050657 |
| GO:0043244 |  | GO:0031974 |  | GO:0070013 | GO:0051236 |
| GO:0006396 |  | GO:0005720 |  | GO:0031974 | GO:0050658 |
| GO:0000377 |  | GO:0044456 |  | GO:0043233 | GO:0034622 |
| GO:0000375 |  | GO:0044430 |  | GO:0005654 | GO:0010468 |
| GO:0008380 |  | GO:0005875 |  | GO:0046930 | GO:0006403 |
| GO:0005938 |  | GO:0005515 |  | GO:0015629 | GO:0005634 |
| GO:0044464 |  | GO:0005488 |  | GO:0043231 | GO:0044428 |
| GO:0005623 |  | GO:0032405 |  | GO:0043227 | GO:0044424 |
| GO:0016585 |  | GO:0032404 |  | GO:0005720 | GO:0044446 |
| GO:0044427 |  | GO:0000217 |  | GO:0019898 | GO:0044422 |
| GO:0005694 |  | GO:0003690 |  | GO:0005856 | GO:0043229 |
| GO:0044430 |  | GO:0043566 |  | GO:0000178 | GO:0043226 |
| GO:0005856 |  | GO:0017111 |  | GO:0003676 | GO:0005622 |
| GO:0005829 |  | GO:0003676 |  | GO:0005488 | GO:0031981 |
| GO:0030530 |  | GO:0016817 |  | GO:0003723 | GO:0044427 |
| GO:0043231 |  | GO:0016462 |  | GO:0008270 | GO:0043227 |
| GO:0043232 |  | GO:0016818 |  | GO:0003677 | GO:0005694 |
| GO:0070013 |  | GO:0003677 |  | GO:0043566 | GO:0043231 |
| GO:0044446 |  |  |  | GO:0046914 | GO:0005654 |
| GO:0043229 |  |  |  | GO:0003690 | GO:0070013 |
| GO:0044424 |  |  |  | GO:0030983 | GO:0000793 |
| GO:0005622 |  |  |  | GO:0005515 | GO:0043233 |
| GO:0032991 |  |  |  |  | GO:0005719 |
| GO:0043227 |  |  |  |  | GO:0043232 |
| GO:0031974 |  |  |  |  | GO:0043228 |
| GO:0005875 |  |  |  |  | GO:0031974 |
| GO:0015630 |  |  |  |  | GO:0000790 |
| GO:0005874 |  |  |  |  | GO:0016363 |
| GO:0043228 |  |  |  |  | GO:0000791 |
| GO:0016604 |  |  |  |  | GO:0044454 |
| GO:0005635 |  |  |  |  | GO:0034399 |
| GO:0031981 |  |  |  |  | GO:0005635 |
| GO:0044428 |  |  |  |  | GO:0000785 |
| GO:0016607 |  |  |  |  | GO:0000779 |
| GO:0005730 |  |  |  |  | GO:0000792 |
| GO:0044451 |  |  |  |  | GO:0015629 |
| GO:0005654 |  |  |  |  | GO:0032991 |
| GO:0005634 |  |  |  |  | GO:0005637 |
| GO:0043233 |  |  |  |  | GO:0043234 |
| GO:0044422 |  |  |  |  | GO:0000228 |
| GO:0043226 |  |  |  |  | GO:0005720 |
| GO:0043234 |  |  |  |  | GO:0000777 |
| GO:0030529 |  |  |  |  | GO:0005938 |
| GO:0005876 |  |  |  |  | GO:0000178 |
| GO:0005681 |  |  |  |  | GO:0019902 |
| GO:0003779 |  |  |  |  | GO:0003723 |
| GO:0051015 |  |  |  |  | GO:0004035 |
| GO:0030554 |  |  |  |  | GO:0003676 |
| GO:0032559 |  |  |  |  | GO:0019899 |
| GO:0005524 |  |  |  |  | GO:0019903 |
| GO:0016887 |  |  |  |  | GO:0003909 |
| GO:0008026 |  |  |  |  | GO:0003910 |
| GO:0005488 |  |  |  |  | GO:0005515 |
| GO:0005516 |  |  |  |  | GO:0016886 |
| GO:0004693 |  |  |  |  | GO:0005488 |
| GO:0008092 |  |  |  |  |  |
| GO:0003918 |  |  |  |  |  |
| GO:0008047 |  |  |  |  |  |
| GO:0019899 |  |  |  |  |  |
| GO:0005096 |  |  |  |  |  |
| GO:0004386 |  |  |  |  |  |
| GO:0070577 |  |  |  |  |  |
| GO:0042393 |  |  |  |  |  |
| GO:0016818 |  |  |  |  |  |
| GO:0016817 |  |  |  |  |  |
| GO:0008017 |  |  |  |  |  |
| GO:0003676 |  |  |  |  |  |
| GO:0001882 |  |  |  |  |  |
| GO:0017111 |  |  |  |  |  |
| GO:0000166 |  |  |  |  |  |
| GO:0005515 |  |  |  |  |  |
| GO:0008022 |  |  |  |  |  |
| GO:0004672 |  |  |  |  |  |
| GO:0004674 |  |  |  |  |  |
| GO:0070035 |  |  |  |  |  |
| GO:0001883 |  |  |  |  |  |
| GO:0017076 |  |  |  |  |  |
| GO:0032555 |  |  |  |  |  |
| GO:0016462 |  |  |  |  |  |
| GO:0032553 |  |  |  |  |  |
| GO:0003723 |  |  |  |  |  |
| GO:0005200 |  |  |  |  |  |
| GO:0003720 |  |  |  |  |  |
| GO:0042162 |  |  |  |  |  |
| GO:0015631 |  |  |  |  |  |
