# Supplementary material for: VENNTURE–A Novel Venn Diagram Investigational Tool for Multiple Pharmacological Dataset Analysis
Source: PLoS One. 2012 May 14;7(5):e36911. doi: 10.1371/journal.pone.0036911 (PMC3351456; doi:10.1371/journal.pone.0036911)
Supplement: Table S31 — MeCh dose-unique GO term population in control-state SH-SY5Y cells. The GO term groups uniquely and significantly (p≤0.05, n>2 proteins per group) at the specified MeCh dose only, in control-state SH-SY5Y cells are indicated. Hybrid scores for GO term population were generated by multiplication of the GO term enrichment ratio with the –log10 of the enrichment probability. (DOC) [file pone.0036911.s032.doc]

**Table S31.** MeCh dose-unique GO term population in control-state SH-SY5Y. The GO term groups uniquely and significantly (*p*≤0.05, n>2 proteins per group) at the specified MeCh dose only, in control-state SH-SY5Y cells are indicated. Hybrid scores for GO term population were generated by multiplication of the GO term enrichment ratio with the –log_10_ of the enrichment probability.

| **10nM MeCh** |  |  |
| --- | --- | --- |
| **GO code** | **GO term** | **Hybrid** |
| GO:0000132 | establishment of mitotic spindle orientation | 55.58873801 |
| GO:0051294 | establishment of spindle orientation | 55.58873801 |
| GO:0032092 | positive regulation of protein binding | 46.49227159 |
| GO:0040001 | establishment of mitotic spindle localization | 38.40458817 |
| GO:0005858 | axonemal dynein complex | 30.24145612 |
| GO:0000776 | kinetochore | 27.88933333 |
| GO:0000049 | tRNA binding | 24.49607117 |
| GO:0044447 | axoneme part | 18.8248776 |
| GO:0030286 | dynein complex | 16.78582364 |
| GO:0009451 | RNA modification | 14.7322852 |
| GO:0070507 | regulation of microtubule cytoskeleton organization | 13.1358199 |
| GO:0051640 | organelle localization | 9.608222017 |
| GO:0051656 | establishment of organelle localization | 8.866758328 |
| GO:0034728 | nucleosome organization | 8.689026369 |
| GO:0006333 | chromatin assembly or disassembly | 7.686577613 |
| GO:0032993 | protein-DNA complex | 7.500933078 |
| GO:0065003 | macromolecular complex assembly | 6.522396824 |
| GO:0043933 | macromolecular complex subunit organization | 6.258623423 |
| GO:0034622 | cellular macromolecular complex assembly | 5.82826841 |
| GO:0044085 | cellular component biogenesis | 5.56322082 |
| GO:0034621 | cellular macromolecular complex subunit organization | 5.417071559 |
| GO:0022607 | cellular component assembly | 5.199353462 |
| GO:0006281 | DNA repair | 5.119269144 |
| GO:0003697 | nucleic acid binding | 2.21815062 |
| GO:0044237 | cellular metabolic process | 1.9391171 |
| GO:0044238 | primary metabolic process | 1.640182884 |
|  |  |  |
| **100nM MeCh** |  |  |
| **GO code** | **GO term** | **Hybrid** |
| GO:0043247 | telomere maintenance in response to DNA damage | 175.0236054 |
| GO:0016233 | telomere capping | 120.8314357 |
| GO:0070198 | protein localization to chromosome, telomeric region | 71.35566007 |
| GO:0008340 | determination of adult lifespan | 59.0150029 |
| GO:0034502 | protein localization to chromosome | 59.0150029 |
| GO:0007004 | telomere maintenance via telomerase | 56.26439912 |
| GO:0010833 | telomere maintenance via telomere lengthening | 56.26439912 |
| GO:0000782 | telomere cap complex | 54.51380358 |
| GO:0000783 | nuclear telomere cap complex | 54.51380358 |
| GO:0006278 | RNA-dependent DNA replication | 40.28364783 |
| GO:0003725 | double-stranded RNA binding | 39.0348525 |
| GO:0048365 | Rac GTPase binding | 31.89631969 |
| GO:0051053 | negative regulation of DNA metabolic process | 25.58813126 |
| GO:0010970 | microtubule-based transport | 23.82945441 |
| GO:0051059 | NF-kappaB binding | 23.76915057 |
| GO:0004697 | protein kinase C activity | 22.18544293 |
| GO:0048487 | beta-tubulin binding | 22.18544293 |
| GO:0051651 | maintenance of location in cell | 19.03693209 |
| GO:0031625 | ubiquitin protein ligase binding | 14.67365001 |
| GO:0007018 | microtubule-based movement | 13.24704199 |
| GO:0051235 | maintenance of location | 10.75577193 |
| GO:0080135 | regulation of cellular response to stress | 10.43893524 |
| GO:0080134 | regulation of response to stress | 9.154885281 |
| GO:0043566 | structure-specific DNA binding | 9.098090168 |
| GO:0003690 | double-stranded DNA binding | 7.309419878 |
| GO:0015631 | tubulin binding | 7.099119712 |
| GO:0019901 | protein kinase binding | 5.861071865 |
| GO:0010605 | negative regulation of macromolecule metabolic process | 4.841570135 |
| GO:0009892 | negative regulation of metabolic process | 4.202255081 |
| GO:0051716 | cellular response to stimulus | 4.067156713 |
| GO:0042995 | cell projection | 3.665702664 |
| GO:0010468 | regulation of gene expression | 3.442620334 |
| GO:0005737 | cytoplasm | 2.793364147 |
|  |  |  |
| **1μM MeCh** |  |  |
| **GO code** | **GO term** | **Hybrid** |
| GO:0051393 | alpha-actinin binding | 55.25869056 |
| GO:0031519 | PcG protein complex | 51.29460982 |
| GO:0016574 | histone ubiquitination | 39.3960355 |
| GO:0000781 | chromosome, telomeric region | 29.63140003 |
| GO:0005871 | kinesin complex | 23.65697093 |
| GO:0006413 | translational initiation | 18.3438191 |
| GO:0030672 | synaptic vesicle membrane | 18.29090383 |
| GO:0000118 | histone deacetylase complex | 17.26009183 |
| GO:0044454 | nuclear chromosome part | 11.48137697 |
| GO:0000790 | nuclear chromatin | 11.14569521 |
|  |  |  |
| **10μM MeCh** |  |  |
| **GO code** | **GO term** | **Hybrid** |
| GO:0051734 | ATP-dependent polynucleotide kinase activity | 141.8960329 |
| GO:0051733 | polydeoxyribonucleotide kinase activity | 141.8960329 |
| GO:0051731 | polynucleotide kinase activity | 141.8960329 |
| GO:0046404 | ATP-dependent polydeoxyribonucleotide 5'-hydroxyl-kinase activity | 141.8960329 |
| GO:0005697 | telomerase holoenzyme complex | 68.43543032 |
| GO:0003785 | actin monomer binding | 35.3468082 |
| GO:0042805 | actinin binding | 26.019462 |
| GO:0000723 | telomere maintenance | 24.12529408 |
| GO:0032200 | telomere organization | 23.3654423 |
| GO:0000718 | nucleotide-excision repair, DNA damage removal | 19.98433271 |
| GO:0043488 | regulation of mRNA stability | 19.0746618 |
| GO:0043487 | regulation of RNA stability | 16.92133022 |
| GO:0030261 | chromosome condensation | 16.1746468 |
| GO:0031674 | I band | 15.62564815 |
| GO:0051493 | regulation of cytoskeleton organization | 7.10680395 |
| GO:0035023 | regulation of Rho protein signal transduction | 6.943727623 |
| GO:0043292 | contractile fiber | 6.787103114 |
| GO:0007266 | Rho protein signal transduction | 6.272640332 |
| GO:0070161 | anchoring junction | 5.485300747 |
| GO:0014706 | striated muscle tissue development | 4.982522111 |
| GO:0051056 | regulation of small GTPase mediated signal transduction | 4.960549157 |
| GO:0030695 | GTPase regulator activity | 3.564915141 |
| GO:0006259 | DNA metabolic process | 3.553401975 |
| GO:0060589 | nucleoside-triphosphatase regulator activity | 3.429027675 |
| GO:0009987 | cellular process | 2.234683375 |
| GO:0031323 | regulation of cellular metabolic process | 1.875989746 |
|  |  |  |
| **100μM MeCh** |  |  |
| **GO code** | **GO term** | **Hybrid** |
| GO:0001725 | stress fiber | 18.21763181 |
| GO:0032432 | actin filament bundle | 16.37376376 |
| GO:0042641 | actomyosin | 15.72194195 |
| GO:0000502 | proteasome complex | 10.28088126 |
| GO:0051236 | establishment of RNA localization | 9.87064728 |
| GO:0050658 | RNA transport | 9.87064728 |
| GO:0050657 | nucleic acid transport | 9.87064728 |
| GO:0006403 | RNA localization | 9.592991942 |
| GO:0005643 | nuclear pore | 8.304209804 |
