# Supplementary material for: VENNTURE–A Novel Venn Diagram Investigational Tool for Multiple Pharmacological Dataset Analysis
Source: PLoS One. 2012 May 14;7(5):e36911. doi: 10.1371/journal.pone.0036911 (PMC3351456; doi:10.1371/journal.pone.0036911)
Supplement: Table S32 — MeCh dose-unique GO term population in CMP-state SH-SY5Y cells. The GO term groups uniquely and significantly (p≤0.05, n>2 proteins per group) at the specified MeCh dose only, in CMP-state SH-SY5Y cells are indicated. Hybrid scores for GO term population were generated by multiplication of the GO term enrichment ratio with the –log10 of the enrichment probability. (DOC) [file pone.0036911.s033.doc]

**Table S32.** MeCh dose-unique GO term population in CMP-state SH-SY5Y. The GO term groups uniquely and significantly (*p*≤0.05, n>2 proteins per group) at the specified MeCh dose only, in CMP-state SH-SY5Y cells are indicated. Hybrid scores for GO term population were generated by multiplication of the GO term enrichment ratio with the –log_10_ of the enrichment probability.

| **10nM MeCh** |  |  |
| --- | --- | --- |
| **GO code** | **GO term** | **Hybrid** |
| GO:0030027 | lamellipodium | 21.76833 |
| GO:0003682 | chromatin binding | 19.17904 |
| GO:0000775 | chromosome, centromeric region | 9.023798 |
|  |  |  |
| **100nM MeCh** |  |  |
| **GO code** | **GO term** | **Hybrid** |
| GO:0031430 | M band | 103.8471 |
| GO:0032405 | MutLalpha complex binding | 99.97856 |
| GO:0032404 | mismatch repair complex binding | 69.29547 |
| GO:0031672 | A band | 49.00433 |
| GO:0001739 | sex chromatin | 49.00433 |
| GO:0000803 | sex chromosome | 45.56713 |
| GO:0005871 | kinesin complex | 38.17314 |
| GO:0008021 | synaptic vesicle | 11.69944 |
| GO:0030135 | coated vesicle | 11.61633 |
| GO:0030136 | clathrin-coated vesicle | 9.205616 |
| GO:0044456 | synapse part | 5.642307 |
| GO:0043005 | neuron projection | 5.443819 |
|  |  |  |
| **1μM MeCh** |  |  |
| **GO code** | **GO term** | **Hybrid** |
| GO:0060053 | neurofilament cytoskeleton | 24.29 |
| GO:0031941 | filamentous actin | 16.19 |
| GO:0001725 | stress fiber | 13.25 |
| GO:0042641 | actomyosin | 11.66 |
| GO:0005884 | actin filament | 7.88 |
|  |  |  |
| **10μM MeCh** |  |  |
| **GO code** | **GO term** | **Hybrid** |
| GO:0031047 | gene silencing by RNA | 14.20526 |
| GO:0016458 | gene silencing | 10.27306 |
| GO:0046930 | pore complex | 8.076789 |
| GO:0000279 | M phase | 5.263819 |
| GO:0008270 | zinc ion binding | 4.994276 |
| GO:0022403 | cell cycle phase | 4.849616 |
| GO:0019898 | extrinsic to membrane | 3.318734 |
| GO:0046914 | transition metal ion binding | 2.914897 |
| GO:0006807 | nitrogen compound metabolic process | 2.002375 |
|  |  |  |
| **100μM MeCh** |  |  |
| **GO code** | **GO term** | **Hybrid** |
| GO:0021776 | smoothened signaling pathway involved in spinal cord motor neuron cell fate specification | 119.2612 |
| GO:0021775 | smoothened signaling pathway involved in ventral spinal cord interneuron specification | 119.2612 |
| GO:0005719 | nuclear euchromatin | 114.8121 |
| GO:0004035 | alkaline phosphatase activity | 88.6635 |
| GO:0000791 | euchromatin | 78.81712 |
| GO:0060579 | ventral spinal cord interneuron fate commitment | 45.14255 |
| GO:0032331 | negative regulation of chondrocyte differentiation | 45.14255 |
| GO:0021910 | smoothened signaling pathway involved in ventral spinal cord patterning | 45.14255 |
| GO:0021521 | ventral spinal cord interneuron specification | 45.14255 |
| GO:0021520 | spinal cord motor neuron cell fate specification | 45.14255 |
| GO:0021514 | ventral spinal cord interneuron differentiation | 45.14255 |
| GO:0007442 | hindgut morphogenesis | 45.14255 |
| GO:0002062 | chondrocyte differentiation | 30.50902 |
| GO:0032330 | regulation of chondrocyte differentiation | 29.80757 |
| GO:0019902 | phosphatase binding | 24.57284 |
| GO:0019903 | protein phosphatase binding | 18.52405 |
| GO:0016363 | nuclear matrix | 14.41281 |
| GO:0034399 | nuclear periphery | 12.75478 |
| GO:0000793 | condensed chromosome | 11.7258 |
| GO:0000779 | condensed chromosome, centromeric region | 11.35541 |
| GO:0006338 | chromatin remodeling | 10.22055 |
| GO:0051168 | nuclear export | 8.351104 |
| GO:0000777 | condensed chromosome kinetochore | 7.387605 |
| GO:0051236 | establishment of RNA localization | 6.659949 |
| GO:0050658 | RNA transport | 6.659949 |
| GO:0050657 | nucleic acid transport | 6.659949 |
| GO:0006403 | RNA localization | 6.325234 |
| GO:0006913 | nucleocytoplasmic transport | 5.616699 |
| GO:0051169 | nuclear transport | 5.586006 |
| GO:0016568 | chromatin modification | 5.140301 |
| GO:0034621 | cellular macromolecular complex subunit organization | 3.986106 |
| GO:0006325 | chromatin organization | 3.856839 |
| GO:0034622 | cellular macromolecular complex assembly | 3.682692 |
| GO:0065003 | macromolecular complex assembly | 3.422196 |
| GO:0043933 | macromolecular complex subunit organization | 3.253388 |
| GO:0022607 | cellular component assembly | 3.069234 |
| GO:0044085 | cellular component biogenesis | 2.961811 |
| GO:0010468 | regulation of gene expression | 2.038131 |
