# Supplementary material for: VENNTURE–A Novel Venn Diagram Investigational Tool for Multiple Pharmacological Dataset Analysis
Source: PLoS One. 2012 May 14;7(5):e36911. doi: 10.1371/journal.pone.0036911 (PMC3351456; doi:10.1371/journal.pone.0036911)
Supplement: Table S33 — Canonical signaling pathways populated by extracted phosphoproteins in control-state SH-SY5Y cells. Canonical signaling pathways enriched by phosphoproteins extracted from non-stimulated and MeCh-stimulated control-state SH-SY5Y cells are represented in a simple matrix format. Canonical signaling pathways were considered enriched only if at least two proteins were present in each signaling pathway and with a probability of ≤0.05. Hybrid signaling pathway scores, indicated in specific cells of the matrix, were generated by multiplication of the pathway enrichment ratio with the negative log10 of the probability result. Cells in the matrix not possessing a hybrid score were not significantly populated by proteins in that specific stimulation condition. (DOC) [file pone.0036911.s034.doc]

**Table S33**. Canonical signaling pathways populated by extracted phosphoproteins in control-state SH-SY5Y cells. Canonical signaling pathways enriched by phosphoproteins extracted from non-stimulated and MeCh-stimulated control-state SH-SY5Y cells are represented in a simple matrix format. Canonical signaling pathways were considered enriched only if at least two proteins were present in each signaling pathway and with a probability of ≤0.05. Hybrid signaling pathway scores, indicated in specific cells of the matrix, were generated by multiplication of the pathway enrichment ratio with the negative log_10_ of the probability result. Cells in the matrix not possessing a hybrid score were not significanlty populated by proteins in that specific stimulation condition.

| **Canonical signaling pathway** | **non-stimulated** | **10nM** | **100nM** | **1μM** | **10μM** | **100μM** |
| --- | --- | --- | --- | --- | --- | --- |
| 14-3-3-mediated Signaling |  |  | 27 |  |  | 4.93 |
| Actin Cytoskeleton Signaling | 20.7 | 2.3 | 3.02 | 7.08 | 11.8 | 7.59 |
| Activation of IRF by Cytosolic Pattern Recognition Receptors |  |  | 10.1 |  |  |  |
| Acute Myeloid Leukemia Signaling |  |  | 9.26 |  |  |  |
| Agrin Interactions at Neuromuscular Junction |  | 8.2 |  | 17.9 | 30.3 |  |
| Aldosterone Signaling in Epithelial Cells |  |  | 8.51 |  |  |  |
| AMPK Signaling |  | 4.61 |  |  |  |  |
| Amyloid Processing |  |  |  |  | 9.94 |  |
| Androgen Signaling |  |  | 6.14 |  | 4.62 |  |
| Angiopoietin Signaling |  |  |  |  | 8.41 |  |
| Arachidonic Acid Metabolism |  |  | 5.43 |  |  |  |
| Aryl Hydrocarbon Receptor Signaling |  | 4.07 | 4.99 | 10.6 | 7.35 | 11.2 |
| Ascorbate and Aldarate Metabolism |  | 16.7 |  |  |  |  |
| Assembly of RNA Polymerase III Complex | 19.6 |  |  |  |  |  |
| ATM Signaling | 14.8 | 10.5 |  |  |  |  |
| Axonal Guidance Signaling |  |  |  | 2.92 | 7.31 | 3.26 |
| B Cell Receptor Signaling | 24.5 | 3.88 | 4.78 |  |  |  |
| Calcium Signaling |  |  |  |  | 9.51 | 4.61 |
| cAMP-mediated Signaling |  |  |  |  | 3.01 |  |
| Cardiac Hypertrophy Signaling |  |  |  | 6.28 | 3.72 |  |
| Cardiac β-adrenergic Signaling |  |  |  |  | 8.77 |  |
| Caveolar-mediated Endocytosis Signaling |  |  | 16.8 |  |  | 10.4 |
| CCR3 Signaling in Eosinophils |  |  |  |  | 4.77 |  |
| Cdc42 Signaling |  |  |  | 6.89 | 21.7 |  |
| CDK5 Signaling | 15.2 |  |  |  | 6.03 |  |
| Cellular Effects of Sildenafil (Viagra) |  |  |  |  | 8.09 |  |
| Cholecystokinin/Gastrin-mediated Signaling |  |  | 7.05 |  |  |  |
| Clathrin-mediated Endocytosis Signaling |  |  | 4.55 |  | 3.22 |  |
| Cleavage and Polyadenylation of Pre-mRNA |  |  |  | 10.9 |  |  |
| Colorectal Cancer Metastasis Signaling |  |  | 2.55 |  |  |  |
| Corticotropin Releasing Hormone Signaling |  |  | 6.2 |  |  |  |
| CREB Signaling in Neurons |  |  | 13.2 |  |  |  |
| CXCR4 Signaling |  |  | 4.55 | 5.06 | 11.2 |  |
| Death Receptor Signaling |  |  |  | 11.4 |  |  |
| DNA Methylation and Transcriptional Repression Signaling |  |  |  | 20 |  |  |
| EGF Signaling |  |  | 12.5 |  |  |  |
| EIF2 Signaling |  | 7.21 | 8.34 | 16.2 |  |  |
| Endothelin-1 Signaling |  |  | 4.09 | 4.58 |  |  |
| Ephrin Receptor Signaling |  |  |  |  | 5.51 | 4.57 |
| ERK/MAPK Signaling |  |  | 24.6 | 4.13 | 9.17 | 4.44 |
| Erythropoietin Signaling |  |  | 9.9 |  |  |  |
| Estrogen Receptor Signaling |  |  | 12.3 |  |  |  |
| FAK Signaling |  |  | 8.17 | 8.8 | 12.2 |  |
| Fc Epsilon RI Signaling |  |  | 13.4 |  |  |  |
| Fcγ Receptor-mediated Phagocytosis in Macrophages and Monocytes |  |  | 14 |  | 5.96 |  |
| FcγRIIB Signaling in B Lymphocytes |  |  | 14 |  |  |  |
| FGF Signaling |  |  | 8.42 |  |  |  |
| FLT3 Signaling in Hematopoietic Progenitor Cells |  |  | 9.57 |  |  |  |
| G Beta Gamma Signaling |  |  | 7.69 |  | 6.03 |  |
| Germ Cell-Sertoli Cell Junction Signaling |  | 7.52 | 4.59 | 5.1 | 6.74 |  |
| Glioma Signaling |  |  | 7.26 |  |  |  |
| Glucocorticoid Receptor Signaling |  |  |  | 5.45 | 3.08 |  |
| Glycolysis/Gluconeogenesis |  | 7.13 |  |  |  |  |
| GM-CSF Signaling |  |  | 10.2 |  |  |  |
| GNRH Signaling |  | 4.65 | 5.62 |  | 8.31 |  |
| G-Protein Coupled Receptor Signaling |  |  | 3.02 |  |  |  |
| Hepatic Cholestasis |  |  |  |  | 3.83 |  |
| HGF Signaling | 13.2 |  | 7.05 |  |  |  |
| Huntington's Disease Signaling |  |  | 15.6 | 6.95 |  |  |
| IGF-1 Signaling |  |  | 7.77 |  |  |  |
| IL-12 Signaling and Production in Macrophages |  |  | 12.2 |  |  |  |
| IL-3 Signaling |  |  | 9.37 |  |  |  |
| IL-8 Signaling |  |  | 4 |  |  |  |
| ILK Signaling |  |  | 3.9 |  | 2.67 | 4.71 |
| Inositol Phosphate Metabolism | 19.5 |  |  |  | 13 |  |
| Insulin Receptor Signaling |  |  | 5.53 |  | 4.07 |  |
| Integrin Signaling |  |  | 3.36 | 3.81 | 4.88 |  |
| Leukocyte Extravasation Signaling |  |  |  |  | 2.57 | 4.57 |
| LPS/IL-1 Mediated Inhibition of RXR Function |  | 2.46 |  |  |  |  |
| Metabolism of Xenobiotics by Cytochrome P450 |  | 4.43 |  |  |  |  |
| Mitotic Roles of Polo-Like Kinase |  | 10 |  |  |  |  |
| Molecular Mechanisms of Cancer |  | 3.83 |  | 6.07 | 8.24 |  |
| mTOR Signaling |  |  | 9.93 | 5.65 |  |  |
| Myc Mediated Apoptosis Signaling |  |  | 11 |  |  |  |
| Natural Killer Cell Signaling |  |  | 6.73 |  | 5.15 |  |
| Neuregulin Signaling |  |  | 7.77 |  |  |  |
| Neuropathic Pain Signaling In Dorsal Horn Neurons |  |  | 7.05 |  | 10.5 |  |
| Nicotinate and Nicotinamide Metabolism | 13.3 |  |  |  | 11.1 |  |
| Nitric Oxide Signaling in the Cardiovascular System |  |  |  |  | 7.39 |  |
| Non-Small Cell Lung Cancer Signaling |  |  | 9.9 |  |  |  |
| NRF2-mediated Oxidative Stress Response |  | 2.92 | 3.72 |  |  | 4.51 |
| Nucleotide Excision Repair Pathway |  |  |  |  | 12.9 |  |
| p38 MAPK Signaling |  |  |  | 8.08 |  |  |
| p70S6K Signaling |  |  | 17.7 |  | 4.38 |  |
| PDGF Signaling |  |  | 9.26 |  |  |  |
| PI3K/AKT Signaling |  |  | 11.5 |  |  |  |
| Polyamine Regulation in Colon Cancer |  | 10.9 |  |  |  |  |
| PPARα/RXRα Activation |  |  | 3.84 |  |  |  |
| Prolactin Signaling |  |  | 9.26 |  |  |  |
| Protein Ubiquitination Pathway |  | 11.8 |  |  |  | 5.17 |
| Purine Metabolism |  | 2.81 | 10.7 |  |  |  |
| PXR/RXR Activation |  |  |  |  | 7.78 |  |
| Pyrimidine Metabolism |  | 4.23 | 10 |  | 7.61 |  |
| Rac Signaling | 12.7 |  |  |  |  |  |
| RAR Activation |  |  | 4.16 |  | 6.09 |  |
| Reelin Signaling in Neurons |  |  | 9.07 | 17.4 | 13.5 | 10.1 |
| Regulation of Actin-based Motility by Rho |  |  |  |  | 12.7 |  |
| Renal Cell Carcinoma Signaling |  |  |  |  | 7.68 |  |
| Renin-Angiotensin Signaling |  |  | 6.61 | 7.19 | 9.8 |  |
| RhoA Signaling | 24.8 |  |  |  |  |  |
| Role of BRCA1 in DNA Damage Response |  |  | 11.9 |  |  |  |
| Role of NFAT in Regulation of the Immune Response |  |  | 4 |  |  |  |
| Semaphorin Signaling in Neurons | 14.8 |  |  |  | 16.9 |  |
| Starch and Sucrose Metabolism |  |  | 8.17 |  |  |  |
| Synaptic Long Term Depression |  |  | 5.03 |  |  |  |
| Synaptic Long Term Potentiation |  |  |  |  | 4.93 |  |
| Systemic Lupus Erythematosus Signaling |  |  | 10.2 |  | 3.83 |  |
| Thrombin Signaling |  |  | 3.44 |  | 2.29 |  |
| Thrombopoietin Signaling |  |  | 11.3 |  |  |  |
| Tight Junction Signaling |  |  |  |  | 11.8 | 5.7 |
| TREM1 Signaling |  |  |  |  |  | 12.3 |
| VEGF Signaling |  |  | 8.17 |  |  |  |
| Virus Entry via Endocytic Pathways |  |  | 14.7 |  |  |  |
| Wnt/β-catenin Signaling |  |  |  |  |  | 5.25 |
| Xenobiotic Metabolism Signaling |  | 3.29 |  | 2.3 |  |  |
