# Supplementary material for: VENNTURE–A Novel Venn Diagram Investigational Tool for Multiple Pharmacological Dataset Analysis
Source: PLoS One. 2012 May 14;7(5):e36911. doi: 10.1371/journal.pone.0036911 (PMC3351456; doi:10.1371/journal.pone.0036911)
Supplement: Table S34 — Canonical signaling pathways populated by extracted phosphoproteins in CMP-state SH-SY5Y cells. Canonical signaling pathways enriched by phosphoproteins extracted from non-stimulated and MeCh-stimulated CMP-state SH-SY5Y cells are represented in a simple matrix format. Canonical signaling pathways were considered enriched only if at least two proteins were present in each signaling pathway and with a probability of ≤0.05. Hybrid signaling pathway scores, indicated in specific cells of the matrix, were generated by multiplication of the pathway enrichment ratio with the negative log10 of the probability result. Cells in the matrix not possessing a hybrid score were not significantly populated by proteins in that specific stimulation condition. (DOC) [file pone.0036911.s035.doc]

**Table S34.** Canonical signaling pathways populated by extracted phosphoproteins in CMP-state SH-SY5Y cells. Canonical signaling pathways enriched by phosphoproteins extracted from non-stimulated and MeCh-stimulated CMP-state SH-SY5Y cells are represented in a simple matrix format. Canonical signaling pathways were considered enriched only if at least two proteins were present in each signaling pathway and with a probability of ≤0.05. Hybrid signaling pathway scores, indicated in specific cells of the matrix, were generated by multiplication of the pathway enrichment ratio with the negative log_10_ of the probability result. Cells in the matrix not possessing a hybrid score were not significanlty populated by proteins in that specific stimulation condition.

| **Canonical signaling pathway** | **non-stimulated** | **10nM** | **100nM** | **1μM** | **10μM** | **100μM** |
| --- | --- | --- | --- | --- | --- | --- |
| 14-3-3-mediated Signaling | 2.11 |  |  |  | 4.11 |  |
| Actin Cytoskeleton Signaling |  |  |  |  | 3.39 |  |
| Activation of IRF by Cytosolic Pattern Recognition Receptors |  |  |  |  | 13.5 | 6.69 |
| Agrin Interactions at Neuromuscular Junction |  |  | 12.1 |  | 6.61 |  |
| Aminoacyl-tRNA Biosynthesis |  |  |  |  | 6.16 |  |
| Aminosugars Metabolism |  |  |  |  |  | 13.1 |
| AMPK Signaling |  |  |  |  | 3.36 | 2.92 |
| Amyotrophic Lateral Sclerosis Signaling |  |  |  |  | 5.2 |  |
| Androgen Signaling |  |  | 8.61 |  | 7.74 | 3.36 |
| Angiopoietin Signaling |  |  |  |  | 7.4 |  |
| Apoptosis Signaling | 3.05 |  |  |  |  |  |
| April Mediated Signaling |  |  |  |  | 11.1 |  |
| Arachidonic Acid Metabolism |  |  |  |  | 3.25 |  |
| Assembly of RNA Polymerase III Complex | 1.49 |  |  |  |  |  |
| ATM Signaling | 1.45 |  |  |  |  |  |
| Axonal Guidance Signaling |  | 3.58 |  | 5.34 | 11.1 | 4.41 |
| B Cell Activating Factor Signaling |  |  |  |  | 10.7 |  |
| B Cell Receptor Signaling |  |  |  | 3.73 | 14.8 | 2.34 |
| Basal Cell Carcinoma Signaling |  |  |  |  | 7.4 | 12.7 |
| Bladder Cancer Signaling |  |  |  | 6.64 |  |  |
| BMP signaling pathway |  |  |  |  | 6.26 |  |
| Breast Cancer Regulation by Stathmin1 | 1.36 |  |  |  |  |  |
| Calcium Signaling |  | 8.01 |  |  |  |  |
| Calcium Signaling |  |  |  |  | 4.46 |  |
| cAMP-mediated Signaling |  |  |  |  | 2.36 |  |
| Cardiac Hypertrophy Signaling |  |  |  |  | 2.82 |  |
| Cardiac β-adrenergic Signaling |  |  |  |  | 3.65 | 6.63 |
| Caveolar-mediated Endocytosis Signaling |  |  | 12.1 |  |  | 11.4 |
| CCR3 Signaling in Eosinophils |  |  | 15.9 |  | 7.98 |  |
| CD28 Signaling in T Helper Cells |  |  |  |  | 8.06 |  |
| CD40 Signaling |  |  |  |  | 7.4 |  |
| Cdc42 Signaling |  |  | 3.27 |  | 7.98 |  |
| CDK5 Signaling |  |  |  |  | 5.14 | 4.61 |
| Cellular Effects of Sildenafil (Viagra) |  |  | 7.86 |  |  |  |
| Ceramide Signaling |  |  |  |  | 11.8 |  |
| Chemokine Signaling |  |  |  |  | 6.34 |  |
| Chronic Myeloid Leukemia Signaling |  |  |  |  | 4.82 |  |
| Colorectal Cancer Metastasis Signaling |  |  | 4.36 |  | 8.35 |  |
| Corticotropin Releasing Hormone Signaling |  |  | 8.67 |  | 7.82 | 7 |
| CXCR4 Signaling |  |  | 6.79 |  | 5.48 |  |
| Dendritic Cell Maturation |  |  |  |  | 2.44 |  |
| DNA Double-Strand Break Repair by Homologous Recombination | 1.37 |  |  |  |  |  |
| DNA Methylation and Transcriptional Repression Signaling | 1.94 | 25.1 |  | 17.6 |  |  |
| Dopamine Receptor Signaling |  |  |  |  | 6.34 | 5.76 |
| EGF Signaling |  |  |  |  | 9.48 |  |
| Eicosanoid Signaling |  |  |  |  | 2.75 |  |
| Endometrial Cancer Signaling |  |  |  |  | 8.6 |  |
| Endothelin-1 Signaling |  |  | 18.6 |  |  |  |
| Ephrin Receptor Signaling |  |  |  | 2.84 | 12 |  |
| ERK/MAPK Signaling |  |  | 11 |  | 7.53 | 3.64 |
| Erythropoietin Signaling |  |  |  |  | 7.09 |  |
| Estrogen Receptor Signaling | 1.47 |  |  |  |  | 3.68 |
| Factors Promoting Cardiogenesis in Vertebrates |  |  |  |  | 5.77 |  |
| FAK Signaling |  |  |  |  | 5.55 |  |
| Fatty Acid Biosynthesis | 1.49 |  |  |  |  |  |
| Fatty Acid Metabolism |  |  |  |  |  | 2.75 |
| Fcγ Receptor-mediated Phagocytosis in Macrophages and Monocytes |  |  | 10.2 |  |  | 4.54 |
| FLT3 Signaling in Hematopoietic Progenitor Cells |  |  |  |  | 6.8 |  |
| fMLP Signaling in Neutrophils |  |  |  |  | 4.16 |  |
| Folate Biosynthesis |  |  |  |  | 18.9 | 7.54 |
| Germ Cell-Sertoli Cell Junction Signaling |  |  | 6.83 |  | 5.54 |  |
| Glucocorticoid Receptor Signaling |  | 10.9 |  |  | 4.38 | 3.64 |
| Glutamate Metabolism |  |  | 17.9 |  |  | 11 |
| Glycerolipid Metabolism |  |  |  |  | 5.27 | 4.73 |
| Glycerophospholipid Metabolism |  |  | 8.36 |  | 3.65 | 3.19 |
| Glycine, Serine and Threonine Metabolism |  |  |  | 3.24 |  |  |
| GNRH Signaling |  |  | 14.7 |  | 6.99 | 2.96 |
| G-Protein Coupled Receptor Signaling |  |  |  |  | 3.39 |  |
| G Protein Signaling Mediated by Tubby | 1.36 |  |  |  |  |  |
| Gα12/13 Signaling |  |  |  |  | 7.9 |  |
| Glutamate Metabolism | 1.21 |  |  |  |  |  |
| Granzyme B Signaling | 1.26 |  |  |  |  |  |
| Hepatic Cholestasis |  |  |  |  | 3.1 |  |
| HGF Signaling |  |  |  |  | 4.59 |  |
| HIF1α Signaling |  |  |  | 18.2 | 4.82 |  |
| Histidine Metabolism |  |  |  |  | 3.78 |  |
| HMGB1 Signaling |  |  |  |  | 9.76 |  |
| Human Embryonic Stem Cell Pluripotency |  |  |  | 4.87 | 7.58 |  |
| Huntington's Disease Signaling |  |  |  |  | 3.29 |  |
| iCOS-iCOSL Signaling in T Helper Cells |  |  |  |  | 14.5 |  |
| IGF-1 Signaling |  |  |  |  | 10.1 |  |
| IL-12 Signaling and Production in Macrophages |  |  |  |  | 4.11 |  |
| IL-17 Signaling |  |  |  |  | 6.52 |  |
| IL-2 Signaling |  |  |  |  | 8.47 |  |
| IL-3 Signaling |  |  |  |  | 12.4 |  |
| IL-4 Signaling |  |  |  |  | 13.4 |  |
| IL-6 Signaling |  |  |  |  | 5.14 |  |
| ILK Signaling |  |  |  |  | 8.03 | 3.94 |
| Inositol Phosphate Metabolism | 2.75 |  |  |  |  |  |
| Insulin Receptor Signaling |  |  |  | 4.41 | 11.4 | 2.89 |
| Integrin Signaling |  |  | 5.37 |  |  | 3.26 |
| Leukocyte Extravasation Signaling |  |  |  | 2.84 |  |  |
| Linoleic Acid Metabolism |  |  |  |  | 5.27 |  |
| LPS-stimulated MAPK Signaling |  |  |  |  | 6.52 |  |
| Lysine Degradation |  |  |  |  |  | 8.96 |
| Melanocyte Development and Pigmentation Signaling |  |  |  |  | 5.48 |  |
| Mitotic Roles of Polo-Like Kinase |  |  | 5.72 |  |  |  |
| Molecular Mechanisms of Cancer |  |  | 5.25 |  | 12.4 |  |
| mTOR Signaling |  |  |  |  | 2.99 |  |
| Natural Killer Cell Signaling |  |  |  |  | 4.31 |  |
| Neuregulin Signaling |  |  |  | 6.49 |  |  |
| Neuropathic Pain Signaling In Dorsal Horn Neurons |  |  |  |  | 9.05 |  |
| Neurotrophin/TRK Signaling |  |  |  |  | 7.09 |  |
| NF-κB Signaling |  |  |  | 3.84 |  |  |
| NF-κB Signaling |  |  |  |  | 2.83 |  |
| Nicotinate and Nicotinamide Metabolism | 1.48 |  |  |  |  | 8.76 |
| Nitric Oxide Signaling in the Cardiovascular System |  |  | 11.9 |  | 6.43 |  |
| O-Glycan Biosynthesis |  |  |  |  |  | 5.2 |
| p53 Signaling |  |  | 10.5 |  | 16.3 | 4.73 |
| Pantothenate and CoA Biosynthesis |  |  |  |  |  | 6.57 |
| PDGF Signaling |  |  |  |  | 6.52 |  |
| Phospholipid Degradation |  |  | 11.4 |  | 2.06 | 5.44 |
| PI3K/AKT Signaling |  |  |  |  | 3.78 | 3.32 |
| Polyamine Regulation in Colon Cancer |  |  |  |  | 9.17 | 23.8 |
| PPARα/RXRα Activation |  |  |  |  |  | 3.86 |
| Production of Nitric Oxide and Reactive Oxygen Species in Macrophages |  |  | 6.66 |  | 5.33 |  |
| Prolactin Signaling |  |  |  |  | 6.52 |  |
| Propanoate Metabolism |  |  |  |  |  | 7.11 |
| Prostate Cancer Signaling |  |  |  |  | 5.77 |  |
| Protein Ubiquitination Pathway |  |  | 6.54 |  | 2.39 | 4.5 |
| Purine Metabolism |  | 4.86 | 6.61 | 8.08 | 3.32 | 7.25 |
| RAN Signaling | 2.22 |  |  |  |  |  |
| RAR Activation |  |  |  |  | 2.26 | 4.28 |
| Reelin Signaling in Neurons | 1.39 |  |  |  |  |  |
| Regulation of Actin-based Motility by Rho |  |  |  |  | 5.85 |  |
| Relaxin Signaling |  |  |  |  | 6.93 |  |
| Renal Cell Carcinoma Signaling |  |  |  |  | 12.6 |  |
| Renin-Angiotensin Signaling |  |  | 9.12 |  | 13.7 | 3.72 |
| RhoA Signaling | 1.84 |  |  |  |  |  |
| Riboflavin Metabolism |  |  |  | 17.6 |  | 6.17 |
| Role of BRCA1 in DNA Damage Response |  |  | 14.7 |  |  |  |
| Role of NANOG in Mammalian Embryonic Stem Cell Pluripotency |  |  |  | 5.29 | 4.11 | 3.63 |
| Role of NFAT in Regulation of the Immune Response |  |  |  |  | 4.72 |  |
| Role of Pattern Recognition Receptors in Recognition of Bacteria and Viruses |  |  |  |  | 11 | 5.22 |
| SAPK/JNK Signaling |  |  |  |  | 5.07 |  |
| Semaphorin Signaling in Neurons | 1.45 |  |  |  | 8.34 |  |
| Sonic Hedgehog Signaling |  |  |  |  | 13.2 |  |
| Sonic Hedgehog Signaling |  |  |  |  |  | 21.9 |
| Sphingolipid Metabolism |  |  |  |  | 6.61 |  |
| Sphingosine-1-phosphate Signaling |  |  |  |  | 4.42 |  |
| Starch and Sucrose Metabolism |  |  |  |  | 5.55 |  |
| Synaptic Long Term Depression |  |  | 13.6 |  |  |  |
| Synaptic Long Term Potentiation |  |  | 8.99 | 8.23 | 3.63 |  |
| Systemic Lupus Erythematosus Signaling |  |  |  |  | 16.1 |  |
| T Cell Receptor Signaling |  |  |  |  | 9.05 |  |
| Thrombin Signaling |  |  | 10.5 |  |  |  |
| Thrombopoietin Signaling |  |  |  |  | 15.2 |  |
| Tight Junction Signaling |  | 16.8 |  | 3.77 | 5.86 | 5.14 |
| Tryptophan Metabolism |  |  |  |  | 3.1 | 2.68 |
| Valine, Leucine and Isoleucine Degradation |  |  |  |  |  | 6.3 |
| VDR/RXR Activation |  |  |  |  |  | 5.44 |
| Virus Entry via Endocytic Pathways |  |  |  |  | 5.41 | 4.86 |
| Wnt/β-catenin Signaling |  |  | 6.62 |  | 9.09 |  |
| β-alanine Metabolism |  |  |  |  |  | 7.83 |
