# Supplementary material for: VENNTURE–A Novel Venn Diagram Investigational Tool for Multiple Pharmacological Dataset Analysis
Source: PLoS One. 2012 May 14;7(5):e36911. doi: 10.1371/journal.pone.0036911 (PMC3351456; doi:10.1371/journal.pone.0036911)
Supplement: Table S35 — Canonical signaling pathways populated by extracted phosphoproteins unique to non-stimulated or MeCh-stimulated control-state SH-SY5Y cells. Significantly populated canonical signaling pathways, unique to a specific stimulation condition (non-stimulated or with a specific MeCh dose) are listed. Canonical signaling pathways were considered enriched only if at least two proteins were present in each signaling pathway and with a probability of ≤0.05. Hybrid signaling pathway scores indicated were generated by multiplication of the pathway enrichment ratio with the negative log10 of the probability result. (DOC) [file pone.0036911.s036.doc]

**Table S35.** Canonical signaling pathways populated by extracted phosphoproteins unique to non-stimulated or MeCh-stimulated control-state SH-SY5Y cells. Significantly populated canonical signaling pathways, unique to a specific stimulation condition (non-stimulated or with a specific MeCh dose) are listed. Canonical signaling pathways were considered enriched only if at least two proteins were present in each signaling pathway and with a probability of ≤0.05. Hybrid signaling pathway scores indicated were generated by multiplication of the pathway enrichment ratio with the negative log_10_ of the probability result.

| **Canonical signaling pathway** |  |
| --- | --- |
|  | **Hybrid** |
| **10nM MeCh** |  |
| Ascorbate and Aldarate Metabolism | 16.7 |
| Polyamine Regulation in Colon Cancer | 10.9 |
| Mitotic Roles of Polo-Like Kinase | 10 |
| Glycolysis/Gluconeogenesis | 7.13 |
| AMPK Signaling | 4.61 |
| Metabolism of Xenobiotics by Cytochrome P450 | 4.43 |
| LPS/IL-1 Mediated Inhibition of RXR Function | 2.46 |
|  |  |
| **100nM MeCh** |  |
| FcγRIIB Signaling in B Lymphocytes | 14 |
| Fc Epsilon RI Signaling | 13.4 |
| CREB Signaling in Neurons | 13.2 |
| EGF Signaling | 12.5 |
| Estrogen Receptor Signaling | 12.3 |
| IL-12 Signaling and Production in Macrophages | 12.2 |
| Role of BRCA1 in DNA Damage Response | 11.9 |
| PI3K/AKT Signaling | 11.5 |
| Myc Mediated Apoptosis Signaling | 11 |
| GM-CSF Signaling | 10.2 |
| Activation of IRF by Cytosolic Pattern Recognition Receptors | 10.1 |
| Erythropoietin Signaling | 9.9 |
| Non-Small Cell Lung Cancer Signaling | 9.9 |
| FLT3 Signaling in Hematopoietic Progenitor Cells | 9.57 |
| IL-3 Signaling | 9.37 |
| Acute Myeloid Leukemia Signaling | 9.26 |
| PDGF Signaling | 9.26 |
| Prolactin Signaling | 9.26 |
| Aldosterone Signaling in Epithelial Cells | 8.51 |
| FGF Signaling | 8.42 |
| Starch and Sucrose Metabolism | 8.17 |
| VEGF Signaling | 8.17 |
| IGF-1 Signaling | 7.77 |
| Neuregulin Signaling | 7.77 |
| Glioma Signaling | 7.26 |
| Cholecystokinin/Gastrin-mediated Signaling | 7.05 |
| Corticotropin Releasing Hormone Signaling | 6.2 |
| Arachidonic Acid Metabolism | 5.43 |
| Synaptic Long Term Depression | 5.03 |
| IL-8 Signaling | 4 |
| Role of NFAT in Regulation of the Immune Response | 4 |
| PPARa/RXRa Activation | 3.84 |
| Thrombopoietin Signaling | 3.44 |
| G-Protein Coupled Receptor Signaling | 3.02 |
| Colorectal Cancer Metastasis Signaling | 2.55 |
|  |  |
| **1μM MeCh** |  |
| DNA Methylation and Transcriptional Repression Signaling | 20 |
| Death Receptor Signaling | 11.4 |
| Cleavage and Polyadenylation of Pre-mRNA | 10.9 |
| p38 MAPK Signaling | 8.08 |
|  |  |
| **10μM MeCh** |  |
| Nucleotide Excision Repair Pathway | 12.9 |
| Regulation of Actin-based Motility by Rho | 12.7 |
| Amyloid Processing | 9.94 |
| Cardiac β-adrenergic Signaling | 8.77 |
| Angiopoietin Signaling | 8.41 |
| Cellular Effects of Sildenafil (Viagra) | 8.09 |
| PXR/RXR Activation | 7.78 |
| Renal Cell Carcinoma Signaling | 7.68 |
| Nitric Oxide Signaling in the Cardiovascular System | 7.39 |
| Synaptic Long Term Potentiation | 4.93 |
| CCR3 Signaling in Eosinophils | 4.77 |
| Hepatic Cholestasis | 3.83 |
| cAMP-mediated Signaling | 3.01 |
|  |  |
| **10μM MeCh** |  |
|  |  |
| TREM1 Signaling | 12.3 |
| Wnt/ß-catenin Signaling | 5.25 |
