# Supplementary material for: VENNTURE–A Novel Venn Diagram Investigational Tool for Multiple Pharmacological Dataset Analysis
Source: PLoS One. 2012 May 14;7(5):e36911. doi: 10.1371/journal.pone.0036911 (PMC3351456; doi:10.1371/journal.pone.0036911)
Supplement: Table S36 — Canonical signaling pathways populated by extracted phosphoproteins unique to non-stimulated or MeCh-stimulated CMP-state SH-SY5Y cells. Significantly populated canonical signaling pathways, unique to a specific stimulation condition (non-stimulated or with a specific MeCh dose) are listed. Canonical signaling pathways were considered enriched only if at least two proteins were present in each signaling pathway and with a probability of ≤0.05. Hybrid signaling pathway scores indicated were generated by multiplication of the pathway enrichment ratio with the negative log10 of the probability result. (DOC) [file pone.0036911.s037.doc]

**Table S36.** Canonical signaling pathways populated by extracted phosphoproteins unique to non-stimulated or MeCh-stimulated CMP-state SH-SY5Y cells. Significantly populated canonical signaling pathways, unique to a specific stimulation condition (non-stimulated or with a specific MeCh dose) are listed. Canonical signaling pathways were considered enriched only if at least two proteins were present in each signaling pathway and with a probability of ≤0.05. Hybrid signaling pathway scores indicated were generated by multiplication of the pathway enrichment ratio with the negative log_10_ of the probability result.

| **Canonical signaling pathway** |  |
| --- | --- |
|  | **Hybrid** |
| **10nM MeCh** |  |
|  |  |
| **100nM MeCh** |  |
| Endothelin-1 Signaling | 18.6 |
| Role of BRCA1 in DNA Damage Response | 14.7 |
| Synaptic Long Term Depression | 13.6 |
| Thrombin Signaling | 10.5 |
| Cellular Effects of Sildenafil (Viagra) | 7.86 |
| Mitotic Roles of Polo-Like Kinase | 5.72 |
|  |  |
| **1μM MeCh** |  |
| Bladder Cancer Signaling | 6.64 |
| Neuregulin Signaling | 6.49 |
| Glycine, Serine and Threonine Metabolism | 3.24 |
| Leukocyte Extravasation Signaling | 2.84 |
|  |  |
| **10μM MeCh** |  |
| Systemic Lupus Erythematosus Signaling | 16.1 |
| Thrombopoietin Signaling | 15.2 |
| iCOS-iCOSL Signaling in T Helper Cells | 14.5 |
| IL-4 Signaling | 13.4 |
| Renal Cell Carcinoma Signaling | 12.6 |
| IL-3 Signaling | 12.4 |
| Ceramide Signaling | 11.8 |
| April Mediated Signaling | 11.1 |
| B Cell Activating Factor Signaling | 10.7 |
| IGF-1 Signaling | 10.1 |
| HMGB1 Signaling | 9.76 |
| EGF Signaling | 9.48 |
| Neuropathic Pain Signaling In Dorsal Horn Neurons | 9.05 |
| T Cell Receptor Signaling | 9.05 |
| Endometrial Cancer Signaling | 8.6 |
| IL-2 Signaling | 8.47 |
| CD28 Signaling in T Helper Cells | 8.06 |
| Angiopoietin Signaling | 7.4 |
| CD40 Signaling | 7.4 |
| Erythropoietin Signaling | 7.09 |
| Neurotrophin/TRK Signaling | 7.09 |
| Relaxin Signaling | 6.93 |
| FLT3 Signaling in Hematopoietic Progenitor Cells | 6.8 |
| Sphingolipid Metabolism | 6.61 |
| IL-17 Signaling | 6.52 |
| LPS-stimulated MAPK Signaling | 6.52 |
| PDGF Signaling | 6.52 |
| Prolactin Signaling | 6.52 |
| Chemokine Signaling | 6.34 |
| BMP signaling pathway | 6.26 |
| Aminoacyl-tRNA Biosynthesis | 6.16 |
| Regulation of Actin-based Motility by Rho | 5.85 |
| Factors Promoting Cardiogenesis in Vertebrates | 5.77 |
| Prostate Cancer Signaling | 5.77 |
| FAK Signaling | 5.55 |
| Starch and Sucrose Metabolism | 5.55 |
| Melanocyte Development and Pigmentation Signaling | 5.48 |
| Linoleic Acid Metabolism | 5.27 |
| Amyotrophic Lateral Sclerosis Signaling | 5.2 |
| IL-6 Signaling | 5.14 |
| SAPK/JNK Signaling | 5.07 |
| Chronic Myeloid Leukemia Signaling | 4.82 |
| Role of NFAT in Regulation of the Immune Response | 4.72 |
| HGF Signaling | 4.59 |
| Sphingosine-1-phosphate Signaling | 4.42 |
| Natural Killer Cell Signaling | 4.31 |
| fMLP Signaling in Neutrophils | 4.16 |
| IL-12 Signaling and Production in Macrophages | 4.11 |
| Histidine Metabolism | 3.78 |
| Actin Cytoskeleton Signaling | 3.39 |
| G-Protein Coupled Receptor Signaling | 3.39 |
| Huntington's Disease Signaling | 3.29 |
| Arachidonic Acid Metabolism | 3.25 |
| Hepatic Cholestasis | 3.1 |
| mTOR Signaling | 2.99 |
| Cardiac Hypertrophy Signaling | 2.82 |
| Eicosanoid Signaling | 2.75 |
| Dendritic Cell Maturation | 2.44 |
| cAMP-mediated Signaling | 2.36 |
|  |  |
| **100μM MeCh** |  |
| Aminosugars Metabolism | 13.1 |
| Lysine Degradation | 8.96 |
| ß-alanine Metabolism | 7.83 |
| Propanoate Metabolism | 7.11 |
| Pantothenate and CoA Biosynthesis | 6.57 |
| Valine, Leucine and Isoleucine Degradation | 6.3 |
| VDR/RXR Activation | 5.44 |
| O-Glycan Biosynthesis | 5.2 |
| PPARa/RXRa Activation | 3.86 |
| Fatty Acid Metabolism | 2.75 |
